# Disease Burden and Geographic Inequalities in 15 Types of Neonatal Infectious Diseases in 131 Low- and Middle-Income Countries and Territories

**DOI:** 10.34133/hds.0186

**Published:** 2024-10-01

**Authors:** Chenyuan Qin, Qiao Liu, Yaping Wang, Jie Deng, Min Du, Min Liu, Jue Liu

**Affiliations:** ^1^ School of Public Health, Peking University, Beijing, 100191, China.; ^2^ Institute for Global Health and Development, Peking University, Beijing, 100871, China.; ^3^ National Health Commission Key Laboratory of Reproductive Health, Peking University, Beijing, 100191, China.; ^4^ Peking University Health Science Center-Weifang Joint Research Center for Maternal and Child Health, Peking University, Beijing, 100191, China.

## Abstract

**Background:** The burden of neonatal infections in low- and middle-income countries and territories (LMICs) is a critical public health challenge, while our understanding of specific burden and secular trends remains limited. **Methods:** We gathered annual data on 15 types of neonatal infections in LMICs from 1990 to 2019 from the Global Burden of Disease 2019. Numbers, rates, percent changes, and estimated annual percentage changes of incidence and deaths were calculated. We also explored the association between disease burden, socio-demographic index (SDI), and universal health coverage index (UHCI). **Results:** Enteric infections and upper respiratory infections owned the top highest incidence rates for neonates in 2019. Neonatal sepsis and other neonatal infections, as well as otitis media, demonstrated an increasing trend of incidence across all 3 low- and middle-income regions. The top 3 causes of neonatal mortality in 2019 were neonatal sepsis and other neonatal infections, lower respiratory infections, and enteric infections. Between 1990 and 2019, all of the neonatal infection-related mortality rates suggested an overall decline. Sex differences could be found in the incidence and mortality of some neonatal infections, but most disease burdens decreased more rapidly in males. SDI and UHCI were both negatively associated with most of the disease burden, but there were exceptions. **Conclusions:** Our study serves as a vital exploration into the realities of neonatal infectious diseases in LMICs. The identified trends and disparities not only provide a foundation for future research but also underscore the critical need for targeted policy initiatives to alleviate on a global scale.

## Introduction

The first month of life represents a child’s most delicate and crucial phase for survival, and about 47% of all deaths among children under 5 occurred in the neonatal period in 2022 [1]. Neonatal infections is regard as one of the leading causes of neonatal deaths [1]. Neonatal infections refer to a spectrum of health issues in infants within the first 28 d of life caused by pathogens such as bacteria, viruses, and fungi, encompassing both communicable and noncommunicable diseases [2]. The underdeveloped nature of immune system makes them particularly vulnerable to various pathogenic microorganisms [3,4]. Besides, factors such as maternal or neonatal malnutrition, maternal transmission, unhygienic birthing conditions, prematurity, low birth weight, inappropriate use of antibiotics, and poor housing conditions all serve as potential threats of infection in newborns, especially in low- and middle-income countries and territories (LMICs) [2,4].

Despite advancements in neonatal medicine, infectious diseases in neonates, such as neonatal sepsis, enteric infections, upper respiratory infections (URIs), lower respiratory infections (LRIs), and tetanus, continue to be the major causes of morbidity and/or mortality [5–9]. Neonatal sepsis, characterized by nonspecific signs and symptoms resulting from pathogen invasion, exhibits an incidence of 1 to 4 cases per 1,000 live neonates in high-income countries, but 49 to 170 cases per 1,000 live neonates contrastingly in LMICs, with a mortality rate as high as 24% [3,10–15]. Those survivors of neonatal sepsis may face an elevated risk of negative outcomes related to neurodevelopment [16]. Diarrheal diseases, a common set of enteric infections, are major health concerns responsible for approximately 8.6% of pediatric deaths worldwide [17]. LRIs constitute a main cause of global mortality, causing over 2 million deaths annually, particularly among children under 5 years of age [18,19]. Additionally, evidence from the Global Burden of Disease (GBD) study reported that the URIs had exceeding 250,000 cases per 100,000 individuals under the age of 10 in 2019 [7]. In 2019, the global hepatitis B surface antigen prevalence among infants and children under the age of 5 was reported at 1.0% [20]. Strengthening prevention and treatment measures for neonatal infectious diseases is crucial for improving the survival and quality of life of neonates in LMICs. Aligned with the Sustainable Development Goals, by 2030, all nations are expected to eliminate preventable deaths of newborns and lower the rate of neonatal mortality to a maximum of 12 per 1,000 live births [21].

In the realm of global public health, narrowing the gap between high-income regions and LMICs in the disease burden of neonatal infectious diseases among newborns is still under way. In this study, we aimed to display the current epidemic status and secular trends (1990 to 2019) of 15 common neonatal infectious diseases (post birth within 28 d) in LMICs at the regional and national levels. Then, we also explored the associations between the burden of neonatal infections and socioeconomic and universal health coverage status. Our study may complement previous evidences for achieving Sustainable Development Goals and holds the promise of enhancing the provision of high-quality healthcare services and health security for neonates on a global scale.

## Methods

### Study design and data sources

The GBD database comprehensively assesses epidemiological trends and disease burden for 369 diseases and injuries across 204 countries and territories from 1990 to 2019 [18]. Disease burden estimation utilizes standardized tools detailed in previous studies and the Supplementary Materials [18,22]. We collected yearly data on 15 types of infectious diseases among neonates (0 to 28 d) in LMICs between 1990 and 2019 at country and regional levels from GBD 2019 query tool [18]. The compilation of the ultimately included list of 131 LMICs was guided by the World Bank Group country classifications 2019, including 29 low-income countries and territories (LICs), 48 lower-middle-income countries and territories (LMCs) and 54 upper-middle-income countries and territories (UMCs) in this study (Table S1) [23,24]. Socio-demographic index (SDI) from 1990 to 2019 and universal health coverage index (UHCI) in 2019 were also acquired from the GBD query tool [25,26].

### Neonatal infectious diseases (post birth within 28 d)

Neonatal infectious diseases can be caused by a variety of pathogens, including bacteria, viruses, fungi, and parasites, affecting newborns in the first 28 d of life (the neonatal period) [2]. A total of 15 common infectious diseases (acute hepatitis, bacterial skin diseases, encephalitis, enteric infections, fungal skin diseases, LRIs, meningitis, neglected tropical diseases and malaria, neonatal sepsis and other neonatal infections, otitis media, scabies, tetanus, tuberculosis, URIs, and varicella and herpes zoster) during the neonatal period were included in this study. All diseases were classified based on the *International Classification of Diseases Tenth Revision* [18]. The definition and specific details are shown in the Supplementary Materials. In this study, we retrieved annual data on the incident cases and incidence rate (per 100,000 live-born neonates), death cases, and mortality rates (per 100,000 live-born neonates) of each disease among newborns (0 to 28 d) between 1990 and 2019 for both sex. Mortality data on acute hepatitis, bacterial skin diseases, fungal skin diseases, scabies, and tuberculosis were not included. To ascertain the uncertainty of each metric, 95% uncertainty intervals were calculated from 1,000 samples of the posterior distribution, using the 25th and 975th ordered values as the thresholds.

### SDI and UHCI

The SDI, ranging from 0 to 1, functions as a composite measure tightly linked to economic development status, derived from the geometric mean of 3 factors: total fertility rate for individuals younger than 25, mean education for individuals aged 15 and over, and lag distributed income per capita [26,27]. The UHCI is composed of 23 indicators that are distributed among 5 domains of health services, involving promotion, prevention, treatment, rehabilitation, and palliation, representing the average coverage of primary healthcare services [25,28]. The UHCI varies from 0 to 100, along with an increasing number of high-quality health services [25,28]. Table S2 shows the SDIs and UHCIs of 131 LMICs in 2019.

### Statistical analysis

We performed absolute incidences and deaths with 95% uncertainty intervals delineating the prevailing status of specific neonatal infections in individual nations and 3 low- and middle-income regions. The relative percent change was calculated utilizing the formula: (Numbers in 2019 − Numbers in 1990)/Numbers in 1990 × 100%. Estimated annual percentage change (EAPC) was determined through a regression model, where Y represented the natural logarithm of the rate (e.g., incidence rates or mortality rates), X denoted the calendar year, ε signified the error term, and β represented the directional trend [7]. The EAPC was derived as 100*(exp(β) − 1). An upward trend of incidence or mortality was discerned when the EAPC and its lower 95% confidence interval (CI) boundary were positive, while the downward trend was identified by negative values for both the EAPC and its upper 95% CI boundary. When these criteria were not met, morbidity and mortality remain stable over time. Spearman rank correlation analyses were employed to explore associations of the burden of neonatal infectious diseases with SDI and UHCI in LMICs. Additionally, we also utilized polynomial curves to model observed trends. All statistical analyses were executed using R version 4.2.2. Two-tailed *P* values of less than 0.05 were considered to be statistically significant.

## Results

### Regional trends of neonatal infectious diseases in incidence among LICs, LMCs, and UMCs

As detailed in Table S3, in 2019, the highest incidence rates of neonatal infectious diseases in LICs were observed in enteric infections (348,418.42 per 100,000 live-born neonates) and URIs (292,946.03). URIs (LMCs: 284,464.85; UMCs: 319,174.17) surpassed enteric infections with the highest incidence rate in both LMCs and UMCs (Table S3). Surprisingly, the incidence rates of neonatal sepsis and other neonatal infections, as well as otitis media, demonstrated slightly increasing trends across all of 3 low- and middle-income regions (Table 1 and Fig. S1). Among those 3 regions, only LMCs reported an uptrend in the incidence rate of bacterial skin diseases (EAPC = 0.17%). Except for UMCs, where the incidence rate of URIs remained stable, the incidence rates of all other neonatal infections all exhibited downtrends between 1990 and 2019 (all EAPCs <0). Importantly, the EAPC of tetanus in incidence rate even decreased by more than 5%, with the most remarkable decrease in UMCs (EAPC = −17.96%). Furthermore, the substantial decline in the incidence rates of tuberculosis and LRIs in UMCs was noteworthy.

**Table 1.** EAPCs of incidence rates (per 100,000 live-born neonates) of neonatal infectious diseases in 131 LMICs from 1990 to 2019

Neonatal infections	LICs 1990–2019	LMCs 1990–2019	UMCs 1990–2019
	EAPC (%, 95% CI)	EAPC (%, 95% CI)	EAPC (%, 95% CI)
Acute hepatitis	−0.42 (−0.47 to −0.36)	−1.06 (−1.17 to −0.96)	−1.28 (−1.37 to −1.18)
Bacterial skin diseases	0.01 (−0.002 to 0.03)	0.17 (0.16 to 0.17)	−0.11 (−0.19 to −0.04)
Encephalitis	−0.84 (−0.90 to −0.78)	−1.58 (−1.69 to −1.47)	−1.3 (−1.40 to −1.21)
Enteric infections	−0.13 (−0.16 to −0.11)	−0.44 (−0.64 to −0.23)	−1.74 (−1.90 to −1.58)
Fungal skin diseases	−0.16 (−0.20 to −0.12)	−0.36 (−0.38 to −0.34)	−0.16 (−0.19 to −0.13)
Lower respiratory infections	−2.12 (−2.23 to −2.00)	−2.17 (−2.37 to −1.97)	−5.18 (−5.35 to −5.01)
Meningitis	−2.69 (−3.00 to −2.37)	−2.04 (−2.35 to −1.73)	−3.53 (−3.93 to −3.14)
Neglected tropical diseases and malaria	−2.01 (−2.34 to −1.68)	− 1.39 (−1.61 to −1.16)	−1.49 (−1.89 to −1.09)
Neonatal sepsis and other neonatal infections	0.30 (0.23 to 0.36)	0.06 (0.02 to 0.09)	1.34 (1.26 to 1.42)
Otitis media	0.04 (0.04 to 0.05)	0.05 (0.05 to 0.06)	0.25 (0.13 to 0.36)
Scabies	−0.49 (−0.52 to −0.47)	−0.29 (−0.34 to −0.23)	−0.31 (−0.38 to −0.23)
Tetanus	−7.12 (−7.26 to −6.97)	−9.25 (−9.55 to −8.95)	−17.96 (−18.68 to −17.22)
Tuberculosis	−2.65 (−2.72 to −2.57)	−2.61 (−2.69 to −2.53)	−5.14 (−5.54 to −4.73)
Upper respiratory infections	−0.33 (−0.39 to −0.27)	−0.27 (−0.41 to −0.12)	0.00 (−0.07 to 0.06)
Varicella and herpes zoster	−0.59 (−0.63 to −0.55)	−0.30 (−0.33 to −0.26)	−0.42 (−0.45 to −0.40)

CI, confidence interval; EAPC, estimated annual percentage change; LICs, low-income countries and territories; LMCs, lower-middle-income countries and territories; LMICs, low- and middle-income countries and territories; UMCs, upper-middle-income countries and territories.

Table S4 reported the percent changes of incident cases between 1990 and 2019. Apparently, the new cases of tetanus, tuberculosis, LRIs, and meningitis all declined markedly between 1990 and 2019 across 3 regions, while the number of new cases in neonatal sepsis and other neonatal infections, bacterial skin diseases, and otitis media increased both in LICs and LMCs. In UMCs, almost all neonatal infectious diseases were witnessed a decrease in new cases.

### Regional trends of neonatal infectious diseases in death among LICs, LMCs, and UMCs

As shown in Table S5, the highest mortality rates were occurred in neonatal sepsis and other neonatal infections (LICs: 3,974.60; LMCs: 2,382.85; UMCs: 721.32) and LRIs (LICs: 2,626.91; LMCs: 2,820.08; UMCs: 355.61). Compared with LICs and LMCs, UMCs exhibited lower neonatal mortality rates. As for the regional trends between 1990 and 2019, the estimated annual changes of 10 diseases across 3 regions all showed the downward trends (Table 2 and Fig. S2). Specifically, the most remarkable decreases were found in tetanus (LICs: −6.90%; LMCs: −9.26%; UMCs: −17.22%), enteric infections (LICs: −4.49%; LMCs: −5.68%; UMCs: −9.13%), and URIs (LICs: −4.71%; LMCs: −4.46%; UMCs: −9.43%).

**Table 2.** EAPCs of mortality rates (per 100,000 live-born neonates) of neonatal infectious diseases in 131 LMICs from 1990 to 2019

Neonatal infections	LICs 1990–2019	LMCs 1990–2019	UMCs 1990–2019
	EAPC (%, 95% CI)	EAPC (%, 95% CI)	EAPC (%, 95% CI)
Encephalitis	−1.23 (−1.32 to −1.15)	−1.81 (−1.92 to −1.70)	−2.87 (−3.16 to −2.59)
Enteric infections	−4.49 (−4.85 to −4.14)	−5.68 (−5.93 to −5.42)	−9.13 (−9.41 to −8.85)
Lower respiratory infections	−3.58 (−3.74 to −3.41)	−3.31 (−3.43 to −3.19)	−6.85 (−7.12 to −6.58)
Meningitis	−3.65 (−4.01 to −3.28)	−2.20 (−2.55 to −1.84)	−6.14 (−6.29 to −5.98)
Neglected tropical diseases and malaria	−2.97 (−3.45 to −2.49)	−1.58 (−2.05 to −1.10)	−2.68 (−2.90 to −2.46)
Neonatal sepsis and other neonatal infections	−0.54 (−0.65 to −0.43)	−1.14 (−1.25 to −1.03)	−1.03 (−1.41 to −0.65)
Otitis media	−3.90 (−4.03 to −3.77)	−11.96 (−12.48 to −11.44)	−7.71 (−9.20 to −6.19)
Tetanus	−6.90 (−7.14 to −6.67)	−9.26 (−9.56 to −8.96)	−17.22 (−17.88 to −16.54)
Upper respiratory infections	−4.71 (−4.89 to −4.52)	−4.46 (−4.60 to −4.31)	−9.43 (−9.76 to −9.09)
Varicella and herpes zoster	−2.41 (−2.51 to −2.31)	−2.68 (−2.76 to −2.60)	−4.40 (−4.60 to −4.20)

It is worth noting that only the deaths of neonatal sepsis and other neonatal infections in LICs increased by 32.15% from 55,425 in 1990 to 73,244 in 2019 (Table S6). LRIs, meningitis, enteric infections, and tetanus all had a negative percent change of death cases over 40% between 1990 and 2019 in LICs. Almost all categories of deaths showed downward trends in LMCs. UMCs demonstrated remarkable success in reducing neonatal infection-related deaths, with reductions ranging from 42.77% to 99.46% (Table S6).

### National trends in incidence of neonatal infectious diseases

The national burden and trends of incidence rates of 15 neonatal infectious diseases among 131 LMICs are shown in Tables S7 to S21. Bangladesh, Peru, and the Dominican Republic faced the most severe burden of neonatal sepsis and other neonatal infections of more than 130,000 per 100,000 live-born neonates in 2019, and more than half of 131 LMICs experienced remarkable upward trends in the incidence rates of neonatal sepsis and other neonatal infections over the past 3 decades, especially in North Macedonia (EAPC = 8.41%) and Bulgaria (EAPC = 6.25%) (Table S15). The acute hepatitis had high incidence rates exceeding 50,000 per 100,000 live-born neonates in 17 LMICs in 2019, such as South Sudan, Somalia, and Lesotho. Except for Thailand (EAPC = 1.39%), Papua New Guinea (EAPC = 0.53%), Bulgaria (EAPC = 0.16%), Vanuatu (EAPC = 0.15%), and Ecuador (EAPC = 0.10%), almost all other LMICs were observed negative EAPCs in the incidence rates of acute hepatitis, ranging from −0.10% in Afghanistan to −2.12% in Egypt (Table S7). The incidence rates of enteric infections in 2019 ranged from 373,339.47 per 100,000 live-born neonates (Sudan) to 155,145.02 per 100,000 live-born neonates (China), with the downtrends over 3% annually from 1990 to 2019 in Kazakhstan, China, Uzbekistan, and Ukraine (Table S10).

As for the bacterial skin diseases in neonates, its incidence rates steadily declined in 5 LMICs (Mexico, Egypt, Nepal, China, and Honduras) from 1990 to 2019, while they increased in 120 LMICs (all EAPCs >0) (Table S8). Notably, LRIs had decreased incidence rates in 127 LMICs, ranging from −0.11% in Dominica to −6.76% in China (Table S12). The EAPC of the incidence rate of otitis media showed relatively small fluctuations in both upward and downward directions across countries and territories, with the exception of Thailand (EAPC = 0.09%) (Table S16). Concerns also raised from the incidence of neonatal tetanus in Somalia (2,576.56 per 100,000 live-born neonates), Nepal (1,281.56 per 100,000 live-born neonates), and Chad (1,206.64 per 100,000 live-born neonates) (Table S18). National incidence rates of URIs were highest in the Central African Republic (532,068.18 per 100,000 live-born neonates) and Thailand (506,125.99 per 100,000 live-born neonates) (Table S20). Most countries exhibited a fast decline in URIs, encephalitis, fungal skin diseases, meningitis, scabies, tetanus, tuberculosis, and varicella and herpes zoster, with a few exceptions. Additionally, the number of new cases and the percent change in 1990 and 2019 for the 131 LMICs are shown simultaneously in Tables S7 to S21.

### National trend in mortality of neonatal infectious diseases

The national burden and trends of death cases and mortality rates in 131 LMICs are exhibited in Tables S22 to S31. In Mali, approximately 8,541.00 per 100,000 newborns (0 to 28 d) died due to neonatal sepsis and other neonatal infections, with similar high mortality burdens observed in Ghana and Burkina Faso. Many LMICs such as Jamaica, Dominica, and South Africa still faced a heavy and increasing mortality burden of neonatal sepsis (Table S27). In 2019, the mortality rate of enteric infections reached the highest values at 3,088.13 per 100,000 live-born neonates in the Central African Republic and 2,997.91 per 100,000 live-born neonates in Chad. A total of 10 countries such as Malaysia, Turkey, and China reported mortality rates below 10.00 per 100,000 live-born neonates for enteric infections (Table S23). Concerning LRIs, Nigeria (6,320.73 per 100,000 live-born neonates) and the Central African Republic (5,708.07 per 100,000 live-born neonates) had the highest mortality rates (Table S24). Between 1990 and 2019, the neonatal mortality rates for enteric infections and LRIs both experienced declines in almost all nations, with upward trends of enteric infections in Serbia (EAPC = 0.82%) and increasing mortalities of LRIs in Dominica (EAPC = 1.02%), Venezuela (Bolivarian Republic of) (EAPC = 1.30%), and Guatemala (EAPC = 0.93%) (Tables S23 and S24). In addition, a total of 8 countries, such as the Democratic Republic of the Congo, Guinea, and Côte d’Ivoire, still faced a heavy mortality burden contributed to neglected tropical diseases and malaria in 2019, with mortality rates exceeding 1,000.00 per 100,000 live-born neonates. In addition, nations with high burden of neonatal deaths showed clear downward trends in mortality rates, such as Democratic Republic of the Congo (EAPC = −2.66%), Guinea (EAPC = −0.53%), Côte d’Ivoire (EAPC = −2.95%), Liberia (EAPC = −2.64%) , and Sierra Leone (EAPC = −2.33%) (Table S26).

Moreover, the risk of neonatal death due to tetanus in 2019 remained noteworthy, especially in Somalia, where the mortality rate was 2,011.97 per 100,000 live-born neonates, despite substantial variations among countries (Table S29). Undoubtedly, over the past 30 years, the neonatal mortality rates for encephalitis, meningitis, otitis media, tetanus, URIs, and varicella and herpes zoster in most LMICs decreased by nearly or more than 1%, while the neonatal mortality burden in 2019 for these infectious diseases were relatively low in LMICs and remained considerably lower than for other diseases (Tables S22, S25, S28, S30, and S31).

### Sex differences of neonatal infectious diseases among LICs, LMCs, and UMCs

**Fig. 1.**
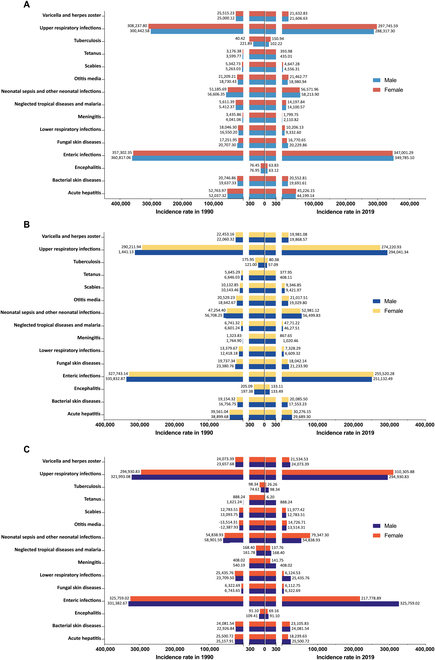
The incidence rates of 15 neonatal infectious diseases by sex across 3 regions in 1990 and 2019. (A) Sex differences of neonatal infectious diseases among LICs. (B) Sex differences of neonatal infectious diseases among LMCs. (C) Sex differences of neonatal infectious diseases among UMCs.

Across the 3 low- and middle-income regions, the incidence rates of enteric infections and URIs were the highest among both males and females in 1990 and 2019, which is much higher than other neonatal infectious diseases (Fig. 1). Of these, only LICs had a slightly higher incidence of URIs per 100,000 live-born neonates in females than in males (308,237.80 versus 300,442.58 in 1990; 297,745.59 versus 288,317.30 in 2019). However, regarding the incidence rates of 13 other infectious neonatal diseases in 2019, tetanus, neonatal sepsis and other neonatal infections, meningitis, and fungal skin diseases all exhibited higher incidence rates among males in LICs and LMICs. Scabies presented a higher incidence rate in males within the LMICs. Furthermore, in UMCs, only otitis media, neonatal sepsis and other neonatal infections, and meningitis had a higher incidence rate among females compared to males (Fig. 1). As for the EAPC (Table S32 and Figs. S3 and S4), only the otitis media showed an increasing trend in both males and females across all regions (all EAPCs > 0). The incidence rate of neonatal sepsis and other neonatal infections in males only declined at LMC between 1990 and 2019 (EAPC = −0.12%). In most of the neonatal infections with decreasing incidence, males usually had the faster decline (Table S32 and Figs. S3 and S4).

**Fig. 2.**
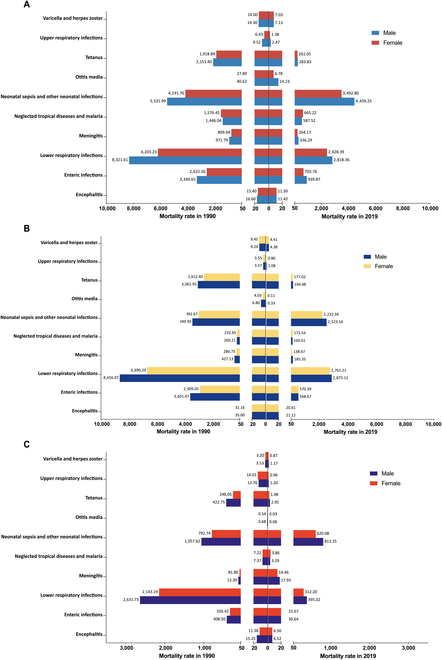
The mortality rates of 15 neonatal infectious diseases by sex across 3 regions in 1990 and 2019. (A) Sex differences of neonatal infectious diseases among LICs. (B) Sex differences of neonatal infectious diseases among LMCs. (C) Sex differences of neonatal infectious diseases among UMCs.

As for the mortality rates among 3 low- and middle-income regions, both in 1990 and 2019, neonatal sepsis and other neonatal infections, along with LRIs, were the leading causes of death among both males and females, with males experiencing a higher mortality rate (Fig. 2). Only the female mortality rates of neglected tropical diseases and malaria in LICs and LMCs were markedly higher than males across all diseases and regions (Fig. 2). Most neonatal infectious diseases decreased more rapidly in males, especially in LMC and UMC (Table S33 and Figs. S5 and S6).

### Factors associated with the burden of neonatal infectious diseases

**Fig. 3.**
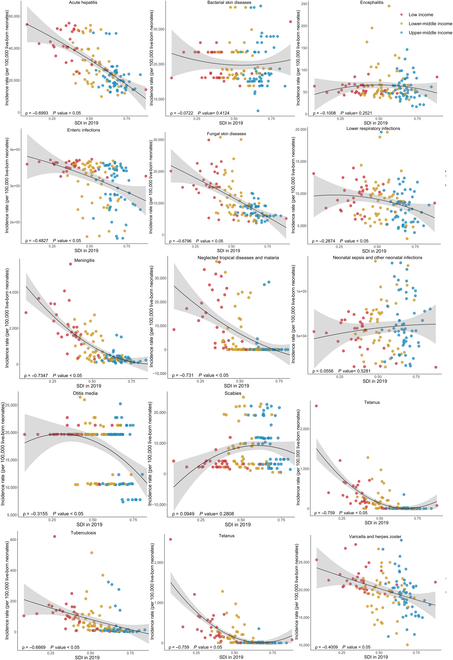
The correlation between the incidence rates of neonatal infectious diseases and SDI in 2019 among 131 LMICs.

**Fig. 4.**
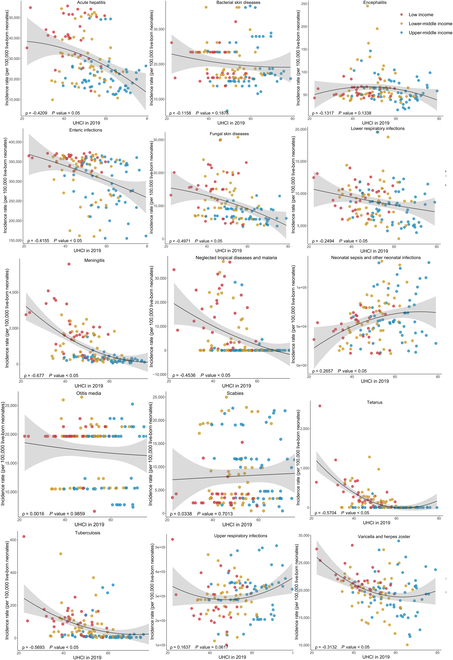
The correlation between the incidence rates of neonatal infectious diseases and UHCI in 2019 among 131 LMICs.

**Fig. 5.**
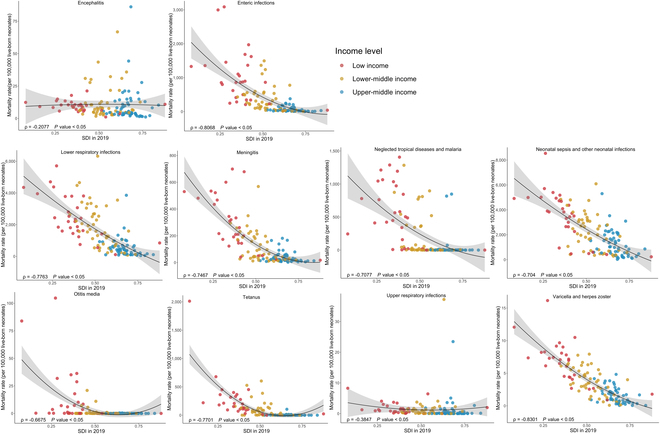
The correlation between the mortality rates of neonatal infectious diseases and SDI in 2019 among 131 LMICs.

**Fig. 6.**
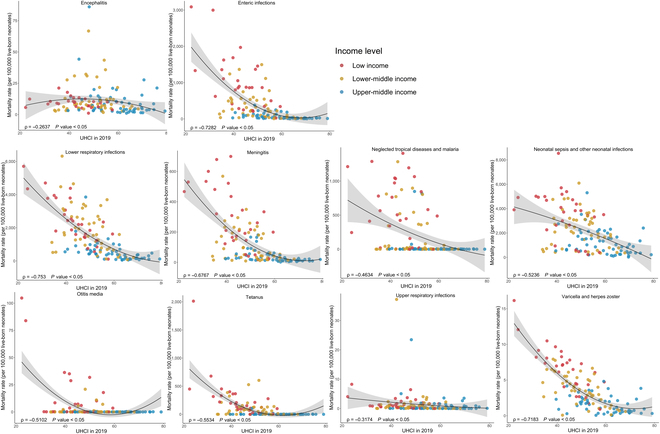
The correlation between the mortality rates for 10 types of infectious diseases among newborns (0 to 28 d) and UHCI in 2019 among 131 LMICs.

As is shown in Fig. 3, generally, the incidence rates of acute hepatitis, enteric infections, fungal skin diseases, LRIs, meningitis, neglected tropical diseases and malaria, otitis media, tetanus, tuberculosis, and varicella and herpes zoster all significantly decreased with the increase of SDI in 2019 (all ρ < 0, *P* < 0.05). Similar patterns were observed between the incidence rates and the UHCI in 2019, except for otitis media (Fig. 4). Surprisingly, with the growth of UHCI in 2019, the incidence rate of neonatal sepsis and other neonatal infections increased (ρ = 0.2657, *P* < 0.05). In 131 LMICs, the neonatal mortality rates of all 10 infectious diseases were negatively correlated with SDI and UHCI in 2019, detailed in Figs. 5 and 6 (all ρ < 0, *P* < 0.05).

## Discussion

To our knowledge, this is the first study to focus on the epidemic status and 30-year long-term secular trends of incidence and mortality of multiple neonatal infectious diseases. Compared with LICs and LMCs, UMCs exhibited markedly lower diseases burden. Enteric infections, URIs, and neonatal sepsis and other neonatal infections among the population aged 0 to 28 d had the top 3 highest incidence rates in 3 low- and middle-income regions in 2019. A vast majority of neonatal infections across those 3 regions have exhibited substantial downward trends of incidence from 1990 to 2019, with the most remarkable decrease in tetanus in UMCs. Surprisingly, incidence rates of neonatal sepsis and other neonatal infections, as well as otitis media, slightly increased across the areas of LICs, LMCs, and UMCs. The mortality rates of neonatal sepsis and other neonatal infections and LRIs were much higher than that of other diseases. The estimated annual changes of mortality rates for 10 diseases across 3 regions all showed the downward trends. There are notable sex differences in the incidence and mortality of some neonatal infectious diseases across regions, but most disease burdens decreased more rapidly in males. Across 131 LMICs, national trends of incidence and deaths for different neonatal infectious diseases varied widely. Higher SDI and UHCI were generally correlated with reduced incidence and mortality rates for the majority of diseases. However, exceptions, such as increased neonatal sepsis and other neonatal infections with UHCI growth, highlighted the nuanced relationship.

Enteric infections and URIs exhibited the highest neonatal incidence rates in 2019 across 3 low- and middle-income regions. Multifaceted reasons, such as maternal malnutrition, unsafe water and sanitation facilities, inadequate hygiene practices, deficient healthcare infrastructure, and social instability, may cause the high incidence of enteric infections, especially for typical countries like Sudan, Niger, Afghanistan, and Somalia in LICs [6,17,29–35]. These factors collectively create an environment conducive to the proliferation and transmission of enteric pathogens—i.e., rotavirus, *Cryptosporidum* *spp.*, *Shigella* *spp*., and enterotoxigenic *E. coli* producing heat-stable toxin, particularly among newborns [36]. Socioeconomic inequality, coupled with challenges in preventive measures, further increases the vulnerability of newborns to these infections [6]. Prolonged diarrhea, dehydration, and malnutrition resulting from enteric infections may lead to long-term developmental problems, increasing the burden of morbidity and mortality among children in resource-constrained areas [11,31–34,37]. Concerning the widespread URIs in LMICs, aside from the similar risk factors already mentioned above in enteric infections, air pollution and crowded living environments also heighten the risk of respiratory pathogen transmission [33,35,38–40]. Soesanti et al. found that perinatal exposure to fine particles may increase the risk of respiratory infections of infants less than 6 months old, especially URIs [40]. Moreover, raising educational level and health education of the adults emerge as crucial factors in reducing the incidence of URIs and other infections [41]. Countries like the Central African Republic, Thailand, Nigeria, India, and Bangladesh consistently report URIs as one of the most common health issues among newborns in our research. Undoubtedly, similar constraints in healthcare resources, adverse living conditions, and relatively lower education levels contribute to the occurrence of infectious diseases such as acute hepatitis, neonatal sepsis, skin infections, and meningitis in newborns in several LMICs [15,18,42–44].

According to our results, compared with other infectious diseases, neonatal sepsis and LRIs stood out as the primary infectious causes of mortality in LMICs. Annually, about 4 million infants died in the first 28 d of life, and about one-third of all neonatal deaths were attributable to sepsis [45]. LRIs are the leading cause of sepsis and contribute to a substantial global burden of health loss and mortality [46]. International efforts aimed at addressing LRIs encompass various strategic initiatives [47]. Importantly, the lack of both adequate healthcare services and high-quality maternal and newborn health care may be perhaps the most noteworthy contributors to the high mortality rates [47–49]. The high mortality rates are reflective of the complex interplay between healthcare accessibility, resource distribution, and education levels within these regions [41,47].

The dynamic nature of neonatal infectious diseases is evident in the changing patterns between 1990 and 2019. Neonatal sepsis and other neonatal infections and otitis media showed increased incidence across all regions in our research, and LICs and LMCs report a slight rise of incidence rate in bacterial skin diseases. Among the multitude of factors, some warrant particular emphasis for discussion. Firstly, inappropriate antibiotic use is a current hot issue worth exploring. Associations have been observed between clinical procedures including the application of antibiotics during pregnancy and delivery via caesarean section, with instances of sepsis in newborns [44]. This link may be attributed to alterations in the mother’s vaginal microbiome and a heightened risk of bacterial exposure that can lead to sepsis [44]. Antibiotic misuse can generate antibiotic-resistant strains, making these pathogens more likely to cause skin infections [50]. Secondly, immunization remains a crucial public health intervention [9]. Pathogens like respiratory syncytial virus, *Streptococcus* *pneumoniae*, and *Hemophilus* *influenzae* can cause otitis media, particularly in infants and toddlers [51]. Delayed preventive vaccinations may also promote to increased otitis media incidence [51]. In addition, although the notable decline in tetanus incidence is commendable, challenges persist in registering cases born and deceased at home, rendering the true burden unknown [52]. Examining national trends provides a more granular understanding of the challenges and triumphs.

Studies have indicated that intrinsic biological differences in immune system between male and female infants could correlate with variations in their susceptibility to infections [53,54]. Generally, the mortality rate of infectious diseases is higher in males than in females, often attributing to the more robust immune response in females [55]. However, these differences are not consistently observed across all types of infections or age groups. Moreover, our research has discovered a more pronounced annual decline in the incidence and mortality rates of infectious diseases among male neonates compared to females from 1990 to 2019. Beyond the intrinsic biological distinctions, part of this trend may be ascribed to social and cultural factors, such as the propensity for boys to receive greater access to healthcare resources in many low- and middle-income regions [56].

Regarding the negative correlation between SDI and UHCI with incidence and mortality rates, our findings underscore the importance of interventions considering socioeconomic factors and health system coverage to improve neonatal health outcomes [26,28,57,58]. Surprisingly, the positive correlation between UHCI and the incidence rate of neonatal sepsis and other neonatal infections warrants further exploration to understand the complex interplay of healthcare infrastructure, access, and disease dynamics. The incidence of such diseases in LICs, LMCs, and UMCs demonstrated slightly increasing trends between 1990 and 2019, with UMCS having the highest incidence as 80,265.09 per 100,000 live-born neonates in 2019. This association may stem from improved reporting, heightened survival rates, increased medical interventions for high-risk populations, and regional variations in healthcare practices [25,48,59]. Progress in the fields of sepsis prevention, screening, early diagnosis, and treatment has improved the timely identification and management of neonates susceptible to sepsis [60]. However, it cannot be ruled out that specific countries or populations may still face health challenges leading to increased rates of neonatal infection [61]. According to our results, neonatal mortality of neonatal sepsis and other neonatal infections decreased with increasing UHCI, which was in line with our expectations. While a high health coverage index does not directly cause the increased neonatal infection, it may indicate enhanced surveillance and healthcare services for pregnant women and newborns to some extent [62–64]. A detailed understanding would necessitate thorough research into the unique circumstances of each country.

Addressing neonatal infections in LMICs requires a collaborative approach geared toward specific challenges, including economic constraints, fragile healthcare systems, and postconflict conditions [65,66]. Emphasis should be placed on financial and technical assistance to bolster healthcare resilience. Moreover, adaptable global health policies and strategic resource allocation by local governments are necessary to enhance healthcare facilities and train medical staff [65,66]. Disease-specific health policy tailoring and the adaptation of diagnostic and treatment protocols at the local level are vital [67]. Implementing technological solutions and robust monitoring for disease management, alongside customized maternal health consultations and adherence to postnatal care guidelines, is imperative [68,69]. Furthermore, promoting vaccination adherence is essential to newborn immunity in these challenging settings [70,71].

We have to acknowledge certain limitations inherent. First, GBD relies on diverse data sources, including surveys and healthcare systems, introducing challenges such as underreporting and incomplete data, particularly in LMICs with limited healthcare infrastructure. Second, GBD’s periodic updates may cause temporal gaps, impacting data relevance in dynamic settings, like postconflict regions or rapidly developing economies. Furthermore, our study, focusing on GBD’s level-2 disease classifications, may not fully capture specific pathogens, antimicrobial resistance variations, or emerging threats, crucial for tailored prevention and treatment strategies. Researchers and policymakers should interpret results cautiously, considering complementary data sources and qualitative research for a nuanced understanding. Addressing these limitations is vital for precise and context-specific public health interventions, enhancing global efforts to improve neonatal health.

Our study serves as a vital exploration into the realities of neonatal infectious diseases in LMICs, emphasizing the urgency for tailored interventions that consider the unique challenges faced by these nations. The identified trends and disparities not only provide a foundation for future research but also underscore the critical need for targeted policy initiatives to alleviate the burden of neonatal infectious diseases on a global scale.

## Data Availability

All data in the study are available at https://ghdx.healthdata.org/gbd-2019.
