# Supplementary material for: Disease Burden and Geographic Inequalities in 15 Types of Neonatal Infectious Diseases in 131 Low- and Middle-Income Countries and Territories
Source: Health Data Sci. 2024 Oct 1;4:0186. doi: 10.34133/hds.0186 (PMC11443844; doi:10.34133/hds.0186)
Supplement: Supplementary 1 — Figs. S1 to S6 Tables S1 to S33 [file hds.0186.f1.docx]

Supplementary Materials

**Disease burden and geographic inequalities in 15 types of neonatal infectious diseases in 131 low- and middle-income countries and territories (LMICs)**

CONTENTS

[Supplementary Materials 5](#_Toc168780411)

[Supplementary Methods 5](#_Toc168780412)

[1. GBD Overview 5](#_Toc168780413)

[2. Disease Definition 6](#_Toc168780414)

[Supplementary Figures 16](#_Toc168780415)

[Supplementary Figure S1. Incidence rates of neonatal infectious diseases globally and in low- and middle-income regions over 30 years, 1990–2019 16](#_Toc168780416)

[Supplementary Figure S2. Mortality rates of neonatal infectious diseases globally and in low- and middle-income regions over 30 years, 1990–2019 17](#_Toc168780417)

[Supplementary Figure S3. Male incidence rates of neonatal infectious diseases globally and in low- and middle-income regions over 30 years, 1990–2019 18](#_Toc168780418)

[Supplementary Figure S4. Female incidence rates neonatal infectious diseases globally and in low- and middle-income regions over 30 years, 1990–2019 19](#_Toc168780419)

[Supplementary Figure S5. Male mortality rates of neonatal infectious diseases globally and in low- and middle-income regions over 30 years, 1990–2019 20](#_Toc168780420)

[Supplementary Figure S6. Female mortality rates of neonatal infectious diseases globally and in low- and middle-income regions over 30 years, 1990–2019 21](#_Toc168780421)

[Supplementary Tables 22](#_Toc168780422)

[Supplementary Table S1. 131 LMICs as per Word Bank Classification (2019) ^†^ 22](#_Toc168780423)

[Supplementary Table S2. The SDI and UHCI of 131 low- and middle-income countries and territories in 2019. 24](#_Toc168780424)

[Supplementary Table S3. Incidence rates (per 100,000 liveborn neonates) of neonatal infectious diseases in LMICs with estimated annual percentage change (EAPC) from 1990 to 2019. 28](#_Toc168780425)

[Supplementary Table S4. Incident cases of neonatal infectious diseases in LMICs and their relative percent change from 1990 to 2019. 31](#_Toc168780426)

[Supplementary Table S5. Mortality rates (per 100,000 liveborn neonates) of neonatal infectious diseases in LMICs with estimated annual percentage change (EAPC) from 1990 to 2019. 34](#_Toc168780427)

[Supplementary Table S6. Deaths of neonatal infectious diseases in LMICs and their relative percent change from 1990 to 2019. 36](#_Toc168780428)

[Supplementary Table S7. National burden and trends of incident cases and incidence rates (per 100,000 liveborn neonates) of acute hepatitis in 131 LMICs between 1990 and 2019. 38](#_Toc168780429)

[Supplementary Table S8. National burden and trends of incident cases and incidence rates (per 100,000 liveborn neonates) of bacterial skin diseases in 131 LMICs between 1990 and 2019. 55](#_Toc168780430)

[Supplementary Table S9. National burden and trends of incident cases and incidence rates (per 100,000 liveborn neonates) of encephalitis in 131 LMICs between 1990 and 2019. 72](#_Toc168780431)

[Supplementary Table S10. National burden and trends of incident cases and incidence rates (per 100,000 liveborn neonates) of enteric infections in 131 LMICs between 1990 and 2019. 89](#_Toc168780432)

[Supplementary Table S11. National burden and trends of incident cases and incidence rates (per 100,000 liveborn neonates) of fungal skin diseases in 131 LMICs between 1990 and 2019. 106](#_Toc168780433)

[Supplementary Table S12. National burden and trends of incident cases and incidence rates (per 100,000 liveborn neonates) of lower respiratory infections in 131 LMICs between 1990 and 2019. 126](#_Toc168780434)

[Supplementary Table S13. National burden and trends of incident cases and incidence rates (per 100,000 liveborn neonates) of meningitis in 131 LMICs between 1990 and 2019. 144](#_Toc168780435)

[Supplementary Table S14. National burden and trends of incident cases and incidence rates (per 100,000 liveborn neonates) of neglected tropical diseases and malaria in 131 LMICs between 1990 and 2019. 161](#_Toc168780436)

[Supplementary Table S15. National burden and trends of incident cases and incidence rates (per 100,000 liveborn neonates) of neonatal sepsis and other neonatal infections in 131 LMICs between 1990 and 2019. 179](#_Toc168780437)

[Supplementary Table S16. National burden and trends of incident cases and incidence rates (per 100,000 liveborn neonates) of otitis media in 131 LMICs between 1990 and 2019. 196](#_Toc168780438)

[Supplementary Table S17. National burden and trends of incident cases and incidence rates (per 100,000 liveborn neonates) of scabies in 131 LMICs between 1990 and 2019. 213](#_Toc168780439)

[Supplementary Table S18. National burden and trends of incident cases and incidence rates (per 100,000 liveborn neonates) of tetanus in 131 LMICs between 1990 and 2019. 230](#_Toc168780440)

[Supplementary Table S19. National burden and trends of incident cases and incidence rates (per 100,000 liveborn neonates) of tuberculosis in 131 LMICs between 1990 and 2019. 248](#_Toc168780441)

[Supplementary Table S20. National burden and trends of incident cases and incidence rates (per 100,000 liveborn neonates) of upper respiratory infections in 131 LMICs between 1990 and 2019. 266](#_Toc168780442)

[Supplementary Table S21. National burden and trends of incident cases and incidence rates (per 100,000 liveborn neonates) of varicella and herpes zoster in 131 LMICs between 1990 and 2019. 283](#_Toc168780443)

[Supplementary Table S22. National burden and trends of deaths and mortality rates (per 100,000 liveborn neonates) of encephalitis in 131 LMICs between 1990 and 2019. 300](#_Toc168780444)

[Supplementary Table S23. National burden and trends of deaths and mortality rates (per 100,000 liveborn neonates) of enteric infections in 131 LMICs between 1990 and 2019. 317](#_Toc168780445)

[Supplementary Table S24. National burden and trends of deaths and mortality rates (per 100,000 liveborn neonates) of lower respiratory infections in 131 LMICs between 1990 and 2019. 334](#_Toc168780446)

[Supplementary Table S25. National burden and trends of deaths and mortality rates (per 100,000 liveborn neonates) of meningitis in 131 LMICs between 1990 and 2019. 351](#_Toc168780447)

[Supplementary Table S26. National burden and trends of deaths and mortality rates (per 100,000 liveborn neonates) of neglected tropical diseases and malaria in 131 LMICs between 1990 and 2019. 368](#_Toc168780448)

[Supplementary Table S27. National burden and trends of deaths and mortality rates (per 100,000 liveborn neonates) of neonatal sepsis and other neonatal infections in 131 LMICs between 1990 and 2019. 386](#_Toc168780449)

[Supplementary Table S28. National burden and trends of deaths and mortality rates (per 100,000 liveborn neonates) of otitis media in 131 LMICs between 1990 and 2019. 403](#_Toc168780450)

[Supplementary Table S29. National burden and trends of deaths and mortality rates (per 100,000 liveborn neonates) of tetanus in 131 LMICs between 1990 and 2019. 421](#_Toc168780451)

[Supplementary Table S30. National burden and trends of deaths and mortality rates (per 100,000 liveborn neonates) of upper respiratory infections in 131 LMICs between 1990 and 2019. 439](#_Toc168780452)

[Supplementary Table S31. National burden and trends of deaths and mortality rates (per 100,000 liveborn neonates) of varicella and herpes zoster in 131 LMICs between 1990 and 2019. 457](#_Toc168780453)

[Supplementary Table S32. Estimated annual percentage change (EAPCs) of incidence rates (per 100,000 liveborn neonates) of neonatal infectious diseases for both sexes in LMICs from 1990 to 2019. 475](#_Toc168780454)

[Supplementary Table S33. Estimated annual percentage change (EAPCs) of mortality rates (per 100,000 liveborn neonates) of neonatal infectious diseases for both sexes in LMICs from 1990 to 2019. 477](#_Toc168780455)

# Supplementary Materials

# Supplementary Methods

In this section, we have excerpted and consolidated content from the main text and supplementary materials of the article titled “Global burden of 369 diseases and injuries in 204 countries and territories, 1990–2019: a systematic analysis for the Global Burden of Disease Study 2019” by GBD 2019 Diseases and Injuries Collaborators (Lancet 2020; 396: 1204–22). It is essential to make this statement in acknowledgment. We would like to express our sincere gratitude to the 2019 Global Burden of Disease Study collaborators for providing the data used in this study.

## 1. GBD Overview

The Global Burden of Diseases, Injuries, and Risk Factors Study (GBD) provides a systematic scientific assessment of published, publicly available, and contributed data on global descriptive epidemiology. It estimates incidence, prevalence, mortality, years of life lost (YLLs), years lived with disability (YLDs), and disability-adjusted life-years (DALYs) due to 369 diseases and injuries, for two sexes, and for 204 countries and territories in the past decades. The numbers and rates of incidence, prevalence, years lived with disability (YLDs), and disability-adjusted life-years (DALYs) were estimated for the years 1990–2019, while deaths and years of life lost (YLLs) were estimated for 1980–2019. The cause and sequelae list were based on input from the Scientific Council and GBD collaborator network.

GBD Input data were extracted from censuses, household surveys, civil registration and vital statistics, disease registries, health service use, air pollution monitors, satellite imaging, disease notifications, and other sources. Cause-specific death rates and cause fractions were calculated using the Cause of Death Ensemble model and spatiotemporal Gaussian process regression. Cause-specific deaths were adjusted to match the total all-cause deaths calculated as part of the GBD population, fertility, and mortality estimates. Deaths were multiplied by standard life expectancy at each age to calculate YLLs. A Bayesian meta-regression modelling tool, DisMod-MR 2.1, was used to ensure consistency between incidence, prevalence, remission, excess mortality, and cause-specific mortality for most causes. Prevalence estimates were multiplied by disability weights for mutually exclusive sequelae of diseases and injuries to calculate YLDs. Uncertainty intervals (UIs) were generated for every metric using the 25th and 975th ordered 1000 draw values of the posterior distribution.

## 2. Disease Definition

| Causes | Definition |
| --- | --- |
| Acute hepatitis | Acute Hepatitis: A, B, C, and E.  (1) Acute Hepatitis A: We define acute hepatitis A as an infection with the hepatitis A virus resulting in anti-HAV IgG seroconversion, regardless of symptoms.  (2) Acute Hepatitis B: We define acute hepatitis B as the period corresponding to initial infection with the hepatitis B virus, regardless of symptoms.  (3) Acute Hepatitis C: We define acute hepatitis C as the period corresponding to initial infection with the hepatitis C virus, resulting in anti-HCV IgG seroconversion, regardless of symptoms.  (4) Acute Hepatitis E: We define acute hepatitis E as an infection with the hepatitis E virus resulting in anti-HEV IgG seroconversion, regardless of symptoms. |
| Bacterial skin diseases | (1) Cellulitis: Cellulitis was included in the GBD 2019 cause group of skin and subcutaneous conditions. Cellulitis is a skin disease marked by a bacterial infection that affects and spreads through the skin and soft tissues. Symptoms of cellulitis include pain, tenderness, and reddening in the affected area, fever, chills, and lymphadenopathy (ICD-10: L03) .  (2) Pyoderma: Pyoderma refers to any skin disease that is pyogenic, ie, involves the development of pus. These include superficial bacterial conditions such as impetigo, furuncles, ulcers, and abscesses. In line with GBD 2017, for GBD 2019, pyoderma was modelled as two separate groups: impetigo, and abscess and other bacterial skin diseases. Impetigo is a highly contagious bacterial skin infection often characterized by red sores, which eventually leak pus or fluid (ICD-10: L01). An abscess is a collection of pus that builds up within the tissue of the body, with carbuncles and furuncles being examples of specific types of abscess. The abscess and other bacterial skin diseases group included all bacterial skin diseases except impetigo (ICD-10: L00, L02, L04, L05, L08). |
| Encephalitis | Encephalitis is a disease caused by an acute inflammation of the brain. Symptoms of encephalitis can include flu-like symptoms like headache, fever, drowsiness, and fatigue, and at times, seizures, hallucinations, or stroke. Included in the GBD modelling were cases meeting ICD-10 diagnostic criteria for encephalitis (A83-A86.4, B94.1, F07.1, G04-G05.8) |
| Enteric infections | Enteric infections consist of four parts：Diarrheal disease, Typhoid and paratyphoid fevers, Invasive non-typhoidal salmonella (iNTS).  (1) Diarrheal disease: We defined diarrheal disease episodes as three or more loose stools in a 24-hour period. In the diarrhea models, self-reported prevalence is the reference category for all data adjustments. Hospital input data use ICD9 codes 001-009.9 and ICD10 codes A00-A09. We excluded gastroenteritis as a case definition as this is often syndromic (vomiting or diarrhea).  (2) Typhoid and paratyphoid fevers: Typhoid and paratyphoid are acute bacterial infections that most commonly cause febrile illness and gastrointestinal symptoms. Severe cases are associated with intestinal bleeding and perforation, altered mental state and, in some cases, death. We define a confirmed case as one for which there has been a positive blood culture test for either Salmonella enterica typhi or para-typhi. Diagnostic criteria do not typically accompany national surveillance reports; however, with blood culture being the standard diagnostic, we treat reported cases as confirmed. Given the poor sensitivity of blood culture, however, we estimated case definition as simply febrile illness resulting from an infection with Salmonella enterica typhi or para-typhi. This is effectively a counterfactual definition in which we attempt to estimate the number of true infections regardless of test result. These causes include all ICD-10 codes under the heading A01 (Typhoid and paratyphoid fevers).  (3) Invasive non-typhoidal salmonella (iNTS): Non-typhoidal salmonella infections are typically associated with diarrhoea. When these bacteria invade a typically sterile site like blood, they produce invasive non-typhoidal salmonella (iNTS) disease. Whereas non-typhoidal salmonella infections typically produce diarrhoeal illness, iNTS is typically febrile and can manifest in diverse symptoms that vary with severity and the exact site of the infection. Blood culture is the standard diagnostic for iNTS, and has good sensitivity and specificity. We thus define a case of iNTS as any blood-culture-confirmed non-typhoidal salmonella infection.  (4) In addition to the intestinal infectious diseases described above, there are many diverse types of intestinal infectious diseases. Because these intestinal infectious diseases are diverse in their underlying causes and risk factors as well as in their associated health outcomes, modelling them together in a DisMod-MR model would not produce reliable estimates of prevalence or excess mortality. Instead, we calculated the YLDs caused by intestinal infectious diseases directly using a YLD/YLL ratio. |
| Fungal skin diseases | Fungal diseases were included in the GBD 2019 cause group of skin and subcutaneous conditions and consisted of tinea capitis and a residual group of “any” other fungal disease. Similar to GBD 2017, tinea capitis was modelled separately from the other fungal skin diseases. This was done to better accommodate differences in burden between tinea capitis and other subtypes of fungal skin diseases. |
| Lower respiratory infections | We used clinician-diagnosed pneumonia or bronchiolitis as our case definition for lower respiratory infections (LRI). We included ICD9 codes 073.0-073.6, 079.82, 466-469, 480-489, 513.0, and 770.0 and ICD10 codes A48.1, J09-J22, J85.1, P23-P23.9, and U04. LRI etiologies are modeled separately from overall LRI incidence and prevalence. The etiologies include influenza, respiratory syncytial virus, Streptococcus pneumoniae, and Haemophilus influenzae type b and are episodes of LRI where the etiology is the causal pathogen in the infection. |
| Meningitis | Meningitis is a disease caused by inflammation of the meninges, the protective membrane surrounding the brain and spinal cord, and is typically caused by an infection in the cerebrospinal fluid. Symptoms include headache, fever, stiff neck, and sometimes seizures. Included in the GBD modelling were cases meeting ICD-10 diagnostic criteria for meningitis due to bacteria or viruses (A39-A39.9, A87-A87.9, and G00.0-G00.8). In GBD 2019, meningitis encompasses viral meningitis and four bacterial aetiologies: pneumococcal, Haemophilus influenzae type B (HiB), meningococcal, and other bacterial meningitis. |
| Neglected tropical diseases and malaria | (1) Malaria: Malaria is an acute parasitic mosquito-borne disease. An individual with uncomplicated malaria experiences one to two weeks of persistent fever, chills/shivering, sweating, joint pains, and headache. The individual will likely be lethargic and feverish, causing loss of daily function during the attack. Individuals with an untreated P. falciparum infection may develop severe malaria, which includes the symptoms of uncomplicated malaria but may also involve swelling, difficulty breathing, unconsciousness, and potentially death. Microscopy is considered the gold-standard diagnostic approach for the purposes of GBD. The relevant ICD-10 codes are B50-B54.  (2) Chagas disease: Chagas disease is defined by infection with the protozoa Trypanosoma cruzi, which is transmitted by Triatominae insect vectors (most common), blood transfusion, organ transplant, and congenital transmission. It includes an acute phase corresponding with the time of infection, and is typically asymptomatic. Chronic infection may be latent (ie, asymptomatic), or result in cardiovascular or digestive sequelae. It includes all ICD-10 codes under the heading B57 (Chagas disease), with codes B57.0-B75.1 corresponding to the acute phase, B57.2 corresponding to chronic cardiovascular sequelae, and B57.3 corresponding to chronic digestive sequelae.  (3) Visceral leishmaniasis: Visceral leishmaniasis (VL) is the most serious manifestation of disease caused by the Leishmania parasite, transmitted through the bite of phlebotomine sandflies. Those infected typically present with fever, weight loss, anaemia, leukopenia, thrombocytopenia, and enlargement of the spleen and liver. If left untreated, it can be fatal. Transmission varies by geographic region, with a variety of reservoir hosts implicated, and different vector species associated, maintaining both zoonotic and anthroponotic transmission cycles. The ICD9 code related to visceral leishmaniasis is 085.0, and the ICD10 code is B55.0.  (4) Cutaneous leishmaniasis: Cutaneous leishmaniasis is the most common manifestation of infection by the protozoan Leishmania, transmitted by infected female phlebotomine sandflies. It is a tropical disease associated with poverty and poor living conditions. The ICD9 code related to visceral leishmaniasis is 085.0, and the ICD10 code is B55.0, B55.1, B55.2. (5) Human African Trypanosomiasis (HAT): Human African trypanosomiasis (HAT), also known as sleeping sickness, is a vector-borne disease which is transmitted by the bite of the tsetse fly. It is caused by the parasite Trypanosoma brucei with two subspecies, namely T.b. rhodesiense (makes up less than 5% of total HAT cases) and T.b. gambiense. Cases are diagnosed through laboratory methods which rest on finding the parasite in body fluid or tissue by microscopy. In highly endemic or epidemic areas where the likelihood of false positives in serological tests is deemed lower, a seropositive individual is considered affected even in the absence of parasitological confirmation. The ICD-10 codes for HAT are B56.0, B56.1 and B56.9.  (6) Schistosomiasis: Schistosomiasis, also known as bilharzia or “snail fever,” is a helminth disease caused by infection with five species of the parasite Schistosoma, namely, S mansoni, S japonicum, S haematobium, S mekongi, and S intercalatum. It is considered a neglected tropical disease (NTD). The first three species cause the most infection and the last two rarely cause disease. Diagnosis is made by microscopic exam of stool or urine for parasite eggs. For less advanced infections, serologic techniques are used. The ICD-10 codes for schistosomiasis are B65-B65.9.  (7) Cysticercosis: Cysticercosis, or neurocysticercosis (NCC), is a parasitic disease caused by the pig tapeworm Taenia solium. It is transmitted via ingestion of eggs or gravid proglottids shed by a human or non-human host with an intestinal infection of the same helminth known as Taeniasis. In rare cases, auto-infection is also possible among people with intestinal infections. Diagnosis is made by magnetic resonance imaging (MRI) or computerized tomography (CT) brain scans to identify cysts. The ICD-10 codes for cysticercosis are B69-B69.9.  (8) Cystic Echinococcosis: Cystic echinococcosis is a parasitic disease caused by infection with the Echinococcus granulosis tapeworm. It is a natural parasite of canines, with sheep being the most common intermediate host in the two-stage lifecycle, but can be spread to humans through ingestion of soil, water, or food contaminated with the fecal matter of an infected dog containing infective eggs. Diagnosis is made by clinical findings, imaging, serology, and tissue pathology. The ICD-9 and ICD-10 codes for echinococcosis are 122.0-122.9 and B67-B67.9, respectively.  (9) Lymphatic Filariasis: Lymphatic filariasis (LF) is a neglected tropical disease in which threadlike nematodes invade the lymphatic system. The worms responsible – Wuchereria bancrofti, Brugia malayi, and Brugia timori – are spread from human to human via mosquitoes. The most prominent clinical manifestations of LF are lymphoedema (a swelling of the legs, also known in its more extreme manifestation as elephantiasis) and hydrocele (a collection of fluid in the sac around the testicles).  (10) Onchocerciasis: Onchocerciasis, also known as river blindness, is a parasitic disease caused by Onchocerca volvulus. It is transmitted via the bite of one of several species of Simulium blackflies that have historically bred in fast-moving freshwater rivers and tributaries throughout sub-Saharan Africa, Central America, and South America. Diagnosis can be made by skin snip biopsy to identify larvae, surgical removal of nodules and exam for adult worms, slit lamp exam of anterior part of the eye where larvae or lesions caused by them are visible, and antibody tests (mostly useful to visitors to areas with parasites). The ICD-10 code for onchocerciasis is B73.  (11) Dengue: Dengue is mosquito-borne viral infection that causes febrile illness and, in severe cases, jaundice, haemorrhage, and death. It includes all ICD-10 codes under the heading A90 (Dengue fever [classical dengue]) and A91 (Dengue haemorrhagic fever).  (12) Yellow Fever: Yellow fever is mosquito-borne viral infection that causes febrile illness and, in severe cases, jaundice, haemorrhage, and death. It is considered a neglected tropical disease (NTD). It includes all ICD-10 codes under the heading A95 (yellow fever).  (13) Rabies: Rabies is a fatal viral infection transmitted by animal bites. Without prophylactic vaccination the disease is almost universally fatal. The disease has a long incubation period (1-3 months), and early intervention with prophylactic vaccination is nearly 100% effective in preventing symptomatic disease. It is considered a neglected tropical disease (NTD). We model symptomatic infections, not including those infections in which intervention prevented the onset of symptomatic disease, corresponding to the ICD10 code A82.  (14) Ascariasis: Ascariasis is a helminthic disease caused by the parasitic roundworm Ascaris lumbricoides. It is one of the three intestinal nematode infections (INI), or soil-transmitted helminthiasis (STH), that are modelled in GBD. Diagnosis is made by examination of stool by microscope or PCR, with or without concentration procedures. The ICD-10 codes for ascariasis are B77-B77.9.  (15) Trichuriasis: Trichuriasis is a helminth diseases caused by the parasitic whipworm Trichuris trichiura. It is one of the three intestinal nematode infections (INI), or soil-transmitted helminthiasis (STH), that we model in GBD. Diagnosis is made by examination of stool by microscope or PCR, with or without concentration procedures. The ICD-10 code for trichuriasis is B79.  (16) Hookworm Disease: Hookworm disease is a helminthic disease caused by intestinal parasites in the roundworm group, Ancylostoma duodenale and Necator americanus. It is one of the three intestinal nematode infections (INI), or soil-transmitted helminthiasis (STH), that we model in GBD. Diagnosis is made by examination of stool by microscope or PCR, with or without concentration procedures. The ICD-10 codes for hookworm disease are B76-B76.9.  (17) Foodborne Trematodiases: Human foodborne trematodiases (FBT) is defined as the infection with parasitic worms of the class trematoda, which are also known as flukes. Trematodes are transmitted via contaminated food, and infection is highly related to food habits. Definitive hosts, including humans, become infected when ingesting viable metacercariae by consuming contaminated aquatic products (eg, watercress). In the ICD-10, FBT are listed under code B66. FBT is subdivided into six types of FBT (see Table 1):  • Clonorchiasis  • Fascioliasis  • Intestinal fluke  • Opisthorchiasis  • Paragonimiasis (normal and cerebral infections).  (18) Leprosy: Leprosy is a chronic bacterial infection caused by Mycobacterium leprae, primarily affecting the nervous system, skin, respiratory tract, and eyes. Transmission is facilitated through contact with fluid from the nose and mouth of an infected individual. The ICD-10 code for Leprosy is A30.9.  (19) Ebola virus disease: Ebola virus is a relatively rare viral pathogen linked with high case fatality rates in both humans and non-human primates. The disease is zoonotic, and while bats have been implicated as reservoirs, definitive host species are yet to be identified. Once a human becomes infected after viral transmission from animal sources either directly or indirectly, secondary human-to-human transmission is possible, primarily through exchange of infectious bodily fluids and secretions. Clinical cases typically present initially as a febrile illness, similar to a number of different pathogens, which can be subsequently followed by haemorrhagic complications and death. Historically there have been a number of outbreaks, usually no more than a few hundred cases, typically constrained to one country, focused in Central Africa. The West African outbreak, however, which started in Guinea in 2013, claimed more lives than all previous outbreaks combined, and spread across the region seeding additional outbreaks. There is an ICD code for 659 Ebola, A98.4, but no data used in the modelling reference that coding (ie, all the data are from literature extractions). Data for Ebola virus disease were only included if the case was identified as either “probable” or “confirmed” as per WHO definitions [http://www.who.int/csr/resources/publications/ebola/ebola-case-definition-contact-en.pdf]. A confirmed case is any suspected or probable case with a positive laboratory result through either detection of virus RNA via reverse transcriptase-polymerase chain reaction, or by detection of IgM antibodies directed against Ebola. A probable case is any suspected case evaluated by a clinician or any deceased suspected case with an epidemiological link to a confirmed case.  (20) Zika: Zika virus disease is an infectious disease caused by the Zika virus, which is primarily transmitted to humans through the bite of an infected Aedes mosquito, and it can also be transmitted through sexual contact and from mother to fetus during pregnancy. The infection is usually mild and self-limiting, but it has been associated with severe birth defects, such as microcephaly, when contracted during pregnancy.  (21) Dracunculiasis (Guinea worm): A Guinea worm case is defined as an individual with Guinea worm disease. A person is counted as a case only once in a calendar year, ie, when the first Guinea worm emerged from that person, although an individual may have more than one worm emerge at a time and/or more than one worm emerge during the year. These cases are confirmed through the Guinea worm eradication program infrastructure by clinical exam and verification by local supervisors. All specimens from case-patients are sent to the CDC for laboratory evaluation and confirmation.  (22) Other neglected tropical diseases: In addition to the neglected tropical diseases described above, there are many diverse types of neglected tropical diseases, which are encompassed by the following ICD 10 codes: A68 Relapsing fevers  A68.0 Louse-borne relapsing fever  A68.1 Tick-borne relapsing fever  A68.9 Relapsing fever, unspecified  A69.2 Lyme disease  A69.20 Lyme disease, unspecified  A69.21 Meningitis due to Lyme disease  A69.22 Other neurologic disorders in Lyme disease  A69.23 Arthritis due to Lyme disease  A69.29 Other conditions associated with Lyme disease  A69.5 There is not this code in ICD10 site, but we have this in mortality data  A69.8 Other specified spirochetal infections  A69.9 Spirochetal infection, unspecified  A75 Typhus fever  A75.0 Epidemic louse-borne typhus fever due to Rickettsia prowazekii  A75.1 Recrudescent typhus [Brill's disease]  A75.2 Typhus fever due to Rickettsia typhi  A75.3 Typhus fever due to Rickettsia tsutsugamushi  A75.9 Typhus fever, unspecified  A77 Spotted fever [tick-borne rickettsioses]  A77.0 Spotted fever due to Rickettsia rickettsii  A77.1 Spotted fever due to Rickettsia conorii  A77.2 Spotted fever due to Rickettsia siberica  A77.3 Spotted fever due to Rickettsia australis  A77.4 Ehrlichiosis  A77.40 Ehrlichiosis, unspecified  A77.41 Ehrlichiosis chafeensis [E. chafeensis]  A77.49 Other ehrlichiosis  A77.8 Other spotted fevers  A77.9 Spotted fever, unspecified  A78 Q fever  A79 Other rickettsioses  A79.0 Trench fever  A79.1 Rickettsialpox due to Rickettsia akari  A79.8 Other specified rickettsioses  A79.81 Rickettsiosis due to Ehrlichia sennetsu  A79.89 Other specified rickettsioses  A79.9 Rickettsiosis, unspecified  A92 Other mosquito-borne viral fevers  A92.0 Chikungunya virus disease  A92.1 O'nyong-nyong fever  A92.2 Venezuelan equine fever  A92.3 West Nile virus infection  A92.30 West Nile virus infection, unspecified  A92.31 West Nile virus infection with encephalitis  A92.32 West Nile virus infection with other neurologic manifestation  A92.39 West Nile virus infection with other complications  A92.4 Rift Valley fever  A92.8 Other specified mosquito-borne viral fevers  A92.9 Mosquito-borne viral fever, unspecified  A93 Other arthropod-borne viral fevers, not elsewhere classified  A93.0 Oropouche virus disease  A93.1 Sandfly fever  A93.2 Colorado tick fever  A93.8 Other specified Arthropod-borne viral fevers  A94 Unspecified arthropod-borne viral fever  A94.0 Unspecified arthropod-borne viral fever  A96 Arenaviral hemorrhagic fever  A96.0 Junin hemorrhagic fever  A96.1 Machupo hemorrhagic fever  A96.2 Lassa fever  A96.8 Other arenaviral hemorrhagic fevers  A96.9 Arenaviral hemorrhagic fever, unspecified  A98 Other viral hemorrhagic fevers, not elsewhere classified  A98.0 Crimean-Congo hemorrhagic fever  A98.1 Omsk hemorrhagic fever  A98.2 Kyasanur Forest disease  A98.3 Marburg virus disease  A98.5 Hemorrhagic fever with renal syndrome  A98.8 Other specified viral hemorrhagic fevers  B33.0 Epidemic myalgia  B33.1 Ross River disease  B60 Other protozoal diseases, not elsewhere classified  B60.0 Babesiosis  B60.1  Acanthamebiasis  B60.10 Acanthamebiasis, unspecified  B60.11 Meningoencephalitis due to Acanthamoeba (culbertsoni)  B60.12 Conjunctivitis due to Acanthamoeba  B60.13 Keratoconjunctivitis due to Acanthamoeba  B60.19 Other acanthamebic disease  B60.2 Naegleriasis  B60.8 Other specified protozoal diseases  B67.5 Echinococcus multilocularis infection of liver  B67.6 Echinococcus multilocularis infection, other and multiple sites  B67.61 Echinococcus multilocularis infection, multiple sites  B67.69 Echinococcus multilocularis infection, other sites  B67.7 Echinococcus multilocularis infection, unspecified  B70 Diphyllobothriasis and sparganosis  B70.0 Diphyllobothriasis  B70.1 Sparganosis  B71 Other cestode infections  B71.0 Hymenolepiasis  B71.1 Dipylidiasis  B71.8 Other specified cestode infections  B71.9 Cestode infection, unspecified  B74.3 Loiasis  B74.4 Mansonelliasis  B74.8 Other filariases  B74.9 Filariasis, unspecified  B75 Trichinellosis  B83 Other helminthiases  B83.0 Visceral larva migrans  B83.1 Gnathostomiasis  B83.2 Angiostrongyliasis due to Parastrongylus cantonensis  B83.3 Syngamiasis  B83.4 Internal hirudiniasis  B83.8 Other specified helminthiases P37.1 Congenital toxoplasmosis. |
| Neonatal sepsis and other neonatal infections | Neonatal sepsis and other neonatal infections are infections during the neonatal period that advance to a systemic bloodstream infection (sepsis) and infections that occur during the neonatal period that are not already modeled separately in the GBD. |
| Otitis media | Otitis media is an infection of the middle ear space. We included acute otitis media, chronic otitis media, and hearing loss due to chronic otitis media in the GBD non-fatal outcome modelling. Hearing loss due to chronic otitis media estimation is included in the hearing loss report provided separately. The ICD 10 codes are H65-H75.83, and ICD 9 codes are 381-384.9. |
| Scabies | Scabies was included in the GBD 2019 cause group of skin and subcutaneous conditions. According to the International Classification of Diseases (ICD-10), scabies is a skin disease caused by the microscopic mite Sarcoptes scabiei. The main symptom is an itchy, pimple-like rash (ICD-10: B86). |
| Tetanus | Tetanus is a serious bacterial disease caused by the bacterium Clostridium tetani. For tetanus, the ICD 10 codes are A33-A35.0, Z23.5, and ICD 9 codes are 037-037.9,771.3,V03.7. |
| Tuberculosis | Tuberculosis (TB) is an infectious disease caused by Mycobacterium tuberculosis. The case definition includes all forms of TB, including pulmonary TB and extrapulmonary TB, which are bacteriologically confirmed or clinically diagnosed. For TB, the ICD 10 codes are A10-A19.9, B90-B90.9, K67.3, K93.0, M49.0, P37.0, and ICD 9 codes are 010-019.9, 137-137.9, 138.0, 138.9, 139.9, 320.4, 730.4-730.6. For HIV-TB, the ICD 10 code is B20.0. Latent TB infection is defined as an infection with Mycobacterium tuberculosis, without any symptoms or signs of active TB disease. We separately estimated the incidence and prevalence of multidrug-resistant tuberculosis and extensively drug-resistant tuberculosis by HIV status. The case definitions are shown below.  (1) Multidrug-resistant TB without extensive drug resistance: a form of TB (among HIV-negative individuals) that is resistant to the two most effective first-line anti-tuberculosis drugs (isoniazid and rifampicin), but is not resistant to any fluoroquinolone and any second-line injectable drugs (amikacin, kanamycin, or capreomycin).  (2) Extensively drug-resistant TB: a form of TB (among HIV-negative individuals) that is resistant to isoniazid and rifampicin, plus any fluoroquinolone and any second-line injectable drugs.  (3) Drug-susceptible TB: TB (among HIV-negative individuals) that is susceptible to isoniazid and rifampicin.  (4) HIV/AIDS - Multidrug-resistant TB without extensive drug resistance: a form of TB (among HIVpositive individuals) that is resistant to the two most effective first-line anti-tuberculosis drugs (isoniazid and rifampicin), but is not resistant to any fluoroquinolone and any second-line injectable drugs (amikacin, kanamycin, or capreomycin).  (5) HIV/AIDS - Extensively drug-resistant TB: a form of TB (among HIV-positive individuals) that is resistant to isoniazid and rifampicin, plus any fluoroquinolone and any second-line injectable drugs.  (6) HIV/AIDS - Drug-susceptible TB: TB (among HIV-positive individuals) that is susceptible to isoniazid and rifampicin. |
| Upper respiratory infections | Upper respiratory infections (URI) include cough, acute nasopharyngitis, sinusitis, pharyngitis, tonsillitis, laryngitis/tracheitis, epiglottitis, rhinitis, rhinosinusitis, rhinopharyngitis, supraglottitis, and the common cold. For URI, ICD 10 codes are J00-J02, J02.8-J03, J03.8-J06.9, J36, J36.0, `and ICD 9 codes are 460-465.9, 475-475.9, 476.9. |
| Varicella and herpes zoster | Varicella (also known as chickenpox) is an acute infectious disease caused by primary infection of the varicella-zoster virus. Herpes zoster (also known as shingles) is caused by the reactivation of the same virus that causes varicella in adults. For varicella and herpes zoster, the ICD 10 codes are B01-B02.9, P35.8, Z20.820, and ICD 9 codes are 052-053.9, V01.71, V01.79, V05.4. |
| ICD, International Classification of Diseases. | |

#
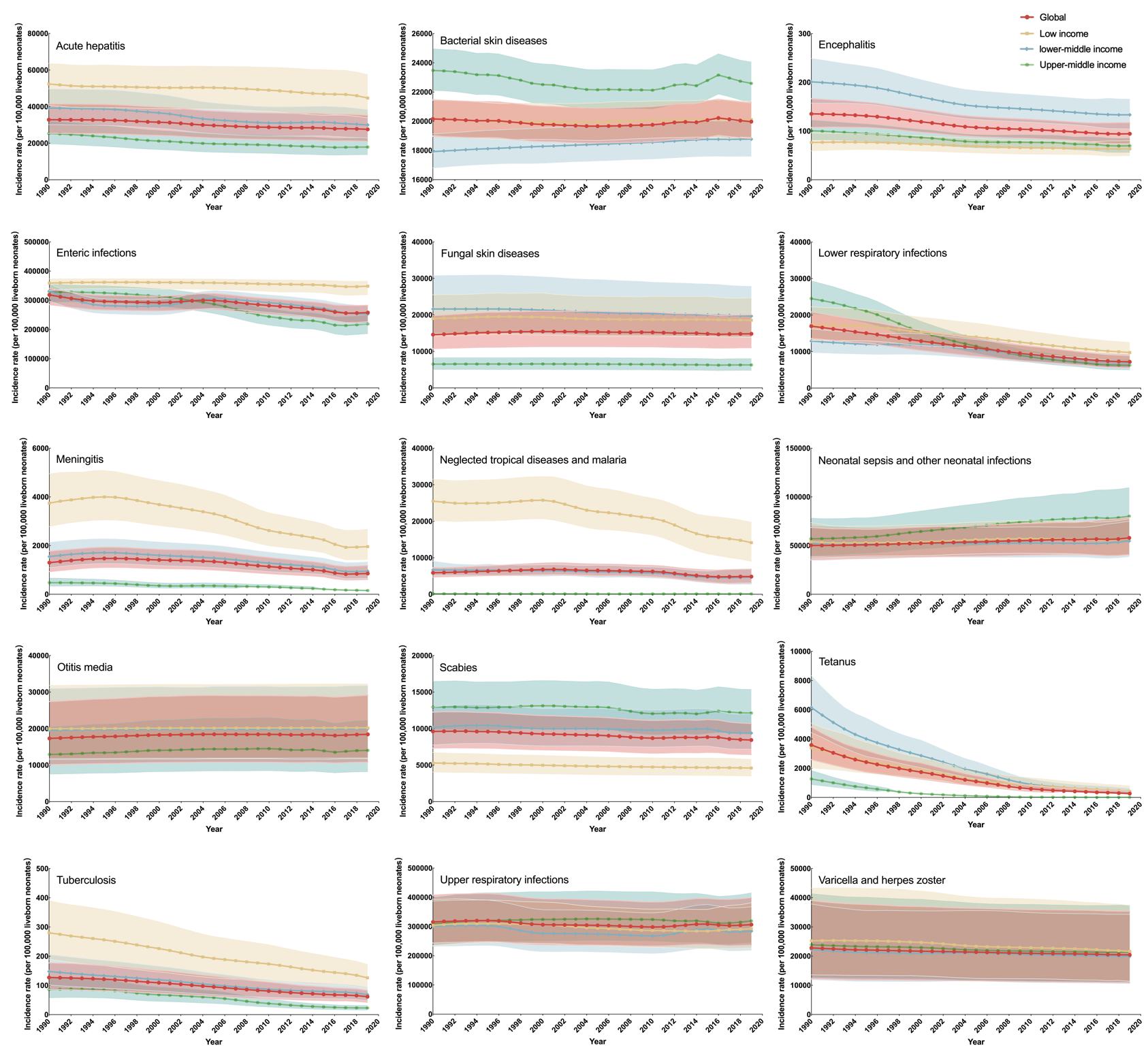
Supplementary Figures

## Supplementary Figure S1. Incidence rates of neonatal infectious diseases globally and in low- and middle-income regions over 30 years, 1990–2019

##
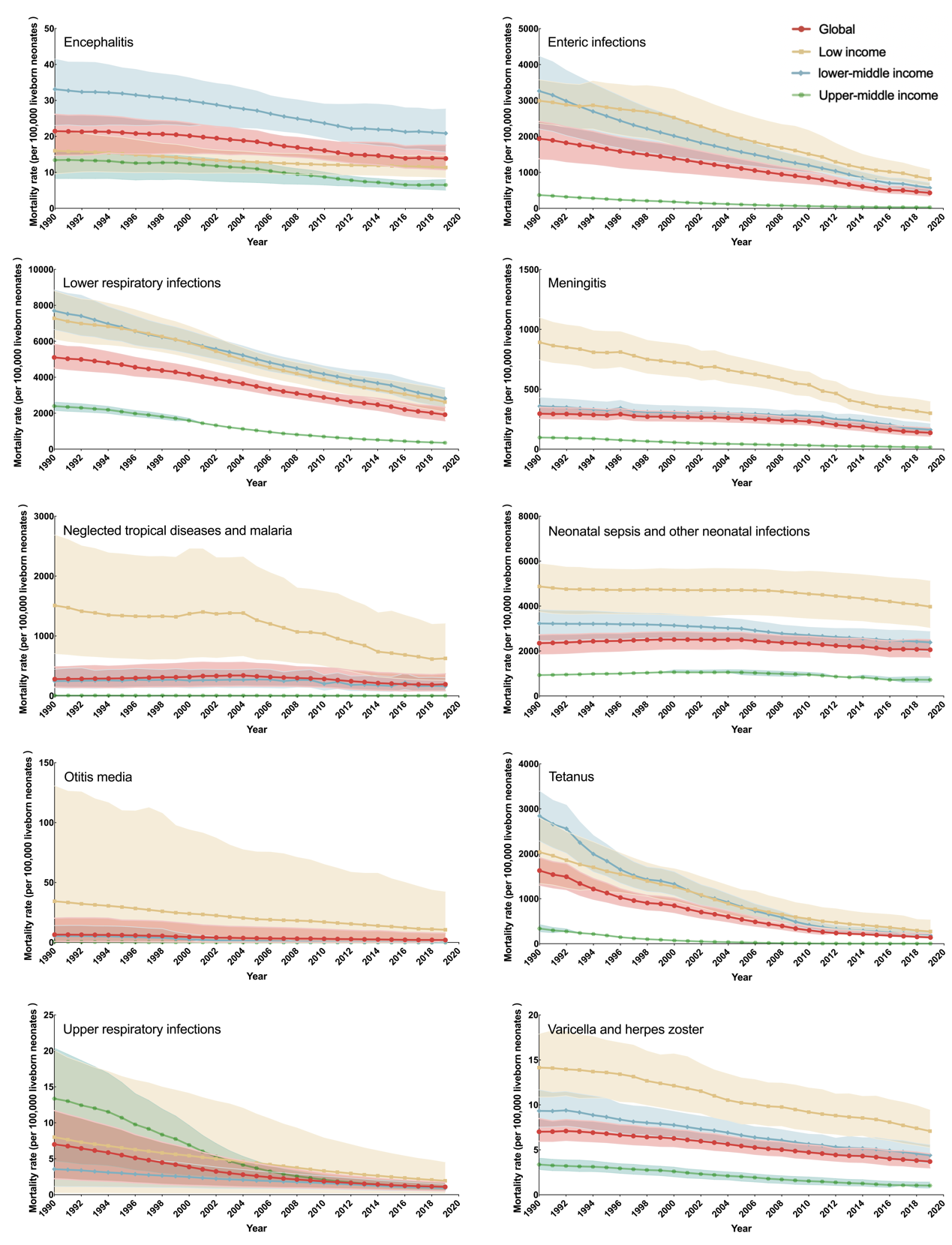
Supplementary Figure S2. Mortality rates of neonatal infectious diseases globally and in low- and middle-income regions over 30 years, 1990–2019

**
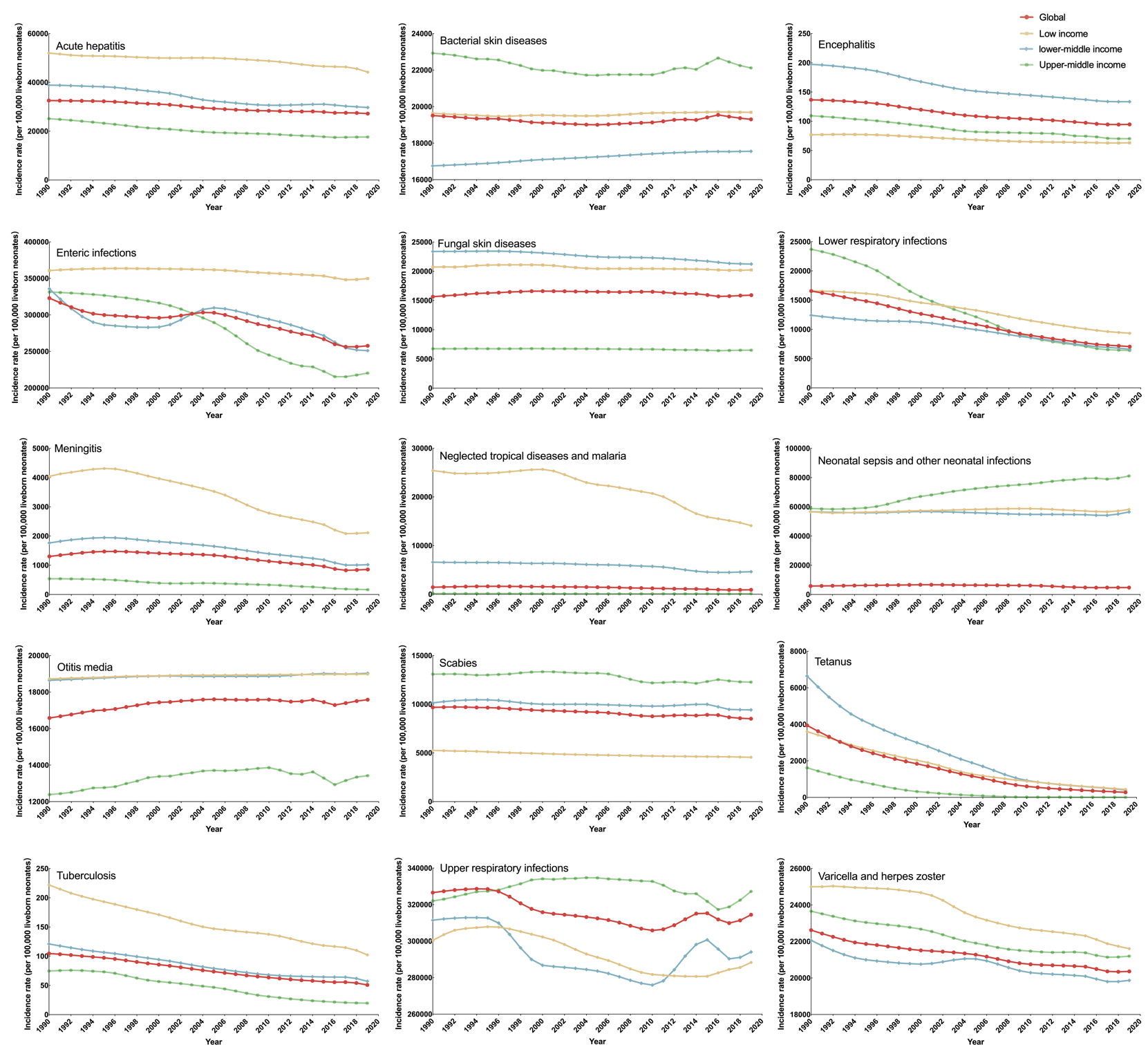
**

## Supplementary Figure S3. Male incidence rates of neonatal infectious diseases globally and in low- and middle-income regions over 30 years, 1990–2019

##
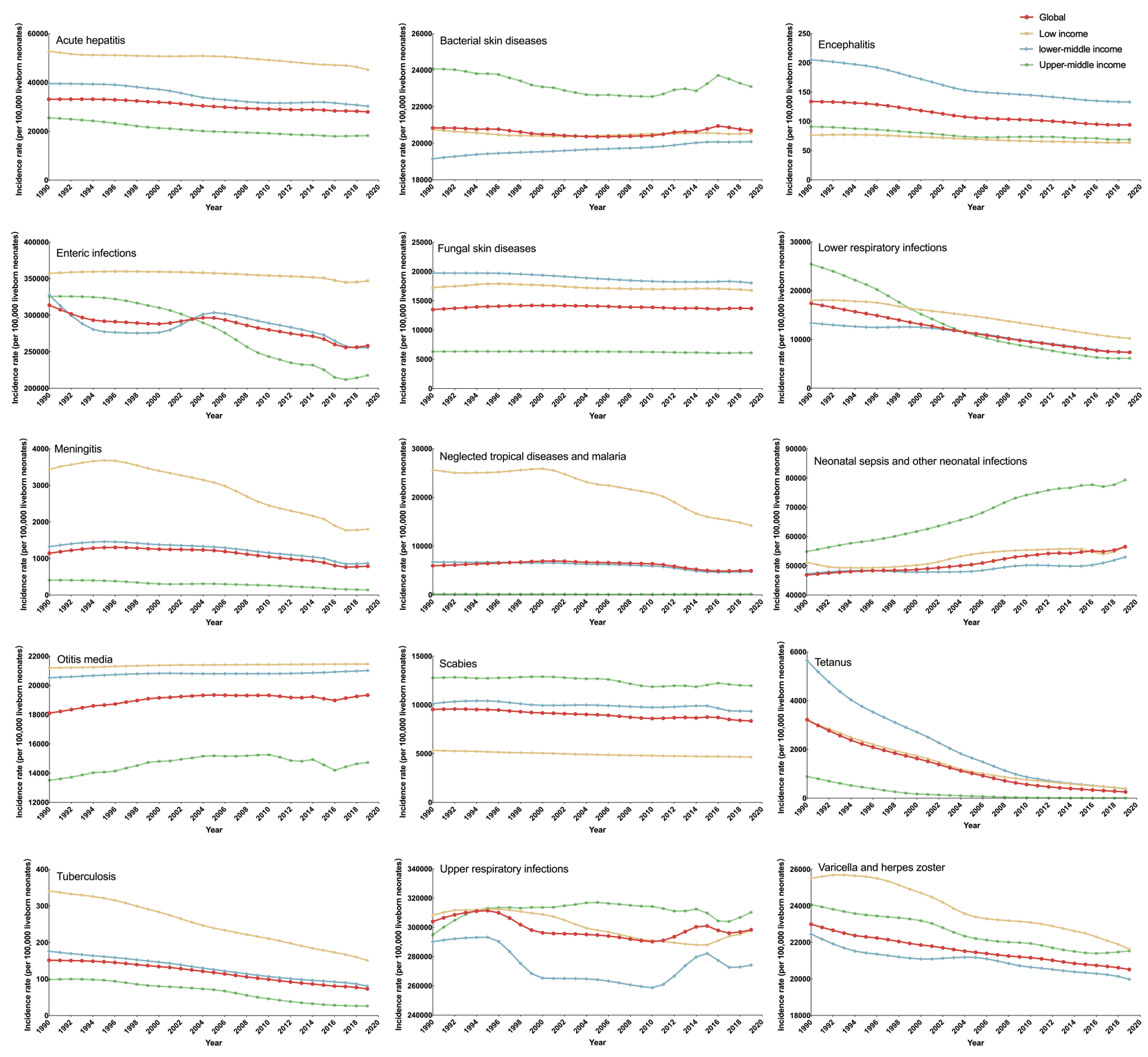
Supplementary Figure S4. Female incidence rates neonatal infectious diseases globally and in low- and middle-income regions over 30 years, 1990–2019

##
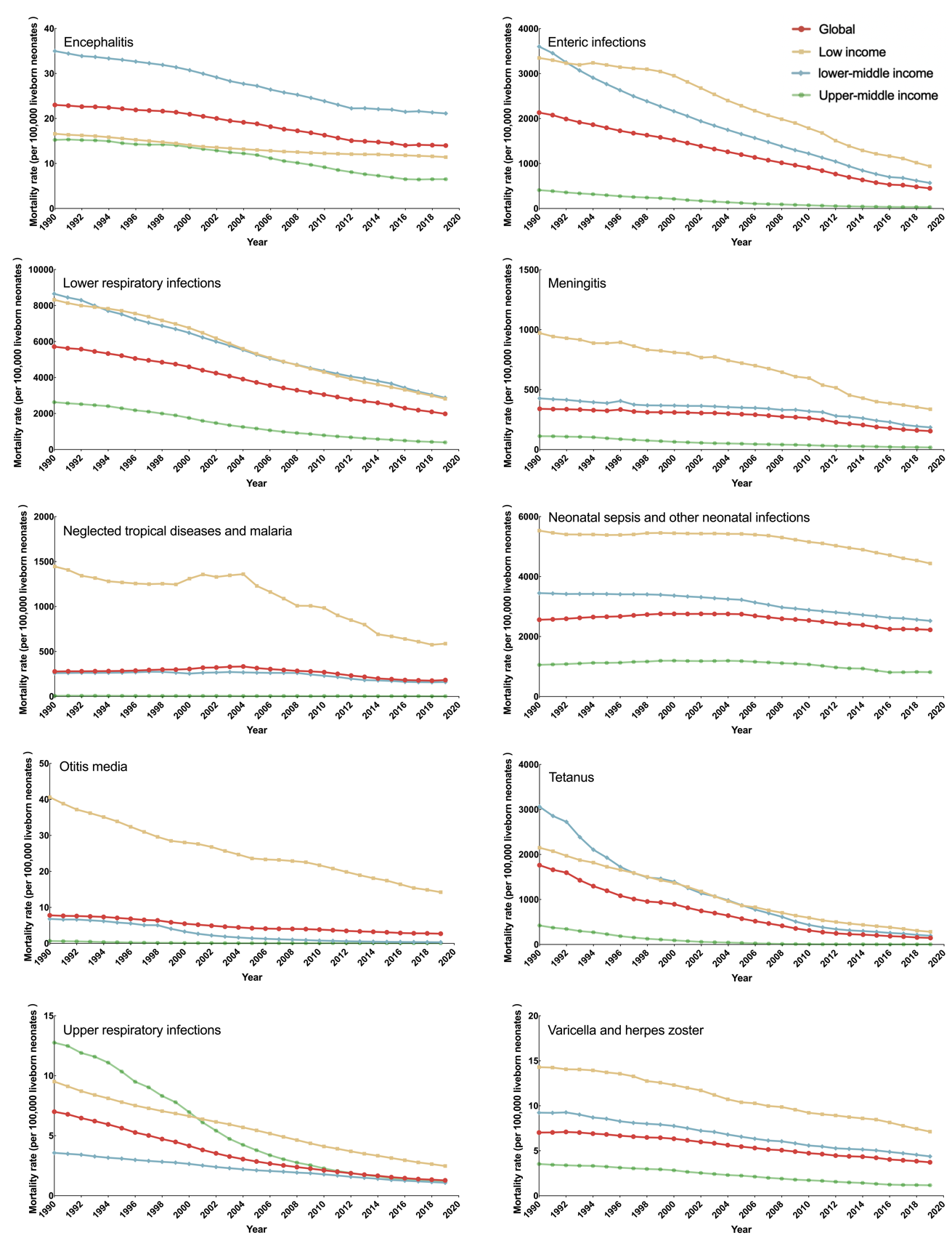
Supplementary Figure S5. Male mortality rates of neonatal infectious diseases globally and in low- and middle-income regions over 30 years, 1990–2019

**
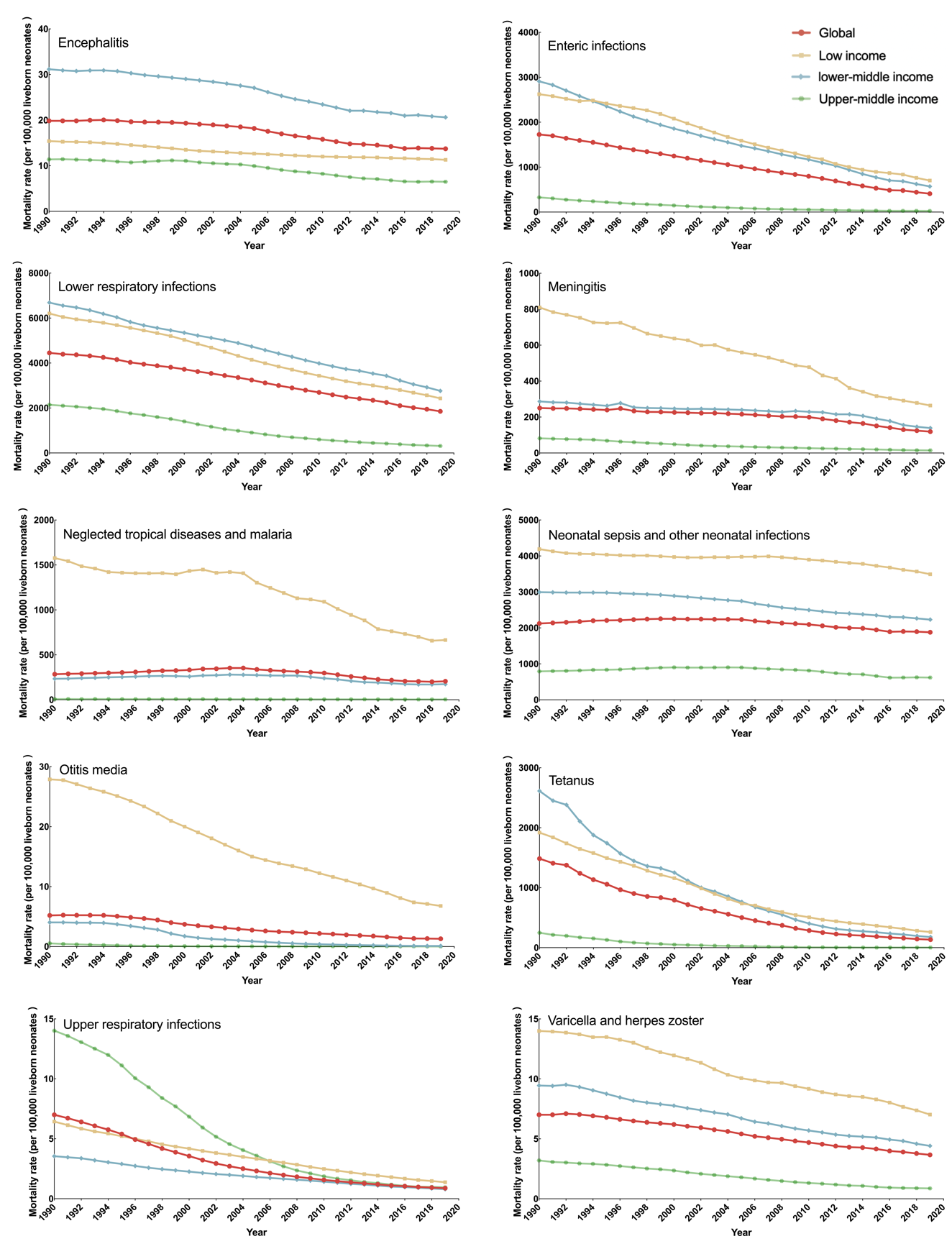
**

## Supplementary Figure S6. Female mortality rates of neonatal infectious diseases globally and in low- and middle-income regions over 30 years, 1990–2019

Notes: LMICs, low- and middle-income countries and territories.

# Supplementary Tables

Notes: It is imperative to clarify upfront that certain supplementary tables may indicate zero incident numbers or deaths for a specific disease in a particular country for both 1990 and 2019, while the relative percentage change is not zero. This anomaly stems from our approach to retaining only integers during the calculation of absolute numbers, while the computation of relative percentage change adheres to the original decimal places.

## Supplementary Table S1. 131 LMICs as per Word Bank Classification (2019) ^†^

| **Word Bank income levels** | **Number** | **Countries** |
| --- | --- | --- |
| Low-income countries and territories | 29 | Afghanistan; Burkina Faso; Burundi; Central African Republic; Chad; Democratic People’s Republic of Korea; Democratic Republic of the Congo; Eritrea; Ethiopia; Gambia; Guinea; Guinea-Bissau; Haiti; Liberia; Madagascar; Malawi; Mali; Mozambique; Niger; Rwanda; Sierra Leone; Somalia; South Sudan; Sudan; Syrian Arab Republic; Tajikistan; Togo; Uganda; Yemen |
| Lower-middle-income countries and territories | 48 | Algeria; Angola; Bangladesh; Benin; Bhutan; Bolivia (Plurinational State of); Cabo Verde; Cambodia; Cameroon; Comoros; Congo; Côte d’Ivoire; Djibouti; Egypt; El Salvador; Eswatini; Ghana; Honduras; India; Kenya; Kiribati; Kyrgyzstan; Lao People’s Democratic Republic; Lesotho; Mauritania; Micronesia (Federated States of); Mongolia; Morocco; Myanmar; Nepal; Nicaragua; Nigeria; Pakistan; Papua New Guinea; Philippines; Republic of Moldova; Senegal; Solomon Islands; Sri Lanka; Timor-Leste; Tunisia; Ukraine; United Republic of Tanzania; Uzbekistan; Vanuatu; Viet Nam; Zambia; Zimbabwe |
| Upper-middle-income countries and territories | 54 | Albania; American Samoa; Armenia; Azerbaijan; Belarus; Belize; Bosnia and Herzegovina; Botswana; Brazil; Bulgaria; China; Colombia; Costa Rica; Cuba; Dominica; Dominican Republic; Ecuador; Equatorial Guinea; Fiji; Gabon; Georgia; Grenada; Guatemala; Guyana; Indonesia; Iran (Islamic Republic of); Iraq; Jamaica; Jordan; Kazakhstan; Lebanon; Libya; Malaysia; Maldives; Marshall Islands; Mexico; Montenegro; Namibia; North Macedonia; Paraguay; Peru; Russian Federation; Saint Lucia; Saint Vincent and the Grenadines; Samoa; Serbia; South Africa; Suriname; Thailand; Tonga; Turkey; Turkmenistan; Tuvalu; Venezuela (Bolivarian Republic of) |

Note: LMICs: low- and middle-income countries and territories.

† According to World Bank Group country classifications by income level (<https://datahelpdesk.worldbank.org/knowledgebase/articles/906519-world-bank-country-and-lending-groups>), three economies shift from low-income countries and territories (2019) to lower-middle-income countries and territories (2022), two economies shift from lower-middle-income countries and territories (2019) to upper-middle income countries and territories (2022), two economies shift from upper-middle-income countries and territories (2019) to high-income countries and territories (2022), and four economies shift from upper-middle-income countries and territories (2019) to lower-middle-income countries and territories (2022), and Venezuela (Bolivarian Republic of) has no data for 2022.

## Supplementary Table S2. The SDI and UHCI of 131 low- and middle-income countries and territories in 2019.

| **Order** | **Location** | **SDI in 2019** | **UHCI in 2019** |
| --- | --- | --- | --- |
| 1 | Afghanistan | 0.343 | 39.295 |
| 2 | Albania | 0.681 | 69.626 |
| 3 | Algeria | 0.652 | 64.855 |
| 4 | American Samoa | 0.712 | 53.197 |
| 5 | Angola | 0.470 | 39.158 |
| 6 | Armenia | 0.689 | 62.435 |
| 7 | Azerbaijan | 0.683 | 48.181 |
| 8 | Bangladesh | 0.483 | 53.883 |
| 9 | Belarus | 0.745 | 70.462 |
| 10 | Belize | 0.603 | 54.280 |
| 11 | Benin | 0.352 | 44.624 |
| 12 | Bhutan | 0.455 | 51.301 |
| 13 | Bolivia (Plurinational State of) | 0.566 | 52.399 |
| 14 | Bosnia and Herzegovina | 0.718 | 64.184 |
| 15 | Botswana | 0.634 | 57.518 |
| 16 | Brazil | 0.64 | 64.828 |
| 17 | Bulgaria | 0.764 | 62.557 |
| 18 | Burkina Faso | 0.257 | 41.797 |
| 19 | Burundi | 0.284 | 49.940 |
| 20 | Cabo Verde | 0.525 | 62.188 |
| 21 | Cambodia | 0.469 | 57.079 |
| 22 | Cameroon | 0.490 | 42.290 |
| 23 | Central African Republic | 0.274 | 22.300 |
| 24 | Chad | 0.238 | 31.372 |
| 25 | China | 0.686 | 69.712 |
| 26 | Colombia | 0.633 | 74.397 |
| 27 | Comoros | 0.455 | 48.139 |
| 28 | Congo | 0.568 | 43.904 |
| 29 | Costa Rica | 0.68 | 79.015 |
| 30 | Cuba | 0.668 | 72.592 |
| 31 | Côte d'Ivoire | 0.408 | 43.040 |
| 32 | Democratic People's Republic of Korea | 0.878 | 52.838 |
| 33 | Democratic Republic of the Congo | 0.382 | 45.169 |
| 34 | Djibouti | 0.459 | 45.287 |
| 35 | Dominica | 0.729 | 51.809 |
| 36 | Dominican Republic | 0.592 | 52.500 |
| 37 | Ecuador | 0.640 | 64.454 |
| 38 | Egypt | 0.658 | 54.797 |
| 39 | El Salvador | 0.573 | 61.678 |
| 40 | Equatorial Guinea | 0.685 | 49.994 |
| 41 | Eritrea | 0.396 | 42.275 |
| 42 | Eswatini | 0.577 | 53.397 |
| 43 | Ethiopia | 0.343 | 46.522 |
| 44 | Fiji | 0.664 | 45.176 |
| 45 | Gabon | 0.656 | 53.050 |
| 46 | Gambia | 0.399 | 48.065 |
| 47 | Georgia | 0.841 | 55.953 |
| 48 | Ghana | 0.557 | 49.139 |
| 49 | Grenada | 0.669 | 50.484 |
| 50 | Guatemala | 0.526 | 52.099 |
| 51 | Guinea | 0.325 | 32.335 |
| 52 | Guinea-Bissau | 0.355 | 35.707 |
| 53 | Guyana | 0.618 | 40.622 |
| 54 | Haiti | 0.432 | 35.812 |
| 55 | Honduras | 0.496 | 54.284 |
| 56 | India | 0.566 | 46.826 |
| 57 | Indonesia | 0.660 | 48.728 |
| 58 | Iran (Islamic Republic of) | 0.670 | 69.515 |
| 59 | Iraq | 0.671 | 57.725 |
| 60 | Jamaica | 0.684 | 56.840 |
| 61 | Jordan | 0.731 | 69.967 |
| 62 | Kazakhstan | 0.723 | 59.237 |
| 63 | Kenya | 0.508 | 51.647 |
| 64 | Kiribati | 0.527 | 35.736 |
| 65 | Kyrgyzstan | 0.596 | 52.952 |
| 66 | Lao People's Democratic Republic | 0.490 | 43.855 |
| 67 | Lebanon | 0.708 | 74.533 |
| 68 | Lesotho | 0.507 | 38.737 |
| 69 | Liberia | 0.370 | 47.600 |
| 70 | Libya | 0.709 | 66.328 |
| 71 | Madagascar | 0.396 | 39.691 |
| 72 | Malawi | 0.384 | 55.521 |
| 73 | Malaysia | 0.737 | 66.574 |
| 74 | Maldives | 0.562 | 66.857 |
| 75 | Mali | 0.263 | 40.661 |
| 76 | Marshall Islands | 0.544 | 44.004 |
| 77 | Mauritania | 0.496 | 53.278 |
| 78 | Mexico | 0.649 | 61.437 |
| 79 | Micronesia (Federated States of) | 0.580 | 34.474 |
| 80 | Mongolia | 0.606 | 47.907 |
| 81 | Montenegro | 0.791 | 65.959 |
| 82 | Morocco | 0.548 | 58.032 |
| 83 | Mozambique | 0.307 | 44.044 |
| 84 | Myanmar | 0.521 | 46.954 |
| 85 | Namibia | 0.612 | 62.169 |
| 86 | Nepal | 0.422 | 47.280 |
| 87 | Nicaragua | 0.517 | 57.159 |
| 88 | Niger | 0.162 | 35.026 |
| 89 | Nigeria | 0.515 | 38.339 |
| 90 | North Macedonia | 0.744 | 60.746 |
| 91 | Pakistan | 0.449 | 39.168 |
| 92 | Papua New Guinea | 0.394 | 37.767 |
| 93 | Paraguay | 0.638 | 63.351 |
| 94 | Peru | 0.648 | 75.759 |
| 95 | Philippines | 0.623 | 54.712 |
| 96 | Republic of Moldova | 0.696 | 62.194 |
| 97 | Russian Federation | 0.805 | 68.974 |
| 98 | Rwanda | 0.429 | 59.359 |
| 99 | Saint Lucia | 0.670 | 59.140 |
| 100 | Saint Vincent and the Grenadines | 0.627 | 49.494 |
| 101 | Samoa | 0.641 | 49.796 |
| 102 | Senegal | 0.389 | 49.610 |
| 103 | Serbia | 0.767 | 63.349 |
| 104 | Sierra Leone | 0.347 | 42.120 |
| 105 | Solomon Islands | 0.407 | 39.333 |
| 106 | Somalia | 0.081 | 23.940 |
| 107 | South Africa | 0.678 | 59.727 |
| 108 | South Sudan | 0.363 | 41.694 |
| 109 | Sri Lanka | 0.690 | 65.563 |
| 110 | Sudan | 0.515 | 51.835 |
| 111 | Suriname | 0.636 | 50.134 |
| 112 | Syrian Arab Republic | 0.619 | 57.565 |
| 113 | Tajikistan | 0.539 | 47.878 |
| 114 | Thailand | 0.687 | 71.600 |
| 115 | Timor-Leste | 0.514 | 45.954 |
| 116 | Togo | 0.417 | 42.809 |
| 117 | Tonga | 0.636 | 52.417 |
| 118 | Tunisia | 0.672 | 68.106 |
| 119 | Turkey | 0.748 | 69.210 |
| 120 | Turkmenistan | 0.670 | 44.013 |
| 121 | Tuvalu | 0.589 | 39.570 |
| 122 | Uganda | 0.404 | 52.748 |
| 123 | Ukraine | 0.736 | 56.752 |
| 124 | United Republic of Tanzania | 0.423 | 55.249 |
| 125 | Uzbekistan | 0.631 | 42.185 |
| 126 | Vanuatu | 0.485 | 34.084 |
| 127 | Venezuela (Bolivarian Republic of) | 0.607 | 60.968 |
| 128 | Viet Nam | 0.617 | 59.707 |
| 129 | Yemen | 0.412 | 49.046 |
| 130 | Zambia | 0.505 | 52.694 |
| 131 | Zimbabwe | 0.476 | 54.461 |
| SDI, sociodemographic index; UHCI, universal health coverage index. | | | |

## Supplementary Table S3. Incidence rates (per 100,000 liveborn neonates) of neonatal infectious diseases in LMICs with estimated annual percentage change (EAPC) from 1990 to 2019.

| **Neonatal infections** | **LICs** | | | **LMCs** | | | **UMCs** | | |
| --- | --- | --- | --- | --- | --- | --- | --- | --- | --- |
|  | **Incidence rate, 1990**  **(95% UI)** | **Incidence rate, 2019 (95% UI)** | **EAPC, 1990-2019 (%, 95% CI)** | **Incidence rate, 1990**  **(95% UI)** | **Incidence rate, 2019 (95% UI)** | **EAPC, 1990-2019 (%, 95% CI)** | **Incidence rate, 1990**  **(95% UI)** | **Incidence rate, 2019 (95% UI)** | **EAPC, 1990-2019 (%, 95% CI)** |
| Acute hepatitis | 52393.74 (40676.97 to 63588.04) | 44703.34 (33829.51 to 57591.31) | -0.42  (-0.47 to -0.36) | 39220.84 (30160.45 to 49659.57) | 29972.84 (22871.71 to 38330.56) | -1.06  (-1.17 to -0.96) | 25322.14 (19673.12 to 31917.99) | 17922.54 (13715.48 to 22940.20) | -1.28  (-1.37 to -1.18) |
| Bacterial skin diseases | 20181.55 (19012.06 to 21514.89) | 20114.41 (18896.32 to 21374.01) | 0.01  (0.00 to 0.03) | 17921.01 (16810.45 to 19133.27) | 18776.73 (17607.83 to 20059.57) | 0.17  (0.16 to 0.17) | 23480.03 (22105.32 to 24965.84) | 22590.05 (21310.11 to 24056.14) | -0.11  (-0.19 to -0.04) |
| Encephalitis | 76.70 (59.35 to 96.4) | 63.47 (49.44 to 80.27) | -0.84  (-0.90 to -0.78) | 201.12 (157.32 to 248.85) | 133.31 (104.40 to 165.40) | -1.58  (-1.69 to -1.47) | 100.64 (82.19 to 122.28) | 69.75 (56.44 to 85.39) | -1.30  (-1.40 to -1.21) |
| Enteric infections | 359093.11 (334308.61 to 373100.67) | 348418.42 (319148.23 to 366100.03) | -0.13  (-0.16 to -0.11) | 331904.48 (297388.86 to 355807.36) | 253252.51 (219382.43 to 281798.07) | -0.44  (-0.64 to -0.23) | 328688.52 (292949.81 to 353806.48) | 219133.87 (185901.76 to 249313.86) | -1.74  (-1.9 to -1.58) |
| Fungal skin diseases | 19012.47 (14218.92 to 25491.97) | 18531.59 (13945.39 to 24656.94) | -0.16  (-0.20 to -0.12) | 21611.51 (14959.76 to 30742) | 19691.76 (13858.89 to 27838.06) | -0.36  (-0.38 to -0.34) | 6541.98 (5061 to 8280.54) | 6314.6 (4822.45 to 8030.91) | -0.16  (-0.19 to -0.13) |
| Lower respiratory infections | 17284.03 (12995.36 to 22262.65) | 9761.45 (7355.62 to 12508.96) | -2.12  (-2.23 to -2.00) | 12885.08 (9789.91 to 16202.13) | 6956.7 (5309.19 to 8844.22) | -2.17  (-2.37 to -1.97) | 24536.51 (20410.75 to 29363.96) | 6263.67 (5009.28 to 7821.12) | -5.18  (-5.35 to -5.01) |
| Meningitis | 3744.21 (2784.02 to 4950.10) | 1958.1 (1433.78 to 2665.56) | -2.69  (-3.00 to -2.37) | 1550.71 (1092.99 to 2128.39) | 946.63 (657.61 to 1324.75) | -2.04  (-2.35 to -1.73) | 476.87 (330.65 to 668.50) | 154.44 (106.9 to 220.44) | -3.53  (-3.93 to -3.14) |
| Neglected tropical diseases and malaria | 25509.99 (20157.43 to 31484.91) | 14148.32 (9006.20 to 19757.46) | -2.01  (-2.34 to -1.68) | 6669.26 (5152.09 to 9045.57) | 4696.94 (2754.56 to 6947.06) | -1.39  (-1.61 to -1.16) | 164.95 (130.58 to 237.11) | 131.95 (55.98 to 300.08) | -1.49  (-1.89 to -1.09) |
| Neonatal sepsis and other neonatal infections | 53947.54 (38330.81 to 73447.61) | 57407.8 (40627.45 to 77648.58) | 0.30  (0.23 to 0.36) | 52117.44 (36706.87 to 71159.97) | 54799.72 (38678.70 to 75041.60) | 0.06  (0.02 to 0.09) | 56955.27 (39519.23 to 78332.43) | 80265.09 (55104.68 to 109615.10) | 1.34  (1.26 to 1.42) |
| Otitis media | 19946.26 (11776.02 to 31837.13) | 20199.37 (11940.67 to 32249.99) | 0.04  (0.04 to 0.05) | 19558.79 (11501.13 to 30944.91) | 19990.19 (11743.36 to 31755.56) | 0.05  (0.05 to 0.06) | 12927.55 (7536.23 to 20504.54) | 14042.87 (8202.80 to 22288.82) | 0.25  (0.13 to 0.36) |
| Scabies | 5302.12 (4058.82 to 6713.69) | 4600.97 (3500.07 to 5796.75) | -0.49  (-0.52 to -0.47) | 10138.31 (7754.77 to 12939.03) | 9385.67 (7192.30 to 12017.49) | -0.29  (-0.34 to -0.23) | 12945.12 (9861.26 to 16468.69) | 12135.07 (9249.23 to 15367.07) | -0.31  (-0.38 to -0.23) |
| Tetanus | 3392.1 (2019.72 to 5085.21) | 414.87 (249.90 to 783.31) | -7.12  (-7.26 to -6.97) | 6160.07 (4441.51 to 8334.13) | 393.54 (247.30 to 605.61) | -9.25  (-9.55 to -8.95) | 1270.08 (876.43 to 1840.82) | 7.53 (4.61 to 11.7) | -17.96  (-18.68 to -17.22) |
| Tuberculosis | 280.03 (189.39 to 390.55) | 126.14 (89.46 to 172.07) | -2.65  (-2.72 to -2.57) | 147.68 (101.49 to 205.26) | 68.34 (47.87 to 92.81) | -2.61  (-2.69 to -2.53) | 85.98 (57.64 to 123.53) | 22.75 (15.82 to 31.16) | -5.14  (-5.54 to -4.73) |
| Upper respiratory infections | 304266.1 (234975.93 to 392773.57) | 292946.03 (226327.99 to 381567.19) | -0.33  (-0.39 to -0.27) | 301132.19 (232627.93 to 387353.58) | 284464.85 (219326.3 to 367964.85) | -0.27  (-0.41 to -0.12) | 309028.22 (237895.19 to 399308.37) | 319174.17 (245033.74 to 415334.99) | 0.00  (-0.07 to 0.06) |
| Varicella and herpes zoster | 25252.78 (13560.86 to 43309.50) | 21619.49 (11383.93 to 37340.26) | -0.59  (-0.63 to -0.55) | 22251.08 (11978.28 to 37827.00) | 19922.93 (10618.2 to 34254.24) | -0.30  (-0.33 to -0.26) | 23856.84 (12806.50 to 41348.29) | 21356.6 (11286.59 to 37474.12) | -0.42  (-0.45 to -0.4) |

CI, confidence interval; EAPC, estimated annual percentage change; LICs, low-income countries and territories; LMCs, lower-middle-income countries and territories; LMICs, low- and middle-income countries and territories; UI, uncertain interval; UMCs, upper-middle-income countries and territories.

## Supplementary Table S4. Incident cases of neonatal infectious diseases in LMICs and their relative percent change from 1990 to 2019.

| **Neonatal infections** | **LICs** | | | **LMCs** | | | **UMCs** | | |
| --- | --- | --- | --- | --- | --- | --- | --- | --- | --- |
|  | **Incident cases, 1990, No. × 10^5^**  **(95% UI)** | **Incident cases, 2019, No. × 10^5^**  **(95% UI)** | **Percent change, 1990-2019**  **(%, 95% CI)** | **Incident cases, 1990, No. × 10^5^**  **(95% UI)** | **Incident cases, 2019, No. × 10^5^**  **(95% UI)** | **Percent change, 1990-2019**  **(%, 95% CI)** | **Incident cases, 1990, No. × 10^5^**  **(95% UI)** | **Incident cases, 2019, No. × 10^5^**  **(95% UI)** | **Percent change, 1990-2019**  **(%, 95% CI)** |
| Acute hepatitis | 5.96 (4.63 to 7.23) | 8.24 (6.23 to 10.61) | 38.28  (31.18 to 49.53) | 18.37 (14.13 to 23.26) | 14.71 (11.23 to 18.82) | -19.9  (-22.58 to -17.11) | 8.69 (6.75 to 10.95) | 4.54 (3.48 to 5.81) | -47.73  (-50.12 to -45.67) |
| Bacterial skin diseases | 2.29 (2.16 to 2.45) | 3.71 (3.48 to 3.94) | 61.53  (59.01 to 63.6) | 8.39 (7.87 to 8.96) | 9.22 (8.64 to 9.85) | 9.82  (8.77 to 10.73) | 8.06 (7.59 to 8.57) | 5.73 (5.40 to 6.10) | -28.95  (-29.85 to -28.26) |
| Encephalitis | 0.01 (0.01 to 0.01) | 0.01 (0.01 to 0.01) | 34.1  (30.17 to 37.51) | 0.09 (0.07 to 0.12) | 0.07 (0.05 to 0.08) | -30.52  (-32.71 to -28.64) | 0.03 (0.03 to 0.04) | 0.02 (0.01 to 0.02) | -48.82  (-50.07 to -47.48) |
| Enteric infections | 40.83 (38.01 to 42.42) | 64.21 (58.81 to 67.46) | 57.26  (52.53 to 60.72) | 155.44 (139.28 to 166.64) | 124.32 (107.70 to 138.34) | -20.02  (-23.32 to -15.95) | 112.81 (100.55 to 121.43) | 55.54 (47.12 to 63.19) | -50.77  (-53.71 to -47.26) |
| Fungal skin diseases | 2.16 (1.62 to 2.90) | 3.41 (2.57 to 4.54) | 57.97  (51.62 to 65.31) | 10.12 (7.01 to 14.4) | 9.67 (6.80 to 13.67) | -4.49  (-6.94 to -1.67) | 2.25 (1.74 to 2.84) | 1.60 (1.22 to 2.04) | -28.72  (-31.55 to -26.57) |
| Lower respiratory infections | 1.97 (1.48 to 2.53) | 1.80 (1.36 to 2.31) | -8.47  (-13.11 to -3.63) | 6.03 (4.58 to 7.59) | 3.42 (2.61 to 4.34) | -43.41  (-44.89 to -41.90) | 8.42 (7.01 to 10.08) | 1.59 (1.27 to 1.98) | -81.15  (-82.46 to -79.87) |
| Meningitis | 0.43 (0.32 to 0.56) | 0.36 (0.26 to 0.49) | -15.24  (-19.25 to -11.04) | 0.73 (0.51 to 1.00) | 0.46 (0.32 to 0.65) | -36.01  (-37.70 to -34.24) | 0.16 (0.11 to 0.23) | 0.04 (0.03 to 0.06) | -76.08  (-77.08 to -74.99) |
| Neglected tropical diseases and malaria | 2.90 (2.29 to 3.58) | 2.61 (1.66 to 3.64) | -10.11  (-32.20 to 12.03) | 3.12 (2.41 to 4.24) | 2.31 (1.35 to 3.41) | -26.18  (-54.41 to 1.66) | 0.06 (0.04 to 0.08) | 0.03 (0.01 to 0.08) | -40.93  (-76.83 to 45.19) |
| Neonatal sepsis and other neonatal infections | 6.13 (4.36 to 8.35) | 10.58 (7.49 to 14.31) | 72.47  (66.43 to 79.38) | 24.41 (17.19 to 33.33) | 26.90 (18.99 to 36.84) | 10.21  (6.71 to 13.89) | 19.55 (13.56 to 26.89) | 20.34 (13.97 to 27.78) | 4.07  (-2.43 to 10.04) |
| Otitis media | 2.27 (1.34 to 3.62) | 3.72 (2.20 to 5.94) | 64.13  (63.43 to 64.96) | 9.16 (5.39 to 14.49) | 9.81 (5.76 to 15.59) | 7.13  (5.72 to 8.39) | 4.44 (2.59 to 7.04) | 3.56 (2.08 to 5.65) | -19.78  (-20.43 to -19.19) |
| Scabies | 0.60 (0.46 to 0.76) | 0.85 (0.64 to 1.07) | 40.64  (37.04 to 44.18) | 4.75 (3.63 to 6.06) | 4.61 (3.53 to 5.90) | -2.96  (-4.21 to -1.67) | 4.44 (3.38 to 5.65) | 3.08 (2.34 to 3.89) | -30.77  (-31.78 to -29.77) |
| Tetanus | 0.39 (0.23 to 0.58) | 0.08 (0.05 to 0.14) | -80.18  (-88.53 to -47.36) | 2.88 (2.08 to 3.90) | 0.19 (0.12 to 0.30) | -93.3  (-95.77 to -89.11) | 0.44 (0.30 to 0.63) | 0.0019 (0.0012 to 0.0030) | -99.56  (-99.7 to -99.27) |
| Tuberculosis | 0.03 (0.02 to 0.04) | 0.02 (0.02 to 0.03) | -26.99  (-35.3 to -16.74) | 0.07 (0.05 to 0.10) | 0.03 (0.02 to 0.05) | -51.49  (-55.04 to -47.2) | 0.03 (0.02 to 0.04) | 0.0058 (0.0040 to 0.0079) | -80.46  (-82.35 to -78.08) |
| Upper respiratory infections | 34.60 (26.72 to 44.66) | 53.98 (41.71 to 70.31) | 56.04  (46.63 to 62.48) | 141.03 (108.95 to 181.41) | 139.64 (107.67 to 180.63) | -0.98  (-3.09 to 0.79) | 106.06 (81.65 to 137.05) | 80.9 (62.11 to 105.27) | -23.73  (-24.89 to -22.64) |
| Varicella and herpes zoster | 2.87 (1.54 to 4.92) | 3.98 (2.10 to 6.88) | 38.75  (25.79 to 49.71) | 10.42 (5.61 to 17.72) | 9.78 (5.21 to 16.82) | -6.15  (-9.89 to -2.09) | 8.19 (4.4 to 14.19) | 5.41 (2.86 to 9.50) | -33.89  (-38.46 to -28.94) |

CI, confidence interval; LICs, low-income countries and territories; LMCs, lower-middle-income countries and territories; LMICs, low- and middle-income countries and territories; UI, uncertain interval; UMCs, upper-middle-income countries and territories

## Supplementary Table S5. Mortality rates (per 100,000 liveborn neonates) of neonatal infectious diseases in LMICs with estimated annual percentage change (EAPC) from 1990 to 2019.

| **Neonatal infections** | **LICs** | | | **LMCs** | | | **UMCs** | | |
| --- | --- | --- | --- | --- | --- | --- | --- | --- | --- |
|  | **Mortality rate, 1990**  **(95% UI)** | **Mortality rate, 2019 (95% UI)** | **EAPC, 1990-2019 (%, 95% CI)** | **Mortality rate, 1990**  **(95% UI)** | **Mortality rate, 2019 (95% UI)** | **EAPC, 1990-2019 (%, 95% CI)** | **Mortality rate, 1990**  **(95% UI)** | **Mortality rate, 2019 (95% UI)** | **EAPC, 1990-2019 (%, 95% CI)** |
| Encephalitis | 16.01 (9.59 to 21.80) | 11.36 (8.57 to 14.66) | -1.23  (-1.32 to -1.15) | 33.13 (23.06 to 41.5) | 20.88 (15.62 to 27.63) | -1.81  (-1.92 to -1.70) | 13.40 (8.26 to 15.94) | 6.51 (5.02 to 8.02) | -2.87  (-3.16 to -2.59) |
| Enteric infections | 2993.01 (2369.60 to 3575.19) | 823.47 (622.38 to 1077.48) | -4.49  (-4.85 to -4.14) | 3265.23 (2208.78 to 4230.15) | 569.50 (455.69 to 707.85) | -5.68  (-5.93 to -5.42) | 369.18 (321.24 to 414.76) | 28.27 (22.96 to 34.7) | -9.13  (-9.41 to -8.85) |
| Lower respiratory infections | 7282.55 (6129.20 to 8802.76) | 2626.91 (2063.26 to 3296.71) | -3.58  (-3.74 to -3.41) | 7701.49 (6653.61 to 8857.73) | 2820.08 (2327.34 to 3407.33) | -3.31  (-3.43 to -3.19) | 2397.68 (2148.47 to 2628.63) | 355.61 (304.94 to 413.87) | -6.85  (-7.12 to -6.58) |
| Meningitis | 892.41 (746.14 to 1096.83) | 300.89 (232.74 to 396.32) | -3.65  (-4.01 to -3.28) | 358.96 (300.46 to 430.90) | 162.80 (129.39 to 211.58) | -2.20  (-2.55 to -1.84) | 97.69 (86.82 to 110.52) | 16.28 (13.58 to 19.36) | -6.14  (-6.29 to -5.98) |
| Neglected tropical diseases and malaria | 1510 (705.76 to 2684.30) | 625.67 (260.25 to 1206.04) | -2.97  (-3.45 to -2.49) | 246.97 (135.26 to 444.40) | 166.85 (79.73 to 311.93) | -1.58  (-2.05 to -1.10) | 7.30 (4.82 to 10.56) | 3.56 (2.14 to 5.80) | -2.68  (-2.90 to -2.46) |
| Neonatal sepsis and other neonatal infections | 4874.61 (3748.72 to 5881.66) | 3974.60 (3040.74 to 5112.97) | -0.54  (-0.65 to -0.43) | 3227.61 (2466.16 to 3839.90) | 2382.85 (1990.30 to 2864.04) | -1.14  (-1.25 to -1.03) | 930.72 (839.34 to 1022.48) | 721.32 (594.91 to 867.65) | -1.03  (-1.41 to -0.65) |
| Otitis media | 34.38 (1.13 to 130.6) | 10.57 (0.33 to 42.13) | -3.90  (-4.03 to -3.77) | 5.45 (0.83 to 14.13) | 0.22 (0.05 to 0.70) | -11.96  (-12.48 to -11.44) | 0.62 (0.44 to 0.95) | 0.05 (0.03 to 0.08) | -7.71  (-9.20 to -6.19) |
| Tetanus | 2037.36 (1340.58 to 2795.33) | 272.65 (174.37 to 528.42) | -6.90  (-7.14 to -6.67) | 2843.65 (2283.07 to 3392.85) | 186.04 (130.16 to 260.40) | -9.26  (-9.56 to -8.96) | 339.06 (265.64 to 426.93) | 2.49 (1.68 to 3.69) | -17.22  (-17.88 to -16.54) |
| Upper respiratory infections | 8.00 (0.29 to 20.08) | 1.94 (0.21 to 4.48) | -4.71  (-4.89 to -4.52) | 3.56 (1.16 to 6.86) | 0.94 (0.48 to 1.45) | -4.46  (-4.60 to -4.31) | 13.36 (4.70 to 20.39) | 1.09 (0.72 to 1.80) | -9.43  (-9.76 to -9.09) |
| Varicella and herpes zoster | 14.15 (10.86 to 17.87) | 7.08 (5.26 to 9.42) | -2.41  (-2.51 to -2.31) | 9.34 (7.52 to 11.68) | 4.39 (3.50 to 5.55) | -2.68  (-2.76 to -2.60) | 3.37 (2.80 to 4.09) | 1.03 (0.74 to 1.42) | -4.40  (-4.60 to -4.20) |

CI, confidence interval; EAPC, estimated annual percentage change; LICs, low-income countries and territories; LMCs, lower-middle-income countries and territories; LMICs, low- and middle-income countries and territories; UI, uncertain interval; UMCs, upper-middle-income countries and territories

## Supplementary Table S6. Deaths of neonatal infectious diseases in LMICs and their relative percent change from 1990 to 2019.

| **Neonatal infections** | **LICs** | | | **LMCs** | | | **UMCs** | | |
| --- | --- | --- | --- | --- | --- | --- | --- | --- | --- |
|  | **Deaths, 1990,**  **No. × 10^3^**  **(95% UI)** | **Deaths, 2019,**  **No. × 10^3^**  **(95% UI)** | **Percent change, 1990-2019 (%, 95% CI)** | **Deaths, 1990,**  **No. × 10^3^**  **(95% UI)** | **Deaths, 2019,**  **No. × 10^3^**  **(95% UI)** | **Percent change, 1990-2019 (%, 95% CI)** | **Deaths, 1990,**  **No. × 10^3^**  **(95% UI)** | **Deaths, 2019,**  **No. × 10^3^**  **(95% UI)** | **Percent change, 1990-2019 (%, 95% CI)** |
| Encephalitis | 0.18 (0.11 to 0.25) | 0.21 (0.16 to 0.27) | 14.97  (-25.33 to 76.57) | 1.55 (1.08 to 1.94) | 1.02 (0.77 to 1.36) | -33.96  (-57.11 to 3.03) | 0.46 (0.28 to 0.55) | 0.16 (0.13 to 0.20) | -64.12  (-72.80 to -42.88) |
| Enteric infections | 34.03 (26.94 to 40.65) | 15.17 (11.47 to 19.86) | -55.41  (-66.58 to -40.32) | 152.92 (103.44 to 198.11) | 27.96 (22.37 to 34.75) | -81.72  (-87.45 to -70.73) | 12.67 (11.03 to 14.24) | 0.72 (0.58 to 0.88) | -94.34  (-95.50 to -92.84) |
| Lower respiratory infections | 82.80 (69.69 to 100.09) | 48.41 (38.02 to 60.75) | -41.54  (-56.14 to -22.46) | 360.68 (311.61 to 414.83) | 138.44 (114.25 to 167.27) | -61.62  (-69.83 to -50.84) | 82.29 (73.74 to 90.22) | 9.01 (7.73 to 10.49) | -89.05  (-90.89 to -86.87) |
| Meningitis | 10.15 (8.48 to 12.47) | 5.54 (4.29 to 7.30) | -45.36  (-58.34 to -28.92) | 16.81 (14.07 to 20.18) | 7.99 (6.35 to 10.39) | -52.46  (-63.42 to -38.13) | 3.35 (2.98 to 3.79) | 0.41 (0.34 to 0.49) | -87.69  (-90.21 to -85.04) |
| Neglected tropical diseases and malaria | 17.17 (8.02 to 30.52) | 11.53 (4.80 to 22.22) | -32.85  (-64.97 to 20.07) | 11.57 (6.33 to 20.81) | 8.19 (3.91 to 15.31) | -29.18  (-62.43 to 20.10) | 0.25 (0.17 to 0.36) | 0.09 (0.05 to 0.15) | -63.98  (-77.33 to -43.83) |
| Neonatal sepsis and other neonatal infections | 55.42 (42.62 to 66.88) | 73.24 (56.03 to 94.22) | 32.15  (0.92 to 76.41) | 151.16 (115.50 to 179.83) | 116.97 (97.70 to 140.60) | -22.61  (-39.60 to 6.29) | 31.94 (28.81 to 35.09) | 18.28 (15.08 to 21.99) | -42.77  (-53.64 to -30.06) |
| Otitis media | 0.39 (0.01 to 1.48) | 0.19 (0.01 to 0.78) | -50.15  (-79.40 to 14.60) | 0.26 (0.04 to 0.66) | 0.01 (0.00 to 0.03) | -95.72  (-98.50 to -85.88) | 0.02 (0.01 to 0.03) | 0.001 (0.0007 to 0.0021) | -94.16  (-96.85 to -88.91) |
| Tetanus | 23.16 (15.24 to 31.78) | 5.02 (3.21 to 9.74) | -78.31  (-87.37 to -41.75) | 133.18 (106.92 to 158.90) | 9.13 (6.39 to 12.78) | -93.14  (-95.57 to -89.1) | 11.64 (9.12 to 14.65) | 0.06 (0.04 to 0.09) | -99.46  (-99.63 to -99.09) |
| Upper respiratory infections | 0.09 (0.00 to 0.23) | 0.04 (0.00 to 0.08) | -60.82  (-75.77 to 36.57) | 0.17 (0.05 to 0.32) | 0.05 (0.02 to 0.07) | -72.25  (-83.26 to -32.51) | 0.46 (0.16 to 0.70) | 0.03 (0.02 to 0.05) | -93.99  (-96.88 to -74.51) |
| Varicella and herpes zoster | 0.16 (0.12 to 0.20) | 0.13 (0.10 to 0.17) | -18.93  (-43.76 to 16.45) | 0.44 (0.35 to 0.55) | 0.22 (0.17 to 0.27) | -50.69  (-64.16 to -34.69) | 0.12 (0.10 to 0.14) | 0.03 (0.02 to 0.04) | -77.48  (-83.07 to -69.87) |

CI, confidence interval; LICs, low-income countries and territories; LMCs, lower-middle-income countries and territories; LMICs, low- and middle-income countries and territories; UI, uncertain interval; UMCs, upper-middle-income countries and territories

## Supplementary Table S7. National burden and trends of incident cases and incidence rates (per 100,000 liveborn neonates) of acute hepatitis in 131 LMICs between 1990 and 2019.

| **Location** | **Incident cases in 1990**  **(95% UI)** | **Incident rate in 1990**  **( 95% UI)** | **Incident cases in 2019**  **(95% UI)** | **Incident rate in 2019**  **( 95% UI)** | **Percent change**  **(%, 95 CI)** | **EAPC**  **(%, 95 CI)** |
| --- | --- | --- | --- | --- | --- | --- |
| Global | 3390645 (2627142 to 4278893) | 32840.56 (25445.55 to 41443.81) | 2817006 (2147006 to 3601146) | 27568.11 (21011.27 to 35241.94) | -16.92  (-19.38 to -14.46) | -0.69  (-0.74 to -0.65) |
| Afghanistan | 12449 (9151 to 16365) | 32175.49 (23653.4 to 42298.39) | 34748 (25988 to 45717) | 31069.26 (23236.96 to 40877.16) | 179.13  (144.61 to 221.07) | -0.1  (-0.17 to -0.03) |
| Albania | 1580 (1170 to 2065) | 24303.58 (17999.14 to 31753.87) | 657 (485 to 862) | 24648.29 (18213.6 to 32349.69) | -58.43  (-63.86 to -52.52) | -0.09  (-0.19 to 0.01) |
| Algeria | 17661 (13158 to 23354) | 30408.48 (22655.25 to 40210.25) | 15459 (11647 to 19909) | 23310.51 (17562.98 to 30020.68) | -12.47  (-23.82 to 0.55) | -0.9  (-0.96 to -0.84) |
| American Samoa | 21 (16 to 27) | 16111.87 (12121.96 to 20637.07) | 13 (10 to 17) | 16334.96 (12405.38 to 20930.81) | -37.34  (-44.39 to -28.65) | 0  (-0.12 to 0.13) |
| Angola | 15911 (12188 to 20676) | 41986.69 (32161.95 to 54562.42) | 27109 (20794 to 34958) | 32514.48 (24940.41 to 41929.02) | 70.38  (50.53 to 92.51) | -0.87  (-0.96 to -0.77) |
| Armenia | 1414 (1084 to 1835) | 24621.05 (18878.81 to 31947.52) | 574 (439 to 735) | 20054.69 (15365.01 to 25702.52) | -59.44  (-63.97 to -53.79) | -0.81  (-0.86 to -0.75) |
| Azerbaijan | 4569 (3401 to 6009) | 31942.1 (23775.98 to 42013.58) | 2713 (2029 to 3521) | 24957.25 (18663.78 to 32390.12) | -40.62  (-47.76 to -32.14) | -1.02  (-1.14 to -0.89) |
| Bangladesh | 102211 (76032 to 133900) | 34918.16 (25974.62 to 45743.8) | 52931 (39112 to 69044) | 26052.92 (19251.03 to 33983.96) | -48.21  (-55.15 to -41.14) | -1.13  (-1.72 to -0.53) |
| Belarus | 2463 (1835 to 3309) | 22964.5 (17113.08 to 30856.16) | 1220 (922 to 1575) | 15527.48 (11738.38 to 20042.2) | -50.46  (-57.87 to -41.15) | -1.59  (-1.82 to -1.37) |
| Belize | 124 (92 to 165) | 27827.69 (20655.42 to 37008.44) | 127 (94 to 163) | 21864.55 (16241.56 to 28106.49) | 2.55  (-10.49 to 16.24) | -0.86  (-0.95 to -0.76) |
| Benin | 8542 (6497 to 10974) | 48008.83 (36518.29 to 61681.63) | 15295 (11407 to 19615) | 40445.44 (30163.81 to 51867.34) | 79.06  (58.64 to 99.23) | -0.55  (-0.59 to -0.51) |
| Bhutan | 687 (511 to 904) | 41779.95 (31103.04 to 55021.37) | 332 (246 to 429) | 32716.91 (24254.89 to 42294.81) | -51.71  (-57.56 to -45.49) | -0.93  (-0.99 to -0.87) |
| Bolivia (Plurinational State of) | 6472 (4802 to 8334) | 35441.14 (26295.56 to 45637.63) | 6246 (4627 to 8176) | 25604.36 (18965.64 to 33513.38) | -3.49  (-16.78 to 11.42) | -1.01  (-1.23 to -0.79) |
| Bosnia and Herzegovina | 811 (619 to 1049) | 15680.48 (11966.13 to 20281.19) | 253 (192 to 333) | 12372.72 (9393.71 to 16288.79) | -68.78  (-72.23 to -64.73) | -0.98  (-1.12 to -0.84) |
| Botswana | 1760 (1340 to 2211) | 57248.07 (43597.07 to 71942.14) | 1786 (1345 to 2291) | 49058.72 (36947.34 to 62942.66) | 1.48  (-9.15 to 14.44) | -0.49  (-0.51 to -0.47) |
| Brazil | 48337 (36931 to 61391) | 18623.78 (14229.3 to 23653.46) | 29861 (22674 to 38801) | 12707.85 (9649.46 to 16512.71) | -38.22  (-42.61 to -33.9) | -2.11  (-2.47 to -1.74) |
| Bulgaria | 1736 (1307 to 2245) | 23131.88 (17420.7 to 29921.81) | 1094 (798 to 1455) | 23895.52 (17423.1 to 31792.93) | -36.98  (-44.37 to -28.81) | 0.16  (0.11 to 0.21) |
| Burkina Faso | 16738 (12781 to 21689) | 48829.38 (37286.62 to 63273.73) | 29454 (22032 to 37859) | 41758.97 (31235.92 to 53675.34) | 75.97  (57.18 to 96.14) | -0.57  (-0.65 to -0.5) |
| Burundi | 12294 (9398 to 14934) | 62150.31 (47510.5 to 75496.3) | 18344 (13487 to 23805) | 52301.66 (38453.94 to 67874.02) | 49.2  (28.95 to 75.59) | -0.54  (-0.62 to -0.47) |
| Cabo Verde | 426 (320 to 549) | 47872.59 (36025.83 to 61756.19) | 287 (218 to 368) | 35312.83 (26727.41 to 45170.95) | -32.48  (-41.22 to -23.79) | -1.18  (-1.24 to -1.12) |
| Cambodia | 10398 (8087 to 13159) | 31719.05 (24669.56 to 40141.74) | 5681 (4358 to 7316) | 20502.09 (15727.58 to 26402.32) | -45.36  (-52.84 to -37.73) | -1.74  (-1.86 to -1.62) |
| Cameroon | 16743 (12838 to 21290) | 48050.76 (36842.32 to 61099.16) | 26907 (19994 to 34897) | 39583.18 (29413.84 to 51336.92) | 60.7  (39.6 to 81.17) | -0.5  (-0.58 to -0.42) |
| Central African Republic | 3796 (2879 to 4792) | 40358.02 (30612.5 to 50943.78) | 5191 (3915 to 6753) | 35225.62 (26563.74 to 45826) | 36.75  (22.8 to 52.81) | -0.44  (-0.5 to -0.38) |
| Chad | 11454 (8769 to 14838) | 48322.34 (36996.28 to 62597.19) | 26108 (19660 to 33522) | 44130.95 (33231 to 56663.08) | 127.94  (98.44 to 159.85) | -0.27  (-0.31 to -0.23) |
| China | 435162 (340807 to 549117) | 23895.56 (18714.36 to 30153.06) | 159157 (122374 to 204567) | 13994.17 (10759.97 to 17987.01) | -63.43  (-66.07 to -60.81) | -1.76  (-1.83 to -1.69) |
| Colombia | 22755 (17209 to 29274) | 32258.49 (24395.78 to 41500.27) | 13956 (10728 to 18115) | 23174.3 (17813.63 to 30081.6) | -38.67  (-45.28 to -29.1) | -1.2  (-1.38 to -1.02) |
| Comoros | 926 (713 to 1111) | 62922.17 (48438.62 to 75486.49) | 627 (467 to 805) | 50473.27 (37548 to 64719.42) | -32.22  (-41.04 to -20.25) | -0.73  (-0.82 to -0.65) |
| Congo | 2978 (2276 to 3833) | 39671.25 (30318.82 to 51053.97) | 3419 (2545 to 4432) | 31480.73 (23438.33 to 40806.37) | 14.79  (1.62 to 27.9) | -0.79  (-0.87 to -0.71) |
| Costa Rica | 1868 (1394 to 2365) | 30328.85 (22626.56 to 38395.2) | 1337 (991 to 1783) | 26420.04 (19587.39 to 35236.81) | -28.44  (-36.68 to -19.18) | -0.47  (-0.51 to -0.44) |
| Côte d'Ivoire | 19842 (15087 to 25248) | 46571.17 (35410.25 to 59257.88) | 26442 (19965 to 34148) | 39915.83 (30138.49 to 51548.32) | 33.26  (17.88 to 50.59) | -0.5  (-0.53 to -0.47) |
| Cuba | 3032 (2278 to 3941) | 21970.89 (16509.07 to 28560.27) | 1440 (1080 to 1883) | 18032.78 (13525.46 to 23581.42) | -52.51  (-59.45 to -46.18) | -0.66  (-0.73 to -0.59) |
| Democratic People's Republic of Korea | 10888 (8503 to 13804) | 20073.32 (15675.66 to 25448.16) | 3713 (2853 to 4864) | 14212.95 (10921.63 to 18619.55) | -65.9  (-70.51 to -60.66) | -1.37  (-1.49 to -1.26) |
| Democratic Republic of the Congo | 55036 (41302 to 71199) | 39290.69 (29485.83 to 50829.55) | 70903 (52634 to 92934) | 32222.65 (23919.95 to 42234.76) | 28.83  (13.84 to 45.06) | -0.59  (-0.68 to -0.51) |
| Djibouti | 1035 (794 to 1246) | 62377.41 (47863.84 to 75138.53) | 1293 (955 to 1677) | 49553.04 (36619.65 to 64287.25) | 24.93  (8.65 to 46.91) | -0.75  (-0.84 to -0.66) |
| Dominica | 31 (23 to 40) | 24091.88 (18023.2 to 30955.53) | 12 (9 to 16) | 19140.79 (14296.14 to 25031.12) | -61.34  (-65.61 to -56.74) | -0.8  (-0.86 to -0.73) |
| Dominican Republic | 5291 (3922 to 7021) | 29367.4 (21771 to 38972.15) | 3915 (2884 to 5110) | 22677.55 (16708.31 to 29601.73) | -26  (-34.61 to -16.4) | -1.69  (-2.02 to -1.35) |
| Ecuador | 5264 (3915 to 6863) | 23236.69 (17284.58 to 30294.93) | 6438 (4787 to 8391) | 24242.71 (18025.58 to 31596.09) | 22.31  (5.87 to 40.51) | 0.1  (0.05 to 0.15) |
| Egypt | 56535 (41638 to 74813) | 39794.37 (29308.23 to 52660.17) | 34983 (25733 to 45809) | 21727.53 (15982.28 to 28451.43) | -38.12  (-46.64 to -27.82) | -2.12  (-2.25 to -2) |
| El Salvador | 5031 (3800 to 6542) | 40619.75 (30679.39 to 52819.6) | 2566 (1929 to 3357) | 29995.4 (22545.96 to 39243.62) | -48.99  (-55.27 to -42.63) | -1.09  (-1.16 to -1.01) |
| Equatorial Guinea | 682 (517 to 890) | 39906.72 (30257.64 to 52055.22) | 843 (629 to 1106) | 28861.31 (21544.13 to 37875.99) | 23.52  (9.53 to 38.07) | -1.2  (-1.26 to -1.13) |
| Eritrea | 5993 (4692 to 7124) | 64267.97 (50313.45 to 76399.21) | 7741 (5810 to 10058) | 51556.38 (38697.1 to 66989.4) | 29.16  (10.93 to 52.53) | -0.73  (-0.77 to -0.68) |
| Eswatini | 1394 (1060 to 1749) | 58077.57 (44129.18 to 72826.6) | 1149 (854 to 1477) | 51738.66 (38458.12 to 66520.87) | -17.64  (-27.57 to -8.25) | -0.35  (-0.38 to -0.33) |
| Ethiopia | 115997 (89564 to 137799) | 62981.15 (48629.15 to 74818.53) | 148868 (112896 to 190799) | 54128.87 (41049.35 to 69374.93) | 28.34  (17.79 to 44.32) | 0.04  (-0.18 to 0.25) |
| Fiji | 244 (184 to 316) | 16910.16 (12786.89 to 21906.35) | 218 (162 to 283) | 16402.66 (12188.57 to 21290) | -10.46  (-22.34 to 0.78) | -0.06  (-0.15 to 0.04) |
| Gabon | 1129 (861 to 1438) | 38235.85 (29169.2 to 48688.27) | 921 (688 to 1181) | 28592.72 (21356.26 to 36662.67) | -18.42  (-27.6 to -9.22) | -0.94  (-0.98 to -0.9) |
| Gambia | 1611 (1208 to 2060) | 44722.87 (33516.79 to 57176.38) | 2090 (1569 to 2732) | 38950.81 (29235.33 to 50917.54) | 29.7  (15.23 to 45.77) | -0.47  (-0.53 to -0.42) |
| Georgia | 1738 (1311 to 2229) | 27493.6 (20739.55 to 35269.33) | 818 (620 to 1062) | 23544.01 (17855.56 to 30589.62) | -52.94  (-57.83 to -45.97) | -0.68  (-0.76 to -0.59) |
| Ghana | 22086 (16892 to 28060) | 48868.89 (37377.62 to 62087.58) | 25667 (19093 to 33330) | 39957.05 (29722.76 to 51885.06) | 16.22  (3.01 to 30.7) | -0.73  (-0.77 to -0.69) |
| Grenada | 36 (27 to 47) | 24068.53 (18017.5 to 31294.07) | 20 (15 to 26) | 18703.03 (14132.34 to 24618.18) | -45.49  (-51.86 to -37.23) | -0.87  (-0.96 to -0.78) |
| Guatemala | 12506 (9203 to 16506) | 49229.93 (36229.07 to 64975.85) | 15545 (11737 to 20357) | 49675.56 (37507.03 to 65054.62) | 24.3  (8.42 to 40.73) | -0.21  (-0.31 to -0.12) |
| Guinea | 11271 (8657 to 14231) | 48114.22 (36953.78 to 60751.74) | 14853 (11174 to 19040) | 41302.75 (31071.27 to 52945.73) | 31.78  (16.93 to 46.97) | -0.54  (-0.6 to -0.49) |
| Guinea-Bissau | 1668 (1264 to 2147) | 48172.98 (36523.97 to 62006.3) | 1906 (1436 to 2481) | 40713.68 (30687.98 to 53012.11) | 14.26  (1.13 to 28.41) | -0.58  (-0.66 to -0.5) |
| Guyana | 432 (321 to 568) | 25899.8 (19238.78 to 34033.12) | 221 (166 to 291) | 20425.64 (15300.52 to 26856.84) | -48.8  (-53.96 to -42) | -0.79  (-0.81 to -0.77) |
| Haiti | 5195 (3890 to 6800) | 28129.17 (21059.55 to 36816.42) | 6055 (4517 to 7999) | 24356.23 (18170.97 to 32179.96) | 16.54  (2 to 31.64) | -0.49  (-0.52 to -0.46) |
| Honduras | 4210 (3201 to 5358) | 31044.13 (23600.67 to 39508.54) | 4385 (3329 to 5716) | 24585.94 (18668.4 to 32054.25) | 4.13  (-8.65 to 20.14) | -0.84  (-0.86 to -0.82) |
| India | 898822 (683331 to 1148260) | 45780.5 (34804.7 to 58485.35) | 543015 (410175 to 703137) | 30633.03 (23139.13 to 39665.99) | -39.59  (-42.4 to -36.22) | -1.62  (-1.78 to -1.46) |
| Indonesia | 80065 (62077 to 101521) | 22316.29 (17302.59 to 28296.95) | 53266 (40706 to 68427) | 18288.33 (13976.14 to 23493.9) | -33.47  (-36.93 to -29.92) | -0.61  (-0.77 to -0.44) |
| Iran (Islamic Republic of) | 28530 (21849 to 36244) | 21217.45 (16249.22 to 26954.74) | 17157 (13010 to 21929) | 16729.96 (12686.11 to 21382.57) | -39.86  (-43.11 to -35.61) | -1.61  (-2.18 to -1.04) |
| Iraq | 17539 (13150 to 22725) | 33191.65 (24885.37 to 43005.34) | 18403 (13638 to 23972) | 25294.97 (18746.09 to 32949.93) | 4.93  (-6.71 to 20.25) | -0.95  (-1.07 to -0.83) |
| Jamaica | 885 (682 to 1132) | 20833.32 (16057.04 to 26644.51) | 485 (365 to 629) | 17707.64 (13337.06 to 22983.66) | -45.2  (-51.79 to -37.08) | -0.28  (-0.42 to -0.14) |
| Jordan | 3194 (2429 to 4156) | 30946.36 (23536.85 to 40260.66) | 4390 (3236 to 5674) | 23490.92 (17317.15 to 30362.04) | 37.43  (21.15 to 58.39) | -1  (-1.19 to -0.81) |
| Kazakhstan | 7207 (5456 to 9404) | 26759.64 (20256.21 to 34913.68) | 5328 (4072 to 6898) | 19825.97 (15151.7 to 25669.66) | -26.08  (-34.32 to -15.72) | -1.07  (-1.28 to -0.87) |
| Kenya | 45885 (35487 to 55486) | 60974.46 (47156.32 to 73731.65) | 52143 (39439 to 66978) | 51407.65 (38883.17 to 66033.63) | 13.64  (7.48 to 26.86) | -0.52  (-0.59 to -0.46) |
| Kiribati | 37 (29 to 48) | 19211.68 (14693.76 to 24629.62) | 43 (32 to 56) | 18691.98 (14162.81 to 24338.35) | 14.67  (0.93 to 28.91) | -0.06  (-0.17 to 0.06) |
| Kyrgyzstan | 2905 (2188 to 3772) | 30537.43 (23004.63 to 39651.54) | 2923 (2221 to 3815) | 26459.4 (20104.64 to 34538.4) | 0.61  (-9.51 to 14.38) | -0.55  (-0.61 to -0.5) |
| Lao People's Democratic Republic | 2809 (2163 to 3530) | 22702.94 (17482.3 to 28525.5) | 2320 (1791 to 2982) | 17930.37 (13844.46 to 23047.82) | -17.43  (-27.08 to -6.06) | -0.96  (-1.09 to -0.83) |
| Lebanon | 2184 (1616 to 2814) | 26310.26 (19469.64 to 33904.3) | 1598 (1200 to 2082) | 19920.54 (14949.63 to 25953.28) | -26.81  (-35.77 to -16.04) | -0.77  (-0.93 to -0.61) |
| Lesotho | 2708 (2073 to 3395) | 59792.65 (45768.75 to 74959.44) | 1893 (1409 to 2478) | 54351.66 (40448.47 to 71158) | -30.1  (-38.15 to -22.29) | -0.3  (-0.32 to -0.28) |
| Liberia | 3112 (2352 to 4007) | 51899.46 (39229.73 to 66820.62) | 4626 (3431 to 5962) | 45705.69 (33901.52 to 58907.03) | 48.66  (32.32 to 67.53) | -0.5  (-0.55 to -0.45) |
| Libya | 3092 (2372 to 4017) | 27327.17 (20967.08 to 35496.94) | 1400 (1046 to 1800) | 22541.33 (16848.37 to 28981.53) | -54.73  (-59.92 to -47.89) | -0.74  (-0.81 to -0.67) |
| Madagascar | 25566 (19983 to 30480) | 64065.61 (50075.99 to 76381.81) | 33624 (25012 to 43739) | 52138.44 (38784.32 to 67823.56) | 31.52  (13.42 to 51.51) | -0.62  (-0.72 to -0.51) |
| Malawi | 22868 (17790 to 26999) | 65761.43 (51157.73 to 77638.78) | 20797 (15587 to 27119) | 50368.13 (37748.41 to 65677.64) | -9.06  (-22.28 to 8.33) | -0.94  (-1.02 to -0.86) |
| Malaysia | 7267 (5450 to 9504) | 19231.28 (14424.8 to 25152.2) | 6265 (4762 to 7965) | 15141.74 (11510.84 to 19251.09) | -13.79  (-24.77 to -1.39) | -0.66  (-0.75 to -0.57) |
| Maldives | 141 (107 to 179) | 21328.8 (16223.03 to 27095.75) | 101 (77 to 130) | 15782.9 (12066.22 to 20315.04) | -28.34  (-36.86 to -18.23) | -1.01  (-1.06 to -0.95) |
| Mali | 15588 (11864 to 20216) | 47450.01 (36113.38 to 61537.07) | 29395 (22150 to 38323) | 40765.39 (30717.48 to 53147.05) | 88.58  (67.5 to 108.67) | -0.56  (-0.6 to -0.52) |
| Marshall Islands | 33 (25 to 44) | 29008.25 (21689.12 to 38265.85) | 25 (18 to 33) | 26318.06 (19709.45 to 34873.52) | -26.13  (-35.01 to -15.44) | -0.29  (-0.38 to -0.2) |
| Mauritania | 3205 (2435 to 4180) | 48077.93 (36519.39 to 62705.44) | 3258 (2430 to 4224) | 39482.05 (29456.47 to 51197.47) | 1.64  (-10.62 to 15.75) | -0.71  (-0.77 to -0.65) |
| Mexico | 85646 (64170 to 110956) | 45621.74 (34181.84 to 59103.66) | 45603 (34439 to 58859) | 28620.75 (21613.74 to 36939.99) | -46.75  (-49.1 to -44.05) | -1.52  (-2.2 to -0.83) |
| Micronesia (Federated States of) | 29 (22 to 36) | 10853.24 (8424.41 to 13636.33) | 15 (11 to 19) | 9899.92 (7646.61 to 12754.39) | -48.65  (-53.96 to -42.14) | -0.26  (-0.48 to -0.03) |
| Mongolia | 1877 (1415 to 2415) | 34083.4 (25690.93 to 43838.59) | 1670 (1257 to 2139) | 27420.42 (20633.68 to 35112.54) | -11.02  (-21.15 to -0.94) | -0.74  (-0.94 to -0.54) |
| Montenegro | 117 (88 to 152) | 15403.52 (11632.04 to 19957.6) | 72 (54 to 93) | 14094.29 (10625.22 to 18230.47) | -38.53  (-46.06 to -30.01) | -0.39  (-0.43 to -0.36) |
| Morocco | 19180 (14459 to 25000) | 32264.87 (24322.59 to 42055.09) | 10816 (8118 to 14103) | 23693.69 (17783.94 to 30894.6) | -43.61  (-50.37 to -35.92) | -1.13  (-1.16 to -1.1) |
| Mozambique | 28452 (22163 to 33966) | 65168.02 (50764.7 to 77796.92) | 43603 (32875 to 56496) | 51594.03 (38900.51 to 66850.65) | 53.25  (32.43 to 77.9) | -0.81  (-0.88 to -0.74) |
| Myanmar | 19033 (14457 to 24083) | 19179.95 (14567.96 to 24268.76) | 12607 (9588 to 16107) | 15993.98 (12163.61 to 20433.98) | -33.76  (-41.72 to -25.09) | -0.68  (-0.73 to -0.63) |
| Namibia | 2152 (1660 to 2666) | 59019.21 (45532.5 to 73134.06) | 2334 (1758 to 3021) | 49360.09 (37185.87 to 63897.23) | 8.45  (-4.35 to 23.52) | -0.54  (-0.59 to -0.49) |
| Nepal | 22243 (16609 to 28684) | 36676.09 (27386.05 to 47295.97) | 11791 (8692 to 15592) | 25365.75 (18699.13 to 33543.82) | -46.99  (-53.59 to -39.78) | -1.39  (-1.44 to -1.34) |
| Nicaragua | 5216 (3894 to 6744) | 48031.8 (35853.78 to 62102.46) | 2957 (2217 to 3908) | 29678.72 (22251.32 to 39221.77) | -43.3  (-52.37 to -33.05) | -1.83  (-1.94 to -1.72) |
| Niger | 16086 (12354 to 20869) | 48399.46 (37169.99 to 62792.04) | 37079 (28158 to 48055) | 42932.07 (32602.72 to 55639.85) | 130.51  (104.89 to 158.92) | -0.37  (-0.43 to -0.32) |
| Nigeria | 114310 (88843 to 146149) | 37113.94 (28845.4 to 47451.14) | 185564 (141950 to 237276) | 32853.1 (25131.44 to 42008.5) | 62.33  (52.53 to 73.61) | -0.51  (-0.76 to -0.26) |
| North Macedonia | 462 (349 to 592) | 17372.73 (13114.29 to 22261.27) | 235 (176 to 309) | 13886.38 (10420 to 18233.07) | -49.07  (-54.29 to -41.64) | -0.9  (-0.97 to -0.83) |
| Pakistan | 167153 (125490 to 214484) | 46735.28 (35086.37 to 59969.01) | 195445 (147029 to 252699) | 39249.07 (29526.36 to 50746.89) | 16.93  (8.27 to 26.51) | -0.65  (-0.8 to -0.5) |
| Papua New Guinea | 2299 (1781 to 2938) | 20496.66 (15885.22 to 26196.73) | 5861 (4439 to 7681) | 23727.09 (17968.73 to 31093.37) | 155  (125.6 to 187.49) | 0.53  (0.34 to 0.72) |
| Paraguay | 1849 (1390 to 2372) | 19097.21 (14362.6 to 24505.62) | 1213 (904 to 1582) | 12633.7 (9416.29 to 16473.43) | -34.38  (-42.9 to -25.69) | -1.58  (-1.67 to -1.49) |
| Peru | 15401 (11394 to 20054) | 30832.5 (22810.48 to 40148.81) | 11802 (8908 to 15430) | 24302.09 (18341.53 to 31770.64) | -23.37  (-32.62 to -11.79) | -0.93  (-0.99 to -0.86) |
| Philippines | 27890 (22009 to 34531) | 17846.19 (14082.7 to 22095.31) | 27834 (21550 to 35438) | 13859.03 (10730.46 to 17645.32) | -0.2  (-8.87 to 8.16) | -0.67  (-0.77 to -0.57) |
| Republic of Moldova | 1103 (839 to 1407) | 18817.34 (14315.65 to 24009.76) | 393 (291 to 517) | 16042.85 (11877.02 to 21098.61) | -64.36  (-68.71 to -59.55) | -0.6  (-0.66 to -0.55) |
| Russian Federation | 21230 (16279 to 27261) | 14422.29 (11058.86 to 18519.09) | 18775 (14301 to 24366) | 14725.57 (11216.39 to 19110.95) | -11.57  (-13.89 to -9.12) | -1.15  (-1.62 to -0.68) |
| Rwanda | 15118 (11663 to 17979) | 63566.74 (49038.09 to 75596.54) | 13552 (10040 to 17828) | 50926.53 (37730.9 to 66995.75) | -10.36  (-21.71 to 5.35) | -0.79  (-0.89 to -0.69) |
| Saint Lucia | 68 (51 to 88) | 23719.05 (17866.14 to 30864.63) | 25 (19 to 32) | 18526.07 (14154.64 to 23967.47) | -63.16  (-67.4 to -57.95) | -0.87  (-0.94 to -0.81) |
| Saint Vincent and the Grenadines | 50 (38 to 66) | 25122.46 (18757.5 to 33057.32) | 22 (17 to 29) | 19499.7 (14668.21 to 25521.45) | -55.53  (-60.69 to -49.76) | -0.86  (-0.92 to -0.79) |
| Samoa | 40 (30 to 50) | 15232.39 (11701.03 to 19314.01) | 45 (35 to 57) | 15570.18 (12010.01 to 19590.67) | 14.45  (2.23 to 30.69) | 0.08  (-0.03 to 0.19) |
| Senegal | 14045 (10595 to 18355) | 54746.36 (41296.71 to 71543.9) | 15192 (11326 to 19334) | 43504.86 (32435.33 to 55368.06) | 8.16  (-3.84 to 21.54) | -0.8  (-0.83 to -0.78) |
| Serbia | 2081 (1552 to 2712) | 20608.35 (15369.1 to 26854.47) | 1208 (907 to 1583) | 19175.63 (14407.91 to 25129.6) | -41.98  (-48.31 to -35.22) | -0.29  (-0.39 to -0.18) |
| Sierra Leone | 5656 (4325 to 7324) | 48334.1 (36963.74 to 62587.98) | 8556 (6410 to 11072) | 40802.98 (30572.06 to 52805.25) | 51.28  (33.84 to 68.99) | -0.58  (-0.64 to -0.52) |
| Solomon Islands | 199 (153 to 257) | 18484.11 (14221.85 to 23860.78) | 287 (217 to 370) | 18156.21 (13730.44 to 23412.63) | 43.93  (27.99 to 61.15) | 0.02  (-0.06 to 0.1) |
| Somalia | 15520 (12385 to 17829) | 67391.74 (53779.85 to 77415.95) | 35815 (26960 to 45884) | 54870.95 (41304.87 to 70296.55) | 130.76  (93.82 to 172.85) | -0.59  (-0.66 to -0.52) |
| South Africa | 44160 (34015 to 53337) | 60210.23 (46377.76 to 72722.64) | 38426 (29048 to 49558) | 49323.04 (37285.39 to 63610.94) | -12.98  (-18.95 to -2.89) | -0.41  (-0.51 to -0.31) |
| South Sudan | 12024 (9401 to 14296) | 64166.24 (50170.21 to 76292.5) | 15085 (11408 to 19293) | 55676.12 (42105.39 to 71203.99) | 25.46  (10.28 to 44.66) | -0.41  (-0.47 to -0.35) |
| Sri Lanka | 3627 (2813 to 4598) | 13388.89 (10383.39 to 16971.99) | 2435 (1861 to 3162) | 10765.03 (8226.77 to 13980.89) | -32.87  (-41.2 to -24.29) | -0.79  (-0.91 to -0.68) |
| Sudan | 24900 (18436 to 31917) | 34269.45 (25373.49 to 43928.16) | 26487 (19384 to 34868) | 29892.21 (21875.83 to 39350.97) | 6.37  (-6.78 to 20.11) | -0.43  (-0.45 to -0.41) |
| Suriname | 170 (128 to 219) | 24834.03 (18701.24 to 31990.08) | 132 (100 to 172) | 19454.08 (14751.84 to 25384.98) | -22.27  (-31.35 to -12.56) | -0.87  (-0.9 to -0.83) |
| Syrian Arab Republic | 13703 (10300 to 17380) | 36067.77 (27111.03 to 45745.77) | 4652 (3493 to 6030) | 26242.69 (19707.68 to 34018.26) | -66.05  (-70.28 to -60.74) | -1.24  (-1.36 to -1.12) |
| Tajikistan | 5356 (4083 to 6938) | 34659.26 (26425 to 44898.92) | 6055 (4494 to 7886) | 31447.95 (23342.27 to 40957.42) | 13.06  (1.36 to 27.94) | -0.65  (-0.79 to -0.5) |
| Thailand | 8787 (7040 to 10802) | 10929.63 (8756.14 to 13435.62) | 5928 (4498 to 7502) | 13293.54 (10087 to 16823.95) | -32.54  (-40.96 to -21.71) | 1.39  (0.87 to 1.9) |
| Timor-Leste | 597 (455 to 762) | 22228.79 (16956.92 to 28406.97) | 514 (391 to 655) | 17725.9 (13471.18 to 22554.99) | -13.77  (-24.15 to -2.65) | -0.86  (-0.9 to -0.82) |
| Togo | 5762 (4341 to 7423) | 47167.76 (35536.72 to 60762.43) | 7109 (5307 to 9156) | 39996.6 (29855.86 to 51512.38) | 23.39  (11.1 to 39.57) | -0.56  (-0.62 to -0.49) |
| Tonga | 32 (24 to 42) | 16383.13 (12267.79 to 21117.53) | 29 (21 to 37) | 16081.78 (11872.32 to 20903.47) | -11.68  (-21.59 to 1.26) | -0.08  (-0.2 to 0.04) |
| Tunisia | 5727 (4222 to 7564) | 32022.41 (23607.54 to 42291.94) | 3000 (2269 to 3979) | 23601.8 (17852.31 to 31302.89) | -47.61  (-53.39 to -41.41) | -0.87  (-1.02 to -0.72) |
| Turkey | 28995 (21910 to 37407) | 23404.21 (17685.06 to 30193.9) | 12419 (9410 to 16112) | 16685.9 (12642.47 to 21647.76) | -57.17  (-63.14 to -50.74) | -1.45  (-1.78 to -1.12) |
| Turkmenistan | 3190 (2416 to 4153) | 32273.58 (24439.57 to 42008.22) | 2190 (1660 to 2809) | 25799.92 (19563.33 to 33091.01) | -31.36  (-38.98 to -22.98) | -0.88  (-0.99 to -0.77) |
| Tuvalu | 4 (3 to 5) | 17437.85 (13298.73 to 22391.79) | 3 (2 to 4) | 16764.15 (12905.54 to 21433.34) | -26.91  (-34.86 to -18.39) | -0.12  (-0.23 to -0.01) |
| Uganda | 45629 (35387 to 53665) | 66002.53 (51186.98 to 77626.99) | 64271 (48293 to 82355) | 54183.7 (40713.49 to 69429.24) | 40.86  (22.37 to 66.49) | -0.78  (-0.87 to -0.69) |
| Ukraine | 6352 (4801 to 8104) | 12668.49 (9574.12 to 16161.41) | 3544 (2713 to 4544) | 11937.72 (9137.66 to 15303.56) | -44.21  (-49.92 to -37.71) | 0.14  (-0.14 to 0.43) |
| United Republic of Tanzania | 58034 (44896 to 69364) | 63811.9 (49366.14 to 76269.93) | 79818 (59674 to 103865) | 51419.45 (38442.62 to 66910.94) | 37.54  (18.62 to 60.73) | -0.71  (-0.76 to -0.67) |
| Uzbekistan | 20149 (15149 to 25763) | 37376.11 (28102.24 to 47790.95) | 17252 (12927 to 22391) | 31804.21 (23829.9 to 41277.33) | -14.37  (-23.85 to -1.29) | -0.83  (-0.94 to -0.73) |
| Vanuatu | 82 (62 to 105) | 18935.33 (14468.44 to 24336.65) | 112 (84 to 147) | 19213.2 (14406.65 to 25196.82) | 37.43  (22.18 to 54.57) | 0.15  (0.05 to 0.25) |
| Venezuela (Bolivarian Republic of) | 15298 (11751 to 19590) | 35376.31 (27173.3 to 45302.13) | 9542 (7020 to 12410) | 26523.68 (19512.93 to 34494.29) | -37.63  (-45.06 to -28.9) | -0.53  (-0.77 to -0.3) |
| Viet Nam | 40675 (31103 to 52383) | 29472.13 (22536.68 to 37955.16) | 19674 (14776 to 25458) | 19378.85 (14554.03 to 25075.48) | -51.63  (-57.52 to -44.26) | -1.55  (-1.68 to -1.41) |
| Yemen | 15507 (11793 to 19955) | 30688.9 (23339.63 to 39492.67) | 17919 (13309 to 23327) | 25441.02 (18895.96 to 33119.47) | 15.55  (2.03 to 30.88) | -0.68  (-0.81 to -0.56) |
| Zambia | 18206 (14022 to 22010) | 63359.76 (48798.09 to 76595.77) | 23371 (17361 to 30966) | 50544.85 (37547.6 to 66970.85) | 28.37  (11.77 to 50.55) | -0.74  (-0.82 to -0.67) |
| Zimbabwe | 16162 (12295 to 20930) | 56042.01 (42633.91 to 72574.6) | 17579 (12983 to 22643) | 52541.85 (38803.66 to 67677.82) | 8.77  (-3.97 to 21.27) | -0.14  (-0.17 to -0.1) |

CI, confidence interval; EAPC, estimated annual percentage change; LMICs, low- and middle-income countries and territories; UI, uncertain interval.

## Supplementary Table S8. National burden and trends of incident cases and incidence rates (per 100,000 liveborn neonates) of bacterial skin diseases in 131 LMICs between 1990 and 2019.

| **Location** | **Incident cases in 1990**  **(95% UI)** | **Incident rate in 1990**  **( 95% UI)** | **Incident cases in 2019**  **(95% UI)** | **Incident rate in 2019**  **( 95% UI)** | **Percent change**  **(%, 95 CI)** | **EAPC**  **(%, 95 CI)** |
| --- | --- | --- | --- | --- | --- | --- |
| Global | 2081006 (1957718 to 2213508) | 20155.88 (18961.75 to 21439.24) | 2041223 (1922537 to 2171544) | 19976.05 (18814.55 to 21251.42) | -1.91  (-2.59 to -1.35) | -0.01  (-0.05 to 0.03) |
| Afghanistan | 6656 (6214 to 7135) | 17203.52 (16061.57 to 18442.29) | 19369 (18067 to 20665) | 17318.49 (16153.9 to 18477.47) | 191  (175.36 to 204.78) | 0.02  (0.01 to 0.02) |
| Albania | 1819 (1678 to 1968) | 27982.77 (25801.48 to 30273.56) | 744 (687 to 801) | 27931.83 (25767.97 to 30049.5) | -59.09  (-61.26 to -56.61) | 0  (-0.01 to 0.01) |
| Algeria | 9990 (9319 to 10704) | 17199.8 (16045.52 to 18429.19) | 11488 (10743 to 12355) | 17321.73 (16199.23 to 18630.3) | 14.99  (8.69 to 20.03) | 0.02  (0.02 to 0.02) |
| American Samoa | 30 (27 to 32) | 22802.19 (21158.38 to 24583.14) | 19 (17 to 20) | 23518.53 (21735.81 to 25440.51) | -36.25  (-39.73 to -32.7) | 0.11  (0.1 to 0.11) |
| Angola | 9725 (8969 to 10520) | 25664.23 (23667.08 to 27760.2) | 21816 (20221 to 23595) | 26166.18 (24252.87 to 28299.62) | 124.32  (112.67 to 136.37) | 0.1  (0.08 to 0.12) |
| Armenia | 783 (730 to 836) | 13629.59 (12703.87 to 14564.32) | 396 (365 to 426) | 13837.99 (12758.1 to 14907.27) | -49.44  (-52.03 to -46.72) | 0.07  (0.05 to 0.09) |
| Azerbaijan | 1950 (1810 to 2092) | 13629.95 (12655.25 to 14626.21) | 1507 (1394 to 1626) | 13868.79 (12829.03 to 14958.77) | -22.67  (-27.17 to -19.1) | 0.08  (0.06 to 0.1) |
| Bangladesh | 52798 (48809 to 57262) | 18037.23 (16674.37 to 19562.27) | 37416 (34631 to 40450) | 18416.61 (17045.57 to 19909.82) | -29.13  (-33.45 to -24.77) | 0.09  (0.08 to 0.09) |
| Belarus | 1986 (1847 to 2139) | 18523.53 (17219.33 to 19941.98) | 1470 (1362 to 1582) | 18705.55 (17327.41 to 20128.85) | -26.01  (-30.23 to -22.53) | 0.05  (0.03 to 0.06) |
| Belize | 67 (62 to 72) | 14984.16 (13967.9 to 16117.29) | 88 (81 to 94) | 15065.35 (13969.51 to 16131.61) | 31.23  (24.38 to 38.67) | 0.03  (0.02 to 0.04) |
| Benin | 4095 (3785 to 4421) | 23016.4 (21273.02 to 24845.48) | 8855 (8182 to 9526) | 23415.6 (21636.81 to 25190.64) | 116.24  (103.79 to 130.24) | 0.07  (0.07 to 0.07) |
| Bhutan | 297 (273 to 322) | 18042.5 (16617.5 to 19603.87) | 186 (171 to 202) | 18346.78 (16890.97 to 19901.13) | -37.29  (-40.97 to -33.44) | 0.08  (0.07 to 0.09) |
| Bolivia (Plurinational State of) | 3249 (3027 to 3508) | 17793.5 (16575.91 to 19206.98) | 4388 (4073 to 4737) | 17987.84 (16695.28 to 19416.64) | 35.04  (27.93 to 42.57) | 0.06  (0.05 to 0.07) |
| Bosnia and Herzegovina | 1436 (1338 to 1561) | 27769.55 (25884.87 to 30183.43) | 571 (531 to 614) | 27900.19 (25961.57 to 30041.93) | -60.25  (-62.91 to -57.85) | 0.03  (0.02 to 0.04) |
| Botswana | 1102 (1021 to 1194) | 35846.09 (33200.49 to 38828.94) | 1317 (1221 to 1422) | 36179.31 (33543.25 to 39061.67) | 19.52  (12.34 to 25.97) | 0.03  (0.03 to 0.04) |
| Brazil | 31102 (29182 to 33156) | 11983.42 (11243.73 to 12774.76) | 28484 (26763 to 30362) | 12122.06 (11389.56 to 12921.19) | -8.42  (-9.77 to -6.94) | 0.62  (0.46 to 0.78) |
| Bulgaria | 2085 (1931 to 2244) | 27788.08 (25737.46 to 29901.18) | 1278 (1185 to 1381) | 27927.81 (25891.16 to 30159.97) | -38.69  (-42.67 to -35.27) | 0.03  (0.02 to 0.04) |
| Burkina Faso | 7898 (7320 to 8528) | 23039.56 (21353.93 to 24879.65) | 16522 (15265 to 17871) | 23424.17 (21642.51 to 25337.09) | 109.2  (97.78 to 119.02) | 0.06  (0.06 to 0.07) |
| Burundi | 3133 (2894 to 3363) | 15835.4 (14628.43 to 16998.26) | 5658 (5262 to 6097) | 16131.34 (15003.14 to 17383.91) | 80.61  (68.8 to 92.71) | 0.07  (0.06 to 0.08) |
| Cabo Verde | 204 (190 to 220) | 22991.32 (21394.43 to 24687.42) | 191 (176 to 206) | 23424.89 (21632.16 to 25360.31) | -6.73  (-12.03 to -0.54) | 0.07  (0.06 to 0.07) |
| Cambodia | 5369 (4963 to 5821) | 16378.59 (15139.76 to 17757.89) | 4692 (4327 to 5093) | 16931.32 (15615.43 to 18378.83) | -12.62  (-17.22 to -7.98) | 0.12  (0.11 to 0.12) |
| Cameroon | 8009 (7425 to 8674) | 22985.23 (21308.61 to 24893.31) | 15924 (14749 to 17182) | 23425.65 (21697.32 to 25276.23) | 98.82  (87.88 to 109.79) | 0.08  (0.07 to 0.08) |
| Central African Republic | 2415 (2232 to 2612) | 25674.69 (23731.72 to 27766.71) | 3851 (3587 to 4155) | 26131.94 (24340.59 to 28193.22) | 59.46  (51.79 to 69.65) | 0.09  (0.08 to 0.1) |
| Chad | 5461 (5050 to 5901) | 23040.51 (21304.04 to 24896.75) | 13870 (12825 to 14940) | 23443.81 (21678.43 to 25252.93) | 153.96  (143.74 to 167.78) | 0.06  (0.05 to 0.07) |
| China | 525464 (493598 to 559417) | 28854.22 (27104.43 to 30718.65) | 328469 (308830 to 350789) | 28881.28 (27154.52 to 30843.82) | -37.49  (-38.67 to -36.56) | -0.02  (-0.04 to 0) |
| Colombia | 11155 (10393 to 12148) | 15814.38 (14733.78 to 17221.54) | 9533 (8885 to 10237) | 15830.54 (14753.3 to 16999.09) | -14.54  (-19.11 to -9.24) | 0  (-0.01 to 0) |
| Comoros | 233 (217 to 252) | 15843.93 (14762.49 to 17110.43) | 200 (186 to 215) | 16111.75 (14997.55 to 17258.98) | -14.08  (-18.61 to -9.03) | 0.07  (0.06 to 0.08) |
| Congo | 1930 (1799 to 2077) | 25708.68 (23962.57 to 27669.4) | 2841 (2622 to 3045) | 26157.27 (24146.19 to 28036.18) | 47.18  (37.46 to 55.35) | 0.1  (0.09 to 0.11) |
| Costa Rica | 974 (909 to 1047) | 15807.41 (14756.94 to 16992.46) | 803 (748 to 866) | 15866.93 (14778.94 to 17107.98) | -17.54  (-21.87 to -13) | 0  (0 to 0.01) |
| Côte d'Ivoire | 9803 (9107 to 10527) | 23007.64 (21374.97 to 24706.67) | 15550 (14342 to 16809) | 23473.06 (21650.24 to 25374.86) | 58.62  (50.41 to 68.14) | 0.07  (0.06 to 0.08) |
| Cuba | 2066 (1916 to 2236) | 14972.13 (13888.88 to 16207.22) | 1203 (1108 to 1299) | 15068.64 (13884.17 to 16274.2) | -41.77  (-44.96 to -38.22) | 0.04  (0.04 to 0.05) |
| Democratic People's Republic of Korea | 16898 (15734 to 18315) | 31152.01 (29005.87 to 33764.03) | 8392 (7753 to 9017) | 32122.15 (29677.37 to 34516.84) | -50.34  (-53.81 to -47.24) | 0.13  (0.12 to 0.14) |
| Democratic Republic of the Congo | 35985 (33337 to 38893) | 25690.47 (23799.92 to 27766.09) | 57531 (53233 to 61945) | 26145.4 (24192.3 to 28151.77) | 59.87  (50.77 to 69.69) | 0.09  (0.08 to 0.1) |
| Djibouti | 262 (244 to 284) | 15823.49 (14710.75 to 17121.11) | 420 (389 to 450) | 16092.9 (14918.25 to 17264.26) | 59.94  (50.13 to 70.36) | 0.08  (0.07 to 0.08) |
| Dominica | 19 (18 to 21) | 15037.13 (13928.29 to 16210.35) | 10 (9 to 10) | 15150.41 (13983.23 to 16297.45) | -50.97  (-54.19 to -48.35) | 0.06  (0.05 to 0.07) |
| Dominican Republic | 2703 (2509 to 2916) | 15005.36 (13925.66 to 16186.08) | 2594 (2408 to 2794) | 15028.01 (13949.77 to 16182.77) | -4.03  (-9.51 to 1.34) | 0.03  (0.03 to 0.04) |
| Ecuador | 4010 (3740 to 4337) | 17702.64 (16508.53 to 19147.86) | 4974 (4601 to 5349) | 18729.73 (17325.2 to 20142.34) | 24.04  (16.76 to 31.92) | 0.43  (0.32 to 0.53) |
| Egypt | 27268 (25497 to 28972) | 19193.54 (17947.38 to 20393.36) | 30791 (28974 to 32754) | 19123.59 (17995.32 to 20343.24) | 12.92  (7.51 to 19.03) | -0.03  (-0.04 to -0.02) |
| El Salvador | 1954 (1820 to 2101) | 15774.35 (14694.69 to 16961.48) | 1353 (1253 to 1447) | 15810.44 (14648.58 to 16917.95) | -30.77  (-34.78 to -26.42) | 0  (-0.01 to 0) |
| Equatorial Guinea | 438 (406 to 474) | 25641.6 (23741.21 to 27714.19) | 764 (707 to 824) | 26149.11 (24203.72 to 28205.51) | 74.18  (64.09 to 86.8) | 0.1  (0.09 to 0.11) |
| Eritrea | 1475 (1373 to 1594) | 15812.88 (14723.71 to 17095.3) | 2421 (2237 to 2620) | 16128.25 (14899.73 to 17449.2) | 64.22  (55.82 to 73.49) | 0.08  (0.07 to 0.09) |
| Eswatini | 863 (803 to 933) | 35933.92 (33462.11 to 38845.6) | 804 (742 to 866) | 36203.7 (33417.6 to 38997.01) | -6.85  (-12.27 to -1.69) | 0.03  (0.02 to 0.03) |
| Ethiopia | 31481 (29472 to 33356) | 17092.83 (16002.05 to 18110.86) | 48088 (44985 to 51270) | 17484.85 (16356.56 to 18642.04) | 52.75  (48.86 to 56.99) | 0.1  (0.09 to 0.11) |
| Fiji | 327 (304 to 354) | 22716.32 (21130.15 to 24567.45) | 311 (286 to 336) | 23416 (21484.05 to 25242.29) | -4.85  (-11.33 to 0.89) | 0.11  (0.1 to 0.11) |
| Gabon | 760 (701 to 820) | 25722.41 (23736.15 to 27774.15) | 843 (783 to 914) | 26166.72 (24296.22 to 28368.36) | 10.97  (1.49 to 20.27) | 0.09  (0.07 to 0.1) |
| Gambia | 828 (767 to 891) | 22987.78 (21287.69 to 24741.68) | 1255 (1165 to 1354) | 23391.75 (21716.49 to 25231.29) | 51.54  (43.97 to 59.28) | 0.07  (0.06 to 0.08) |
| Georgia | 860 (801 to 920) | 13607.03 (12676.08 to 14564.11) | 476 (443 to 514) | 13714.88 (12744.73 to 14813.65) | -44.62  (-47.35 to -40.75) | 0.04  (0.03 to 0.05) |
| Ghana | 10404 (9650 to 11233) | 23020.99 (21353.11 to 24854.73) | 15067 (13915 to 16222) | 23454.83 (21661.63 to 25253.38) | 44.82  (35.03 to 53.03) | 0.08  (0.07 to 0.08) |
| Grenada | 23 (21 to 24) | 14993.99 (13855.5 to 16021.98) | 16 (15 to 17) | 15111.65 (14019.39 to 16282.39) | -29.31  (-33.27 to -24.61) | 0.05  (0.03 to 0.06) |
| Guatemala | 4020 (3737 to 4343) | 15825.93 (14711.73 to 17097.1) | 4954 (4587 to 5336) | 15831.61 (14657.37 to 17053.35) | 23.23  (15.55 to 30.29) | 0.01  (0.01 to 0.01) |
| Guinea | 5394 (4990 to 5825) | 23024.07 (21301.01 to 24864.6) | 8418 (7813 to 9052) | 23407.29 (21724.71 to 25170.59) | 56.07  (46.53 to 67.2) | 0.06  (0.06 to 0.07) |
| Guinea-Bissau | 797 (739 to 855) | 23007.14 (21345.95 to 24689.36) | 1096 (1014 to 1182) | 23420.71 (21660.8 to 25262.85) | 37.62  (29.88 to 44.92) | 0.06  (0.06 to 0.07) |
| Guyana | 250 (232 to 268) | 14968.89 (13870.42 to 16046.59) | 163 (152 to 175) | 15056.45 (14039.41 to 16158.34) | -34.7  (-38.02 to -31.02) | 0.04  (0.04 to 0.05) |
| Haiti | 2772 (2573 to 2969) | 15006.6 (13933.55 to 16073.81) | 3758 (3456 to 4045) | 15119.33 (13902.15 to 16272.18) | 35.6  (27.41 to 44.29) | 0.04  (0.03 to 0.05) |
| Honduras | 2141 (1988 to 2304) | 15788.56 (14657.14 to 16989.68) | 2816 (2620 to 3044) | 15790.91 (14693.67 to 17070.92) | 31.51  (24.47 to 39.77) | -0.01  (-0.01 to 0) |
| India | 350813 (329595 to 376120) | 17868.29 (16787.54 to 19157.24) | 320679 (300535 to 344600) | 18090.41 (16954.03 to 19439.89) | -8.59  (-9.99 to -7.23) | 0.05  (0.03 to 0.07) |
| Indonesia | 23077 (21531 to 24865) | 6432.15 (6001.17 to 6930.7) | 19410 (18087 to 20886) | 6664.29 (6209.94 to 7170.92) | -15.89  (-17.25 to -14.3) | 0.12  (0.11 to 0.13) |
| Iran (Islamic Republic of) | 23257 (21988 to 24737) | 17295.91 (16352.43 to 18396.84) | 17826 (16803 to 18986) | 17382.15 (16384.69 to 18513.71) | -23.35  (-24.32 to -22.17) | 0.01  (0.01 to 0.01) |
| Iraq | 8595 (7978 to 9244) | 16265.83 (15097.84 to 17494.3) | 11895 (11153 to 12757) | 16350.57 (15330.61 to 17535.15) | 38.4  (31.7 to 45.47) | 0.02  (0.01 to 0.02) |
| Jamaica | 636 (593 to 679) | 14965.88 (13957.54 to 15984.66) | 412 (382 to 443) | 15058.27 (13943.35 to 16194.82) | -35.13  (-38.41 to -31.69) | 0.04  (0.03 to 0.04) |
| Jordan | 1785 (1675 to 1914) | 17297.88 (16227.71 to 18539.29) | 3245 (3026 to 3478) | 17365.61 (16192.56 to 18611.65) | 81.76  (70.49 to 90.41) | 0.02  (0.01 to 0.02) |
| Kazakhstan | 3663 (3378 to 3926) | 13598.23 (12542.87 to 14578.18) | 3701 (3427 to 3971) | 13772.4 (12750.83 to 14776.83) | 1.05  (-4.18 to 6.54) | 0.07  (0.04 to 0.09) |
| Kenya | 12073 (11283 to 12877) | 16043.44 (14993.3 to 17111.16) | 16528 (15515 to 17626) | 16295.32 (15295.92 to 17377.95) | 36.9  (35.27 to 38.5) | 0.03  (0.02 to 0.04) |
| Kiribati | 44 (41 to 48) | 22730.12 (21125.82 to 24526.67) | 53 (49 to 58) | 23383.15 (21526.67 to 25308.83) | 21.24  (15.34 to 28.54) | 0.1  (0.09 to 0.11) |
| Kyrgyzstan | 1291 (1191 to 1387) | 13570 (12523.08 to 14584.14) | 1523 (1420 to 1643) | 13790.55 (12850.83 to 14875.74) | 18  (12.42 to 25.53) | 0.07  (0.04 to 0.09) |
| Lao People's Democratic Republic | 2023 (1873 to 2185) | 16351.8 (15136.64 to 17659.05) | 2193 (2027 to 2379) | 16954.91 (15668.58 to 18386.38) | 8.41  (3.4 to 15.62) | 0.13  (0.12 to 0.13) |
| Lebanon | 1430 (1336 to 1525) | 17225.68 (16094.71 to 18375.83) | 1388 (1298 to 1482) | 17303.47 (16174.89 to 18473.7) | -2.9  (-8.44 to 1.99) | 0.02  (0.01 to 0.02) |
| Lesotho | 1623 (1504 to 1752) | 35839.01 (33212.54 to 38686.46) | 1262 (1173 to 1363) | 36241.21 (33670.93 to 39134.01) | -22.24  (-26.73 to -17.44) | 0.04  (0.04 to 0.05) |
| Liberia | 1381 (1279 to 1484) | 23033.45 (21335.2 to 24757.56) | 2370 (2198 to 2557) | 23417.55 (21714.32 to 25266.55) | 71.62  (62.36 to 79.49) | 0.07  (0.06 to 0.08) |
| Libya | 1948 (1824 to 2089) | 17219.77 (16122.97 to 18463.72) | 1076 (1006 to 1162) | 17319.46 (16202.12 to 18710.34) | -44.8  (-47.46 to -41.06) | 0.02  (0.01 to 0.02) |
| Madagascar | 6301 (5856 to 6808) | 15790.2 (14675.16 to 17059.34) | 10373 (9660 to 11224) | 16084.77 (14979.72 to 17404.98) | 64.62  (55.73 to 73.99) | 0.07  (0.06 to 0.08) |
| Malawi | 5484 (5107 to 5933) | 15770.46 (14687.37 to 17061.3) | 6655 (6204 to 7112) | 16116.42 (15024.28 to 17224.22) | 21.34  (15.67 to 29.14) | 0.08  (0.07 to 0.08) |
| Malaysia | 6179 (5725 to 6746) | 16352.33 (15151.1 to 17854.6) | 6994 (6477 to 7553) | 16905.13 (15654.78 to 18255.5) | 13.2  (6.71 to 19.55) | 0.12  (0.11 to 0.13) |
| Maldives | 108 (100 to 117) | 16374.67 (15126.8 to 17672.29) | 108 (100 to 117) | 16915.93 (15629.5 to 18246.72) | 0.04  (-5.18 to 4.45) | 0.11  (0.1 to 0.12) |
| Mali | 7560 (7023 to 8122) | 23014.13 (21376.81 to 24724.12) | 16891 (15625 to 18119) | 23424.85 (21668.82 to 25127.41) | 123.42  (112.83 to 133.79) | 0.07  (0.07 to 0.08) |
| Marshall Islands | 26 (24 to 28) | 22806.57 (21080.83 to 24605.1) | 22 (20 to 24) | 23465 (21645.01 to 25431.19) | -16.22  (-20.87 to -11.24) | 0.09  (0.08 to 0.1) |
| Mauritania | 1534 (1426 to 1649) | 23014.49 (21390.73 to 24740.39) | 1933 (1798 to 2087) | 23423.26 (21792.39 to 25291.57) | 25.96  (18.56 to 32.84) | 0.07  (0.07 to 0.08) |
| Mexico | 31095 (29143 to 33188) | 16563.71 (15523.64 to 17678.43) | 25058 (23510 to 26821) | 15726.14 (14754.75 to 16832.6) | -19.42  (-20.62 to -18.27) | -0.17  (-0.21 to -0.14) |
| Micronesia (Federated States of) | 60 (56 to 64) | 22710.72 (21118.73 to 24469.56) | 35 (32 to 37) | 23392.3 (21728.48 to 25206.54) | -42.02  (-45.18 to -38.65) | 0.09  (0.09 to 0.1) |
| Mongolia | 752 (699 to 808) | 13650.22 (12695.84 to 14677.22) | 841 (781 to 905) | 13804.71 (12819.45 to 14855.36) | 11.86  (4.95 to 17.48) | 0.06  (0.04 to 0.08) |
| Montenegro | 212 (197 to 229) | 27811.37 (25908.57 to 30139.84) | 143 (133 to 154) | 27918.83 (25978.25 to 30199.72) | -32.56  (-36.44 to -28.45) | 0.04  (0.03 to 0.05) |
| Morocco | 10237 (9558 to 10940) | 17221.26 (16077.64 to 18403.47) | 7909 (7377 to 8536) | 17326.43 (16160.85 to 18699.14) | -22.74  (-26.64 to -18.23) | 0.01  (0.01 to 0.02) |
| Mozambique | 6907 (6447 to 7437) | 15820.6 (14767.25 to 17035.01) | 13633 (12664 to 14689) | 16131.82 (14984.73 to 17381.32) | 97.38  (86.03 to 109.59) | 0.07  (0.07 to 0.08) |
| Myanmar | 16251 (15075 to 17633) | 16376.54 (15190.75 to 17769.11) | 13364 (12374 to 14442) | 16954.75 (15699.1 to 18321.8) | -17.77  (-21.83 to -12.95) | 0.13  (0.12 to 0.14) |
| Namibia | 1307 (1204 to 1409) | 35848.16 (33025.79 to 38645.81) | 1710 (1588 to 1844) | 36159.92 (33587.34 to 39001.84) | 30.8  (23.69 to 39.25) | 0.04  (0.03 to 0.04) |
| Nepal | 11232 (10375 to 12171) | 18520.66 (17107.07 to 20068.28) | 8602 (7959 to 9277) | 18506.16 (17121.46 to 19957.34) | -23.42  (-28.17 to -19.33) | -0.02  (-0.04 to -0.01) |
| Nicaragua | 1718 (1597 to 1839) | 15816.54 (14705.6 to 16937.5) | 1576 (1469 to 1698) | 15819 (14743.46 to 17036.5) | -8.23  (-13.9 to -3.08) | 0  (0 to 0.01) |
| Niger | 7645 (7124 to 8220) | 23002.12 (21435.26 to 24732.85) | 20215 (18793 to 21846) | 23405.87 (21759.3 to 25294.53) | 164.43  (150.63 to 177.18) | 0.07  (0.06 to 0.07) |
| Nigeria | 72182 (67312 to 77138) | 23436.01 (21854.84 to 25045.07) | 134869 (126116 to 144363) | 23877.91 (22328.21 to 25558.68) | 86.85  (84.83 to 88.94) | 0.07  (0.06 to 0.07) |
| North Macedonia | 739 (684 to 799) | 27799.99 (25720.64 to 30056.3) | 472 (439 to 510) | 27889.18 (25938.33 to 30137.08) | -36.08  (-39.56 to -32.37) | 0.03  (0.02 to 0.04) |
| Pakistan | 65102 (60206 to 69932) | 18202.18 (16833.44 to 19552.65) | 92240 (85825 to 99224) | 18523.6 (17235.3 to 19926.06) | 41.69  (37.81 to 46.2) | 0.09  (0.08 to 0.1) |
| Papua New Guinea | 2552 (2336 to 2753) | 22755.75 (20829.48 to 24549.15) | 5792 (5324 to 6279) | 23444.76 (21552.12 to 25418.83) | 126.96  (115.43 to 138.93) | 0.1  (0.09 to 0.11) |
| Paraguay | 1116 (1029 to 1204) | 11529.67 (10624.51 to 12440.19) | 1109 (1031 to 1190) | 11551.93 (10732.1 to 12389.11) | -0.62  (-5.35 to 5.58) | 0.01  (0.01 to 0.02) |
| Peru | 8915 (8247 to 9689) | 17848.06 (16510.11 to 19398.18) | 8724 (8105 to 9424) | 17963.98 (16689.54 to 19405.24) | -2.14  (-8.28 to 3.38) | 0.05  (0.04 to 0.06) |
| Philippines | 34122 (31957 to 36527) | 21834.06 (20448.56 to 23372.66) | 45019 (42254 to 48100) | 22416.31 (21039.08 to 23950.34) | 31.93  (30.7 to 33.1) | 0.06  (0.04 to 0.08) |
| Republic of Moldova | 1085 (1004 to 1166) | 18508.62 (17128.78 to 19892.58) | 459 (424 to 491) | 18716.11 (17306.85 to 20060.27) | -57.73  (-59.83 to -55.43) | 0.04  (0.03 to 0.06) |
| Russian Federation | 27214 (25610 to 29109) | 18487.39 (17397.88 to 19774.56) | 23795 (22410 to 25467) | 18663.09 (17576.49 to 19974.23) | -12.56  (-13.26 to -11.64) | 0.04  (0.03 to 0.06) |
| Rwanda | 3759 (3505 to 4040) | 15807.28 (14735.58 to 16988.18) | 4289 (3981 to 4623) | 16119.28 (14960.32 to 17373.6) | 14.1  (8.34 to 19.89) | 0.07  (0.06 to 0.08) |
| Saint Lucia | 43 (40 to 46) | 14978.33 (13838.16 to 16085) | 20 (19 to 22) | 15132.44 (14032.15 to 16319.91) | -52.35  (-54.72 to -49.56) | 0.05  (0.05 to 0.06) |
| Saint Vincent and the Grenadines | 30 (28 to 32) | 14990.89 (13855.46 to 16114.97) | 17 (16 to 19) | 15142.73 (14012.85 to 16267.31) | -42.13  (-45.91 to -38.22) | 0.05  (0.05 to 0.06) |
| Samoa | 59 (54 to 64) | 22735.39 (20894.46 to 24697.74) | 68 (63 to 74) | 23414.75 (21751.46 to 25280.74) | 15.32  (8.31 to 21.66) | 0.1  (0.09 to 0.11) |
| Senegal | 5904 (5495 to 6357) | 23013.73 (21417.38 to 24777.7) | 8181 (7550 to 8768) | 23427.76 (21620.49 to 25109.96) | 38.56  (31.11 to 46.21) | 0.07  (0.07 to 0.08) |
| Serbia | 2462 (2275 to 2670) | 24375.37 (22524.6 to 26439.56) | 1557 (1441 to 1694) | 24725.66 (22887.41 to 26892.9) | -36.75  (-39.87 to -32.38) | 0.15  (0.12 to 0.18) |
| Sierra Leone | 2695 (2488 to 2881) | 23030.89 (21259.48 to 24624.11) | 4906 (4557 to 5252) | 23398.33 (21732.32 to 25046.39) | 82.06  (71.36 to 92.47) | 0.07  (0.06 to 0.08) |
| Solomon Islands | 245 (227 to 265) | 22746.75 (21035.42 to 24631.04) | 370 (343 to 403) | 23416.58 (21716.32 to 25504.31) | 50.84  (43.43 to 60.27) | 0.09  (0.09 to 0.1) |
| Somalia | 3639 (3370 to 3945) | 15802.63 (14631.13 to 17130.32) | 10481 (9712 to 11277) | 16056.98 (14878.95 to 17277.64) | 187.98  (174.59 to 204.73) | 0.07  (0.06 to 0.07) |
| South Africa | 26743 (24953 to 28558) | 36463.16 (34022.64 to 38938.44) | 28517 (26749 to 30443) | 36603.69 (34334.49 to 39076.23) | 6.63  (4.04 to 9.23) | 0.01  (0 to 0.01) |
| South Sudan | 2962 (2743 to 3179) | 15807.4 (14638.34 to 16966.17) | 4368 (4076 to 4718) | 16121.66 (15044.46 to 17413.22) | 47.47  (39.82 to 55.77) | 0.07  (0.07 to 0.08) |
| Sri Lanka | 4422 (4113 to 4775) | 16322.18 (15182.25 to 17624.24) | 3820 (3540 to 4134) | 16886.86 (15652.65 to 18277.67) | -13.62  (-18.42 to -8.7) | 0.12  (0.11 to 0.12) |
| Sudan | 12517 (11689 to 13397) | 17227.21 (16087.81 to 18437.75) | 15329 (14322 to 16479) | 17299.7 (16163.14 to 18597.25) | 22.46  (16.33 to 28.85) | 0.01  (0.01 to 0.01) |
| Suriname | 103 (96 to 111) | 15020.77 (13980.41 to 16154.16) | 103 (95 to 111) | 15148.67 (13948.15 to 16305.53) | 0.07  (-5.73 to 7.37) | 0.05  (0.03 to 0.06) |
| Syrian Arab Republic | 6545 (6093 to 7007) | 17227.87 (16037.02 to 18443.91) | 3070 (2881 to 3293) | 17321.44 (16255 to 18577.8) | -53.09  (-55.5 to -50.52) | 0.01  (0.01 to 0.02) |
| Tajikistan | 2098 (1934 to 2263) | 13576.03 (12514.62 to 14644.11) | 2649 (2458 to 2838) | 13757.3 (12767.16 to 14738.23) | 26.26  (18.71 to 33.52) | 0.06  (0.04 to 0.08) |
| Thailand | 13142 (12180 to 14177) | 16345.29 (15148.84 to 17633.3) | 7538 (6962 to 8151) | 16903.44 (15613.1 to 18279.44) | -42.64  (-46.15 to -39.13) | 0.12  (0.11 to 0.13) |
| Timor-Leste | 440 (407 to 475) | 16391.25 (15170.84 to 17709.5) | 491 (455 to 531) | 16921.05 (15694.86 to 18301.36) | 11.63  (4.93 to 17.37) | 0.11  (0.11 to 0.12) |
| Togo | 2810 (2603 to 3025) | 22999.43 (21306.82 to 24762.44) | 4164 (3871 to 4481) | 23423.79 (21775.97 to 25210.2) | 48.19  (40.59 to 56.81) | 0.07  (0.06 to 0.08) |
| Tonga | 45 (41 to 49) | 22701.22 (20956.1 to 24613.82) | 42 (39 to 45) | 23463.62 (21710.57 to 25239.12) | -7  (-12.81 to -1.94) | 0.11  (0.1 to 0.12) |
| Tunisia | 3080 (2885 to 3295) | 17223.55 (16127.91 to 18425.32) | 2206 (2054 to 2372) | 17353.46 (16154 to 18661.76) | -28.39  (-31.79 to -24.59) | 0.02  (0.02 to 0.02) |
| Turkey | 21293 (19851 to 22730) | 17187.39 (16022.88 to 18347.05) | 12837 (12014 to 13757) | 17246.83 (16140.79 to 18482.99) | -39.71  (-42.62 to -36.6) | 0  (0 to 0.01) |
| Turkmenistan | 1343 (1236 to 1449) | 13588.4 (12506.92 to 14653.21) | 1167 (1084 to 1258) | 13744.57 (12766.82 to 14821.11) | -13.15  (-18.39 to -8.38) | 0.07  (0.04 to 0.09) |
| Tuvalu | 5 (5 to 5) | 23043.37 (21313.82 to 24971.73) | 4 (4 to 4) | 23735.15 (22015.82 to 25595.77) | -21.7  (-26.01 to -17.17) | 0.1  (0.09 to 0.11) |
| Uganda | 10924 (10202 to 11735) | 15801.24 (14756.58 to 16974.91) | 19085 (17708 to 20493) | 16089.4 (14928.43 to 17276.64) | 74.71  (64.39 to 84.05) | 0.07  (0.06 to 0.08) |
| Ukraine | 9257 (8564 to 10018) | 18460.94 (17078.25 to 19977.96) | 5539 (5169 to 5989) | 18657.21 (17409.19 to 20173.2) | -40.16  (-42.99 to -37.18) | 0.05  (0.03 to 0.06) |
| United Republic of Tanzania | 14385 (13337 to 15488) | 15816.7 (14665.36 to 17029.59) | 25050 (23304 to 26927) | 16137.53 (15012.36 to 17346.84) | 74.15  (66.34 to 83.85) | 0.07  (0.06 to 0.08) |
| Uzbekistan | 7312 (6796 to 7860) | 13564.48 (12606.19 to 14580.61) | 7482 (6914 to 8076) | 13793.23 (12745.53 to 14887.33) | 2.32  (-2.99 to 7.63) | 0.07  (0.05 to 0.09) |
| Vanuatu | 98 (91 to 106) | 22750.19 (21053.05 to 24574.3) | 137 (126 to 147) | 23412.14 (21645.88 to 25247.76) | 39.38  (29.99 to 47.28) | 0.1  (0.1 to 0.11) |
| Venezuela (Bolivarian Republic of) | 6826 (6338 to 7354) | 15785.34 (14656.63 to 17006.45) | 5699 (5298 to 6125) | 15841.41 (14726.44 to 17025.57) | -16.51  (-20.62 to -12.53) | 0.01  (0 to 0.01) |
| Viet Nam | 40459 (37419 to 43824) | 29315.57 (27112.9 to 31753.79) | 30299 (27917 to 32802) | 29844.07 (27497.79 to 32309.75) | -25.11  (-29.97 to -20.12) | 0.07  (0.05 to 0.08) |
| Yemen | 8693 (8123 to 9356) | 17203.47 (16076.28 to 18515.19) | 12174 (11358 to 13064) | 17284.65 (16126.05 to 18548.1) | 40.05  (33.1 to 47.43) | 0.02  (0.02 to 0.02) |
| Zambia | 4543 (4233 to 4904) | 15808.88 (14731.19 to 17065.05) | 7453 (6924 to 8020) | 16119.06 (14974.53 to 17346.3) | 64.07  (54.37 to 72.3) | 0.07  (0.06 to 0.09) |
| Zimbabwe | 10358 (9650 to 11163) | 35916.43 (33460.34 to 38706.41) | 12120 (11236 to 13085) | 36224.17 (33582.93 to 39111.18) | 17.01  (10.38 to 23.57) | 0.03  (0.03 to 0.04) |

CI, confidence interval; EAPC, estimated annual percentage change; LMICs, low- and middle-income countries and territories; UI, uncertain interval.

## Supplementary Table S9. National burden and trends of incident cases and incidence rates (per 100,000 liveborn neonates) of encephalitis in 131 LMICs between 1990 and 2019.

| **Location** | **Incident cases in 1990**  **(95% UI)** | **Incident rate in 1990**  **( 95% UI)** | **Incident cases in 2019**  **(95% UI)** | **Incident rate in 2019**  **( 95% UI)** | **Percent change**  **(%, 95 CI)** | **EAPC**  **(%, 95 CI)** |
| --- | --- | --- | --- | --- | --- | --- |
| Global | 13976 (11081 to 17182) | 135.36 (107.33 to 166.41) | 9642 (7594 to 11985) | 94.36 (74.32 to 117.29) | -31.01  (-32.61 to -29.66) | -1.41  (-1.49 to -1.33) |
| Afghanistan | 34 (26 to 44) | 88.69 (67.11 to 113.41) | 95 (72 to 119) | 84.54 (64.25 to 106.03) | 175.54  (142.58 to 208.94) | -0.26  (-0.55 to 0.04) |
| Albania | 5 (4 to 6) | 75.09 (60.32 to 92.35) | 1 (1 to 1) | 40.32 (32.04 to 49.24) | -77.99  (-80.31 to -75.25) | -2.88  (-3.15 to -2.6) |
| Algeria | 30 (22 to 38) | 50.85 (38.58 to 65.08) | 31 (23 to 39) | 46.26 (35.42 to 59.1) | 3.87  (-8.21 to 17.53) | -0.37  (-0.46 to -0.28) |
| American Samoa | 0 (0 to 0) | 55.01 (42.01 to 71.17) | 0 (0 to 0) | 55.61 (42.08 to 71.34) | -37.52  (-45.13 to -28.18) | -0.07  (-0.26 to 0.11) |
| Angola | 19 (14 to 24) | 49.02 (36.85 to 62.43) | 32 (24 to 42) | 38.84 (29.07 to 50.24) | 74.31  (52.51 to 94.59) | -1.05  (-1.13 to -0.97) |
| Armenia | 3 (3 to 4) | 58.39 (45.81 to 72.7) | 2 (1 to 2) | 53.68 (42.21 to 66.59) | -54.22  (-59.58 to -47.93) | -0.28  (-0.42 to -0.14) |
| Azerbaijan | 22 (17 to 28) | 153.32 (116.94 to 193.56) | 16 (12 to 20) | 146.3 (109.94 to 183.79) | -27.49  (-34.31 to -19.42) | -0.31  (-0.64 to 0.01) |
| Bangladesh | 297 (220 to 391) | 101.48 (75.06 to 133.64) | 215 (158 to 287) | 106.03 (77.68 to 141.33) | -27.48  (-35.67 to -18.13) | 0.15  (-0.34 to 0.64) |
| Belarus | 10 (8 to 12) | 92.87 (74.48 to 113.69) | 6 (5 to 8) | 81.45 (64.61 to 99.9) | -35.74  (-43 to -27.87) | -0.7  (-0.96 to -0.43) |
| Belize | 0 (0 to 1) | 96.78 (79.83 to 116.37) | 0 (0 to 0) | 64.37 (52.22 to 77.64) | -13.19  (-23.82 to -3.16) | -1.44  (-1.61 to -1.27) |
| Benin | 10 (7 to 13) | 54.38 (41.89 to 70.29) | 19 (14 to 24) | 49.73 (37.81 to 64.05) | 94.38  (73 to 116.22) | -0.37  (-0.4 to -0.34) |
| Bhutan | 4 (3 to 5) | 257.31 (194.05 to 328.77) | 2 (1 to 3) | 195.13 (144.76 to 250.18) | -53.23  (-58.61 to -46.67) | -1.25  (-1.34 to -1.16) |
| Bolivia (Plurinational State of) | 21 (16 to 26) | 114.43 (88.03 to 143.2) | 21 (17 to 27) | 87.07 (68.16 to 108.8) | 1.65  (-9.21 to 13.07) | -1.19  (-1.29 to -1.09) |
| Bosnia and Herzegovina | 1 (1 to 2) | 27.36 (21.31 to 34.25) | 0 (0 to 1) | 18.63 (13.69 to 24.61) | -73.06  (-77.22 to -68.46) | -1.74  (-1.98 to -1.5) |
| Botswana | 1 (1 to 2) | 39.8 (29.75 to 51.79) | 1 (1 to 2) | 39.13 (29.08 to 50.56) | 16.41  (3.36 to 30.86) | -0.1  (-0.11 to -0.08) |
| Brazil | 140 (112 to 174) | 54.05 (43.25 to 67.19) | 79 (63 to 98) | 33.6 (26.63 to 41.7) | -43.72  (-46.8 to -40.73) | -1.92  (-2.13 to -1.71) |
| Bulgaria | 4 (3 to 5) | 49.78 (41.1 to 61.06) | 2 (2 to 2) | 41.33 (33.86 to 49.99) | -49.35  (-54.59 to -43.35) | -0.74  (-0.97 to -0.5) |
| Burkina Faso | 21 (16 to 27) | 61.43 (46.92 to 78.49) | 39 (29 to 50) | 54.67 (40.76 to 70.61) | 83.11  (60.52 to 109.63) | -0.59  (-0.65 to -0.52) |
| Burundi | 13 (10 to 17) | 65.74 (50.76 to 83.89) | 23 (17 to 28) | 64.25 (49.88 to 80.61) | 73.28  (53.97 to 96.03) | -0.17  (-0.21 to -0.12) |
| Cabo Verde | 0 (0 to 1) | 51.61 (38.09 to 67.36) | 0 (0 to 0) | 44.81 (33.16 to 58.16) | -20.51  (-30.04 to -9.96) | -0.52  (-0.64 to -0.41) |
| Cambodia | 20 (15 to 25) | 60.46 (45.03 to 77.05) | 12 (9 to 15) | 43.41 (33.06 to 55.68) | -39.31  (-47.04 to -30.26) | -1.45  (-1.6 to -1.31) |
| Cameroon | 18 (14 to 23) | 52.18 (39.6 to 66.12) | 35 (26 to 45) | 51.13 (38.35 to 65.68) | 91.18  (68.66 to 113.02) | -0.05  (-0.07 to -0.03) |
| Central African Republic | 4 (3 to 6) | 46.34 (34.43 to 60.47) | 6 (5 to 8) | 42.98 (32.57 to 55.9) | 45.3  (27.78 to 62.34) | -0.31  (-0.33 to -0.29) |
| Chad | 13 (10 to 16) | 53.75 (40.7 to 67.83) | 29 (23 to 37) | 49.63 (38.33 to 63.21) | 130.46  (103.87 to 159.28) | -0.34  (-0.36 to -0.32) |
| China | 2311 (1886 to 2807) | 126.9 (103.56 to 154.14) | 1022 (821 to 1253) | 89.84 (72.19 to 110.2) | -55.79  (-57.29 to -54.23) | -1.03  (-1.17 to -0.88) |
| Colombia | 49 (40 to 59) | 69.8 (56.89 to 83.46) | 43 (35 to 52) | 71.25 (58.65 to 85.78) | -12.86  (-22.45 to -2.3) | 0  (-0.14 to 0.13) |
| Comoros | 1 (1 to 1) | 71.17 (54.45 to 88.91) | 1 (1 to 1) | 65.35 (49.37 to 83.81) | -22.42  (-31.04 to -12.77) | -0.29  (-0.33 to -0.26) |
| Congo | 3 (3 to 4) | 45.7 (34.78 to 59) | 4 (3 to 6) | 40.83 (30.7 to 53.06) | 29.26  (14 to 46.84) | -0.54  (-0.59 to -0.49) |
| Costa Rica | 5 (4 to 6) | 79.35 (65.26 to 98.23) | 3 (2 to 4) | 58.41 (43.93 to 74.31) | -39.53  (-50.21 to -28.49) | -1.31  (-1.53 to -1.08) |
| Côte d'Ivoire | 23 (17 to 30) | 53.64 (40.15 to 69.27) | 34 (25 to 44) | 51.15 (38.35 to 67.05) | 48.26  (31.94 to 65.32) | -0.24  (-0.28 to -0.21) |
| Cuba | 13 (11 to 16) | 94.83 (78.68 to 116.8) | 4 (3 to 5) | 53.18 (40.86 to 66.93) | -67.55  (-72.65 to -62.15) | -2.31  (-2.78 to -1.85) |
| Democratic People's Republic of Korea | 49 (37 to 64) | 90.76 (68.74 to 117.34) | 22 (17 to 28) | 84.11 (64.49 to 106.52) | -55.37  (-59.94 to -49.67) | -0.41  (-0.49 to -0.33) |
| Democratic Republic of the Congo | 66 (50 to 86) | 46.87 (35.49 to 61.24) | 95 (72 to 119) | 43.11 (32.86 to 54.15) | 44.47  (27.1 to 62.18) | -0.38  (-0.42 to -0.34) |
| Djibouti | 1 (1 to 2) | 72.41 (55.75 to 92.69) | 2 (1 to 2) | 64.38 (48.82 to 85.22) | 39.81  (23.22 to 57.85) | -0.44  (-0.46 to -0.43) |
| Dominica | 0 (0 to 0) | 102.9 (84.25 to 124.94) | 0 (0 to 0) | 94.77 (76.28 to 117.32) | -55.18  (-60.42 to -48.95) | -0.25  (-0.47 to -0.04) |
| Dominican Republic | 14 (11 to 18) | 79.15 (63.27 to 99.23) | 11 (9 to 14) | 64.71 (49.48 to 82.57) | -21.65  (-31.58 to -8.89) | -0.88  (-1.09 to -0.66) |
| Ecuador | 19 (15 to 23) | 82.99 (67.98 to 99.6) | 15 (12 to 19) | 58.14 (46.26 to 71.45) | -17.87  (-28.27 to -4.87) | -1.64  (-1.77 to -1.5) |
| Egypt | 147 (113 to 184) | 103.6 (79.76 to 129.32) | 130 (98 to 168) | 80.57 (60.78 to 104.4) | -11.86  (-23.35 to -0.01) | -1.14  (-1.31 to -0.96) |
| El Salvador | 9 (7 to 11) | 69.8 (56.17 to 87.81) | 4 (3 to 5) | 46.16 (34.48 to 59.29) | -54.32  (-60.65 to -46.77) | -1.73  (-2.01 to -1.45) |
| Equatorial Guinea | 1 (1 to 1) | 50.19 (37.81 to 64.39) | 1 (1 to 1) | 39.42 (29.05 to 50.69) | 34.13  (18.95 to 50.91) | -1.06  (-1.17 to -0.95) |
| Eritrea | 7 (5 to 9) | 75.31 (57.35 to 94.37) | 10 (8 to 13) | 65.83 (50.7 to 83.36) | 40.75  (25.72 to 57.17) | -0.61  (-0.71 to -0.51) |
| Eswatini | 1 (1 to 1) | 37.89 (27.95 to 49.55) | 1 (1 to 1) | 36.48 (26.95 to 48.03) | -10.98  (-21.14 to -0.9) | -0.17  (-0.2 to -0.14) |
| Ethiopia | 152 (118 to 190) | 82.45 (63.91 to 103.39) | 186 (143 to 235) | 67.58 (51.92 to 85.58) | 22.39  (15.28 to 30.68) | -0.81  (-0.88 to -0.75) |
| Fiji | 1 (1 to 1) | 64.26 (47.94 to 82.94) | 1 (1 to 1) | 66.04 (50.92 to 83.91) | -5.13  (-18.69 to 6.38) | 0.09  (-0.13 to 0.32) |
| Gabon | 1 (1 to 2) | 46.87 (35.13 to 59.72) | 1 (1 to 2) | 45.44 (34.42 to 58.02) | 5.75  (-5.01 to 18.17) | -0.16  (-0.18 to -0.13) |
| Gambia | 2 (1 to 2) | 54.23 (41.53 to 69.24) | 3 (2 to 4) | 53.25 (40.22 to 68.49) | 46.23  (29.05 to 63.79) | -0.07  (-0.1 to -0.04) |
| Georgia | 5 (4 to 6) | 80.89 (64.84 to 101.42) | 2 (2 to 3) | 61.85 (49.66 to 76.58) | -57.99  (-63.02 to -51.98) | -0.95  (-1.03 to -0.87) |
| Ghana | 27 (21 to 34) | 60.13 (45.83 to 76.16) | 36 (28 to 46) | 56.28 (43.3 to 71.46) | 33.02  (19.44 to 49.75) | -0.35  (-0.4 to -0.3) |
| Grenada | 0 (0 to 0) | 92.46 (76.28 to 112.8) | 0 (0 to 0) | 65.01 (50.7 to 81.46) | -50.68  (-58.04 to -44.26) | -1.3  (-1.38 to -1.22) |
| Guatemala | 17 (14 to 21) | 67.75 (56.4 to 81.64) | 16 (13 to 20) | 51.64 (41.8 to 62.77) | -6.1  (-18.02 to 5.92) | -1.21  (-1.31 to -1.11) |
| Guinea | 13 (10 to 17) | 56.16 (42.5 to 71.73) | 18 (14 to 24) | 51.32 (39.34 to 65.86) | 40.28  (23.69 to 59.43) | -0.45  (-0.51 to -0.38) |
| Guinea-Bissau | 2 (1 to 2) | 54.46 (42.52 to 69.9) | 2 (2 to 3) | 51.17 (39.28 to 65.16) | 27.03  (12.59 to 41.64) | -0.28  (-0.31 to -0.26) |
| Guyana | 2 (2 to 3) | 127.53 (106.76 to 151.03) | 1 (1 to 1) | 103.22 (85.96 to 126.93) | -47.46  (-53.28 to -41.49) | -0.84  (-0.9 to -0.79) |
| Haiti | 21 (15 to 26) | 111.35 (82.88 to 141.78) | 23 (17 to 28) | 90.6 (69.3 to 113.88) | 9.51  (-2 to 19.78) | -0.89  (-0.97 to -0.82) |
| Honduras | 18 (14 to 23) | 134.02 (102.46 to 169.52) | 20 (15 to 26) | 113.77 (84.36 to 146.51) | 11.62  (-0.48 to 25.92) | -0.77  (-0.98 to -0.55) |
| India | 7175 (5657 to 8807) | 365.47 (288.14 to 448.57) | 4344 (3441 to 5344) | 245.04 (194.09 to 301.48) | -39.46  (-42.08 to -37.37) | -1.53  (-1.66 to -1.39) |
| Indonesia | 241 (173 to 320) | 67.31 (48.28 to 89.33) | 121 (88 to 159) | 41.63 (30.38 to 54.6) | -49.79  (-51.84 to -47.41) | -2.03  (-2.31 to -1.74) |
| Iran (Islamic Republic of) | 61 (46 to 77) | 45.13 (34.47 to 57.53) | 46 (35 to 59) | 45.08 (34.55 to 57.48) | -23.82  (-26.09 to -21.37) | 0  (-0.13 to 0.14) |
| Iraq | 47 (36 to 60) | 89.65 (68.25 to 113.58) | 58 (46 to 72) | 80.1 (63.6 to 98.49) | 23.01  (8.13 to 41.4) | -0.6  (-0.76 to -0.44) |
| Jamaica | 4 (3 to 5) | 90.68 (74.7 to 108.94) | 2 (2 to 3) | 82.75 (65.88 to 102.76) | -41.17  (-47.3 to -33.71) | -0.44  (-0.49 to -0.4) |
| Jordan | 5 (4 to 7) | 48.77 (36.09 to 63.41) | 7 (5 to 10) | 37.72 (26.84 to 51.18) | 40.04  (20.38 to 61.66) | -1.28  (-1.45 to -1.11) |
| Kazakhstan | 40 (34 to 47) | 148.51 (126.49 to 175.91) | 28 (23 to 33) | 104.73 (86.74 to 124.57) | -29.63  (-38.49 to -19.59) | -1.47  (-1.82 to -1.13) |
| Kenya | 56 (43 to 71) | 73.87 (56.85 to 94.1) | 78 (59 to 99) | 76.53 (58.53 to 97.41) | 39.63  (36.5 to 43.09) | 0.32  (0.23 to 0.4) |
| Kiribati | 0 (0 to 0) | 45.44 (34.5 to 58.4) | 0 (0 to 0) | 41.71 (30.56 to 54.49) | 8.18  (-6.24 to 22.16) | -0.39  (-0.44 to -0.34) |
| Kyrgyzstan | 7 (6 to 9) | 78.04 (61.11 to 97.26) | 6 (4 to 7) | 51.16 (39.54 to 64.87) | -23.88  (-32.38 to -13.53) | -1.86  (-2.09 to -1.62) |
| Lao People's Democratic Republic | 8 (6 to 10) | 63.26 (46.46 to 81.44) | 7 (5 to 8) | 51.87 (38.75 to 64.94) | -14.27  (-26.32 to -2.06) | -0.86  (-0.93 to -0.8) |
| Lebanon | 6 (5 to 8) | 75.33 (57.97 to 96.03) | 5 (4 to 7) | 65.94 (49.09 to 85.66) | -15.39  (-24.23 to -4.27) | -0.58  (-0.66 to -0.51) |
| Lesotho | 2 (1 to 2) | 38.4 (28.37 to 50.52) | 1 (1 to 2) | 37.18 (27.77 to 48.33) | -25.56  (-34.1 to -15.9) | -0.09  (-0.11 to -0.07) |
| Liberia | 3 (3 to 4) | 55.11 (42.57 to 70.51) | 5 (4 to 6) | 47.38 (35.62 to 59.51) | 45.11  (28.05 to 62.51) | -0.65  (-0.71 to -0.59) |
| Libya | 5 (4 to 6) | 42.78 (32.04 to 55.56) | 2 (2 to 3) | 38.76 (28.7 to 50.06) | -50.27  (-55.69 to -44.45) | -0.41  (-0.44 to -0.38) |
| Madagascar | 27 (21 to 35) | 68.56 (53.77 to 87.52) | 43 (32 to 54) | 66.27 (50.1 to 84) | 56.22  (40.03 to 75.08) | -0.1  (-0.13 to -0.07) |
| Malawi | 24 (18 to 31) | 69.82 (52.81 to 89.77) | 27 (21 to 35) | 66.04 (50.01 to 83.79) | 12.31  (0.14 to 24.85) | -0.24  (-0.26 to -0.23) |
| Malaysia | 19 (14 to 25) | 50.75 (38.11 to 65.15) | 20 (15 to 26) | 48.97 (36.17 to 62.96) | 5.64  (-6.09 to 18.9) | -0.11  (-0.22 to -0.01) |
| Maldives | 0 (0 to 1) | 62.27 (46.66 to 79.69) | 0 (0 to 0) | 47.25 (36.1 to 59.72) | -26.51  (-33.9 to -16.93) | -1.13  (-1.22 to -1.04) |
| Mali | 23 (17 to 29) | 68.83 (52.74 to 87.17) | 48 (37 to 61) | 66.56 (51.61 to 84.41) | 112.25  (87.88 to 139.62) | -0.27  (-0.34 to -0.2) |
| Marshall Islands | 0 (0 to 0) | 52.12 (38.59 to 66.86) | 0 (0 to 0) | 50 (37.19 to 65.59) | -21.88  (-31.34 to -12.39) | -0.18  (-0.2 to -0.17) |
| Mauritania | 4 (3 to 5) | 54.09 (41.45 to 70.31) | 5 (3 to 6) | 55.47 (41.78 to 70.75) | 26.93  (11.41 to 43.81) | 0.12  (0.09 to 0.15) |
| Mexico | 214 (179 to 258) | 114.18 (95.54 to 137.4) | 121 (101 to 145) | 75.68 (63.25 to 91.3) | -43.75  (-45.51 to -41.61) | -2.12  (-2.44 to -1.79) |
| Micronesia (Federated States of) | 0 (0 to 0) | 51.32 (37.87 to 66.23) | 0 (0 to 0) | 47.38 (34.93 to 62.65) | -48.03  (-53.82 to -40.32) | -0.36  (-0.4 to -0.32) |
| Mongolia | 9 (7 to 11) | 159.95 (123.16 to 198.47) | 8 (6 to 10) | 133.21 (100.3 to 169.35) | -7.88  (-18.92 to 4.56) | -0.86  (-1.07 to -0.66) |
| Montenegro | 0 (0 to 0) | 23.39 (17.82 to 29.38) | 0 (0 to 0) | 21.32 (15.66 to 28.03) | -38.77  (-46.64 to -30.43) | -0.51  (-0.58 to -0.44) |
| Morocco | 36 (27 to 45) | 59.83 (45.5 to 75.98) | 24 (18 to 30) | 52.03 (39.59 to 65.74) | -33.22  (-40.67 to -24.98) | -0.6  (-0.65 to -0.56) |
| Mozambique | 37 (28 to 46) | 84.23 (65.27 to 105.38) | 58 (45 to 72) | 68.33 (53.33 to 84.74) | 57.03  (40.73 to 78.49) | -0.95  (-1.04 to -0.87) |
| Myanmar | 65 (48 to 84) | 65.12 (48.63 to 84.7) | 42 (32 to 54) | 53.1 (40.18 to 68.4) | -35.23  (-42.27 to -26.7) | -0.89  (-0.96 to -0.82) |
| Namibia | 1 (1 to 2) | 39.28 (29.4 to 50.93) | 2 (1 to 2) | 38.52 (28.56 to 50.67) | 27.15  (13.72 to 42.85) | -0.08  (-0.09 to -0.07) |
| Nepal | 141 (107 to 181) | 233.18 (176.27 to 298.31) | 76 (58 to 98) | 163.35 (123.86 to 210.96) | -46.31  (-52.16 to -39.59) | -1.89  (-2.09 to -1.69) |
| Nicaragua | 9 (7 to 11) | 80.78 (64.56 to 99.19) | 7 (5 to 8) | 65.51 (51.62 to 81.06) | -25.59  (-33.58 to -16.21) | -0.89  (-1.02 to -0.77) |
| Niger | 19 (14 to 25) | 57.09 (42.96 to 74.23) | 42 (32 to 55) | 48.97 (37.15 to 63.26) | 122.88  (97.17 to 150.55) | -0.74  (-0.84 to -0.64) |
| Nigeria | 193 (148 to 246) | 62.61 (48.11 to 79.73) | 332 (259 to 418) | 58.86 (45.82 to 73.94) | 72.42  (68.16 to 77.47) | -0.33  (-0.37 to -0.29) |
| North Macedonia | 0 (0 to 1) | 18.74 (14.21 to 24.03) | 0 (0 to 0) | 16.8 (12.13 to 22.43) | -42.9  (-50.29 to -34.72) | -0.37  (-0.53 to -0.21) |
| Pakistan | 411 (312 to 526) | 114.81 (87.11 to 147.12) | 533 (403 to 681) | 107.08 (80.98 to 136.74) | 29.85  (21.29 to 38.56) | -0.24  (-0.36 to -0.13) |
| Papua New Guinea | 6 (4 to 7) | 49.84 (37.67 to 63.62) | 12 (9 to 15) | 47.67 (35.29 to 61.13) | 110.68  (83.68 to 138.14) | -0.1  (-0.13 to -0.06) |
| Paraguay | 4 (3 to 5) | 43.96 (34.96 to 55.27) | 3 (2 to 4) | 29.08 (22.71 to 36.93) | -34.39  (-42.56 to -25.16) | -1.74  (-1.88 to -1.6) |
| Peru | 83 (68 to 101) | 166.26 (135.45 to 201.35) | 48 (38 to 59) | 98.48 (78.11 to 121.45) | -42.41  (-49.53 to -33.47) | -2.39  (-2.59 to -2.19) |
| Philippines | 110 (86 to 137) | 70.64 (54.74 to 87.97) | 113 (89 to 141) | 56.32 (44.1 to 70.15) | 2.46  (-0.45 to 5.7) | -0.91  (-0.99 to -0.84) |
| Republic of Moldova | 2 (1 to 2) | 31.49 (25.07 to 38.61) | 1 (1 to 1) | 32.72 (25.65 to 41.27) | -56.56  (-61.14 to -51.31) | 0.07  (-0.11 to 0.26) |
| Russian Federation | 100 (84 to 120) | 68.2 (56.85 to 81.69) | 54 (44 to 65) | 42.04 (34.65 to 51.08) | -46.61  (-48.68 to -44.64) | -2.09  (-2.24 to -1.94) |
| Rwanda | 17 (13 to 21) | 71.12 (54.75 to 89.76) | 17 (13 to 22) | 63.86 (49.49 to 81.99) | 0.47  (-10.68 to 13.65) | -0.46  (-0.49 to -0.43) |
| Saint Lucia | 0 (0 to 0) | 98.74 (82.47 to 119.86) | 0 (0 to 0) | 79.79 (64.71 to 96.99) | -61.89  (-66.37 to -56.59) | -0.81  (-0.9 to -0.73) |
| Saint Vincent and the Grenadines | 0 (0 to 0) | 88.45 (73.69 to 106.95) | 0 (0 to 0) | 65.46 (53.69 to 81.22) | -57.6  (-62.19 to -52.15) | -1.1  (-1.2 to -0.99) |
| Samoa | 0 (0 to 0) | 50.11 (37.05 to 65.26) | 0 (0 to 0) | 48.53 (36.24 to 63.48) | 8.44  (-4 to 22.38) | -0.08  (-0.1 to -0.07) |
| Senegal | 12 (9 to 16) | 48.64 (36.72 to 62.2) | 16 (12 to 21) | 45.77 (34.55 to 58.77) | 28.07  (15.82 to 44.42) | -0.25  (-0.29 to -0.21) |
| Serbia | 2 (2 to 3) | 21.93 (15.84 to 29.72) | 1 (1 to 2) | 21.79 (15.57 to 29.11) | -38.06  (-45.6 to -29.47) | -0.15  (-0.37 to 0.06) |
| Sierra Leone | 7 (5 to 8) | 56.29 (42.22 to 70.92) | 10 (8 to 13) | 48.38 (37.1 to 62.37) | 54.01  (35.99 to 71.67) | -0.69  (-0.74 to -0.64) |
| Solomon Islands | 1 (0 to 1) | 46.47 (33.63 to 61.16) | 1 (1 to 1) | 42.85 (31.88 to 56.65) | 35.13  (20.24 to 49.62) | -0.35  (-0.38 to -0.32) |
| Somalia | 16 (12 to 20) | 70.05 (53.97 to 87.95) | 41 (32 to 53) | 63.36 (48.49 to 80.5) | 156.35  (131.2 to 191.09) | -0.51  (-0.6 to -0.41) |
| South Africa | 28 (21 to 37) | 38.51 (29.3 to 49.89) | 27 (20 to 35) | 34.72 (26.02 to 45.18) | -4.25  (-9.18 to 0.51) | -0.34  (-0.47 to -0.21) |
| South Sudan | 12 (9 to 16) | 65.5 (50.51 to 83.71) | 17 (13 to 21) | 61.2 (47.11 to 78.54) | 35.1  (19.37 to 50.52) | -0.29  (-0.39 to -0.19) |
| Sri Lanka | 25 (20 to 31) | 92.81 (74.41 to 114.07) | 15 (12 to 18) | 65.41 (52.53 to 81.35) | -41.16  (-48.72 to -33.55) | -1.48  (-1.56 to -1.4) |
| Sudan | 40 (30 to 52) | 55.58 (41.39 to 71) | 41 (31 to 51) | 45.8 (35.22 to 58.01) | 0.5  (-11.64 to 16.36) | -0.78  (-0.81 to -0.74) |
| Suriname | 1 (1 to 1) | 136.89 (109.39 to 165.31) | 1 (1 to 1) | 97.75 (77.17 to 121) | -29.15  (-36.77 to -21.75) | -1.26  (-1.33 to -1.2) |
| Syrian Arab Republic | 20 (16 to 25) | 53.34 (42.53 to 65.94) | 8 (6 to 11) | 46.95 (36.26 to 59.37) | -58.93  (-63.23 to -52.27) | -0.47  (-0.51 to -0.43) |
| Tajikistan | 8 (6 to 11) | 53.39 (37.61 to 71.24) | 10 (7 to 14) | 53.95 (38.42 to 72.82) | 25.91  (11.15 to 42.71) | 0.18  (-0.1 to 0.47) |
| Thailand | 66 (49 to 84) | 82.18 (60.47 to 104.57) | 21 (17 to 27) | 48.06 (37.15 to 60.26) | -67.57  (-71.77 to -62.6) | -2.47  (-2.69 to -2.26) |
| Timor-Leste | 2 (1 to 2) | 65.58 (49.9 to 84.12) | 1 (1 to 2) | 51.39 (39.01 to 65.83) | -15.26  (-24.87 to -5.68) | -1.11  (-1.22 to -1) |
| Togo | 7 (5 to 9) | 54.07 (42.04 to 69.78) | 9 (7 to 12) | 52.33 (39.44 to 66.69) | 40.84  (25.07 to 61.35) | -0.14  (-0.19 to -0.1) |
| Tonga | 0 (0 to 0) | 60.86 (45.17 to 79.31) | 0 (0 to 0) | 60.58 (45 to 78.75) | -10.44  (-21.21 to 0.83) | -0.09  (-0.17 to 0) |
| Tunisia | 9 (7 to 11) | 50.61 (37.64 to 64.05) | 6 (4 to 8) | 45.92 (34.38 to 59.57) | -35.51  (-42.43 to -26.92) | -0.39  (-0.42 to -0.35) |
| Turkey | 63 (47 to 82) | 51.22 (37.6 to 66.2) | 38 (27 to 50) | 50.57 (36.76 to 67.3) | -40.68  (-48.68 to -32.27) | -0.43  (-0.78 to -0.08) |
| Turkmenistan | 10 (8 to 12) | 97.07 (80.79 to 117.69) | 11 (9 to 13) | 124.87 (102.23 to 151.02) | 10.45  (-2.46 to 24.9) | 1.28  (0.67 to 1.89) |
| Tuvalu | 0 (0 to 0) | 47.51 (35.64 to 61.09) | 0 (0 to 0) | 47.13 (34.77 to 60.59) | -24.58  (-32.66 to -14.32) | -0.05  (-0.07 to -0.02) |
| Uganda | 45 (34 to 57) | 64.61 (49.64 to 82.15) | 75 (57 to 96) | 63 (47.99 to 80.82) | 67.3  (51.21 to 86.96) | -0.12  (-0.2 to -0.05) |
| Ukraine | 37 (29 to 45) | 72.85 (57.85 to 90.08) | 16 (13 to 20) | 54.84 (44.86 to 66.87) | -55.43  (-60.51 to -49.54) | -1.18  (-1.35 to -1.01) |
| United Republic of Tanzania | 62 (47 to 78) | 67.95 (51.85 to 85.93) | 101 (77 to 127) | 64.78 (49.49 to 81.78) | 62.71  (46.24 to 83.83) | -0.18  (-0.2 to -0.16) |
| Uzbekistan | 87 (73 to 104) | 161.58 (135.12 to 192.03) | 69 (59 to 81) | 127.28 (108.42 to 148.64) | -20.73  (-28.58 to -9.49) | -0.94  (-1.06 to -0.81) |
| Vanuatu | 0 (0 to 0) | 48.86 (35.6 to 64.81) | 0 (0 to 0) | 47.46 (35.35 to 62.25) | 31.55  (16.01 to 49.9) | -0.12  (-0.15 to -0.1) |
| Venezuela (Bolivarian Republic of) | 33 (28 to 39) | 76.72 (63.66 to 90.85) | 20 (15 to 25) | 54.67 (43 to 68.98) | -40.72  (-48.89 to -31.87) | -1.31  (-1.56 to -1.05) |
| Viet Nam | 167 (129 to 213) | 120.92 (93.45 to 154.5) | 99 (75 to 128) | 97.47 (74.3 to 125.95) | -40.7  (-47.95 to -33.04) | -0.91  (-0.99 to -0.83) |
| Yemen | 28 (21 to 36) | 55.99 (42.54 to 71.04) | 34 (26 to 43) | 48.05 (36.64 to 60.67) | 19.61  (8.01 to 35.18) | -0.66  (-0.71 to -0.62) |
| Zambia | 20 (15 to 25) | 68.03 (51.42 to 85.43) | 31 (24 to 40) | 67.07 (51.44 to 86.5) | 58.63  (40.89 to 82.81) | -0.04  (-0.09 to 0.01) |
| Zimbabwe | 12 (9 to 16) | 43.2 (32.44 to 54.76) | 15 (11 to 20) | 46.03 (34.17 to 59.39) | 23.61  (9.33 to 41.22) | 0.31  (0.15 to 0.47) |

CI, confidence interval; EAPC, estimated annual percentage change; LMICs, low- and middle-income countries and territories; UI, uncertain interval.

## Supplementary Table S10. National burden and trends of incident cases and incidence rates (per 100,000 liveborn neonates) of enteric infections in 131 LMICs between 1990 and 2019.

| **Location** | **Incident cases in 1990**  **(95% UI)** | **Incident rate in 1990**  **( 95% UI)** | **Incident cases in 2019**  **(95% UI)** | **Incident rate in 2019**  **( 95% UI)** | **Percent change**  **(%, 95% CI)** | **EAPC**  **(%, 95% CI)** |
| --- | --- | --- | --- | --- | --- | --- |
| Global | 32884255 (29461746 to 35199398) | 318505.01 (285355.83 to 340928.65) | 26355396 (23161513 to 28908819) | 257922.17 (226665.83 to 282910.77) | -19.85  (-22.12 to -17.12) | -0.6  (-0.7 to -0.49) |
| Afghanistan | 141141 (129147 to 145904) | 364802.79 (333802.96 to 377115.27) | 407153 (375351 to 421481) | 364048.81 (335613.64 to 376859.95) | 188.47  (175.76 to 200.5) | -0.01  (-0.04 to 0.01) |
| Albania | 23111 (20697 to 24204) | 355448.73 (318328.54 to 372252.46) | 9217 (7970 to 9889) | 345870.75 (299093.19 to 371122.32) | -60.12  (-65.52 to -57.33) | -0.12  (-0.14 to -0.09) |
| Algeria | 205799 (178967 to 218747) | 354333.16 (308136.1 to 376626.6) | 235017 (208385 to 249011) | 354376.7 (314218.69 to 375478.5) | 14.2  (7.04 to 23.69) | -0.06  (-0.09 to -0.04) |
| American Samoa | 371 (294 to 443) | 286567.9 (227166.39 to 342469.28) | 215 (167 to 264) | 268756.56 (208842.89 to 330513.33) | -42.04  (-49.36 to -33) | -0.18  (-0.26 to -0.1) |
| Angola | 138973 (130498 to 143339) | 366735.11 (344371.12 to 378256.18) | 297877 (260154 to 314311) | 357275.06 (312029.26 to 376986.13) | 114.34  (89.95 to 128.27) | -0.11  (-0.14 to -0.09) |
| Armenia | 20841 (20098 to 21232) | 362897.38 (349951.24 to 369700.85) | 8414 (6829 to 9927) | 294217.55 (238776.12 to 347127.9) | -59.63  (-67.35 to -52.23) | -0.71  (-0.86 to -0.56) |
| Azerbaijan | 51979 (50045 to 53010) | 363404.54 (349887.72 to 370613.89) | 37266 (31410 to 40132) | 342842.44 (288965.86 to 369205.12) | -28.31  (-39.89 to -22.44) | -0.24  (-0.26 to -0.21) |
| Bangladesh | 694579 (564391 to 833922) | 237286.97 (192811.46 to 284890.32) | 325061 (253736 to 397256) | 159997.58 (124890.85 to 195532.46) | -53.2  (-60.5 to -44.53) | -1.79  (-2.1 to -1.48) |
| Belarus | 35855 (30864 to 39555) | 334344.13 (287806.13 to 368842.46) | 15099 (11584 to 18521) | 192160.74 (147416.73 to 235709.91) | -57.89  (-66.63 to -49.16) | -2.72  (-3.01 to -2.43) |
| Belize | 1620 (1514 to 1667) | 363566.07 (339937.82 to 374157.47) | 2069 (1870 to 2169) | 355783.36 (321542.51 to 372999.7) | 27.73  (18.25 to 34.44) | -0.1  (-0.12 to -0.08) |
| Benin | 63825 (57685 to 67029) | 358724.44 (324217.96 to 376733.73) | 124065 (98464 to 140545) | 328064.4 (260369.34 to 371643.21) | 94.38  (58.38 to 121.73) | -0.45  (-0.5 to -0.4) |
| Bhutan | 4639 (3652 to 5583) | 282254.03 (222216.06 to 339698.49) | 2139 (1638 to 2649) | 211076.87 (161591.82 to 261343.69) | -53.88  (-59.73 to -47.46) | -1.17  (-1.24 to -1.11) |
| Bolivia (Plurinational State of) | 61967 (50535 to 68899) | 339317.84 (276720.34 to 377273.83) | 78489 (62814 to 90155) | 321733.29 (257481.65 to 369554.16) | 26.66  (7.6 to 44.02) | -0.25  (-0.31 to -0.19) |
| Bosnia and Herzegovina | 18510 (16716 to 19381) | 358020.37 (323313.52 to 374857.44) | 7143 (6273 to 7579) | 349219.88 (306667.81 to 370524.11) | -61.41  (-65.08 to -58.93) | -0.13  (-0.17 to -0.09) |
| Botswana | 11305 (10756 to 11596) | 367785.6 (349913.56 to 377253.95) | 12637 (11050 to 13550) | 347166.05 (303584.21 to 372250.48) | 11.78  (-2.02 to 20.89) | -0.2  (-0.24 to -0.17) |
| Brazil | 880174 (815255 to 929373) | 339124.02 (314111.21 to 358079.96) | 568791 (468906 to 663542) | 242060.65 (199552.75 to 282384.03) | -35.38  (-43.83 to -26.82) | -1.03  (-1.21 to -0.85) |
| Bulgaria | 23663 (19420 to 27135) | 315376.59 (258821.83 to 361645.24) | 14413 (11497 to 16656) | 314870.5 (251171.48 to 363875.18) | -39.09  (-47.97 to -30.36) | 0.02  (-0.04 to 0.07) |
| Burkina Faso | 126040 (116751 to 129684) | 367699.7 (340599.53 to 378330.6) | 255797 (232161 to 266101) | 362660.47 (329149.52 to 377268.33) | 102.95  (89.29 to 111.05) | -0.06  (-0.08 to -0.05) |
| Burundi | 71577 (65762 to 74683) | 361835.31 (332438.28 to 377532.41) | 127548 (116158 to 132624) | 363669.54 (331194.33 to 378142.6) | 78.2  (70.78 to 86.4) | 0.03  (0.01 to 0.05) |
| Cabo Verde | 3270 (3091 to 3363) | 367623.33 (347524.63 to 378189.62) | 2753 (2311 to 3001) | 338177.5 (283802.47 to 368587.41) | -15.79  (-29.29 to -8.16) | -0.31  (-0.33 to -0.29) |
| Cambodia | 120087 (112527 to 123862) | 366320.21 (343259.14 to 377837.43) | 96049 (82868 to 103225) | 346607.51 (299040.84 to 372501.05) | -20.02  (-31.34 to -13.03) | -0.21  (-0.25 to -0.18) |
| Cameroon | 128143 (119168 to 131887) | 367753.29 (341997.35 to 378498.62) | 246481 (225113 to 256799) | 362602.32 (331167.18 to 377781.36) | 92.35  (77.86 to 102.22) | -0.06  (-0.07 to -0.04) |
| Central African Republic | 34440 (32047 to 35579) | 366153.3 (340712.81 to 378261.15) | 53725 (49525 to 55690) | 364574.46 (336071.71 to 377906.07) | 56  (49.35 to 61.61) | -0.02  (-0.02 to -0.01) |
| Chad | 87693 (82887 to 89759) | 369958.28 (349680.48 to 378675.09) | 219635 (210039 to 224296) | 371251.66 (355030.37 to 379128.65) | 150.46  (145.32 to 158.02) | 0.01  (0 to 0.02) |
| China | 5859498 (5029179 to 6435281) | 321756.25 (276161.84 to 353373.56) | 1764474 (1423954 to 2103218) | 155145.02 (125204.11 to 184929.8) | -69.89  (-72.66 to -66.44) | -3.27  (-3.65 to -2.9) |
| Colombia | 261915 (254061 to 267186) | 371300.81 (360166.87 to 378774.08) | 140649 (116418 to 169299) | 233553.84 (193318.15 to 281129.02) | -46.3  (-55.45 to -35.3) | -1.79  (-2.05 to -1.53) |
| Comoros | 5373 (4964 to 5559) | 365181.32 (337426.41 to 377832.95) | 4481 (4094 to 4684) | 360488.24 (329336.16 to 376771.58) | -16.59  (-21.53 to -13.28) | -0.07  (-0.08 to -0.05) |
| Congo | 27358 (25130 to 28381) | 364424.87 (334750.7 to 378050.77) | 38211 (32758 to 40834) | 351855.02 (301645.92 to 376011.06) | 39.67  (22.35 to 51.01) | -0.13  (-0.16 to -0.1) |
| Costa Rica | 15710 (12640 to 18957) | 255064.45 (205215.3 to 307776.53) | 8159 (6750 to 9949) | 161244.12 (133399.26 to 196640.37) | -48.07  (-56.81 to -37.56) | -1.6  (-1.86 to -1.35) |
| Côte d'Ivoire | 155740 (144349 to 160990) | 365533.28 (338796.86 to 377855.33) | 236098 (208078 to 250012) | 356406.45 (314108.12 to 377410.29) | 51.6  (36.14 to 61.87) | -0.1  (-0.11 to -0.08) |
| Cuba | 39579 (33273 to 45528) | 286840.81 (241141.15 to 329959.76) | 22123 (17550 to 26384) | 277105.11 (219823.48 to 330481.81) | -44.1  (-53.53 to -34.45) | -0.32  (-0.44 to -0.19) |
| Democratic People's Republic of Korea | 179947 (143357 to 202489) | 331738.79 (264284.14 to 373295.72) | 85328 (66476 to 96901) | 326624.68 (254460.62 to 370922.2) | -52.58  (-58.06 to -47.66) | -0.16  (-0.21 to -0.11) |
| Democratic Republic of the Congo | 502808 (452001 to 527148) | 358960.4 (322688.6 to 376337.59) | 791590 (706811 to 831848) | 359746.93 (321218.19 to 378042.54) | 57.43  (47.68 to 65.17) | 0  (-0.03 to 0.03) |
| Djibouti | 5966 (5420 to 6254) | 359688.5 (326727.23 to 377030.52) | 8982 (7495 to 9758) | 344330.38 (287303.07 to 374074.76) | 50.55  (30.59 to 63.25) | -0.23  (-0.26 to -0.19) |
| Dominica | 458 (408 to 482) | 353797.16 (315278.79 to 372153.19) | 216 (184 to 234) | 342336.02 (291558.72 to 371963.26) | -52.91  (-59 to -48.86) | -0.14  (-0.15 to -0.13) |
| Dominican Republic | 67089 (64942 to 68158) | 372406.72 (360490.92 to 378341.73) | 62552 (57869 to 65099) | 362335.22 (335209.06 to 377085.59) | -6.76  (-13.79 to -3.06) | -0.09  (-0.11 to -0.08) |
| Ecuador | 82544 (76719 to 85636) | 364394.67 (338680.28 to 378043.66) | 73428 (59288 to 87719) | 276501.92 (223256.21 to 330313.85) | -11.04  (-27.19 to 5.95) | -0.93  (-1.14 to -0.72) |
| Egypt | 533164 (522160 to 538156) | 375288.35 (367542.69 to 378801.83) | 593626 (565168 to 608030) | 368693.46 (351018.47 to 377639.8) | 11.34  (5.96 to 14.04) | -0.06  (-0.07 to -0.06) |
| El Salvador | 45545 (42810 to 46772) | 367720.03 (345633.8 to 377627.49) | 22851 (18045 to 27670) | 267098.25 (210920.35 to 323431.3) | -49.83  (-59.87 to -39.73) | -1.27  (-1.4 to -1.13) |
| Equatorial Guinea | 6338 (6012 to 6480) | 370643.2 (351577.7 to 378916.46) | 9576 (7673 to 10781) | 327867.57 (262702.56 to 369129) | 51.08  (19.78 to 71.37) | -0.5  (-0.57 to -0.43) |
| Eritrea | 33648 (30082 to 35215) | 360836.92 (322604.49 to 377640.76) | 54557 (49695 to 56714) | 363377.38 (330991.92 to 377744.68) | 62.14  (54.16 to 74.37) | 0.02  (0.01 to 0.03) |
| Eswatini | 8870 (8411 to 9077) | 369440.25 (350301.35 to 378029.23) | 8073 (7471 to 8378) | 363671.63 (336546.61 to 377432.06) | -8.99  (-15.64 to -5.59) | -0.06  (-0.07 to -0.05) |
| Ethiopia | 657384 (593936 to 691355) | 356929.21 (322480.15 to 375374.19) | 964525 (860632 to 1021744) | 350703.13 (312927.28 to 371508.24) | 46.72  (39.62 to 52.67) | -0.08  (-0.1 to -0.05) |
| Fiji | 5077 (4433 to 5401) | 352418.24 (307698.28 to 374949.12) | 4432 (3794 to 4914) | 333262.36 (285280.38 to 369566.91) | -12.71  (-22.33 to -4.42) | -0.24  (-0.28 to -0.21) |
| Gabon | 10561 (9466 to 11107) | 357605.59 (320546.02 to 376109.95) | 10119 (8021 to 11777) | 314109.88 (248966.83 to 365553.82) | -4.18  (-21.08 to 11.88) | -0.5  (-0.55 to -0.46) |
| Gambia | 13055 (11935 to 13623) | 362327.41 (331241.23 to 378091.09) | 18948 (16545 to 20220) | 353145.99 (308358.36 to 376842.9) | 45.14  (28.43 to 55.19) | -0.09  (-0.12 to -0.05) |
| Georgia | 22610 (20698 to 23462) | 357744.37 (327495.83 to 371234.59) | 8140 (6545 to 9729) | 234392.12 (188466.34 to 280140.04) | -64  (-70.8 to -56.62) | -1.78  (-2.06 to -1.49) |
| Ghana | 164242 (152257 to 170652) | 363419.79 (336899.95 to 377602.61) | 225020 (194919 to 241964) | 350294.79 (303435.96 to 376671.53) | 37  (19.99 to 47.9) | -0.11  (-0.13 to -0.09) |
| Grenada | 537 (488 to 564) | 356970.61 (324250.08 to 374491.46) | 363 (319 to 395) | 344021.07 (301937.23 to 373915.94) | -32.4  (-39.45 to -27.24) | -0.21  (-0.27 to -0.15) |
| Guatemala | 94245 (87468 to 97186) | 370994.23 (344315.95 to 382572.32) | 65813 (54179 to 80580) | 210317.19 (173136.99 to 257505.72) | -30.17  (-41.9 to -14.72) | -2.33  (-2.51 to -2.16) |
| Guinea | 85191 (77961 to 88398) | 363667.21 (332803.16 to 377358.25) | 121650 (103916 to 132811) | 338274.11 (288963 to 369312.02) | 42.8  (22.88 to 56.44) | -0.27  (-0.32 to -0.21) |
| Guinea-Bissau | 12506 (11438 to 13082) | 361242.7 (330391.94 to 377884.16) | 16399 (14318 to 17586) | 350373.38 (305911.67 to 375738.41) | 31.12  (16.23 to 42) | -0.15  (-0.17 to -0.13) |
| Guyana | 6126 (5916 to 6244) | 366993.99 (354377.25 to 374062.7) | 3868 (3511 to 4034) | 356934.34 (324018.6 to 372255.5) | -36.86  (-42.52 to -34.23) | -0.09  (-0.1 to -0.08) |
| Haiti | 68888 (66817 to 69920) | 372974.18 (361764.67 to 378563.93) | 92137 (87647 to 94163) | 370648.63 (352585.69 to 378801.61) | 33.75  (27.82 to 37.43) | -0.02  (-0.02 to -0.01) |
| Honduras | 50093 (47763 to 51312) | 369341.48 (352163.71 to 378325.32) | 62758 (54471 to 67102) | 351912.88 (305440.06 to 376271.82) | 25.28  (9.2 to 34.04) | -0.15  (-0.17 to -0.13) |
| India | 6252041 (5273982 to 6922595) | 318440.79 (268624.42 to 352594.74) | 2868557 (2294843 to 3481811) | 161823.49 (129458.61 to 196418.89) | -54.12  (-57.51 to -48.7) | -0.73  (-1.39 to -0.06) |
| Indonesia | 1279043 (1169315 to 1335099) | 356505.87 (325921.49 to 372130.2) | 1012467 (907047 to 1069220) | 347622.47 (311427.52 to 367108.08) | -20.84  (-25.11 to -18.43) | -0.06  (-0.16 to 0.03) |
| Iran (Islamic Republic of) | 458715 (415844 to 486734) | 341145.35 (309262.61 to 361982.88) | 332393 (289078 to 361145) | 324117.49 (281880.61 to 352153.4) | -27.54  (-32.95 to -23.99) | -0.17  (-0.2 to -0.15) |
| Iraq | 193653 (182959 to 199305) | 366481.76 (346242.7 to 377177.52) | 265523 (245770 to 273643) | 364967.04 (337815.3 to 376128.59) | 37.11  (30.55 to 43.11) | -0.02  (-0.03 to -0.01) |
| Jamaica | 15599 (14904 to 15988) | 367268.62 (350905.83 to 376438.94) | 9677 (8655 to 10206) | 353432.81 (316077.42 to 372732.58) | -37.96  (-44.26 to -34.75) | -0.16  (-0.24 to -0.07) |
| Jordan | 34101 (27897 to 38246) | 330373.91 (270271.71 to 370532.31) | 63857 (53659 to 69921) | 341707.77 (287137.3 to 374162.35) | 87.26  (68.59 to 116.24) | 0.18  (0.11 to 0.24) |
| Kazakhstan | 96131 (88795 to 99702) | 356914.02 (329677.78 to 370174.1) | 41794 (33818 to 50466) | 155520.87 (125838.63 to 187790.2) | -56.52  (-64.67 to -47.95) | -3.51  (-3.83 to -3.2) |
| Kenya | 263577 (242743 to 276569) | 350252.31 (322566.47 to 367515.56) | 348209 (317623 to 367831) | 343299.62 (313144.59 to 362645.28) | 32.11  (29.28 to 34.49) | -0.09  (-0.14 to -0.03) |
| Kiribati | 702 (636 to 733) | 361732.63 (327531.13 to 377589.98) | 797 (690 to 855) | 348454.45 (301763.45 to 373773.92) | 13.53  (-0.94 to 23.06) | -0.17  (-0.18 to -0.16) |
| Kyrgyzstan | 34388 (32740 to 35325) | 361481.76 (344159.35 to 371326.05) | 26550 (21672 to 32117) | 240352.79 (196192.14 to 290748.03) | -22.79  (-35.52 to -7.23) | -1.67  (-1.9 to -1.44) |
| Lao People's Democratic Republic | 43240 (37098 to 46574) | 349461.73 (299824.2 to 376406.39) | 39279 (31032 to 46382) | 303632.64 (239883.99 to 358543.77) | -9.16  (-23.6 to 7.12) | -0.71  (-0.77 to -0.64) |
| Lebanon | 29854 (27026 to 31133) | 359641.76 (325564.33 to 375039.89) | 28923 (26052 to 30223) | 360459.15 (324680.95 to 376664.4) | -3.12  (-8.17 to 2.15) | -0.02  (-0.03 to 0) |
| Lesotho | 15510 (12701 to 16936) | 342439.03 (280413.03 to 373932.05) | 12761 (12101 to 13119) | 366400.22 (347446.87 to 376680.69) | -17.73  (-25.45 to 0.98) | 0.65  (0.46 to 0.84) |
| Liberia | 20986 (18615 to 22237) | 349994.53 (310445.42 to 370861.54) | 35237 (29809 to 38112) | 348131.14 (294500.68 to 376528.79) | 67.91  (48.52 to 81.9) | -0.09  (-0.15 to -0.02) |
| Libya | 39681 (34327 to 42295) | 350686.19 (303375.58 to 373789.44) | 21994 (19616 to 23278) | 354154.79 (315851.22 to 374817.27) | -44.57  (-47.43 to -38.86) | 0.01  (-0.02 to 0.03) |
| Madagascar | 146327 (137212 to 150803) | 366686.23 (343845.26 to 377903.16) | 233305 (213090 to 243265) | 361771.5 (330425.4 to 377216.21) | 59.44  (51.19 to 65.81) | -0.05  (-0.06 to -0.03) |
| Malawi | 128155 (119697 to 131799) | 368529.35 (344206.4 to 379006.7) | 148859 (135601 to 155765) | 360515.51 (328406.62 to 377241.73) | 16.16  (7.43 to 21.61) | -0.07  (-0.08 to -0.06) |
| Malaysia | 89834 (69212 to 110070) | 237751.01 (183172.19 to 291306.69) | 108524 (83155 to 133837) | 262304.85 (200987.63 to 323487.75) | 20.8  (7.2 to 37.08) | 0.52  (0.29 to 0.75) |
| Maldives | 2335 (2060 to 2484) | 352835.38 (311242.02 to 375271.08) | 2210 (1868 to 2402) | 344876.8 (291456.37 to 374789.52) | -5.34  (-14 to 2.38) | -0.15  (-0.23 to -0.06) |
| Mali | 121413 (114282 to 124509) | 369584.37 (347879.03 to 379008.73) | 258457 (236691 to 271457) | 358431.59 (328245.43 to 376459.68) | 112.87  (96.73 to 123.91) | -0.13  (-0.14 to -0.11) |
| Marshall Islands | 415 (381 to 433) | 361246.12 (331736.81 to 376863.58) | 296 (237 to 344) | 316451.83 (253319.68 to 366957.49) | -28.67  (-42.4 to -17.75) | -0.61  (-0.67 to -0.55) |
| Mauritania | 24419 (22446 to 25219) | 366286.37 (336679.46 to 378278.38) | 30274 (27662 to 31233) | 366909.46 (335257.4 to 378527.85) | 23.98  (20.13 to 27.68) | -0.01  (-0.03 to 0) |
| Mexico | 627768 (570252 to 669158) | 334396.66 (303759.71 to 356444.48) | 318413 (267168 to 370612) | 199836.57 (167674.81 to 232596.54) | -49.28  (-55.02 to -43.69) | -2  (-2.14 to -1.85) |
| Micronesia (Federated States of) | 920 (790 to 985) | 349998.82 (300442.17 to 374731.08) | 451 (360 to 531) | 304591.56 (243142.93 to 358560.94) | -51.01  (-58.74 to -43.15) | -0.69  (-0.77 to -0.62) |
| Mongolia | 19907 (18194 to 20710) | 361420.97 (330315.93 to 376004) | 20577 (17159 to 22484) | 337765.14 (281651.71 to 369066.48) | 3.37  (-12.5 to 13.02) | -0.25  (-0.26 to -0.23) |
| Montenegro | 1833 (1459 to 2231) | 241070.92 (191806.27 to 293340.74) | 1278 (972 to 1614) | 250197.1 (190203.93 to 315837.18) | -30.28  (-40.74 to -19.81) | -0.15  (-0.24 to -0.06) |
| Morocco | 220819 (210730 to 224886) | 371456.49 (354484.68 to 378297.39) | 164977 (152406 to 171728) | 361397.28 (333858.92 to 376186.59) | -25.29  (-30.9 to -22.03) | -0.11  (-0.12 to -0.1) |
| Mozambique | 155875 (138865 to 164735) | 357027.74 (318066.25 to 377319.5) | 275409 (222432 to 312063) | 325884.82 (263197.64 to 369256.44) | 76.69  (47.95 to 98.42) | -0.38  (-0.46 to -0.3) |
| Myanmar | 363045 (341115 to 375033) | 365842.53 (343742.75 to 377922.8) | 276399 (243362 to 293721) | 350659.56 (308746.53 to 372634.81) | -23.87  (-31.31 to -19.54) | -0.22  (-0.25 to -0.2) |
| Namibia | 13320 (12467 to 13769) | 365321.41 (341947.95 to 377650.06) | 16848 (15178 to 17774) | 356351.61 (321031.41 to 375931.72) | 26.49  (13.75 to 33.97) | -0.06  (-0.08 to -0.04) |
| Nepal | 208555 (177328 to 227482) | 343883.48 (292393.51 to 375092.43) | 109045 (85541 to 130578) | 234593.82 (184028.78 to 280918.55) | -47.71  (-56.11 to -37.45) | -1.41  (-1.62 to -1.2) |
| Nicaragua | 40036 (37959 to 41005) | 368650.55 (349524.61 to 377568.51) | 25177 (19567 to 31085) | 252661.7 (196359.31 to 311949.54) | -37.11  (-51.59 to -22.71) | -1.45  (-1.55 to -1.34) |
| Niger | 122286 (114343 to 125716) | 367938.88 (344040.39 to 378258.23) | 316400 (289681 to 326873) | 366341.67 (335404.45 to 378467.6) | 158.74  (148.9 to 165.84) | -0.02  (-0.03 to -0.01) |
| Nigeria | 1109115 (1011411 to 1158063) | 360104.6 (328382.3 to 375997.18) | 1855335 (1537105 to 2057276) | 328477.07 (272136.2 to 364229.72) | 67.28  (42.44 to 81.81) | -0.39  (-0.43 to -0.35) |
| North Macedonia | 9735 (9412 to 9909) | 366214.28 (354056.71 to 372754.6) | 5920 (5193 to 6306) | 349543.5 (306623.63 to 372337.81) | -39.19  (-46.33 to -34.97) | -0.19  (-0.21 to -0.16) |
| Pakistan | 1203344 (1038049 to 1308489) | 336450.2 (290234.25 to 365848.16) | 1347639 (1072037 to 1610859) | 270632.23 (215286 to 323491.84) | 11.99  (-2.94 to 28.25) | -0.63  (-0.74 to -0.53) |
| Papua New Guinea | 40782 (37303 to 42405) | 363664.11 (332641.69 to 378133.34) | 88330 (78967 to 92919) | 357563.28 (319661.67 to 376137.35) | 116.59  (99.61 to 126.53) | -0.08  (-0.09 to -0.07) |
| Paraguay | 34242 (30662 to 36332) | 353690.48 (316716.68 to 375281.3) | 25433 (20159 to 31385) | 264839.71 (209924.23 to 326823.2) | -25.73  (-38.06 to -8.1) | -1.28  (-1.41 to -1.15) |
| Peru | 182891 (167750 to 189592) | 366148.6 (335837.34 to 379564.26) | 159559 (131593 to 179398) | 328545.35 (270960.97 to 369395.11) | -12.76  (-26.89 to -0.58) | -0.74  (-0.87 to -0.62) |
| Philippines | 512806 (441292 to 559505) | 328130.73 (282370.59 to 358012.36) | 495597 (400336 to 589511) | 246770.18 (199337.52 to 293532.53) | -3.36  (-12.72 to 7.53) | -0.97  (-1.06 to -0.88) |
| Republic of Moldova | 20882 (19114 to 21764) | 356329.99 (326164.69 to 371372.84) | 6456 (5274 to 7709) | 263521.43 (215251.79 to 314666.39) | -69.08  (-74.3 to -63.34) | -1.28  (-1.46 to -1.1) |
| Russian Federation | 483378 (428925 to 522767) | 328373.23 (291381.1 to 355130.9) | 314572 (262401 to 363343) | 246725.48 (205807.23 to 284978.04) | -34.92  (-40.67 to -28.83) | -0.97  (-1.11 to -0.83) |
| Rwanda | 84532 (73690 to 89922) | 355436.12 (309847.41 to 378097.52) | 93049 (79854 to 99894) | 349668.08 (300083.98 to 375393.27) | 10.07  (-0.26 to 19.34) | -0.09  (-0.11 to -0.07) |
| Saint Lucia | 1040 (973 to 1072) | 362636.87 (339513.57 to 374012.77) | 490 (447 to 508) | 362387.31 (330454.21 to 375585.61) | -52.87  (-55.62 to -50.36) | -0.01  (-0.02 to 0) |
| Saint Vincent and the Grenadines | 729 (692 to 748) | 363093 (344384.27 to 372524.77) | 410 (368 to 430) | 356322.21 (319792.83 to 373562.78) | -43.78  (-48.67 to -41.25) | -0.07  (-0.09 to -0.06) |
| Samoa | 668 (525 to 833) | 256467.36 (201434.66 to 319794.16) | 757 (577 to 937) | 259648.67 (197842.84 to 321233.08) | 13.36  (-0.41 to 30.26) | -0.14  (-0.26 to -0.02) |
| Senegal | 94516 (89187 to 97086) | 368404.51 (347633.44 to 378422.05) | 125021 (111279 to 132064) | 358025.78 (318673.13 to 378195.02) | 32.28  (17.29 to 40.52) | -0.11  (-0.13 to -0.09) |
| Serbia | 19094 (15106 to 23259) | 189055.08 (149569.79 to 230298.69) | 17668 (13926 to 20622) | 280537.01 (221119.78 to 327444.2) | -7.47  (-20.02 to 6.04) | 1.58  (1.51 to 1.66) |
| Sierra Leone | 42596 (38754 to 44252) | 364028.48 (331190.43 to 378175.92) | 71332 (59249 to 78191) | 340189.56 (282563.03 to 372897.94) | 67.46  (40.37 to 84.84) | -0.35  (-0.39 to -0.3) |
| Solomon Islands | 3960 (3733 to 4067) | 367624.74 (346561.78 to 377620.32) | 5713 (5205 to 5947) | 361964.41 (329800.98 to 376825.77) | 44.27  (32.5 to 50.16) | -0.08  (-0.1 to -0.07) |
| Somalia | 81814 (71376 to 86720) | 355251.24 (309927.11 to 376552.08) | 234716 (213195 to 245990) | 359600.83 (326628.04 to 376873.15) | 186.89  (171.16 to 218.43) | 0  (-0.01 to 0.01) |
| South Africa | 256188 (233243 to 270012) | 349303.4 (318019.18 to 368152.69) | 181855 (151099 to 216691) | 233425.26 (193946.86 to 278139.63) | -29.01  (-38.03 to -17.27) | -1.93  (-2.28 to -1.58) |
| South Sudan | 68989 (65002 to 70884) | 368162.88 (346886.17 to 378273.76) | 99656 (94155 to 102483) | 367802.07 (347498.3 to 378233.66) | 44.45  (40.84 to 47.75) | 0  (-0.01 to 0) |
| Sri Lanka | 93070 (81698 to 100219) | 343537.17 (301561.43 to 369924.98) | 58419 (45771 to 71026) | 258282.99 (202364.44 to 314020.25) | -37.23  (-47.82 to -24.79) | -1.21  (-1.28 to -1.15) |
| Sudan | 270928 (259102 to 275173) | 372879.27 (356602.93 to 378721.33) | 330806 (318784 to 335564) | 373339.47 (359772.36 to 378709.23) | 22.1  (19.92 to 24.66) | 0.01  (0 to 0.01) |
| Suriname | 2510 (2392 to 2562) | 366668.94 (349391.86 to 374279.2) | 2444 (2269 to 2528) | 359843.16 (334129.07 to 372124.56) | -2.62  (-10.06 to 1.16) | -0.08  (-0.09 to -0.08) |
| Syrian Arab Republic | 137001 (122852 to 142507) | 360602.26 (323359.28 to 375095.16) | 57256 (46199 to 65218) | 323014.86 (260635.51 to 367936) | -58.21  (-66.29 to -52.25) | -0.47  (-0.52 to -0.43) |
| Tajikistan | 57367 (55281 to 58368) | 371234.24 (357736.12 to 377709.72) | 65600 (56204 to 71267) | 340699.75 (291900.35 to 370128.84) | 14.35  (-1.82 to 24.78) | -0.31  (-0.36 to -0.27) |
| Thailand | 263542 (221776 to 295748) | 327789.25 (275841.34 to 367847) | 122633 (97771 to 147154) | 275011.66 (219256.98 to 330001.35) | -53.47  (-60.82 to -45.81) | -0.91  (-1.03 to -0.78) |
| Timor-Leste | 9812 (8961 to 10149) | 365638.05 (333908.94 to 378186) | 9790 (8024 to 10831) | 337333.48 (276491.92 to 373211.91) | -0.23  (-17.31 to 11.16) | -0.38  (-0.42 to -0.34) |
| Togo | 45227 (42823 to 46294) | 370237.61 (350556.09 to 378974.57) | 65207 (60555 to 67220) | 366852.42 (340678.5 to 378176.68) | 44.18  (37.3 to 48.94) | -0.04  (-0.06 to -0.03) |
| Tonga | 591 (460 to 705) | 299382.56 (233170.15 to 357122.92) | 425 (336 to 528) | 238943.5 (189013.24 to 297275.07) | -28.19  (-38.57 to -15.28) | -1.07  (-1.23 to -0.91) |
| Tunisia | 64140 (57432 to 67407) | 358619.96 (321115.68 to 376888.52) | 44576 (38942 to 47548) | 350659.5 (306340.34 to 374037.93) | -30.5  (-37.16 to -25.03) | -0.05  (-0.07 to -0.03) |
| Turkey | 403422 (322664 to 461645) | 325630.78 (260445.54 to 372626.71) | 253773 (212994 to 279265) | 340958.16 (286168.44 to 375207.02) | -37.09  (-44.38 to -26.71) | 0.19  (0.13 to 0.24) |
| Turkmenistan | 36537 (35092 to 37202) | 369609.35 (354993.16 to 376334.16) | 23300 (19484 to 28146) | 274522.08 (229561.86 to 331618.6) | -36.23  (-46.84 to -22.93) | -0.95  (-1.16 to -0.73) |
| Tuvalu | 75 (65 to 81) | 347856.11 (299825.72 to 373080.3) | 52 (41 to 60) | 314373.8 (246906.99 to 366277.06) | -31.3  (-42.28 to -20.36) | -0.5  (-0.55 to -0.46) |
| Uganda | 253131 (233991 to 261300) | 366153.01 (338467.42 to 377969.39) | 420494 (370943 to 447236) | 354495.42 (312721.68 to 377039.54) | 66.12  (50.41 to 76.24) | -0.14  (-0.16 to -0.12) |
| Ukraine | 171252 (152998 to 184424) | 341520.26 (305116.84 to 367789.7) | 52161 (41708 to 63197) | 175686.65 (140478.7 to 212855) | -69.54  (-74.53 to -63.61) | -3.03  (-3.36 to -2.71) |
| United Republic of Tanzania | 325378 (295936 to 341968) | 357773.26 (325400.31 to 376015.17) | 485009 (384729 to 566065) | 312446.45 (247845.36 to 364663.57) | 49.06  (21.77 to 72.49) | -0.53  (-0.61 to -0.44) |
| Uzbekistan | 197935 (189259 to 202249) | 367172.66 (351079.07 to 375174.81) | 94763 (76795 to 115344) | 174692.14 (141569.86 to 212632.97) | -52.12  (-61.42 to -41.13) | -3.18  (-3.47 to -2.89) |
| Vanuatu | 1571 (1442 to 1627) | 364344.73 (334445.69 to 377300.7) | 2117 (1945 to 2202) | 362425.91 (332957.4 to 376956.38) | 34.72  (27.7 to 41.62) | -0.04  (-0.05 to -0.03) |
| Venezuela (Bolivarian Republic of) | 157354 (148157 to 162114) | 363879.96 (342611.26 to 374886.74) | 99707 (81847 to 118932) | 277150.56 (227507.56 to 330591.43) | -36.64  (-48.54 to -24.04) | -1.08  (-1.18 to -0.98) |
| Viet Nam | 459377 (384139 to 509100) | 332851.96 (278336.81 to 368880.32) | 242911 (192275 to 297713) | 239263.25 (189387.97 to 293242.25) | -47.12  (-54.04 to -37.9) | -1.41  (-1.48 to -1.33) |
| Yemen | 189385 (183732 to 191456) | 374801.11 (363614 to 378898.85) | 260951 (248302 to 266728) | 370499.83 (352541.23 to 378702.59) | 37.79  (33.16 to 40.47) | -0.04  (-0.05 to -0.03) |
| Zambia | 102918 (92900 to 108464) | 358165.31 (323302.43 to 377467.9) | 164623 (147115 to 174077) | 356037.47 (318172.55 to 376486.01) | 59.96  (50.57 to 71.02) | -0.03  (-0.06 to -0.01) |
| Zimbabwe | 99635 (87077 to 107746) | 345485.37 (301941.46 to 373611.68) | 111882 (94128 to 124511) | 334406.73 (281341.62 to 372152.96) | 12.29  (-0.87 to 24.17) | -0.1  (-0.19 to 0) |

CI, confidence interval; EAPC, estimated annual percentage change; LMICs, low- and middle-income countries and territories; UI, uncertain interval.

## Supplementary Table S11. National burden and trends of incident cases and incidence rates (per 100,000 liveborn neonates) of fungal skin diseases in 131 LMICs between 1990 and 2019.

| **Location** | **Incident cases in 1990**  **(95% UI)** | **Incident rate in 1990**  **(95% UI)** | **Incident cases in 2019**  **(95% UI)** | **Incident rate in 2019**  **(95% UI)** | **Percent change**  **(%, 95% CI)** | **EAPC**  **(%, 95% CI)** |
| --- | --- | --- | --- | --- | --- | --- |
| Global | 1508083 (1114513 to 2043875) | 14606.75 (10794.77 to 19796.23) | 1515767 (1128251 to 2048663) | 14833.77 (11041.42 to 20048.86) | 0.51  (-1.38 to 2.53) | -0.04  (-0.11 to 0.03) |
| Afghanistan | 2347 (1726 to 3207) | 6066.93 (4460.12 to 8288.42) | 5958 (4439 to 7931) | 5327.54 (3969.23 to 7091.81) | 153.84  (136.41 to 172.55) | -0.43  (-0.45 to -0.41) |
| Albania | 391 (290 to 517) | 6007.13 (4462.37 to 7953.58) | 158 (117 to 210) | 5930.11 (4387.77 to 7897.05) | -59.54  (-59.94 to -59.28) | -0.04  (-0.04 to -0.04) |
| Algeria | 2859 (2157 to 3773) | 4922.48 (3714.49 to 6496.1) | 2810 (2144 to 3651) | 4237.48 (3233.54 to 5504.99) | -1.71  (-7.93 to 4.46) | -0.48  (-0.5 to -0.46) |
| American Samoa | 9 (7 to 11) | 6869.16 (5164.52 to 8778) | 5 (4 to 7) | 6823.85 (5134.73 to 8733.06) | -38.6  (-39.65 to -37.77) | -0.02  (-0.04 to 0) |
| Angola | 4813 (3508 to 6526) | 12700.82 (9257 to 17222.31) | 9762 (7202 to 13161) | 11708.21 (8638.32 to 15785.78) | 102.82  (79.98 to 130.33) | -0.33  (-0.37 to -0.29) |
| Armenia | 346 (258 to 458) | 6030.68 (4486.94 to 7973.74) | 171 (127 to 227) | 5984.62 (4439.07 to 7927.09) | -50.58  (-51.04 to -50.3) | -0.03  (-0.03 to -0.03) |
| Azerbaijan | 871 (649 to 1146) | 6087.25 (4534.28 to 8010.82) | 655 (487 to 867) | 6027.01 (4478.27 to 7975.02) | -24.76  (-25.46 to -24.29) | -0.04  (-0.05 to -0.03) |
| Bangladesh | 56347 (38912 to 82033) | 19249.5 (13293.33 to 28024.56) | 30890 (22150 to 43448) | 15204.49 (10902.61 to 21385.33) | -45.18  (-50.73 to -39.25) | -0.88  (-0.93 to -0.83) |
| Belarus | 643 (478 to 852) | 5996.99 (4453.51 to 7940.62) | 467 (346 to 622) | 5947.16 (4402.2 to 7911.8) | -27.34  (-27.88 to -27.02) | -0.04  (-0.04 to -0.03) |
| Belize | 40 (30 to 52) | 9068.19 (6824.16 to 11576.68) | 53 (40 to 67) | 9036.5 (6796.65 to 11519.99) | 30.07  (28.75 to 32.09) | -0.01  (-0.02 to -0.01) |
| Benin | 2778 (2038 to 3783) | 15614.71 (11457.23 to 21261.52) | 5717 (4278 to 7770) | 15117.44 (11311.17 to 20546.27) | 105.78  (80.5 to 121.26) | -0.11  (-0.14 to -0.08) |
| Bhutan | 311 (218 to 458) | 18920.42 (13273.68 to 27870.39) | 143 (103 to 201) | 14125.71 (10174.83 to 19788.84) | -53.96  (-59.16 to -48.58) | -1.13  (-1.18 to -1.08) |
| Bolivia (Plurinational State of) | 2519 (1928 to 3182) | 13793.85 (10558.01 to 17423.66) | 3236 (2451 to 4108) | 13264.9 (10047.59 to 16837.06) | 28.46  (24.87 to 30.88) | -0.14  (-0.14 to -0.14) |
| Bosnia and Herzegovina | 310 (230 to 411) | 5998.27 (4455.67 to 7955.17) | 122 (90 to 162) | 5943.08 (4392.72 to 7899.61) | -60.8  (-61.08 to -60.62) | -0.04  (-0.04 to -0.04) |
| Botswana | 261 (199 to 335) | 8475.48 (6488.88 to 10890.55) | 299 (229 to 385) | 8205.62 (6295.65 to 10565.72) | 14.65  (10.99 to 18.27) | -0.09  (-0.12 to -0.06) |
| Brazil | 25018 (18871 to 31764) | 9639.12 (7270.82 to 12238.4) | 22079 (16613 to 28179) | 9396.1 (7070.08 to 11991.95) | -11.75  (-13.43 to -10.64) | -0.08  (-0.09 to -0.08) |
| Bulgaria | 449 (333 to 596) | 5981.12 (4439.83 to 7942.14) | 272 (202 to 362) | 5951.28 (4408.33 to 7912.95) | -39.3  (-39.61 to -39) | -0.02  (-0.02 to -0.02) |
| Burkina Faso | 5156 (3816 to 7077) | 15040.74 (11131.38 to 20646.15) | 10175 (7559 to 13574) | 14425.31 (10716.2 to 19245.3) | 97.35  (82.69 to 116.61) | -0.16  (-0.22 to -0.1) |
| Burundi | 4340 (3135 to 6001) | 21938.11 (15849.21 to 30333.96) | 7052 (5123 to 10084) | 20107.69 (14607.69 to 28753.18) | 62.5  (45.12 to 81.72) | -0.44  (-0.53 to -0.36) |
| Cabo Verde | 122 (92 to 162) | 13716.04 (10288.65 to 18229.82) | 102 (78 to 133) | 12561.34 (9565.19 to 16293.81) | -16.17  (-22.45 to -8.89) | -0.39  (-0.45 to -0.34) |
| Cambodia | 3147 (2389 to 3997) | 9599.54 (7286.34 to 12191.62) | 2562 (1941 to 3268) | 9247.08 (7003.09 to 11791.46) | -18.57  (-20.89 to -17.13) | -0.14  (-0.15 to -0.13) |
| Cameroon | 4564 (3432 to 6000) | 13099.48 (9849.05 to 17218.04) | 8517 (6441 to 11299) | 12529.95 (9475.05 to 16622.31) | 86.6  (66.01 to 105.82) | -0.22  (-0.28 to -0.17) |
| Central African Republic | 1179 (868 to 1612) | 12537.35 (9229.57 to 17139.32) | 1951 (1412 to 2701) | 13239.13 (9582.03 to 18330.49) | 65.44  (49.37 to 85.64) | 0.1  (0.07 to 0.13) |
| Chad | 3652 (2713 to 4969) | 15406.35 (11444.6 to 20963.83) | 9222 (6795 to 12426) | 15588.18 (11485.83 to 21003.91) | 152.53  (133.09 to 176.35) | 0.03  (-0.02 to 0.07) |
| China | 105410 (79834 to 135081) | 5788.28 (4383.85 to 7417.55) | 60425 (45369 to 78479) | 5313.01 (3989.14 to 6900.42) | -42.68  (-45.6 to -40.49) | -0.34  (-0.36 to -0.32) |
| Colombia | 4602 (3544 to 5934) | 6523.77 (5024.72 to 8412.49) | 3758 (2855 to 4887) | 6239.63 (4741.13 to 8115.59) | -18.35  (-20.95 to -16.38) | -0.13  (-0.15 to -0.12) |
| Comoros | 331 (241 to 471) | 22500.83 (16359.69 to 32013.99) | 254 (185 to 352) | 20416.93 (14909.52 to 28318.63) | -23.33  (-30.58 to -15.99) | -0.46  (-0.51 to -0.42) |
| Congo | 954 (696 to 1282) | 12708.85 (9266.99 to 17072.89) | 1325 (968 to 1808) | 12201.39 (8909.02 to 16645.14) | 38.88  (23.41 to 52.02) | -0.24  (-0.33 to -0.16) |
| Costa Rica | 389 (297 to 506) | 6318.59 (4828.62 to 8218.27) | 314 (239 to 409) | 6210.87 (4728.69 to 8090.28) | -19.25  (-20.82 to -18.33) | -0.07  (-0.08 to -0.07) |
| Côte d'Ivoire | 6392 (4780 to 8710) | 15002.28 (11218.2 to 20442.46) | 9999 (7499 to 13528) | 15094.03 (11319.72 to 20421.52) | 56.43  (44.27 to 71.44) | -0.07  (-0.13 to -0.02) |
| Cuba | 1230 (921 to 1574) | 8913.46 (6677.36 to 11408.81) | 700 (521 to 896) | 8768.1 (6522.24 to 11224.59) | -43.08  (-43.87 to -42.53) | -0.06  (-0.07 to -0.05) |
| Democratic People's Republic of Korea | 2768 (2135 to 3576) | 5103.05 (3936.51 to 6592.6) | 1344 (1037 to 1735) | 5144.67 (3968.93 to 6643.15) | -51.45  (-52.17 to -50.47) | 0.03  (0 to 0.07) |
| Democratic Republic of the Congo | 17090 (12529 to 23538) | 12200.83 (8944.81 to 16804.12) | 25863 (18984 to 35152) | 11753.67 (8627.51 to 15975.05) | 51.33  (35.81 to 65.17) | -0.16  (-0.21 to -0.1) |
| Djibouti | 346 (251 to 494) | 20847.18 (15139.21 to 29786.35) | 511 (373 to 697) | 19573.52 (14300.85 to 26737.31) | 47.66  (33.23 to 66.06) | -0.36  (-0.45 to -0.28) |
| Dominica | 12 (9 to 15) | 9124.95 (6863.49 to 11623.16) | 6 (4 to 7) | 9176.43 (6895.29 to 11715.03) | -51.06  (-51.59 to -50.45) | 0.03  (0.02 to 0.04) |
| Dominican Republic | 1668 (1261 to 2127) | 9260.2 (6997.6 to 11805.53) | 1558 (1172 to 1989) | 9024.18 (6786.19 to 11519.94) | -6.61  (-8.94 to -5.19) | -0.05  (-0.07 to -0.03) |
| Ecuador | 3073 (2341 to 3887) | 13564.82 (10334.8 to 17161.27) | 3483 (2639 to 4430) | 13114.88 (9935.72 to 16679.94) | 13.34  (10.44 to 15.35) | -0.11  (-0.12 to -0.09) |
| Egypt | 9897 (7478 to 13208) | 6966.08 (5263.71 to 9296.9) | 7627 (5925 to 10082) | 4736.79 (3679.98 to 6261.52) | -22.94  (-32.38 to -13.09) | -1.05  (-1.15 to -0.95) |
| El Salvador | 823 (634 to 1053) | 6645.28 (5118.46 to 8501.08) | 540 (410 to 704) | 6315.67 (4798.09 to 8223.13) | -34.35  (-36.92 to -32.66) | -0.15  (-0.18 to -0.13) |
| Equatorial Guinea | 218 (159 to 296) | 12731.62 (9319.43 to 17314.27) | 334 (248 to 453) | 11436.67 (8499.19 to 15503.2) | 53.42  (36.63 to 68.79) | -0.48  (-0.56 to -0.4) |
| Eritrea | 2213 (1603 to 3111) | 23732.62 (17189.58 to 33366.6) | 3228 (2332 to 4547) | 21498.21 (15533.39 to 30287.38) | 45.85  (31.69 to 61.32) | -0.37  (-0.38 to -0.36) |
| Eswatini | 207 (159 to 268) | 8628.24 (6633.84 to 11151.23) | 192 (147 to 245) | 8660.34 (6637.23 to 11048.92) | -7.2  (-9.51 to -4.87) | 0.04  (-0.01 to 0.1) |
| Ethiopia | 61493 (44649 to 85499) | 33387.93 (24242.44 to 46422.15) | 82243 (61024 to 111404) | 29903.76 (22188.47 to 40506.69) | 33.74  (21.61 to 46.53) | -0.44  (-0.47 to -0.42) |
| Fiji | 100 (76 to 128) | 6966 (5280.49 to 8886.59) | 92 (70 to 118) | 6943.64 (5259.73 to 8879.76) | -7.99  (-9.95 to -6.45) | 0  (-0.01 to 0.01) |
| Gabon | 349 (261 to 472) | 11834.71 (8852.43 to 15997.6) | 373 (281 to 499) | 11592.82 (8718.05 to 15482.43) | 6.86  (-2.03 to 20.72) | -0.1  (-0.15 to -0.05) |
| Gambia | 534 (394 to 726) | 14828.14 (10945.66 to 20154.45) | 805 (599 to 1095) | 15009.44 (11167.54 to 20408.78) | 50.74  (38.87 to 64.3) | 0.07  (0.05 to 0.08) |
| Georgia | 381 (283 to 503) | 6022.64 (4480.11 to 7966.32) | 208 (155 to 276) | 6002.39 (4456.7 to 7944.2) | -45.24  (-45.59 to -45.03) | -0.01  (-0.01 to -0.01) |
| Ghana | 5048 (3637 to 7060) | 11169.27 (8046.93 to 15622.02) | 6876 (5001 to 9519) | 10703.57 (7784.6 to 14818.02) | 36.21  (23.18 to 47.78) | -0.07  (-0.1 to -0.05) |
| Grenada | 14 (10 to 17) | 9119.45 (6863.65 to 11602.57) | 10 (7 to 12) | 9020.67 (6779.41 to 11514.51) | -30.62  (-31.46 to -29.62) | -0.03  (-0.03 to -0.02) |
| Guatemala | 1730 (1332 to 2209) | 6808.29 (5241.56 to 8696.91) | 2032 (1550 to 2624) | 6493.99 (4954.54 to 8384.85) | 17.5  (13.5 to 20.5) | -0.17  (-0.2 to -0.14) |
| Guinea | 3776 (2785 to 5126) | 16119.76 (11887.76 to 21882.42) | 5649 (4216 to 7666) | 15708.76 (11723.69 to 21316.13) | 49.6  (35.52 to 63.88) | -0.09  (-0.12 to -0.07) |
| Guinea-Bissau | 575 (426 to 792) | 16621.58 (12293.62 to 22866.17) | 758 (560 to 1032) | 16203.16 (11959.15 to 22052) | 31.79  (19.82 to 44.54) | -0.12  (-0.17 to -0.08) |
| Guyana | 156 (118 to 200) | 9359.85 (7072.7 to 11953.49) | 100 (75 to 127) | 9216.01 (6939.8 to 11765.22) | -36.08  (-37.24 to -35.26) | -0.02  (-0.03 to -0.01) |
| Haiti | 1794 (1367 to 2289) | 9713.03 (7400.18 to 12394.63) | 2354 (1781 to 3004) | 9467.92 (7163.6 to 12083.79) | 31.19  (27.97 to 33.65) | -0.08  (-0.09 to -0.08) |
| Honduras | 909 (701 to 1164) | 6703.3 (5165.65 to 8585.8) | 1151 (877 to 1491) | 6453.82 (4920.5 to 8360.73) | 26.59  (22.44 to 29.34) | -0.11  (-0.12 to -0.11) |
| India | 642028 (423107 to 957669) | 32701 (21550.46 to 48777.79) | 532832 (350839 to 793937) | 30058.6 (19791.82 to 44788.26) | -17.01  (-19.5 to -14.9) | -0.3  (-0.33 to -0.28) |
| Indonesia | 35559 (26985 to 44899) | 9911.35 (7521.45 to 12514.66) | 28339 (21481 to 35885) | 9729.99 (7375.29 to 12320.84) | -20.3  (-21.44 to -19.49) | -0.06  (-0.07 to -0.06) |
| Iran (Islamic Republic of) | 6687 (5047 to 8771) | 4973.32 (3753.5 to 6522.75) | 4477 (3409 to 5753) | 4365.8 (3324.04 to 5610.18) | -33.05  (-36.13 to -30.1) | -0.32  (-0.37 to -0.26) |
| Iraq | 2206 (1719 to 2771) | 4174.47 (3252.73 to 5243.84) | 2701 (2128 to 3414) | 3712.07 (2924.38 to 4692.96) | 22.43  (15.24 to 28.37) | -0.49  (-0.54 to -0.44) |
| Jamaica | 383 (288 to 488) | 9013.85 (6784.19 to 11491.78) | 246 (185 to 314) | 8978.85 (6753.91 to 11484.93) | -35.78  (-36.47 to -35.08) | -0.02  (-0.03 to -0.01) |
| Jordan | 521 (392 to 686) | 5048.47 (3801.81 to 6649.23) | 783 (601 to 1008) | 4190.67 (3217.81 to 5392.71) | 50.28  (38.09 to 61.49) | -0.68  (-0.69 to -0.66) |
| Kazakhstan | 1628 (1212 to 2151) | 6045.9 (4499.73 to 7984.45) | 1610 (1196 to 2135) | 5989.47 (4450.14 to 7945.16) | -1.15  (-1.96 to -0.64) | -0.05  (-0.06 to -0.04) |
| Kenya | 12677 (9330 to 17566) | 16845.88 (12397.53 to 23342.48) | 17183 (12544 to 23729) | 16940.69 (12367.25 to 23394.82) | 35.54  (28.83 to 41.89) | -0.02  (-0.05 to 0.01) |
| Kiribati | 14 (11 to 18) | 7310.69 (5531.59 to 9328.03) | 16 (12 to 21) | 7132.76 (5429.34 to 9145.53) | 14.99  (12.13 to 17.34) | -0.1  (-0.11 to -0.09) |
| Kyrgyzstan | 576 (429 to 760) | 6056.43 (4509.78 to 7989.78) | 662 (492 to 878) | 5995.25 (4454.64 to 7944.67) | 14.94  (13.82 to 15.61) | -0.04  (-0.05 to -0.04) |
| Lao People's Democratic Republic | 1210 (932 to 1532) | 9779.05 (7529.24 to 12382.83) | 1221 (925 to 1554) | 9434.71 (7147.1 to 12016.27) | 0.87  (-2.39 to 3.28) | -0.13  (-0.13 to -0.12) |
| Lebanon | 389 (296 to 513) | 4689.63 (3563.4 to 6184.09) | 311 (239 to 401) | 3875.83 (2975.64 to 4992.9) | -20.11  (-26.59 to -13.44) | -0.66  (-0.7 to -0.62) |
| Lesotho | 389 (299 to 500) | 8581.48 (6592.96 to 11036) | 306 (235 to 390) | 8774.15 (6738.25 to 11196.95) | -21.38  (-23.19 to -19.05) | 0.1  (0.06 to 0.14) |
| Liberia | 970 (721 to 1337) | 16174.39 (12029.93 to 22301.15) | 1511 (1119 to 2023) | 14931.61 (11055.21 to 19990.11) | 55.84  (42.56 to 70.11) | -0.26  (-0.35 to -0.18) |
| Libya | 550 (411 to 724) | 4856.63 (3635.21 to 6397.41) | 280 (212 to 368) | 4511.99 (3409.7 to 5922.37) | -49.01  (-52.55 to -45.51) | -0.33  (-0.37 to -0.29) |
| Madagascar | 6543 (4959 to 8760) | 16397.32 (12427.79 to 21951.3) | 10348 (7765 to 14080) | 16046.55 (12040.49 to 21832.91) | 58.15  (47.8 to 70.5) | -0.06  (-0.11 to -0.01) |
| Malawi | 7049 (5089 to 9967) | 20271.8 (14635.43 to 28661.75) | 8203 (5983 to 11379) | 19865.72 (14490.99 to 27558.9) | 16.36  (6.54 to 28.89) | -0.17  (-0.21 to -0.12) |
| Malaysia | 3535 (2672 to 4506) | 9355.89 (7071.33 to 11925.24) | 3762 (2846 to 4804) | 9092.13 (6879.28 to 11610.3) | 6.41  (3.72 to 8.17) | -0.1  (-0.11 to -0.1) |
| Maldives | 61 (46 to 78) | 9280.43 (7025.92 to 11836.21) | 57 (43 to 73) | 8884.51 (6662.83 to 11421.4) | -7.29  (-10.18 to -5.27) | -0.17  (-0.18 to -0.16) |
| Mali | 8051 (5840 to 11296) | 24508.7 (17776.24 to 34385.28) | 16256 (11795 to 22440) | 22543.4 (16356.88 to 31119.89) | 101.9  (78.87 to 123.35) | -0.27  (-0.35 to -0.19) |
| Marshall Islands | 8 (6 to 10) | 7025.45 (5305.77 to 8983.32) | 7 (5 to 8) | 7013.67 (5311.7 to 8967.08) | -18.71  (-20.18 to -17.09) | -0.02  (-0.03 to 0) |
| Mauritania | 1082 (794 to 1463) | 16227.23 (11907.49 to 21944.47) | 1200 (895 to 1609) | 14542.9 (10847.55 to 19506.13) | 10.92  (0.58 to 25.98) | -0.39  (-0.41 to -0.37) |
| Mexico | 13002 (9908 to 16650) | 6926.09 (5277.76 to 8869.06) | 10797 (8227 to 13888) | 6776.41 (5163.41 to 8716.15) | -16.96  (-24.94 to -9.39) | -0.09  (-0.12 to -0.07) |
| Micronesia (Federated States of) | 19 (14 to 24) | 7126.23 (5392.62 to 9106.36) | 10 (8 to 13) | 6892.46 (5210.38 to 8812.54) | -45.56  (-47.17 to -44.4) | -0.12  (-0.12 to -0.11) |
| Mongolia | 338 (252 to 443) | 6133.35 (4580.8 to 8051.33) | 368 (275 to 486) | 6042.62 (4507.01 to 7982.93) | 8.97  (7.44 to 9.9) | -0.07  (-0.08 to -0.07) |
| Montenegro | 45 (34 to 60) | 5968.31 (4424.39 to 7925.2) | 30 (22 to 40) | 5935.92 (4390.28 to 7903.1) | -33.19  (-33.54 to -32.92) | -0.02  (-0.02 to -0.02) |
| Morocco | 3140 (2327 to 4175) | 5282.75 (3915.06 to 7023.3) | 2027 (1542 to 2644) | 4440.55 (3377.21 to 5792.9) | -35.45  (-39.74 to -30.68) | -0.54  (-0.56 to -0.52) |
| Mozambique | 8077 (5973 to 11255) | 18500.7 (13681.94 to 25779.24) | 14633 (10927 to 19960) | 17314.35 (12929.42 to 23618.61) | 81.16  (66.08 to 97.38) | -0.2  (-0.24 to -0.17) |
| Myanmar | 9568 (7318 to 12114) | 9641.79 (7374.37 to 12207.49) | 7372 (5580 to 9421) | 9352.49 (7079.12 to 11952.5) | -22.95  (-25.06 to -21.67) | -0.1  (-0.11 to -0.09) |
| Namibia | 323 (249 to 410) | 8856.18 (6816.61 to 11253.85) | 396 (304 to 509) | 8378.94 (6422.06 to 10774.87) | 22.69  (17.29 to 26.52) | -0.22  (-0.25 to -0.19) |
| Nepal | 8487 (6989 to 10228) | 13994.86 (11524.05 to 16865.05) | 4942 (3986 to 5975) | 10631.04 (8574.45 to 12853.54) | -41.78  (-46.5 to -36.38) | -0.89  (-0.96 to -0.83) |
| Nicaragua | 708 (540 to 914) | 6516.55 (4968.41 to 8415.8) | 622 (473 to 810) | 6246.64 (4742.69 to 8130.26) | -12.05  (-14.78 to -10.13) | -0.17  (-0.18 to -0.16) |
| Niger | 5337 (4022 to 7127) | 16057.61 (12101.74 to 21444.71) | 12933 (9870 to 16950) | 14974.47 (11427.8 to 19624.9) | 142.34  (122.75 to 164.19) | -0.34  (-0.38 to -0.3) |
| Nigeria | 77825 (57251 to 106131) | 25267.98 (18588.25 to 34458.49) | 135331 (99600 to 183808) | 23959.62 (17633.73 to 32542.3) | 73.89  (67.45 to 80.3) | -0.04  (-0.13 to 0.04) |
| North Macedonia | 160 (119 to 211) | 6012.53 (4475.54 to 7954.04) | 101 (75 to 134) | 5944.64 (4402.15 to 7907.39) | -37.01  (-37.59 to -36.65) | -0.04  (-0.04 to -0.04) |
| Pakistan | 71710 (49438 to 105486) | 20049.95 (13822.68 to 29493.36) | 94360 (66776 to 137224) | 18949.3 (13409.89 to 27557.27) | 31.58  (25.77 to 37.38) | -0.25  (-0.33 to -0.17) |
| Papua New Guinea | 790 (598 to 1011) | 7042.1 (5333.83 to 9012.42) | 1721 (1301 to 2204) | 6967.95 (5267.92 to 8922.38) | 117.97  (113.07 to 122.66) | -0.02  (-0.03 to -0.02) |
| Paraguay | 888 (668 to 1132) | 9172.55 (6903.17 to 11695.78) | 861 (647 to 1099) | 8961.65 (6741.84 to 11440.86) | -3.09  (-5 to -1.77) | -0.07  (-0.08 to -0.07) |
| Peru | 6712 (5050 to 8584) | 13438.13 (10110.38 to 17186.02) | 6296 (4669 to 8123) | 12964.37 (9614.87 to 16725.84) | -6.2  (-8.68 to -4.52) | -0.14  (-0.15 to -0.12) |
| Philippines | 15522 (11676 to 19726) | 9932.03 (7471 to 12622.34) | 19688 (14781 to 25109) | 9803.02 (7359.93 to 12502.4) | 26.84  (25.63 to 27.62) | -0.05  (-0.05 to -0.04) |
| Republic of Moldova | 354 (263 to 468) | 6033.14 (4490.45 to 7984.98) | 147 (109 to 194) | 5981.38 (4441.24 to 7932.61) | -58.55  (-58.89 to -58.35) | -0.04  (-0.04 to -0.03) |
| Russian Federation | 9284 (6795 to 12342) | 6306.91 (4615.91 to 8384.32) | 7966 (5822 to 10614) | 6247.88 (4566.32 to 8324.66) | -14.2  (-14.82 to -13.83) | -0.05  (-0.06 to -0.04) |
| Rwanda | 6687 (4644 to 9835) | 28116.91 (19528.51 to 41354.55) | 6148 (4383 to 8633) | 23102.12 (16470.75 to 32441.58) | -8.07  (-19.86 to 6) | -0.95  (-1.05 to -0.86) |
| Saint Lucia | 26 (20 to 33) | 9128.56 (6865.41 to 11629.13) | 12 (9 to 15) | 8977.82 (6744.11 to 11455.13) | -53.61  (-54.38 to -53.11) | -0.05  (-0.06 to -0.05) |
| Saint Vincent and the Grenadines | 18 (14 to 23) | 9074.76 (6826.82 to 11552.34) | 10 (8 to 13) | 9058.81 (6812.72 to 11550.23) | -42.81  (-43.42 to -42.24) | -0.01  (-0.01 to 0) |
| Samoa | 18 (13 to 23) | 6884.93 (5178 to 8789.56) | 20 (15 to 25) | 6716.11 (5048.85 to 8589.17) | 9.23  (6.72 to 11.26) | -0.07  (-0.09 to -0.05) |
| Senegal | 4010 (2961 to 5439) | 15631.39 (11540.45 to 21201.09) | 5253 (3913 to 7083) | 15043.15 (11205.17 to 20283.95) | 30.99  (19.88 to 42.24) | -0.17  (-0.19 to -0.15) |
| Serbia | 605 (449 to 802) | 5985.7 (4449.52 to 7938.72) | 374 (277 to 498) | 5936.68 (4396.33 to 7899.86) | -38.15  (-38.77 to -37.69) | -0.03  (-0.03 to -0.03) |
| Sierra Leone | 1834 (1354 to 2497) | 15669.2 (11574.17 to 21338.46) | 3151 (2312 to 4314) | 15028.31 (11025.2 to 20575.38) | 71.87  (55.49 to 90.46) | -0.16  (-0.19 to -0.13) |
| Solomon Islands | 74 (56 to 95) | 6908.02 (5217.61 to 8794.57) | 107 (80 to 137) | 6780.58 (5095.47 to 8697.94) | 43.82  (40.39 to 46.14) | -0.04  (-0.06 to -0.03) |
| Somalia | 4784 (3453 to 6805) | 20772.67 (14992.51 to 29547.38) | 13116 (9473 to 18626) | 20094.24 (14513.93 to 28535.93) | 174.16  (152.76 to 201.4) | -0.25  (-0.29 to -0.21) |
| South Africa | 6675 (5135 to 8447) | 9101.02 (7002.03 to 11516.87) | 6975 (5371 to 8814) | 8952.56 (6893.68 to 11313.36) | 4.49  (3.07 to 5.63) | -0.06  (-0.12 to -0.01) |
| South Sudan | 4066 (2986 to 5627) | 21696.25 (15935.25 to 30028.51) | 6054 (4413 to 8710) | 22344.5 (16285.95 to 32144.64) | 48.91  (37.98 to 65.89) | 0.01  (-0.03 to 0.05) |
| Sri Lanka | 2772 (2125 to 3535) | 10230.58 (7843.35 to 13047.62) | 2253 (1714 to 2873) | 9959.26 (7577.99 to 12702.67) | -18.73  (-20.65 to -17.49) | -0.09  (-0.1 to -0.08) |
| Sudan | 3897 (2915 to 5218) | 5363.61 (4012.48 to 7181.2) | 4187 (3159 to 5507) | 4725.43 (3565.69 to 6214.62) | 7.44  (0.32 to 14.3) | -0.36  (-0.39 to -0.34) |
| Suriname | 64 (48 to 81) | 9283.62 (6996.38 to 11877.74) | 62 (47 to 79) | 9149.98 (6924.63 to 11613.61) | -2.21  (-4.12 to -0.99) | -0.04  (-0.05 to -0.03) |
| Syrian Arab Republic | 1867 (1412 to 2455) | 4914.36 (3716.77 to 6461.95) | 747 (570 to 969) | 4213.88 (3216.69 to 5468.51) | -60  (-62.48 to -57.68) | -0.56  (-0.62 to -0.51) |
| Tajikistan | 938 (699 to 1238) | 6069.92 (4520.41 to 8010.44) | 1163 (866 to 1537) | 6039.42 (4495.66 to 7980.74) | 23.98  (22.94 to 24.53) | -0.02  (-0.02 to -0.02) |
| Thailand | 7467 (5656 to 9524) | 9287.92 (7034.58 to 11845.72) | 4007 (3023 to 5136) | 8985.26 (6778.98 to 11518.7) | -46.34  (-47.68 to -45.38) | -0.13  (-0.14 to -0.12) |
| Timor-Leste | 259 (197 to 329) | 9647.29 (7351.28 to 12272.51) | 272 (206 to 346) | 9374.03 (7112.39 to 11906.13) | 5.08  (2.31 to 7.21) | -0.09  (-0.1 to -0.08) |
| Togo | 1871 (1384 to 2526) | 15317.93 (11329.77 to 20675.33) | 2680 (1989 to 3668) | 15080.06 (11189.15 to 20633.55) | 43.25  (32.32 to 54.43) | -0.13  (-0.18 to -0.07) |
| Tonga | 14 (10 to 17) | 6869.74 (5211.94 to 8769.93) | 12 (9 to 15) | 6807.01 (5135.88 to 8713.5) | -10.84  (-12.29 to -9.64) | -0.03  (-0.04 to -0.02) |
| Tunisia | 838 (629 to 1114) | 4686.38 (3517.44 to 6227.06) | 513 (390 to 664) | 4036.74 (3071.82 to 5221.91) | -38.78  (-42.84 to -34.49) | -0.5  (-0.52 to -0.49) |
| Turkey | 7221 (5342 to 9747) | 5828.74 (4312.03 to 7867.14) | 3423 (2599 to 4486) | 4598.92 (3492.46 to 6026.62) | -52.6  (-56.77 to -48.44) | -0.98  (-1.06 to -0.9) |
| Turkmenistan | 600 (447 to 791) | 6073.14 (4526.02 to 8003.71) | 511 (380 to 676) | 6025.81 (4479.6 to 7970.44) | -14.81  (-15.5 to -14.36) | -0.03  (-0.04 to -0.03) |
| Tuvalu | 2 (1 to 2) | 7013.64 (5319.4 to 8967.03) | 1 (1 to 1) | 6838.89 (5171.66 to 8737.02) | -25.87  (-27.5 to -24.53) | -0.07  (-0.08 to -0.06) |
| Uganda | 13566 (9912 to 19020) | 19623.3 (14337.01 to 27512.71) | 23032 (16944 to 32566) | 19417.23 (14284.69 to 27454.51) | 69.78  (56.02 to 86.7) | -0.06  (-0.09 to -0.04) |
| Ukraine | 3154 (2308 to 4193) | 6289.38 (4603.07 to 8361.29) | 1856 (1358 to 2473) | 6249.6 (4573.44 to 8328.19) | -41.16  (-41.55 to -40.9) | -0.04  (-0.04 to -0.03) |
| United Republic of Tanzania | 22401 (15799 to 31493) | 24631.73 (17372.35 to 34628.49) | 47764 (32593 to 69894) | 30770.13 (20996.56 to 45026.54) | 113.22  (74.25 to 170.01) | 0.17  (-0.06 to 0.4) |
| Uzbekistan | 3255 (2421 to 4303) | 6037.41 (4490.63 to 7982.21) | 3260 (2421 to 4315) | 6008.98 (4463.5 to 7954.95) | 0.15  (-0.57 to 0.73) | -0.02  (-0.02 to -0.02) |
| Vanuatu | 30 (23 to 39) | 7055.89 (5343.27 to 8998.55) | 41 (31 to 53) | 7045.78 (5356.18 to 9022.88) | 35.24  (32.31 to 37.91) | 0.01  (0 to 0.03) |
| Venezuela (Bolivarian Republic of) | 2834 (2161 to 3642) | 6552.57 (4996.65 to 8421.34) | 2264 (1719 to 2933) | 6292.09 (4777.99 to 8152.29) | -20.11  (-22.86 to -18.22) | -0.15  (-0.17 to -0.13) |
| Viet Nam | 12876 (9766 to 16420) | 9329.95 (7076.28 to 11897.83) | 9176 (6909 to 11743) | 9038.01 (6804.8 to 11566.36) | -28.74  (-30.59 to -27.54) | -0.11  (-0.11 to -0.11) |
| Yemen | 2601 (1960 to 3472) | 5147.13 (3879.69 to 6871.67) | 3321 (2509 to 4361) | 4714.61 (3562.46 to 6192.34) | 27.68  (19.65 to 35.65) | -0.3  (-0.31 to -0.29) |
| Zambia | 6308 (4578 to 8919) | 21951.53 (15931.8 to 31040.74) | 9289 (6774 to 13298) | 20089.26 (14651.12 to 28759.18) | 47.26  (34.43 to 60.81) | -0.41  (-0.47 to -0.35) |
| Zimbabwe | 2412 (1852 to 3108) | 8364.23 (6421.52 to 10775.33) | 2901 (2226 to 3709) | 8670.94 (6652.48 to 11085.75) | 20.27  (16.45 to 25.23) | 0.15  (0.09 to 0.21) |

CI, confidence interval; EAPC, estimated annual percentage change; LMICs, low- and middle-income countries and territories; UI, uncertain interval

## Supplementary Table S12. National burden and trends of incident cases and incidence rates (per 100,000 liveborn neonates) of lower respiratory infections in 131 LMICs between 1990 and 2019.

| **Location** | **Incident cases in 1990**  **(95% UI)** | **Incident rate in 1990**  **(95% UI)** | **Incident cases in 2019**  **(95% UI)** | **Incident rate in 2019**  **(95% UI)** | **Percent change**  **(%, 95% CI)** | **EAPC**  **(%, 95% CI)** |
| --- | --- | --- | --- | --- | --- | --- |
| Global | 1751331 (1402260 to 2153072) | 16962.76 (13581.79 to 20853.88) | 735999 (567293 to 933180) | 7202.72 (5551.7 to 9132.39) | -57.97  (-60.04 to -56.17) | -3.09  (-3.15 to -3.04) |
| Afghanistan | 9401 (6965 to 12436) | 24297.25 (18001.68 to 32142.93) | 15633 (11715 to 20202) | 13978.41 (10474.9 to 18063.15) | 66.3  (44.89 to 90.22) | -2.03  (-2.2 to -1.87) |
| Albania | 2067 (1663 to 2564) | 31795.22 (25580.18 to 39439.04) | 230 (181 to 285) | 8634.73 (6774.92 to 10708.2) | -88.87  (-91.27 to -86.32) | -5.19  (-5.52 to -4.86) |
| Algeria | 11091 (8464 to 14300) | 19096.31 (14572.9 to 24620.85) | 5848 (4469 to 7655) | 8817.98 (6738.52 to 11542.61) | -47.27  (-55.61 to -38.53) | -3.05  (-3.21 to -2.89) |
| American Samoa | 15 (11 to 19) | 11320.54 (8748.19 to 14355.27) | 7 (5 to 9) | 8650.25 (6829.7 to 10894.19) | -52.77  (-59.09 to -44.54) | -0.63  (-0.78 to -0.49) |
| Angola | 6713 (4939 to 8874) | 17714.98 (13032.94 to 23418.08) | 6655 (5070 to 8580) | 7982.27 (6081.11 to 10291.3) | -0.86  (-15.27 to 19.53) | -2.89  (-3.19 to -2.58) |
| Armenia | 1240 (1040 to 1495) | 21593.78 (18115.03 to 26029.99) | 233 (190 to 283) | 8161 (6634.81 to 9885.07) | -81.18  (-84.89 to -77.21) | -3.84  (-4.06 to -3.62) |
| Azerbaijan | 3246 (2649 to 3989) | 22697.29 (18522.13 to 27887.93) | 1153 (896 to 1422) | 10609.5 (8240.12 to 13081.43) | -64.48  (-72.15 to -55.3) | -3.07  (-3.35 to -2.8) |
| Bangladesh | 29618 (21384 to 38784) | 10118.29 (7305.32 to 13249.76) | 11253 (8459 to 14615) | 5538.94 (4163.78 to 7193.44) | -62.01  (-67.32 to -54.99) | -2.22  (-2.41 to -2.02) |
| Belarus | 982 (764 to 1228) | 9161.65 (7127.32 to 11451.33) | 309 (240 to 394) | 3926.22 (3053.07 to 5017.44) | -68.6  (-74.13 to -61.51) | -3.14  (-3.49 to -2.79) |
| Belize | 51 (41 to 63) | 11361 (9191.17 to 14250.52) | 35 (27 to 43) | 5939.69 (4625.41 to 7448.91) | -31.76  (-42.94 to -21.66) | -2.54  (-2.75 to -2.33) |
| Benin | 2069 (1535 to 2735) | 11630.63 (8624.76 to 15370.34) | 2501 (1876 to 3218) | 6613.38 (4960.92 to 8508.82) | 20.86  (6.07 to 39.85) | -2.27  (-2.39 to -2.14) |
| Bhutan | 139 (102 to 186) | 8476.81 (6181.59 to 11342.27) | 50 (37 to 65) | 4940.36 (3695.77 to 6425.73) | -64.06  (-68.49 to -58.4) | -2.06  (-2.13 to -1.99) |
| Bolivia (Plurinational State of) | 4970 (3721 to 6351) | 27214.79 (20373.02 to 34775.05) | 4769 (3718 to 5955) | 19547.07 (15241.35 to 24408.85) | -4.05  (-19.06 to 12.16) | -1.11  (-1.21 to -1.02) |
| Bosnia and Herzegovina | 490 (389 to 629) | 9468.7 (7517.97 to 12163.02) | 218 (172 to 276) | 10651.61 (8419.6 to 13469.58) | -55.5  (-63.43 to -46) | 0.1  (-0.41 to 0.61) |
| Botswana | 327 (248 to 415) | 10641.53 (8071.51 to 13510.5) | 314 (239 to 399) | 8614.97 (6558.34 to 10956.73) | -4.14  (-15.96 to 8.61) | -0.92  (-1 to -0.83) |
| Brazil | 47585 (40264 to 56352) | 18334.12 (15513.23 to 21711.98) | 11170 (9084 to 13651) | 4753.59 (3865.7 to 5809.64) | -76.53  (-78.2 to -74.77) | -5.47  (-6.03 to -4.9) |
| Bulgaria | 2190 (1874 to 2592) | 29181.95 (24973.92 to 34549.33) | 539 (442 to 664) | 11772.44 (9659.12 to 14498.13) | -75.39  (-79.7 to -70.32) | -3.46  (-3.69 to -3.23) |
| Burkina Faso | 3801 (2817 to 5074) | 11088.36 (8217.69 to 14801.43) | 5256 (3877 to 6835) | 7452.28 (5497.27 to 9691.12) | 38.29  (22.88 to 56.79) | -1.52  (-1.65 to -1.39) |
| Burundi | 3278 (2426 to 4221) | 16572.86 (12263 to 21339.94) | 3679 (2742 to 4814) | 10491.01 (7818.71 to 13726.74) | 12.23  (-1.8 to 30.35) | -1.67  (-1.8 to -1.53) |
| Cabo Verde | 103 (76 to 135) | 11555.87 (8566 to 15175.44) | 43 (34 to 56) | 5324.16 (4124.93 to 6841.29) | -57.82  (-63.96 to -49.66) | -3.26  (-3.57 to -2.96) |
| Cambodia | 7834 (5964 to 10047) | 23898.72 (18192.59 to 30648.47) | 2763 (2170 to 3480) | 9971.78 (7831.34 to 12556.82) | -64.73  (-70.62 to -57.58) | -3.42  (-3.79 to -3.05) |
| Cameroon | 3650 (2710 to 4830) | 10473.92 (7777.88 to 13860.96) | 4674 (3487 to 6103) | 6875.49 (5129.21 to 8977.85) | 28.06  (11.77 to 49.3) | -1.62  (-1.79 to -1.44) |
| Central African Republic | 1733 (1279 to 2269) | 18421.23 (13601.28 to 24127.62) | 1840 (1350 to 2385) | 12486.26 (9157.95 to 16182.09) | 6.2  (-6.03 to 18.67) | -1.38  (-1.43 to -1.33) |
| Chad | 2953 (2157 to 3911) | 12459.69 (9101.04 to 16501.22) | 5304 (3905 to 7070) | 8965.92 (6600.11 to 11950.57) | 79.6  (58.93 to 105.97) | -1.27  (-1.45 to -1.1) |
| China | 537840 (450214 to 642876) | 29533.84 (24722.1 to 35301.54) | 54240 (42102 to 68700) | 4769.14 (3701.94 to 6040.59) | -89.92  (-91.07 to -88.69) | -6.76  (-6.96 to -6.56) |
| Colombia | 10608 (8574 to 13041) | 15038.36 (12155.47 to 18487.17) | 5992 (4876 to 7288) | 9949.87 (8096.51 to 12102.53) | -43.52  (-52.72 to -31.5) | -2.01  (-2.19 to -1.84) |
| Comoros | 261 (192 to 348) | 17730.65 (13037.67 to 23662.33) | 122 (92 to 157) | 9777.92 (7397.15 to 12595.24) | -53.41  (-59.57 to -44.13) | -2.07  (-2.15 to -1.99) |
| Congo | 1204 (893 to 1545) | 16040.51 (11891.49 to 20575.67) | 823 (622 to 1060) | 7578.7 (5728.11 to 9760.23) | -31.65  (-40.69 to -21.39) | -2.86  (-2.99 to -2.73) |
| Costa Rica | 1087 (869 to 1330) | 17650.16 (14103.89 to 21601.23) | 438 (347 to 546) | 8655.45 (6856.1 to 10792.72) | -59.72  (-67.1 to -51.81) | -3.11  (-3.38 to -2.84) |
| Côte d'Ivoire | 4754 (3468 to 6402) | 11158.74 (8140.19 to 15024.79) | 4983 (3696 to 6511) | 7522.76 (5579.98 to 9828.55) | 4.82  (-10.41 to 20.34) | -1.44  (-1.52 to -1.36) |
| Cuba | 1112 (843 to 1404) | 8059.12 (6109.75 to 10174.41) | 390 (310 to 492) | 4890.66 (3887.71 to 6156.67) | -64.89  (-71.05 to -57.58) | -1.84  (-2.03 to -1.65) |
| Democratic People's Republic of Korea | 8851 (6579 to 11421) | 16316.77 (12128.77 to 21054.46) | 2226 (1709 to 2874) | 8522.06 (6541.73 to 11001.84) | -74.85  (-78.37 to -70.89) | -2.39  (-2.49 to -2.28) |
| Democratic Republic of the Congo | 27362 (20281 to 35650) | 19534.03 (14479.13 to 25451.12) | 22187 (16569 to 28715) | 10083.27 (7530.05 to 13050.06) | -18.91  (-29.93 to -6.02) | -2.42  (-2.56 to -2.28) |
| Djibouti | 279 (205 to 359) | 16806.28 (12358.05 to 21670.34) | 249 (187 to 322) | 9556.04 (7170.63 to 12358.84) | -10.58  (-22.2 to 3.77) | -2.08  (-2.22 to -1.93) |
| Dominica | 11 (9 to 14) | 8572.75 (6604.52 to 10869.99) | 5 (4 to 6) | 7600.34 (5817.49 to 9708.16) | -56.86  (-61.93 to -51.17) | -0.11  (-0.27 to 0.04) |
| Dominican Republic | 2886 (2266 to 3615) | 16020.82 (12577.6 to 20067.99) | 1250 (957 to 1610) | 7240.67 (5543.67 to 9325.03) | -56.69  (-62.8 to -49.58) | -3.2  (-3.39 to -3.02) |
| Ecuador | 5002 (4080 to 6232) | 22081.11 (18011.56 to 27509.77) | 3261 (2656 to 3921) | 12279.42 (10000.5 to 14763.11) | -34.81  (-44.87 to -23.71) | -2.24  (-2.36 to -2.12) |
| Egypt | 29307 (21877 to 38399) | 20629.21 (15398.72 to 27028.57) | 21776 (16371 to 28000) | 13525.1 (10168.03 to 17390.5) | -25.7  (-36.67 to -11.48) | -1.44  (-1.65 to -1.23) |
| El Salvador | 2872 (2303 to 3523) | 23191.81 (18597.24 to 28446.62) | 1006 (805 to 1253) | 11758.96 (9404.61 to 14642.39) | -64.98  (-70.63 to -58.56) | -2.83  (-3.03 to -2.64) |
| Equatorial Guinea | 332 (244 to 437) | 19429.92 (14264.08 to 25538.82) | 203 (154 to 266) | 6964.69 (5262.54 to 9124.1) | -38.78  (-47.44 to -28.66) | -4.07  (-4.29 to -3.86) |
| Eritrea | 1809 (1341 to 2374) | 19403.78 (14378.65 to 25463.32) | 1648 (1255 to 2110) | 10979.32 (8356.3 to 14050.26) | -8.9  (-21.82 to 5.87) | -2.02  (-2.1 to -1.95) |
| Eswatini | 395 (301 to 501) | 16439.25 (12530.57 to 20852.01) | 269 (204 to 347) | 12120.06 (9167.59 to 15630.68) | -31.84  (-40.35 to -21.26) | -0.8  (-1.08 to -0.52) |
| Ethiopia | 37570 (28002 to 48542) | 20399.01 (15204.03 to 26356.02) | 25605 (19230 to 33131) | 9310.21 (6992.21 to 12046.43) | -31.85  (-36.91 to -26.13) | -2.82  (-3.03 to -2.61) |
| Fiji | 180 (141 to 229) | 12515.18 (9796.54 to 15921.18) | 133 (104 to 169) | 10007.04 (7818.99 to 12736.72) | -26.19  (-35.28 to -15.29) | -0.54  (-0.68 to -0.39) |
| Gabon | 453 (337 to 592) | 15340.73 (11412.53 to 20051.89) | 240 (183 to 312) | 7443.15 (5673.47 to 9686.57) | -47.07  (-53.99 to -39) | -2.59  (-2.68 to -2.49) |
| Gambia | 445 (336 to 589) | 12353.4 (9327.24 to 16339.18) | 322 (244 to 416) | 6008.87 (4546.3 to 7754.13) | -27.56  (-37.71 to -13.25) | -2.79  (-2.97 to -2.62) |
| Georgia | 1609 (1328 to 1976) | 25463.28 (21019.36 to 31272.02) | 285 (229 to 354) | 8204.87 (6598.54 to 10190.54) | -82.29  (-86.19 to -78.03) | -4.9  (-5.35 to -4.45) |
| Ghana | 4347 (3221 to 5669) | 9618.73 (7127.82 to 12544.66) | 3808 (2849 to 4844) | 5928.5 (4435.6 to 7540.58) | -12.39  (-23.34 to 0.25) | -1.79  (-1.91 to -1.67) |
| Grenada | 17 (13 to 21) | 11162.97 (8929.29 to 13852.01) | 6 (5 to 8) | 5873.91 (4683.79 to 7476.29) | -63.09  (-68.17 to -56.82) | -2.42  (-2.55 to -2.29) |
| Guatemala | 4910 (3970 to 6002) | 19326.39 (15629.45 to 23628.32) | 6123 (5006 to 7324) | 19567.38 (15996.8 to 23405.63) | 24.72  (-1.07 to 51.88) | 0  (-0.27 to 0.28) |
| Guinea | 3088 (2247 to 4135) | 13182.98 (9591.24 to 17653.56) | 2915 (2158 to 3806) | 8105.33 (5999.55 to 10583.81) | -5.61  (-16.24 to 9.03) | -1.81  (-1.93 to -1.7) |
| Guinea-Bissau | 366 (274 to 484) | 10564.83 (7916.38 to 13988.02) | 281 (212 to 366) | 6002.69 (4539.25 to 7816.9) | -23.19  (-32.96 to -12.4) | -2.27  (-2.44 to -2.1) |
| Guyana | 155 (121 to 197) | 9282.41 (7244.13 to 11803.22) | 68 (54 to 86) | 6275.86 (4940.85 to 7923.25) | -56.11  (-62.09 to -49.56) | -1.51  (-1.83 to -1.2) |
| Haiti | 2921 (2186 to 3783) | 15816.96 (11835.24 to 20481.53) | 2657 (1939 to 3428) | 10687.49 (7801.71 to 13790.83) | -9.06  (-20.59 to 4.37) | -1.55  (-1.68 to -1.43) |
| Honduras | 2399 (1783 to 3105) | 17689.02 (13143.17 to 22893.52) | 1925 (1452 to 2495) | 10792.8 (8141.6 to 13990.01) | -19.77  (-30.59 to -5.82) | -2.07  (-2.21 to -1.94) |
| India | 178284 (133766 to 232259) | 9080.69 (6813.23 to 11829.85) | 92559 (69155 to 121058) | 5221.53 (3901.25 to 6829.22) | -48.08  (-49.87 to -46.15) | -1.91  (-2.47 to -1.35) |
| Indonesia | 81023 (62323 to 101006) | 22583.38 (17371.07 to 28153.39) | 25394 (19540 to 32160) | 8718.79 (6708.81 to 11041.89) | -68.66  (-70.38 to -66.93) | -2.29  (-2.63 to -1.94) |
| Iran (Islamic Republic of) | 29650 (22867 to 37539) | 22050.71 (17006.19 to 27917.46) | 5274 (4035 to 6810) | 5142.89 (3934.17 to 6640.64) | -82.21  (-83.36 to -81.03) | -5.22  (-5.32 to -5.12) |
| Iraq | 10499 (7787 to 13732) | 19868.91 (14736.47 to 25987.93) | 6114 (4692 to 7760) | 8403.94 (6448.84 to 10666.95) | -41.76  (-51.24 to -31.7) | -3.27  (-3.44 to -3.1) |
| Jamaica | 339 (265 to 436) | 7976.82 (6237.91 to 10266.19) | 115 (91 to 143) | 4187.48 (3310.53 to 5214.77) | -66.16  (-70.67 to -61.26) | -2.57  (-2.8 to -2.33) |
| Jordan | 1776 (1371 to 2277) | 17202.56 (13284.8 to 22060.69) | 1771 (1386 to 2209) | 9475.62 (7418.7 to 11821.67) | -0.27  (-15.74 to 20.87) | -1.93  (-2.15 to -1.71) |
| Kazakhstan | 6434 (5576 to 7671) | 23887.65 (20700.82 to 28482.36) | 1487 (1204 to 1824) | 5534.16 (4478.8 to 6787.84) | -76.88  (-81.78 to -71.85) | -5.68  (-6.27 to -5.09) |
| Kenya | 13274 (10055 to 16964) | 17639.68 (13361.04 to 22543.09) | 6919 (5188 to 8984) | 6821.18 (5115.34 to 8856.88) | -47.88  (-51.02 to -44.44) | -3.14  (-3.26 to -3.01) |
| Kiribati | 30 (24 to 39) | 15501.68 (12254.79 to 19973.44) | 23 (17 to 29) | 9905.75 (7619.46 to 12626.38) | -24.69  (-34.57 to -12.8) | -1.5  (-1.64 to -1.36) |
| Kyrgyzstan | 1253 (1030 to 1494) | 13172.04 (10822.28 to 15700.36) | 1058 (896 to 1271) | 9576.39 (8107.75 to 11501.93) | -15.58  (-29.96 to 4.17) | -1.79  (-2.43 to -1.14) |
| Lao People's Democratic Republic | 2931 (2168 to 3790) | 23689.58 (17517.72 to 30629.11) | 1522 (1179 to 1957) | 11767.77 (9111.92 to 15125.61) | -48.06  (-56.24 to -38.39) | -2.52  (-2.87 to -2.16) |
| Lebanon | 1281 (981 to 1646) | 15428.23 (11819.18 to 19823.87) | 667 (513 to 871) | 8306.97 (6387.5 to 10856.08) | -47.96  (-54.78 to -40.34) | -2.39  (-2.49 to -2.29) |
| Lesotho | 679 (512 to 875) | 14999.37 (11310.74 to 19320.3) | 392 (299 to 505) | 11241.69 (8575.77 to 14502.36) | -42.37  (-49.53 to -34.11) | -0.71  (-0.8 to -0.61) |
| Liberia | 878 (634 to 1148) | 14645.71 (10570.92 to 19151.36) | 695 (516 to 908) | 6864.01 (5099.77 to 8969.18) | -20.89  (-32.29 to -7.61) | -3.05  (-3.24 to -2.86) |
| Libya | 1858 (1410 to 2419) | 16424.79 (12456.77 to 21376.23) | 465 (355 to 595) | 7480.25 (5713 to 9587.79) | -75  (-78.16 to -71.26) | -2.97  (-3.36 to -2.58) |
| Madagascar | 7353 (5486 to 9293) | 18425.49 (13747.36 to 23288.3) | 6346 (4816 to 8195) | 9840.98 (7467.62 to 12708.02) | -13.69  (-27.04 to 1.49) | -2.29  (-2.41 to -2.18) |
| Malawi | 6485 (4900 to 8308) | 18648.39 (14090.66 to 23890.93) | 4240 (3264 to 5455) | 10268.83 (7905.81 to 13211.47) | -34.62  (-43.32 to -25.85) | -2.32  (-2.47 to -2.17) |
| Malaysia | 4036 (3099 to 5134) | 10681.62 (8200.36 to 13588.55) | 2320 (1804 to 2882) | 5608.39 (4360.12 to 6965.21) | -42.51  (-50.08 to -32.42) | -2.17  (-2.66 to -1.67) |
| Maldives | 75 (58 to 96) | 11354.37 (8703.91 to 14504.6) | 33 (26 to 42) | 5199.54 (4062.09 to 6590.54) | -55.65  (-61.58 to -48.43) | -2.76  (-3.04 to -2.47) |
| Mali | 3272 (2424 to 4306) | 9958.86 (7379.78 to 13106.15) | 5247 (3877 to 6779) | 7276.09 (5376.95 to 9401.42) | 60.37  (41.08 to 80.33) | -1.35  (-1.51 to -1.19) |
| Marshall Islands | 18 (14 to 23) | 15658.65 (11909.14 to 20298.45) | 10 (7 to 13) | 10496.31 (7971.68 to 13418.44) | -45.42  (-51.77 to -37.76) | -1.31  (-1.51 to -1.12) |
| Mauritania | 631 (468 to 826) | 9457.5 (7023.85 to 12392.67) | 542 (400 to 702) | 6569.63 (4845.85 to 8508.66) | -14.03  (-24.69 to -2.91) | -1.46  (-1.69 to -1.23) |
| Mexico | 45400 (37236 to 55537) | 24183.55 (19834.8 to 29583.25) | 13574 (10867 to 16956) | 8519.13 (6820.22 to 10641.47) | -70.1  (-72.06 to -67.8) | -4.27  (-4.5 to -4.05) |
| Micronesia (Federated States of) | 45 (35 to 57) | 16970.82 (13183.02 to 21718.96) | 14 (11 to 18) | 9447.35 (7309.44 to 12071.12) | -68.66  (-72.82 to -63.58) | -2.07  (-2.19 to -1.96) |
| Mongolia | 1537 (1205 to 1926) | 27898.09 (21871.28 to 34970.94) | 600 (481 to 743) | 9852.45 (7891.61 to 12191.45) | -60.94  (-67.23 to -53.63) | -4.15  (-4.41 to -3.9) |
| Montenegro | 114 (89 to 145) | 15046.1 (11676.62 to 19104.75) | 36 (28 to 45) | 7073.23 (5568.95 to 8833.03) | -68.42  (-72.63 to -63) | -2.73  (-3.06 to -2.41) |
| Morocco | 11425 (8327 to 14829) | 19219.1 (14006.89 to 24944.96) | 4245 (3254 to 5471) | 9298.58 (7128.83 to 11985.03) | -62.85  (-68.28 to -56.95) | -2.8  (-2.92 to -2.68) |
| Mozambique | 8393 (6172 to 11069) | 19223.26 (14137.72 to 25354.33) | 7517 (5712 to 9732) | 8894.2 (6758.45 to 11516) | -10.44  (-23.08 to 6.21) | -2.98  (-3.15 to -2.81) |
| Myanmar | 18449 (13589 to 23939) | 18590.76 (13693.72 to 24123.8) | 6142 (4787 to 7732) | 7792.8 (6073.13 to 9809.75) | -66.7  (-71.43 to -60.12) | -3.22  (-3.45 to -2.98) |
| Namibia | 536 (402 to 690) | 14691.91 (11039.42 to 18914.74) | 486 (368 to 626) | 10287.18 (7784.59 to 13245.42) | -9.2  (-20.29 to 4.3) | -1.15  (-1.26 to -1.03) |
| Nepal | 5837 (4273 to 7743) | 9624.43 (7046 to 12767.85) | 2516 (1885 to 3248) | 5413.31 (4055.84 to 6987.82) | -56.89  (-62.16 to -50.09) | -2.16  (-2.23 to -2.1) |
| Nicaragua | 2652 (2038 to 3314) | 24416 (18762.5 to 30514.36) | 1873 (1507 to 2285) | 18796.07 (15127.33 to 22934.57) | -29.36  (-41.77 to -15.96) | -0.94  (-1.1 to -0.77) |
| Niger | 4546 (3344 to 6130) | 13678.43 (10062.55 to 18443.06) | 6689 (4920 to 8863) | 7744.76 (5696.7 to 10261.92) | 47.14  (27.64 to 69.35) | -2.3  (-2.45 to -2.14) |
| Nigeria | 38186 (28477 to 50007) | 12398.2 (9245.97 to 16236.07) | 33322 (24839 to 43027) | 5899.41 (4397.62 to 7617.73) | -12.74  (-16.28 to -9.13) | -3.68  (-4.14 to -3.21) |
| North Macedonia | 660 (547 to 789) | 24835.76 (20567.39 to 29696.64) | 175 (139 to 218) | 10323.03 (8234.98 to 12898.73) | -73.52  (-77.63 to -69.29) | -3.17  (-3.52 to -2.81) |
| Pakistan | 27827 (20616 to 35960) | 7780.4 (5764.05 to 10054.24) | 30343 (22314 to 39214) | 6093.54 (4481.06 to 7874.9) | 9.04  (-0.34 to 18.77) | -0.73  (-0.83 to -0.62) |
| Papua New Guinea | 2223 (1691 to 2877) | 19822.26 (15079.56 to 25652.14) | 3560 (2682 to 4559) | 14412.72 (10858.75 to 18454.97) | 60.17  (37.73 to 84.33) | -0.94  (-1.02 to -0.85) |
| Paraguay | 881 (690 to 1092) | 9097.1 (7129.05 to 11279.32) | 438 (344 to 545) | 4560.65 (3586.24 to 5671.96) | -50.27  (-57.17 to -41.25) | -2.41  (-2.69 to -2.13) |
| Peru | 12079 (9247 to 15207) | 24181.89 (18513.14 to 30444.75) | 5570 (4396 to 6899) | 11469.02 (9051.17 to 14205.55) | -53.89  (-61.38 to -45.14) | -3  (-3.17 to -2.83) |
| Philippines | 30278 (24332 to 37024) | 19374.1 (15569.41 to 23690.63) | 16732 (13458 to 20619) | 8331.14 (6701.06 to 10266.82) | -44.74  (-47.37 to -41.91) | -2.95  (-3.12 to -2.77) |
| Republic of Moldova | 615 (464 to 803) | 10491.17 (7913.29 to 13704.61) | 73 (56 to 92) | 2975.37 (2305.73 to 3742.86) | -88.14  (-90.6 to -85.38) | -5.02  (-5.72 to -4.32) |
| Russian Federation | 12349 (9427 to 15811) | 8389.11 (6404.26 to 10741.12) | 6358 (5005 to 7908) | 4987.03 (3925.78 to 6202.5) | -48.51  (-52.61 to -43.22) | -2.22  (-2.69 to -1.75) |
| Rwanda | 4815 (3558 to 6378) | 20243.9 (14960.9 to 26817.78) | 2983 (2245 to 3876) | 11208.93 (8436.01 to 14564.3) | -38.05  (-46.17 to -27.61) | -2.31  (-2.56 to -2.06) |
| Saint Lucia | 23 (18 to 30) | 8166.93 (6444.36 to 10393.61) | 7 (5 to 9) | 5111 (4039.91 to 6356.08) | -70.48  (-74 to -66.2) | -1.38  (-1.61 to -1.16) |
| Saint Vincent and the Grenadines | 17 (14 to 22) | 8632.22 (6813.23 to 11063.77) | 8 (6 to 10) | 6533.5 (5206.05 to 8441.49) | -56.64  (-62.98 to -49.8) | -1.32  (-1.47 to -1.17) |
| Samoa | 39 (30 to 50) | 14998.92 (11596.44 to 19169.03) | 26 (20 to 32) | 8764.1 (6839.83 to 11076.21) | -34.57  (-42.46 to -23.68) | -2.08  (-2.28 to -1.88) |
| Senegal | 2793 (2119 to 3616) | 10886.78 (8260.55 to 14095.22) | 2323 (1736 to 3029) | 6652.38 (4972.03 to 8674.29) | -16.83  (-27.23 to -4.66) | -1.78  (-1.92 to -1.65) |
| Serbia | 1498 (1193 to 1878) | 14834.19 (11809.44 to 18599.22) | 351 (277 to 430) | 5580.55 (4399.91 to 6825.26) | -76.54  (-79.98 to -71.99) | -3.33  (-3.49 to -3.17) |
| Sierra Leone | 1666 (1190 to 2192) | 14237.57 (10166.18 to 18733.03) | 1810 (1319 to 2347) | 8634.11 (6292.45 to 11193.88) | 8.67  (-7.05 to 30.03) | -1.89  (-2.12 to -1.66) |
| Solomon Islands | 183 (141 to 232) | 17027.86 (13060.97 to 21519.28) | 167 (129 to 213) | 10563.46 (8179.54 to 13523.77) | -9.1  (-19.57 to 5.62) | -1.6  (-1.73 to -1.47) |
| Somalia | 4505 (3295 to 5849) | 19561.78 (14308.69 to 25397.51) | 8559 (6338 to 11420) | 13113.07 (9710.98 to 17496.05) | 89.99  (66.7 to 117.99) | -1.55  (-1.65 to -1.44) |
| South Africa | 14596 (11394 to 18198) | 19900.96 (15535.91 to 24811.87) | 9674 (7704 to 12051) | 12417.13 (9888.59 to 15468.07) | -33.72  (-37.38 to -29.66) | -1.47  (-1.76 to -1.18) |
| South Sudan | 3347 (2462 to 4406) | 17863.94 (13139.82 to 23515.45) | 3299 (2420 to 4310) | 12173.97 (8932.95 to 15907.59) | -1.46  (-13.32 to 12.8) | -1.25  (-1.31 to -1.19) |
| Sri Lanka | 2303 (1858 to 2833) | 8501.47 (6856.73 to 10456.35) | 1136 (887 to 1427) | 5022.98 (3921.83 to 6308.47) | -50.67  (-56.96 to -43.61) | -1.48  (-1.62 to -1.35) |
| Sudan | 14787 (11110 to 18991) | 20350.74 (15290.29 to 26137.97) | 9058 (6888 to 11626) | 10222.16 (7774.18 to 13120.45) | -38.74  (-47 to -30.14) | -2.57  (-2.64 to -2.51) |
| Suriname | 69 (53 to 87) | 10009.03 (7770.72 to 12703.47) | 43 (33 to 55) | 6375.03 (4909.54 to 8126.11) | -36.8  (-45.67 to -25.24) | -1.93  (-2.15 to -1.71) |
| Syrian Arab Republic | 6971 (5387 to 8879) | 18349.03 (14180.03 to 23370.41) | 1262 (965 to 1597) | 7120.28 (5441.65 to 9011.6) | -81.9  (-84.5 to -78.91) | -3.77  (-4.65 to -2.89) |
| Tajikistan | 2920 (2327 to 3572) | 18896.52 (15061.43 to 23115.64) | 2206 (1738 to 2785) | 11458.46 (9025.09 to 14464.82) | -24.44  (-36.88 to -11.37) | -1.99  (-2.18 to -1.8) |
| Thailand | 9671 (7540 to 12238) | 12028.19 (9378.3 to 15221.37) | 2460 (1939 to 3030) | 5517.18 (4348.67 to 6795.83) | -74.56  (-78.29 to -69.95) | -2.93  (-3.04 to -2.81) |
| Timor-Leste | 732 (559 to 931) | 27290.51 (20836.06 to 34691.97) | 285 (224 to 361) | 9808.86 (7726.21 to 12436.74) | -61.13  (-67.64 to -53.55) | -4.09  (-4.34 to -3.85) |
| Togo | 1376 (1022 to 1825) | 11260.4 (8362.26 to 14940.28) | 1141 (856 to 1460) | 6419.86 (4813.16 to 8212.61) | -17.04  (-26.64 to -5.28) | -1.95  (-2.05 to -1.85) |
| Tonga | 26 (20 to 33) | 13200 (10128.58 to 16789.95) | 15 (12 to 19) | 8554.09 (6628.55 to 10835.18) | -41.69  (-47.67 to -32.99) | -1.3  (-1.53 to -1.08) |
| Tunisia | 3594 (2707 to 4595) | 20094.92 (15135.17 to 25691.71) | 1119 (849 to 1441) | 8800.48 (6682.05 to 11332.41) | -68.87  (-73.25 to -64.28) | -3.2  (-3.42 to -2.97) |
| Turkey | 26985 (20041 to 35042) | 21781.36 (16176.2 to 28285.17) | 4518 (3519 to 5752) | 6070.19 (4728.52 to 7727.66) | -83.26  (-86.3 to -79.44) | -5.34  (-5.68 to -4.99) |
| Turkmenistan | 1689 (1374 to 2063) | 17084.93 (13902.14 to 20864.6) | 1005 (840 to 1219) | 11845.55 (9900.37 to 14365.97) | -40.47  (-50.36 to -27.68) | -1.18  (-1.6 to -0.76) |
| Tuvalu | 4 (3 to 5) | 19605.45 (14880.48 to 24662.94) | 1 (1 to 2) | 8757.46 (6842.46 to 11141.18) | -66.04  (-70.12 to -60.06) | -2.95  (-3.09 to -2.81) |
| Uganda | 12571 (9495 to 16289) | 18183.98 (13734.27 to 23561.31) | 12300 (9297 to 15614) | 10369.32 (7837.7 to 13163.58) | -2.16  (-16.02 to 14.72) | -1.91  (-2.05 to -1.76) |
| Ukraine | 2303 (1725 to 2969) | 4593.53 (3440.8 to 5921.91) | 1426 (1107 to 1798) | 4801.39 (3728.91 to 6057.38) | -38.11  (-46.47 to -27.66) | 0.21  (-0.45 to 0.87) |
| United Republic of Tanzania | 18962 (14172 to 24451) | 20849.82 (15582.59 to 26885.07) | 18428 (13835 to 23747) | 11871.51 (8912.92 to 15297.79) | -2.82  (-16.88 to 15.21) | -1.95  (-2.06 to -1.84) |
| Uzbekistan | 10754 (9068 to 12909) | 19949.1 (16820.86 to 23946.01) | 5038 (4132 to 6120) | 9287.56 (7617.06 to 11282.21) | -53.15  (-61.05 to -42.49) | -2.97  (-3.54 to -2.39) |
| Vanuatu | 60 (46 to 77) | 13954.48 (10750.1 to 17916.07) | 61 (48 to 77) | 10514.09 (8180.48 to 13123.72) | 2.05  (-9.8 to 15.94) | -0.85  (-0.97 to -0.72) |
| Venezuela (Bolivarian Republic of) | 6887 (5620 to 8406) | 15925.42 (12996.1 to 19438.05) | 2973 (2344 to 3710) | 8263.52 (6514.91 to 10313.68) | -56.83  (-64.9 to -47.07) | -3.04  (-3.49 to -2.58) |
| Viet Nam | 18863 (14460 to 24359) | 13667.5 (10477.26 to 17649.85) | 7240 (5663 to 9067) | 7130.88 (5577.63 to 8930.55) | -61.62  (-65.99 to -56.41) | -2.15  (-2.18 to -2.11) |
| Yemen | 13667 (10089 to 17566) | 27047.15 (19966.46 to 34764) | 8860 (6687 to 11269) | 12578.82 (9494.3 to 15999.46) | -35.17  (-45.05 to -23.73) | -3  (-3.12 to -2.87) |
| Zambia | 5351 (4030 to 6857) | 18623.04 (14025.34 to 23864.05) | 4100 (3109 to 5203) | 8866.87 (6724.68 to 11252.74) | -23.39  (-33.14 to -10.18) | -2.51  (-2.59 to -2.42) |
| Zimbabwe | 3815 (2924 to 4886) | 13227.27 (10140.58 to 16942.88) | 3612 (2779 to 4662) | 10795.77 (8307.3 to 13934.84) | -5.31  (-19.46 to 8.36) | -0.4  (-0.56 to -0.24) |

CI, confidence interval; EAPC, estimated annual percentage change; LMICs, low- and middle-income countries and territories; UI, uncertain interval.

## Supplementary Table S13. National burden and trends of incident cases and incidence rates (per 100,000 liveborn neonates) of meningitis in 131 LMICs between 1990 and 2019.

| **Location** | **Incident cases in 1990**  **(95% UI)** | **Incident rate in 1990**  **(95% UI)** | **Incident cases in 2019**  **(95% UI)** | **Incident rate in 2019**  **(95% UI)** | **Percent change**  **(%, 95% CI)** | **EAPC**  **(%, 95% CI)** |
| --- | --- | --- | --- | --- | --- | --- |
| Global | 134339 (96269 to 182455) | 1301.16 (932.43 to 1767.19) | 87345 (62255 to 120910) | 854.79 (609.24 to 1183.26) | -34.98  (-36.47 to -33.36) | -1.87  (-2.23 to -1.51) |
| Afghanistan | 489 (351 to 658) | 1263.09 (907.46 to 1701.15) | 644 (467 to 868) | 576.14 (417.47 to 775.7) | 31.86  (16.24 to 51.7) | -3.25  (-3.84 to -2.66) |
| Albania | 33 (25 to 42) | 501.19 (382.02 to 639.12) | 4 (3 to 5) | 136.89 (98.48 to 185.14) | -88.81  (-90.58 to -86.82) | -5.11  (-5.72 to -4.49) |
| Algeria | 323 (230 to 442) | 555.47 (395.91 to 761.6) | 182 (125 to 261) | 275.1 (188.81 to 393.55) | -43.45  (-53.08 to -32.69) | -2.87  (-3.04 to -2.69) |
| American Samoa | 1 (0 to 1) | 541.5 (382.66 to 764.66) | 0 (0 to 0) | 402.92 (282.57 to 575.79) | -54.01  (-60.52 to -47.05) | -1.19  (-1.34 to -1.04) |
| Angola | 1549 (1124 to 2047) | 4088.36 (2966.5 to 5401.45) | 1413 (1023 to 1928) | 1694.16 (1226.59 to 2311.9) | -8.83  (-22.42 to 4.14) | -3.93  (-4.29 to -3.56) |
| Armenia | 16 (11 to 22) | 274.67 (197.24 to 381.62) | 4 (3 to 7) | 156.03 (101.35 to 250.86) | -71.71  (-78.12 to -63.54) | -2.44  (-2.67 to -2.21) |
| Azerbaijan | 103 (76 to 135) | 719.83 (533.38 to 943.53) | 37 (25 to 52) | 336.22 (230.95 to 478.29) | -64.5  (-70.05 to -58.2) | -3.19  (-3.72 to -2.66) |
| Bangladesh | 1127 (803 to 1648) | 385.02 (274.23 to 563.16) | 528 (356 to 804) | 259.99 (175.19 to 395.67) | -53.13  (-59.69 to -46.53) | -1.52  (-2.06 to -0.99) |
| Belarus | 66 (52 to 85) | 614.09 (482.45 to 795.37) | 17 (12 to 23) | 215.8 (155.91 to 294.64) | -74.25  (-78.69 to -68.75) | -4.42  (-4.77 to -4.08) |
| Belize | 3 (2 to 4) | 663.07 (507.2 to 839.46) | 2 (1 to 2) | 263.51 (196.99 to 338.72) | -48.13  (-53.67 to -40.64) | -3.83  (-4.09 to -3.56) |
| Benin | 593 (430 to 790) | 3334.48 (2416.16 to 4438.42) | 796 (571 to 1103) | 2103.84 (1509.6 to 2916.72) | 34.11  (16.81 to 56.95) | -2.26  (-2.49 to -2.03) |
| Bhutan | 30 (22 to 38) | 1810.4 (1320.68 to 2316.49) | 7 (5 to 10) | 657.37 (470.25 to 940.8) | -77.61  (-81.8 to -72.63) | -4.07  (-4.33 to -3.81) |
| Bolivia (Plurinational State of) | 104 (75 to 138) | 569.66 (409.42 to 753.02) | 44 (32 to 58) | 178.57 (131.68 to 239.46) | -58.13  (-64.57 to -51.29) | -4.64  (-5.01 to -4.27) |
| Bosnia and Herzegovina | 16 (12 to 21) | 309.98 (234.56 to 400.18) | 4 (3 to 5) | 186.43 (131.96 to 259.98) | -76.21  (-80.18 to -71.79) | -2.42  (-2.76 to -2.07) |
| Botswana | 27 (19 to 39) | 876.87 (621.71 to 1256.71) | 25 (18 to 35) | 682.98 (486.87 to 955.56) | -7.77  (-19.57 to 7.38) | -0.71  (-1.13 to -0.29) |
| Brazil | 1925 (1312 to 2723) | 741.87 (505.43 to 1049.23) | 395 (262 to 581) | 168.15 (111.51 to 247.17) | -79.48  (-81.84 to -77.96) | -0.2  (-2.05 to 1.69) |
| Bulgaria | 27 (21 to 35) | 365.15 (278.44 to 462.24) | 6 (5 to 9) | 138.88 (105.83 to 188.1) | -76.8  (-80.14 to -72.79) | -4.03  (-4.37 to -3.69) |
| Burkina Faso | 2447 (1799 to 3234) | 7138.71 (5248.16 to 9435.45) | 1692 (1264 to 2188) | 2399.51 (1791.76 to 3101.83) | -30.84  (-40.54 to -20.1) | -4.86  (-5.54 to -4.18) |
| Burundi | 594 (438 to 781) | 3000.53 (2216.66 to 3950.25) | 600 (431 to 837) | 1710.69 (1228.6 to 2387.12) | 1.08  (-11.94 to 14.58) | -2.38  (-2.68 to -2.07) |
| Cabo Verde | 23 (17 to 33) | 2621.25 (1883 to 3666.93) | 15 (11 to 21) | 1827.49 (1307.66 to 2570.45) | -36.18  (-45.42 to -25.21) | -1.83  (-2.34 to -1.32) |
| Cambodia | 309 (221 to 418) | 943.88 (674.46 to 1274) | 90 (64 to 127) | 326.11 (231.78 to 457.54) | -70.79  (-75.93 to -66.14) | -4.68  (-5.04 to -4.33) |
| Cameroon | 1269 (906 to 1713) | 3640.68 (2599.17 to 4916.74) | 1630 (1160 to 2280) | 2397.7 (1706.17 to 3354.7) | 28.48  (10.6 to 54.84) | -1.59  (-1.73 to -1.45) |
| Central African Republic | 358 (259 to 478) | 3807.23 (2751.81 to 5085.09) | 405 (291 to 550) | 2749.72 (1976.09 to 3734.36) | 13.16  (-1.27 to 30.6) | -1.31  (-1.6 to -1.01) |
| Chad | 1219 (900 to 1587) | 5143.31 (3795.54 to 6696.64) | 1994 (1430 to 2572) | 3370.06 (2417.52 to 4347.73) | 63.54  (41.49 to 88.3) | -1.64  (-1.95 to -1.32) |
| China | 6584 (4178 to 9629) | 361.53 (229.44 to 528.75) | 943 (610 to 1371) | 82.93 (53.66 to 120.58) | -85.67  (-86.55 to -84.8) | -5.76  (-6.01 to -5.51) |
| Colombia | 524 (412 to 651) | 742.96 (583.5 to 923.33) | 96 (73 to 126) | 160.09 (122.02 to 208.77) | -81.6  (-83.9 to -79.33) | -5.94  (-6.32 to -5.56) |
| Comoros | 45 (33 to 62) | 3086.12 (2242.88 to 4182.06) | 28 (20 to 37) | 2242.63 (1636.26 to 2997.73) | -38.6  (-47.34 to -29.13) | -1.48  (-1.64 to -1.32) |
| Congo | 184 (131 to 247) | 2453.53 (1746.15 to 3284.88) | 143 (102 to 207) | 1317.36 (942.88 to 1903.67) | -22.33  (-34.35 to -8.3) | -2.67  (-2.99 to -2.35) |
| Costa Rica | 22 (17 to 29) | 355.61 (273.9 to 469.11) | 8 (6 to 11) | 160.69 (120.18 to 208.69) | -62.88  (-68.16 to -57) | -3.17  (-3.32 to -3.02) |
| Côte d'Ivoire | 1323 (951 to 1782) | 3105.49 (2231.26 to 4181.44) | 1199 (868 to 1633) | 1809.48 (1309.89 to 2465.63) | -9.41  (-20.75 to 4.05) | -2.34  (-2.67 to -2.02) |
| Cuba | 102 (78 to 133) | 739.71 (564.39 to 960.48) | 17 (13 to 24) | 215.76 (157.66 to 296.35) | -83.12  (-85.69 to -80.19) | -4.38  (-5.36 to -3.38) |
| Democratic People's Republic of Korea | 196 (141 to 260) | 361.12 (260.45 to 479.44) | 44 (32 to 60) | 168.77 (121.07 to 229.86) | -77.49  (-81.19 to -73.3) | -3.15  (-3.44 to -2.86) |
| Democratic Republic of the Congo | 4405 (3185 to 5803) | 3144.46 (2273.99 to 4143.09) | 3508 (2505 to 4826) | 1594.1 (1138.45 to 2193.41) | -20.36  (-31.25 to -8.38) | -2.77  (-3.15 to -2.4) |
| Djibouti | 45 (33 to 61) | 2733.71 (1995.97 to 3699.87) | 47 (34 to 66) | 1817.34 (1322.46 to 2515.39) | 4.55  (-11.17 to 22.43) | -1.93  (-2.22 to -1.64) |
| Dominica | 1 (1 to 1) | 540.89 (397.08 to 724.66) | 0 (0 to 0) | 392.56 (283.55 to 533.95) | -64.68  (-68.95 to -59.59) | -1.41  (-1.58 to -1.23) |
| Dominican Republic | 207 (154 to 269) | 1151.17 (852.41 to 1493.56) | 86 (62 to 115) | 498.2 (358.63 to 665.01) | -58.53  (-64.3 to -51.53) | -3.21  (-3.46 to -2.96) |
| Ecuador | 109 (85 to 142) | 483.29 (377.33 to 626.1) | 43 (33 to 56) | 163.74 (122.87 to 211) | -60.28  (-66.61 to -54.48) | -4.81  (-5.14 to -4.49) |
| Egypt | 1192 (846 to 1658) | 838.89 (595.25 to 1167.19) | 484 (336 to 725) | 300.78 (208.41 to 450.37) | -59.37  (-67.82 to -48.34) | -4.04  (-4.24 to -3.84) |
| El Salvador | 48 (36 to 61) | 383.73 (292.03 to 489.51) | 8 (6 to 11) | 91.43 (67.72 to 124.66) | -83.54  (-85.9 to -80.93) | -5.69  (-5.94 to -5.43) |
| Equatorial Guinea | 54 (39 to 73) | 3159.04 (2251.84 to 4279.07) | 44 (32 to 63) | 1516.12 (1089.78 to 2144.63) | -18.03  (-30.48 to -0.87) | -3.06  (-3.24 to -2.89) |
| Eritrea | 266 (191 to 363) | 2849.53 (2044.75 to 3891.12) | 262 (188 to 371) | 1747.15 (1251.04 to 2467.84) | -1.28  (-15.91 to 13.97) | -2.11  (-2.33 to -1.89) |
| Eswatini | 17 (12 to 23) | 718.22 (517.12 to 952.23) | 11 (8 to 15) | 500.82 (362.59 to 670.62) | -35.53  (-44.19 to -26.15) | -1.34  (-1.83 to -0.85) |
| Ethiopia | 9459 (6596 to 12928) | 5135.63 (3581.29 to 7019.4) | 4488 (3084 to 6343) | 1631.95 (1121.41 to 2306.44) | -52.55  (-56.41 to -48.29) | -4.31  (-4.84 to -3.77) |
| Fiji | 10 (7 to 15) | 708.31 (498.05 to 1010.79) | 5 (4 to 8) | 402.84 (281.86 to 572.55) | -47.5  (-54.12 to -38.98) | -1.44  (-2.31 to -0.57) |
| Gabon | 69 (49 to 97) | 2347.36 (1673.28 to 3269.39) | 52 (37 to 73) | 1618.03 (1160.5 to 2274.34) | -24.81  (-34.13 to -15.03) | -1.47  (-1.68 to -1.27) |
| Gambia | 142 (105 to 194) | 3937.49 (2912.89 to 5372.62) | 81 (57 to 116) | 1510.6 (1065.64 to 2159.01) | -42.87  (-51.36 to -33.08) | -3.52  (-3.9 to -3.14) |
| Georgia | 34 (25 to 46) | 540.76 (399.84 to 735.35) | 8 (6 to 11) | 232.07 (163.32 to 321.37) | -76.42  (-79.88 to -72.08) | -3.53  (-3.81 to -3.25) |
| Ghana | 1483 (1078 to 2070) | 3281 (2385.17 to 4579.55) | 1012 (727 to 1357) | 1575.51 (1131.39 to 2112.82) | -31.75  (-40.75 to -21.37) | -3.44  (-3.77 to -3.11) |
| Grenada | 1 (1 to 1) | 627.17 (486.5 to 788.15) | 0 (0 to 0) | 226.33 (164.46 to 316.69) | -74.69  (-78.47 to -68.74) | -3.93  (-4.27 to -3.6) |
| Guatemala | 72 (56 to 91) | 283.11 (220.81 to 357.99) | 36 (28 to 47) | 116.3 (89.51 to 151.52) | -49.4  (-55.65 to -42.08) | -3.66  (-3.91 to -3.4) |
| Guinea | 1373 (995 to 1823) | 5861.5 (4246.72 to 7780.64) | 1465 (1081 to 1996) | 4074.55 (3005.34 to 5549.11) | 6.71  (-6.71 to 22.53) | -1.66  (-1.81 to -1.52) |
| Guinea-Bissau | 213 (157 to 279) | 6147.48 (4523.58 to 8066.6) | 166 (121 to 238) | 3549.27 (2578.68 to 5079.89) | -21.95  (-34.05 to -6.6) | -2.48  (-2.7 to -2.25) |
| Guyana | 10 (8 to 13) | 596.31 (452.23 to 776.48) | 2 (2 to 3) | 202.2 (147.5 to 280.4) | -77.99  (-80.97 to -74.41) | -4.18  (-4.41 to -3.94) |
| Haiti | 334 (246 to 441) | 1806.81 (1333.51 to 2386.72) | 275 (198 to 368) | 1107.83 (796.15 to 1482.34) | -17.48  (-28.03 to -6.63) | -1.91  (-2.31 to -1.5) |
| Honduras | 58 (42 to 77) | 426.06 (307.77 to 565.94) | 29 (20 to 41) | 163.41 (114.5 to 228.28) | -49.57  (-55.97 to -41.97) | -3.75  (-4.14 to -3.36) |
| India | 25865 (18017 to 36532) | 1317.42 (917.66 to 1860.72) | 12105 (8160 to 17581) | 682.87 (460.32 to 991.8) | -53.2  (-55.48 to -50.55) | -2.24  (-2.48 to -2) |
| Indonesia | 4944 (3302 to 6879) | 1378.16 (920.23 to 1917.44) | 1365 (913 to 1927) | 468.7 (313.35 to 661.57) | -72.39  (-73.88 to -70.96) | -4.17  (-4.59 to -3.76) |
| Iran (Islamic Republic of) | 817 (558 to 1151) | 607.66 (415.13 to 856.21) | 221 (145 to 324) | 215.33 (141.75 to 316.42) | -72.97  (-75.29 to -70.76) | -4.21  (-4.4 to -4.01) |
| Iraq | 483 (347 to 649) | 914.81 (656.26 to 1228.85) | 308 (224 to 416) | 423.24 (307.63 to 572.33) | -36.3  (-44.36 to -24.49) | -3  (-3.44 to -2.56) |
| Jamaica | 33 (26 to 42) | 783.3 (606.34 to 988.6) | 10 (8 to 14) | 379.21 (286.52 to 493.54) | -68.79  (-72.81 to -63.51) | -3.01  (-3.2 to -2.81) |
| Jordan | 50 (35 to 70) | 480.49 (341.18 to 678.38) | 61 (42 to 87) | 324.59 (227.32 to 463.4) | 22.3  (6.39 to 45.63) | -2.06  (-2.47 to -1.66) |
| Kazakhstan | 241 (188 to 307) | 895.76 (697.55 to 1139.46) | 71 (51 to 94) | 263.24 (191.26 to 350.14) | -70.68  (-75.41 to -64.84) | -4.97  (-5.44 to -4.5) |
| Kenya | 1779 (1216 to 2505) | 2363.82 (1615.6 to 3328.28) | 1273 (858 to 1834) | 1254.93 (846.04 to 1808.61) | -28.44  (-32.45 to -25.26) | -2.33  (-2.52 to -2.13) |
| Kiribati | 3 (2 to 4) | 1642.31 (1232.39 to 2158.68) | 2 (1 to 2) | 762.04 (556.08 to 1049.56) | -45.31  (-52.62 to -37.61) | -3.05  (-3.35 to -2.75) |
| Kyrgyzstan | 133 (104 to 165) | 1394.22 (1092.73 to 1733.67) | 37 (28 to 49) | 331.44 (249.37 to 447.91) | -72.4  (-76.96 to -66.32) | -5.88  (-6.2 to -5.56) |
| Lao People's Democratic Republic | 194 (128 to 265) | 1570.73 (1036.26 to 2138.65) | 67 (49 to 89) | 519.26 (382.35 to 685.85) | -65.44  (-71.69 to -57) | -4.75  (-5.19 to -4.31) |
| Lebanon | 36 (25 to 51) | 431.89 (305.45 to 613.52) | 16 (11 to 23) | 200.17 (134.75 to 292.13) | -55.2  (-62.27 to -46.7) | -3.11  (-3.28 to -2.94) |
| Lesotho | 43 (31 to 58) | 949.8 (691.11 to 1273.73) | 23 (17 to 32) | 673.21 (487.47 to 918.71) | -45.5  (-51.82 to -38.63) | -1.29  (-1.72 to -0.86) |
| Liberia | 257 (183 to 336) | 4279.55 (3056.48 to 5604) | 180 (129 to 249) | 1779.88 (1276.44 to 2459.36) | -29.79  (-40.54 to -17.39) | -3.94  (-4.25 to -3.64) |
| Libya | 50 (35 to 72) | 444.33 (311.86 to 638.77) | 11 (7 to 16) | 171.25 (112.63 to 259.48) | -78.85  (-83.02 to -74.51) | -3.95  (-4.19 to -3.71) |
| Madagascar | 1102 (800 to 1442) | 2762.74 (2005.71 to 3614.08) | 1034 (728 to 1454) | 1603.57 (1128.87 to 2254.41) | -6.2  (-19.71 to 11.63) | -2.36  (-2.58 to -2.13) |
| Malawi | 1358 (990 to 1805) | 3904.16 (2846.53 to 5191.54) | 850 (609 to 1154) | 2058.49 (1474.12 to 2794.53) | -37.4  (-45.44 to -28.04) | -2.75  (-2.95 to -2.55) |
| Malaysia | 192 (139 to 263) | 508.04 (368.83 to 695.45) | 129 (91 to 184) | 312.57 (220.86 to 444.15) | -32.63  (-43.55 to -22.21) | -2.06  (-2.51 to -1.6) |
| Maldives | 3 (2 to 4) | 428.72 (304.3 to 620.9) | 1 (1 to 2) | 209.45 (146.8 to 311.97) | -52.69  (-59.8 to -44.47) | -3.02  (-3.27 to -2.78) |
| Mali | 2650 (1946 to 3446) | 8065.28 (5924.76 to 10489.57) | 2414 (1786 to 3255) | 3348.23 (2476.48 to 4513.43) | -8.88  (-19.19 to 2.63) | -3.57  (-3.89 to -3.24) |
| Marshall Islands | 1 (1 to 1) | 704.94 (492.7 to 990.17) | 0 (0 to 1) | 386.26 (270.98 to 574.81) | -55.38  (-62.15 to -48.68) | -2.52  (-2.7 to -2.34) |
| Mauritania | 179 (128 to 250) | 2680.42 (1922.79 to 3756.3) | 134 (98 to 188) | 1626.33 (1188.14 to 2281.86) | -24.91  (-33.95 to -15.34) | -1.89  (-2.36 to -1.41) |
| Mexico | 509 (326 to 740) | 271.06 (173.67 to 394.4) | 96 (60 to 145) | 60.05 (37.91 to 90.72) | -81.2  (-82.58 to -79.53) | -6.49  (-7.11 to -5.87) |
| Micronesia (Federated States of) | 2 (1 to 3) | 732.8 (522.7 to 1039.05) | 0 (0 to 1) | 315.25 (213.15 to 460.04) | -75.78  (-79.57 to -71.46) | -3.51  (-3.74 to -3.27) |
| Mongolia | 65 (46 to 87) | 1179.45 (840.92 to 1579.63) | 12 (8 to 18) | 200.33 (136.75 to 300.51) | -81.21  (-85.09 to -76.9) | -7.6  (-8.15 to -7.05) |
| Montenegro | 2 (1 to 2) | 207.69 (151.68 to 282.15) | 0 (0 to 1) | 92.5 (63.26 to 135.17) | -70.08  (-75.19 to -64.04) | -3.43  (-3.74 to -3.12) |
| Morocco | 651 (471 to 869) | 1094.84 (792 to 1462.33) | 166 (116 to 239) | 364.03 (254.22 to 522.53) | -74.47  (-78.35 to -69.44) | -4.56  (-4.94 to -4.18) |
| Mozambique | 1603 (1181 to 2111) | 3672.62 (2704.85 to 4835.74) | 1280 (923 to 1788) | 1514.09 (1091.59 to 2115.65) | -20.2  (-33.89 to -7.01) | -3.71  (-4.04 to -3.37) |
| Myanmar | 1694 (1201 to 2269) | 1707.16 (1210.7 to 2286.9) | 529 (386 to 697) | 671.37 (489.11 to 884.75) | -68.76  (-73.32 to -62.6) | -4.33  (-4.81 to -3.84) |
| Namibia | 30 (21 to 42) | 816.29 (572.04 to 1154.21) | 24 (17 to 34) | 507.2 (358.89 to 717.9) | -19.43  (-32.49 to -4.35) | -1.76  (-2.06 to -1.46) |
| Nepal | 707 (512 to 943) | 1165.89 (844.65 to 1555) | 212 (151 to 308) | 455.04 (324.85 to 663.31) | -70.09  (-75.02 to -65.16) | -3.99  (-4.34 to -3.63) |
| Nicaragua | 63 (47 to 82) | 581.57 (431.62 to 755.51) | 12 (8 to 15) | 115.57 (83.56 to 149.19) | -81.77  (-84 to -78.67) | -6.72  (-7.16 to -6.28) |
| Niger | 3322 (2422 to 4189) | 9994.73 (7288.63 to 12602.65) | 3157 (2338 to 4194) | 3654.92 (2707.56 to 4856.29) | -4.97  (-17.89 to 10.63) | -4.15  (-4.48 to -3.82) |
| Nigeria | 14262 (9907 to 19336) | 4630.54 (3216.62 to 6277.87) | 15480 (10793 to 21150) | 2740.64 (1910.78 to 3744.58) | 8.54  (4.82 to 12.09) | -2.23  (-2.61 to -1.85) |
| North Macedonia | 12 (9 to 15) | 449.45 (338.28 to 571.53) | 2 (2 to 3) | 142.67 (105.13 to 195.88) | -79.78  (-82.68 to -76.5) | -4.77  (-5.16 to -4.37) |
| Pakistan | 8110 (5545 to 11187) | 2267.57 (1550.47 to 3127.93) | 5648 (3883 to 7849) | 1134.27 (779.87 to 1576.32) | -30.36  (-36.38 to -24.01) | -2.77  (-3.19 to -2.34) |
| Papua New Guinea | 202 (147 to 265) | 1804.78 (1314.89 to 2360.4) | 252 (180 to 343) | 1018.17 (730.08 to 1387.71) | 24.27  (7.57 to 42.17) | -2.19  (-2.44 to -1.95) |
| Paraguay | 149 (107 to 203) | 1536.1 (1102.04 to 2097.95) | 34 (25 to 47) | 354.84 (256.64 to 492.68) | -77.09  (-80.44 to -73.39) | -5.67  (-6 to -5.34) |
| Peru | 178 (131 to 234) | 356.01 (261.61 to 468.26) | 52 (37 to 73) | 107.25 (76.64 to 149.3) | -70.71  (-75.25 to -65.04) | -4.9  (-5.15 to -4.65) |
| Philippines | 790 (545 to 1131) | 505.37 (348.44 to 723.52) | 498 (337 to 727) | 247.96 (167.97 to 361.95) | -36.95  (-39.33 to -34.49) | -2.81  (-2.94 to -2.67) |
| Republic of Moldova | 41 (31 to 50) | 691.29 (534.62 to 861.09) | 7 (5 to 9) | 291.48 (219.93 to 384.35) | -82.37  (-84.77 to -79.44) | -3.52  (-3.7 to -3.34) |
| Russian Federation | 847 (570 to 1218) | 575.69 (387.4 to 827.25) | 208 (140 to 307) | 163.15 (109.58 to 240.62) | -75.45  (-77.38 to -74) | -5.1  (-5.44 to -4.75) |
| Rwanda | 812 (598 to 1071) | 3414.99 (2516.18 to 4501.58) | 440 (313 to 603) | 1652.38 (1176.98 to 2266.08) | -45.86  (-53.91 to -36.38) | -3.55  (-3.92 to -3.18) |
| Saint Lucia | 2 (1 to 2) | 672.18 (517.42 to 857.62) | 0 (0 to 1) | 302.35 (221.77 to 404.07) | -78.78  (-81.44 to -75.19) | -3.27  (-3.47 to -3.06) |
| Saint Vincent and the Grenadines | 2 (1 to 2) | 895.62 (698.52 to 1121.44) | 0 (0 to 1) | 340.6 (262.78 to 436.74) | -78.21  (-80.89 to -75.34) | -3.99  (-4.22 to -3.76) |
| Samoa | 2 (1 to 2) | 609.19 (428.52 to 862.99) | 1 (1 to 2) | 348.95 (242.16 to 523.07) | -35.86  (-44.74 to -27.66) | -2.23  (-2.42 to -2.05) |
| Senegal | 1224 (909 to 1653) | 4770.93 (3544.25 to 6445.03) | 870 (626 to 1222) | 2492.03 (1791.69 to 3499.31) | -28.9  (-38.23 to -18.25) | -2.64  (-2.9 to -2.38) |
| Serbia | 33 (25 to 44) | 331.04 (246.55 to 432.11) | 6 (5 to 9) | 99.38 (73.85 to 135.76) | -81.28  (-84.04 to -77.85) | -4.91  (-5.17 to -4.65) |
| Sierra Leone | 512 (375 to 677) | 4379.22 (3203.35 to 5784.27) | 465 (340 to 620) | 2219.25 (1620.45 to 2954.83) | -9.19  (-21.74 to 4.7) | -3.05  (-3.41 to -2.69) |
| Solomon Islands | 7 (5 to 10) | 675.76 (484.27 to 932.25) | 5 (3 to 7) | 300.82 (206.28 to 418.88) | -34.77  (-43.89 to -22.16) | -3.17  (-3.45 to -2.89) |
| Somalia | 764 (548 to 1007) | 3317.67 (2377.67 to 4374.63) | 1872 (1387 to 2471) | 2867.77 (2124.76 to 3785.81) | 144.99  (109.61 to 180.97) | -0.82  (-0.99 to -0.64) |
| South Africa | 602 (419 to 849) | 820.97 (571.57 to 1157.17) | 348 (232 to 495) | 447.21 (297.34 to 635.51) | -42.14  (-46.33 to -38.06) | -2.32  (-2.57 to -2.07) |
| South Sudan | 1038 (753 to 1434) | 5538.2 (4019.13 to 7653.77) | 1508 (1088 to 2061) | 5566.3 (4016.89 to 7606.54) | 45.33  (25.75 to 68.19) | 0  (-0.11 to 0.11) |
| Sri Lanka | 258 (189 to 355) | 953.2 (697.37 to 1311.6) | 111 (79 to 153) | 490.41 (347.95 to 674.35) | -57.05  (-63.18 to -49.88) | -2.67  (-2.9 to -2.43) |
| Sudan | 807 (582 to 1069) | 1110.41 (801.23 to 1471.31) | 251 (178 to 341) | 283.64 (201.17 to 384.78) | -68.85  (-73.15 to -63.83) | -4.98  (-5.69 to -4.26) |
| Suriname | 6 (5 to 8) | 936.29 (687.14 to 1226.93) | 3 (2 to 4) | 404.72 (293.47 to 548.3) | -57.11  (-63.33 to -50.61) | -3.56  (-3.8 to -3.31) |
| Syrian Arab Republic | 261 (188 to 360) | 687.79 (494.45 to 947.82) | 50 (35 to 69) | 280.89 (195.43 to 389.37) | -80.95  (-84.38 to -78.04) | -3.65  (-4.35 to -2.95) |
| Tajikistan | 188 (141 to 240) | 1213.77 (910.61 to 1552.11) | 95 (66 to 134) | 494.87 (343.57 to 697.7) | -49.2  (-57.87 to -38.6) | -3.84  (-4.43 to -3.24) |
| Thailand | 555 (398 to 773) | 690.37 (494.67 to 961.66) | 132 (93 to 191) | 295.8 (209.01 to 428.61) | -76.24  (-79.71 to -71.35) | -3.76  (-4.09 to -3.43) |
| Timor-Leste | 29 (22 to 37) | 1092.92 (802.67 to 1377.25) | 12 (9 to 17) | 420.38 (300.02 to 599.55) | -58.41  (-66.01 to -48.4) | -4.01  (-4.24 to -3.78) |
| Togo | 326 (235 to 435) | 2669.58 (1926.52 to 3561.82) | 244 (171 to 346) | 1371.71 (961.38 to 1944.55) | -25.23  (-35 to -12.78) | -2.7  (-2.99 to -2.41) |
| Tonga | 2 (2 to 3) | 1189.87 (862.97 to 1643.34) | 1 (1 to 2) | 771.72 (556.46 to 1080.45) | -41.64  (-49.58 to -32.88) | -2.04  (-2.53 to -1.55) |
| Tunisia | 95 (68 to 132) | 531.15 (379.43 to 738.19) | 30 (21 to 44) | 239.04 (164.43 to 343.8) | -68.01  (-72.94 to -62.42) | -3.19  (-3.32 to -3.06) |
| Turkey | 505 (361 to 676) | 407.83 (291.19 to 546.05) | 50 (34 to 77) | 67.84 (46.08 to 102.86) | -90.01  (-92.22 to -87.42) | -7.68  (-8.19 to -7.18) |
| Turkmenistan | 111 (87 to 140) | 1123.8 (876.84 to 1418.62) | 40 (30 to 52) | 465.68 (356.85 to 607.74) | -64.42  (-69.11 to -57.73) | -3.81  (-4.32 to -3.29) |
| Tuvalu | 0 (0 to 0) | 881.91 (630.8 to 1202) | 0 (0 to 0) | 368.91 (255.19 to 514.03) | -68.2  (-72.9 to -62.7) | -3.6  (-3.84 to -3.36) |
| Uganda | 2309 (1664 to 3061) | 3339.27 (2406.6 to 4427.38) | 2445 (1772 to 3439) | 2061.24 (1494.29 to 2899.62) | 5.91  (-8.21 to 24.01) | -2.05  (-2.25 to -1.84) |
| Ukraine | 280 (187 to 410) | 558.61 (372.37 to 817.88) | 57 (37 to 85) | 191.03 (124.34 to 287.91) | -79.75  (-83.1 to -76.33) | -4.38  (-4.62 to -4.14) |
| United Republic of Tanzania | 2119 (1564 to 2776) | 2330.09 (1719.37 to 3052.09) | 2367 (1691 to 3362) | 1524.62 (1089.27 to 2165.94) | 11.68  (-6.93 to 30.55) | -1.8  (-2.03 to -1.56) |
| Uzbekistan | 510 (400 to 634) | 945.56 (741.21 to 1176.79) | 135 (97 to 191) | 248.19 (178.51 to 352.58) | -73.59  (-78.67 to -67.99) | -5.5  (-5.83 to -5.17) |
| Vanuatu | 3 (2 to 4) | 617.75 (438.13 to 878.42) | 3 (2 to 4) | 442.95 (318.29 to 630.77) | -2.89  (-15.51 to 14.3) | -1.26  (-1.41 to -1.11) |
| Venezuela (Bolivarian Republic of) | 291 (226 to 364) | 672.21 (522.79 to 842.71) | 71 (55 to 94) | 197.65 (151.5 to 260.59) | -75.54  (-78.53 to -71.73) | -4.73  (-5.11 to -4.35) |
| Viet Nam | 837 (601 to 1126) | 606.73 (435.63 to 816.2) | 275 (196 to 394) | 271.31 (192.61 to 388.06) | -67.11  (-72.81 to -61.22) | -3.46  (-3.68 to -3.25) |
| Yemen | 384 (271 to 510) | 759.46 (536.03 to 1008.83) | 252 (182 to 346) | 357.38 (259.11 to 490.91) | -34.41  (-43 to -25.32) | -3.21  (-3.46 to -2.96) |
| Zambia | 963 (697 to 1275) | 3350.75 (2426.64 to 4436.97) | 791 (573 to 1091) | 1711.51 (1238.57 to 2358.67) | -17.81  (-30.74 to -2.03) | -2.83  (-3.12 to -2.55) |
| Zimbabwe | 338 (247 to 450) | 1171.48 (856.4 to 1559.64) | 297 (211 to 410) | 888.07 (629.49 to 1224.29) | -12.05  (-25.14 to 1.82) | -0.95  (-1.36 to -0.54) |

CI, confidence interval; EAPC, estimated annual percentage change; LMICs, low- and middle-income countries and territories; UI, uncertain interval.

## Supplementary Table S14. National burden and trends of incident cases and incidence rates (per 100,000 liveborn neonates) of neglected tropical diseases and malaria in 131 LMICs between 1990 and 2019.

| **Location** | **Incident cases in 1990**  **(95% UI)** | **Incident rate in 1990**  **(95% UI)** | **Incident cases in 2019**  **(95% UI)** | **Incident rate in 2019**  **(95% UI)** | **Percent change**  **(%, 95% CI)** | **EAPC**  **(%, 95% CI)** |
| --- | --- | --- | --- | --- | --- | --- |
| Global | 608367 (488243 to 751614) | 5892.42 (4728.95 to 7279.86) | 494799 (322018 to 700708) | 4842.26 (3151.37 to 6857.35) | -18.67  (-41.76 to 3.38) | -0.88  (-1.24 to -0.52) |
| Afghanistan | 1889 (1027 to 3278) | 4882.64 (2654.3 to 8472.22) | 856 (50 to 4173) | 765.76 (44.7 to 3731.2) | -54.66  (-97.42 to 137.77) | -9.36  (-11.33 to -7.35) |
| Albania | 0 (0 to 0) | 0 (0 to 0) | 0 (0 to 0) | 0 (0 to 0) | 0  (0 to 0) | 0  (0 to 0) |
| Algeria | 0 (0 to 2) | 0.72 (0.11 to 2.71) | 0 (0 to 0) | 0 (0 to 0.01) | -99.72  (-99.95 to -98.33) | -20.16  (-22.43 to -17.82) |
| American Samoa | 0 (0 to 0) | 0 (0 to 0) | 0 (0 to 0) | 0 (0 to 0) | 0  (0 to 0) | 82.18  (21.58 to 172.98) |
| Angola | 11072 (5964 to 17578) | 29217.09 (15739.01 to 46387.23) | 15160 (8147 to 25056) | 18183.37 (9771.79 to 30052.41) | 36.93  (-17.56 to 129.97) | -2.65  (-3.32 to -1.97) |
| Armenia | 0 (0 to 0) | 0 (0 to 0) | 0 (0 to 0) | 0 (0 to 0) | 0  (0 to 0) | -32.71  (-62.65 to 21.24) |
| Azerbaijan | 0 (0 to 0) | 0.15 (0.09 to 0.22) | 0 (0 to 0) | 0 (0 to 0) | -100  (-100 to -100) | -63.56  (-73.33 to -50.19) |
| Bangladesh | 3516 (136 to 20667) | 1201.14 (46.38 to 7060.44) | 1 (0 to 4) | 0.42 (0.04 to 1.81) | -99.98  (-100 to -99.35) | -21.5  (-23.14 to -19.83) |
| Belarus | 0 (0 to 0) | 0 (0 to 0) | 0 (0 to 0) | 0 (0 to 0) | 0  (0 to 0) | 0  (0 to 0) |
| Belize | 6 (0 to 49) | 1369.44 (8.89 to 11098.57) | 1 (0 to 3) | 196.92 (49.44 to 580.93) | -81.23  (-98.34 to 2852.13) | -13.04  (-20.36 to -5.03) |
| Benin | 6963 (4067 to 9678) | 39134.83 (22859.46 to 54392.65) | 13979 (9534 to 18632) | 36965.82 (25210.91 to 49267.82) | 100.77  (47.95 to 195.78) | -0.12  (-0.37 to 0.13) |
| Bhutan | 546 (36 to 2358) | 33199.84 (2194.25 to 143486.38) | 0 (0 to 0) | 0.76 (0.1 to 2.81) | -100  (-100 to -99.97) | -33.62  (-34.61 to -32.61) |
| Bolivia (Plurinational State of) | 837 (110 to 4475) | 4580.75 (600.53 to 24505.62) | 46 (27 to 90) | 186.74 (111.43 to 368.67) | -94.55  (-98.99 to -50.72) | -11.14  (-12.76 to -9.49) |
| Bosnia and Herzegovina | 0 (0 to 0) | 0 (0 to 0) | 0 (0 to 0) | 0 (0 to 0) | 0  (0 to 0) | 0  (0 to 0) |
| Botswana | 0 (0 to 1) | 13.44 (8.8 to 19.69) | 1 (0 to 1) | 16.39 (0.65 to 29.63) | 44.43  (-92.78 to 201.2) | -4.67  (-7.59 to -1.65) |
| Brazil | 1775 (1438 to 2171) | 683.81 (554.19 to 836.51) | 181 (121 to 286) | 77.05 (51.7 to 121.63) | -89.8  (-93.49 to -83.34) | -5.88  (-6.86 to -4.88) |
| Bulgaria | 0 (0 to 0) | 0 (0 to 0) | 0 (0 to 0) | 0 (0 to 0) | 0  (0 to 0) | 0  (0 to 0) |
| Burkina Faso | 18852 (15683 to 22278) | 54997.17 (45751.61 to 64993.24) | 13765 (7842 to 21504) | 19515.67 (11118.56 to 30488.07) | -26.98  (-62.09 to 8.55) | -2.49  (-3.28 to -1.69) |
| Burundi | 8682 (4638 to 11558) | 43889.85 (23443.77 to 58426.14) | 5416 (1587 to 9876) | 15440.94 (4526.26 to 28159.06) | -37.62  (-80.25 to 7.84) | -4.71  (-5.59 to -3.83) |
| Cabo Verde | 0 (0 to 0) | 0.08 (0.06 to 0.11) | 3 (0 to 7) | 337.37 (0.08 to 803.94) | 377138.17  (-21.54 to 955847.21) | 14.04  (6.4 to 22.23) |
| Cambodia | 303 (230 to 393) | 922.83 (700.99 to 1200.21) | 6 (2 to 17) | 22.64 (6.07 to 61.52) | -97.93  (-99.45 to -94.05) | -12.42  (-13.99 to -10.83) |
| Cameroon | 13308 (10092 to 16955) | 38193.67 (28962.24 to 48657.37) | 15871 (6303 to 29069) | 23348.18 (9272 to 42764.47) | 19.26  (-44.09 to 96.87) | -2.06  (-2.48 to -1.64) |
| Central African Republic | 4302 (2814 to 5518) | 45742.9 (29918.03 to 58665.51) | 4944 (2341 to 7259) | 33550.57 (15889.07 to 49262.03) | 14.91  (-33.94 to 70.89) | -0.99  (-1.25 to -0.73) |
| Chad | 4762 (2300 to 8315) | 20090.6 (9702.12 to 35077.5) | 7189 (2738 to 15739) | 12152.35 (4627.67 to 26604.18) | 50.97  (-31.96 to 198.48) | -1.85  (-2.12 to -1.57) |
| China | 80 (39 to 273) | 4.37 (2.14 to 14.99) | 0 (0 to 0) | 0 (0 to 0) | -100  (-100 to -100) | -52.56  (-63.52 to -38.31) |
| Colombia | 192 (141 to 258) | 272.88 (199.53 to 365.41) | 21 (10 to 44) | 34.14 (17.11 to 73.18) | -89.32  (-95.02 to -75.62) | -4.58  (-7.71 to -1.35) |
| Comoros | 4719 (1 to 1833) | 320767.4 (79.93 to 124560.66) | 60 (0 to 528) | 4826.59 (17.57 to 42512.38) | -98.73  (-99.87 to 4591.86) | -15.19  (-20.31 to -9.73) |
| Congo | 3055 (1479 to 4345) | 40694.11 (19698.48 to 57882.82) | 2590 (1006 to 4547) | 23851.88 (9260.23 to 41867) | -15.21  (-58.4 to 54.4) | -2.9  (-3.45 to -2.35) |
| Costa Rica | 3 (2 to 3) | 43.39 (33.28 to 55.42) | 2 (1 to 5) | 40.32 (16.89 to 106.54) | -23.68  (-67.33 to 95.31) | -2.53  (-5.56 to 0.6) |
| Côte d'Ivoire | 22553 (17879 to 29227) | 52933 (41963.3 to 68598.87) | 21484 (12362 to 33437) | 32431.16 (18661.98 to 50474.71) | -4.74  (-46.95 to 41.99) | -1.8  (-2.23 to -1.36) |
| Cuba | 0 (0 to 0) | 0 (0 to 0) | 1 (0 to 3) | 11.56 (2.87 to 36.42) | 0  (0 to 0) | 110.78  (44.36 to 207.76) |
| Democratic People's Republic of Korea | 0 (0 to 0) | 0 (0 to 0) | 1 (0 to 3) | 2.68 (0.32 to 10.07) | 0  (0 to 0) | 157.95  (66.22 to 300.3) |
| Democratic Republic of the Congo | 63872 (49521 to 79643) | 45599.27 (35353.69 to 56858.38) | 67726 (41201 to 99427) | 30778.98 (18724.08 to 45185.82) | 6.03  (-28.79 to 33.23) | -1.64  (-2.08 to -1.2) |
| Djibouti | 9 (6 to 12) | 520.27 (360.66 to 721.16) | 60 (0 to 461) | 2312.45 (4.55 to 17661.33) | 598.99  (-98.37 to 5785.96) | 6.04  (0.99 to 11.33) |
| Dominica | 0 (0 to 0) | 0 (0 to 0) | 0 (0 to 0) | 0 (0 to 0) | 0  (0 to 0) | 73.78  (19.73 to 152.24) |
| Dominican Republic | 0 (0 to 0) | 1.25 (0.98 to 1.6) | 0 (0 to 0) | 0.6 (0.12 to 1.88) | -53.68  (-90.86 to 50.21) | -2.97  (-5.49 to -0.38) |
| Ecuador | 143 (99 to 204) | 629.18 (437.15 to 900.18) | 9 (6 to 14) | 35.5 (22.42 to 54.24) | -93.38  (-96.15 to -88.45) | -6.55  (-9.51 to -3.49) |
| Egypt | 0 (0 to 0) | 0 (0 to 0) | 0 (0 to 0) | 0 (0 to 0) | 0  (0 to 0) | 0  (0 to 0) |
| El Salvador | 12 (9 to 16) | 99.33 (75.48 to 129.3) | 12 (4 to 36) | 145.75 (46.61 to 422.76) | 1.36  (-66.99 to 215.87) | 8.69  (2.88 to 14.82) |
| Equatorial Guinea | 645 (358 to 864) | 37735.44 (20951.75 to 50544.36) | 827 (199 to 1332) | 28322.56 (6801.66 to 45606.49) | 28.19  (-68.83 to 99.73) | -0.77  (-0.99 to -0.56) |
| Eritrea | 322 (30 to 1410) | 3448.96 (318.77 to 15116.41) | 88 (13 to 338) | 588.29 (85.58 to 2249.13) | -72.54  (-97.86 to 296.27) | -6.55  (-8.93 to -4.11) |
| Eswatini | 5 (0 to 30) | 210.61 (5.65 to 1255.06) | 0 (0 to 1) | 11.23 (0 to 28.76) | -95.07  (-100 to 98.36) | -16.07  (-19.11 to -12.91) |
| Ethiopia | 1070 (329 to 2585) | 580.75 (178.9 to 1403.79) | 7354 (1305 to 26659) | 2673.89 (474.4 to 9693.14) | 587.53  (2.27 to 3654.51) | 6.17  (3.02 to 9.42) |
| Fiji | 0 (0 to 0) | 0 (0 to 0) | 0 (0 to 0) | 0 (0 to 0) | 0  (0 to 0) | 0  (0 to 0) |
| Gabon | 1271 (605 to 1771) | 43043.77 (20490.63 to 59979.99) | 878 (314 to 1603) | 27241.77 (9735.7 to 49756.9) | -30.96  (-72.08 to 19.33) | -1.53  (-2.81 to -0.23) |
| Gambia | 590 (390 to 841) | 16377.19 (10836.92 to 23337.83) | 178 (19 to 657) | 3325.59 (345.63 to 12241.17) | -69.76  (-96.71 to 13.62) | -2.19  (-4.09 to -0.25) |
| Georgia | 0 (0 to 0) | 0 (0 to 0) | 0 (0 to 0) | 0 (0 to 0) | 0  (0 to 0) | -31.72  (-63.05 to 26.19) |
| Ghana | 21588 (16107 to 26909) | 47768.57 (35641.19 to 59542.63) | 14004 (6369 to 22817) | 21800.74 (9915.14 to 35519.27) | -35.13  (-71.03 to 6.6) | -2.58  (-3.17 to -1.98) |
| Grenada | 0 (0 to 0) | 0 (0 to 0) | 0 (0 to 0) | 0 (0 to 0) | 0  (0 to 0) | 77.97  (20.66 to 162.49) |
| Guatemala | 269 (174 to 399) | 1060.3 (684.19 to 1571.48) | 28 (12 to 72) | 89.77 (36.97 to 230.88) | -89.57  (-96.06 to -70.11) | -9.54  (-11.32 to -7.72) |
| Guinea | 10545 (7752 to 13651) | 45016.76 (33094.06 to 58274.03) | 9013 (3127 to 15752) | 25063.85 (8694.13 to 43800.96) | -14.53  (-67.47 to 36.95) | -1.68  (-2.05 to -1.3) |
| Guinea-Bissau | 1619 (1036 to 2056) | 46776.8 (29936.58 to 59402.09) | 436 (85 to 1227) | 9308.87 (1808.37 to 26206.93) | -73.1  (-94.58 to -21.64) | -8.81  (-10.23 to -7.37) |
| Guyana | 58 (42 to 79) | 3472.38 (2495.29 to 4737.58) | 10 (3 to 26) | 963.33 (264.07 to 2441.98) | -81.99  (-95.17 to -50.01) | -8.91  (-10.44 to -7.37) |
| Haiti | 16 (10 to 22) | 84.16 (54.74 to 120.41) | 18 (2 to 81) | 73.64 (7.81 to 327.25) | 17.76  (-86.98 to 430.61) | -3.32  (-6.09 to -0.46) |
| Honduras | 244 (143 to 390) | 1799.84 (1054.67 to 2873.52) | 10 (5 to 23) | 54.82 (26.92 to 126.23) | -95.99  (-98.31 to -88.94) | -10.56  (-13.85 to -7.13) |
| India | 47483 (19835 to 128478) | 2418.52 (1010.28 to 6543.9) | 2225 (746 to 5839) | 125.52 (42.11 to 329.39) | -95.31  (-98.82 to -81.7) | -9.4  (-10.04 to -8.76) |
| Indonesia | 2386 (547 to 7103) | 665.09 (152.4 to 1979.88) | 214 (39 to 742) | 73.63 (13.26 to 254.66) | -91.01  (-98.76 to -37.92) | -5.38  (-7.31 to -3.4) |
| Iran (Islamic Republic of) | 219 (128 to 344) | 163.24 (95.53 to 255.46) | 0 (0 to 0) | 0.01 (0 to 0.06) | -99.99  (-100 to -99.97) | -31.81  (-34.33 to -29.2) |
| Iraq | 3 (2 to 4) | 5.95 (4.1 to 8.35) | 0 (0 to 0) | 0 (0 to 0) | -100  (-100 to -100) | -75.27  (-81.21 to -67.45) |
| Jamaica | 0 (0 to 0) | 0 (0 to 0) | 0 (0 to 0) | 0.21 (0.05 to 0.63) | 0  (0 to 0) | 257.82  (151.93 to 408.21) |
| Jordan | 0 (0 to 0) | 0 (0 to 0) | 0 (0 to 0) | 0 (0 to 0) | 0  (0 to 0) | 0  (0 to 0) |
| Kazakhstan | 0 (0 to 0) | 0 (0 to 0) | 0 (0 to 0) | 0 (0 to 0) | 0  (0 to 0) | 0  (0 to 0) |
| Kenya | 16834 (9592 to 25091) | 22370.2 (12745.67 to 33341.73) | 9680 (3150 to 18913) | 9543.79 (3105.15 to 18646.1) | -42.5  (-75.77 to -6.91) | -5.28  (-6.41 to -4.14) |
| Kiribati | 0 (0 to 0) | 0 (0 to 0) | 0 (0 to 0) | 0 (0 to 0) | 0  (0 to 0) | 0  (0 to 0) |
| Kyrgyzstan | 0 (0 to 0) | 0 (0 to 0) | 0 (0 to 0) | 0 (0 to 0) | 0  (0 to 0) | -27.44  (-59.81 to 30.99) |
| Lao People's Democratic Republic | 40 (31 to 52) | 326.13 (252 to 423.39) | 3 (0 to 12) | 22.99 (3.81 to 91.54) | -92.63  (-98.83 to -68.47) | -11.43  (-14.01 to -8.77) |
| Lebanon | 0 (0 to 0) | 0 (0 to 0) | 0 (0 to 0) | 0 (0 to 0) | 0  (0 to 0) | 0  (0 to 0) |
| Lesotho | 0 (0 to 0) | 0 (0 to 0) | 0 (0 to 0) | 0 (0 to 0) | 0  (0 to 0) | 0  (0 to 0) |
| Liberia | 2896 (1809 to 3997) | 48294.17 (30166.88 to 66663.91) | 3714 (1476 to 5477) | 36694.23 (14580.14 to 54108.93) | 28.26  (-48.34 to 114.61) | -1.47  (-1.85 to -1.09) |
| Libya | 0 (0 to 0) | 0 (0 to 0) | 0 (0 to 0) | 0 (0 to 0) | 0  (0 to 0) | 0  (0 to 0) |
| Madagascar | 7929 (4855 to 12338) | 19869.1 (12167.22 to 30918.57) | 4790 (1971 to 8519) | 7427.49 (3056.97 to 13209.99) | -39.59  (-69.52 to -2.96) | -5.03  (-6.02 to -4.04) |
| Malawi | 14428 (8867 to 19946) | 41489.29 (25497.93 to 57356.93) | 7341 (3352 to 11578) | 17778.68 (8118.15 to 28040.43) | -49.12  (-72.25 to -13.84) | -3.1  (-3.67 to -2.54) |
| Malaysia | 38 (32 to 45) | 100.78 (83.43 to 119.51) | 1 (0 to 1) | 1.24 (0.5 to 2.71) | -98.65  (-99.45 to -97.02) | -16.84  (-18.17 to -15.5) |
| Maldives | 0 (0 to 0) | 0 (0 to 0) | 0 (0 to 0) | 0 (0 to 0) | 0  (0 to 0) | 0  (0 to 0) |
| Mali | 15033 (11613 to 19059) | 45761.27 (35349.02 to 58016.82) | 9935 (4450 to 16153) | 13778.35 (6171 to 22400.82) | -33.91  (-67.94 to 7.63) | -3.23  (-4.07 to -2.38) |
| Marshall Islands | 0 (0 to 0) | 0 (0 to 0) | 0 (0 to 0) | 0 (0 to 0) | 0  (0 to 0) | 0  (0 to 0) |
| Mauritania | 33 (19 to 52) | 489.21 (284.23 to 776.66) | 1083 (53 to 5488) | 13121.69 (646.04 to 66510.73) | 3219.61  (65.11 to 17285.25) | -0.23  (-3.16 to 2.8) |
| Mexico | 174 (132 to 228) | 92.74 (70.49 to 121.24) | 59 (41 to 83) | 36.78 (25.66 to 52.23) | -66.34  (-74.62 to -54.9) | -1.95  (-2.98 to -0.91) |
| Micronesia (Federated States of) | 0 (0 to 0) | 0 (0 to 0) | 0 (0 to 0) | 0 (0 to 0) | 0  (0 to 0) | 0  (0 to 0) |
| Mongolia | 0 (0 to 0) | 0 (0 to 0) | 0 (0 to 0) | 0 (0 to 0) | 0  (0 to 0) | 0  (0 to 0) |
| Montenegro | 0 (0 to 0) | 0 (0 to 0) | 0 (0 to 0) | 0 (0 to 0) | 0  (0 to 0) | 0  (0 to 0) |
| Morocco | 0 (0 to 1) | 0.71 (0.6 to 0.85) | 0 (0 to 0) | 0 (0 to 0) | -100  (-100 to -100) | -74.66  (-79.79 to -68.23) |
| Mozambique | 21117 (17523 to 25616) | 48367.23 (40136.16 to 58671.88) | 22927 (15908 to 30395) | 27129.42 (18823.12 to 35965.34) | 8.57  (-25.06 to 34.07) | -2.07  (-2.24 to -1.9) |
| Myanmar | 650 (405 to 1005) | 655.15 (407.86 to 1013.1) | 9 (0 to 53) | 10.97 (0 to 67.01) | -98.67  (-100 to -90.85) | -8.38  (-12.04 to -4.57) |
| Namibia | 364 (6 to 2293) | 9991.38 (174.81 to 62897.72) | 215 (1 to 1784) | 4550.34 (27.19 to 37740.48) | -40.94  (-99.63 to 3665.59) | -14.67  (-20.3 to -8.64) |
| Nepal | 61 (35 to 99) | 100.5 (56.9 to 163.06) | 0 (0 to 1) | 0.46 (0.07 to 1.88) | -99.65  (-99.95 to -98.53) | -14.52  (-15.95 to -13.08) |
| Nicaragua | 110 (68 to 163) | 1010.6 (629.18 to 1496.38) | 13 (4 to 30) | 129.74 (41.83 to 296.75) | -88.22  (-96.43 to -68.09) | -12.15  (-16.23 to -7.87) |
| Niger | 9964 (3374 to 16724) | 29979.75 (10150.87 to 50320.17) | 14864 (4629 to 27974) | 17209.86 (5359.1 to 32389.65) | 49.18  (-46.01 to 268.86) | -0.85  (-1.46 to -0.23) |
| Nigeria | 132368 (96040 to 167562) | 42977.04 (31182.01 to 54403.74) | 136057 (68319 to 208072) | 24088.15 (12095.45 to 36837.95) | 2.79  (-47.25 to 47.98) | -2.05  (-2.39 to -1.7) |
| North Macedonia | 0 (0 to 0) | 0 (0 to 0) | 0 (0 to 0) | 0 (0 to 0) | 0  (0 to 0) | 0  (0 to 0) |
| Pakistan | 7580 (1055 to 29584) | 2119.23 (295 to 8271.46) | 1407 (620 to 2878) | 282.51 (124.46 to 577.96) | -81.44  (-96.51 to 54.56) | -7.12  (-7.94 to -6.29) |
| Papua New Guinea | 1779 (626 to 3798) | 15866.5 (5582.48 to 33863.55) | 963 (240 to 2695) | 3897.28 (972.66 to 10908.85) | -45.89  (-88.82 to 125.53) | -7.21  (-8.06 to -6.34) |
| Paraguay | 7 (5 to 9) | 71.61 (54.8 to 90.48) | 3 (2 to 6) | 31.94 (17.92 to 66.16) | -55.76  (-73.74 to -4.27) | -2.12  (-3.69 to -0.52) |
| Peru | 286 (111 to 767) | 573.37 (222.47 to 1536.43) | 67 (28 to 197) | 137.97 (57.54 to 405.96) | -76.6  (-93.31 to -2.48) | -4.27  (-7.39 to -1.04) |
| Philippines | 239 (120 to 513) | 153.11 (76.8 to 328.07) | 3 (1 to 10) | 1.54 (0.31 to 4.97) | -98.7  (-99.78 to -94.69) | -15.88  (-17.19 to -14.54) |
| Republic of Moldova | 0 (0 to 0) | 0 (0 to 0) | 0 (0 to 0) | 0 (0 to 0) | 0  (0 to 0) | 0  (0 to 0) |
| Russian Federation | 0 (0 to 0) | 0 (0 to 0) | 0 (0 to 0) | 0 (0 to 0) | 0  (0 to 0) | 0  (0 to 0) |
| Rwanda | 4820 (1256 to 8962) | 20267.82 (5282.75 to 37683.03) | 1010 (209 to 2418) | 3796.55 (787.22 to 9087.82) | -79.04  (-95.43 to -44.5) | -7.41  (-8.88 to -5.91) |
| Saint Lucia | 0 (0 to 0) | 0 (0 to 0) | 0 (0 to 0) | 0 (0 to 0) | 0  (0 to 0) | 0  (0 to 0) |
| Saint Vincent and the Grenadines | 0 (0 to 0) | 0 (0 to 0) | 0 (0 to 0) | 0 (0 to 0) | 0  (0 to 0) | 0  (0 to 0) |
| Samoa | 0 (0 to 0) | 0 (0 to 0) | 0 (0 to 0) | 0 (0 to 0) | 0  (0 to 0) | 0  (0 to 0) |
| Senegal | 1471 (422 to 4089) | 5733.58 (1643.6 to 15939.31) | 1113 (117 to 4520) | 3187.74 (335.9 to 12942.96) | -24.33  (-93.41 to 445.32) | -4.37  (-6.08 to -2.62) |
| Serbia | 0 (0 to 0) | 0 (0 to 0) | 0 (0 to 0) | 0 (0 to 0) | 0  (0 to 0) | 0  (0 to 0) |
| Sierra Leone | 5381 (3141 to 7393) | 45986.17 (26846.03 to 63176.97) | 6649 (2180 to 11053) | 31709.19 (10398.05 to 52714.38) | 23.56  (-53.16 to 91.38) | -0.82  (-1.12 to -0.51) |
| Solomon Islands | 119 (17 to 391) | 11077.69 (1559.9 to 36311.31) | 31 (4 to 116) | 1966.75 (258 to 7380.36) | -73.99  (-97.2 to 107.52) | -4.85  (-5.8 to -3.89) |
| Somalia | 3900 (1357 to 6999) | 16932.83 (5894.36 to 30390.21) | 5478 (1469 to 12677) | 8393.13 (2250.41 to 19422.4) | 40.48  (-43.03 to 182.06) | -4.38  (-6.31 to -2.41) |
| South Africa | 59 (1 to 288) | 80.33 (1.77 to 392.95) | 62 (0 to 490) | 79.9 (0.62 to 628.57) | 5.65  (-99.23 to 4317.82) | -9.87  (-15.18 to -4.22) |
| South Sudan | 6800 (4539 to 9711) | 36287.34 (24224.93 to 51822.85) | 5619 (2439 to 10339) | 20738.72 (9000.97 to 38157.07) | -17.36  (-56.03 to 25.63) | -2.57  (-2.91 to -2.23) |
| Sri Lanka | 279 (235 to 330) | 1030.61 (866.36 to 1219.56) | 0 (0 to 0) | 0 (0 to 0) | -100  (-100 to -100) | -69.24  (-77.82 to -57.36) |
| Sudan | 5840 (2543 to 11130) | 8037.68 (3500.08 to 15317.9) | 3039 (1080 to 6086) | 3429.38 (1218.75 to 6868.08) | -47.97  (-76.77 to 0.7) | -4.75  (-5.75 to -3.75) |
| Suriname | 17 (0 to 137) | 2497.66 (37.6 to 20059.7) | 0 (0 to 0) | 51.6 (1.35 to 55.83) | -97.95  (-99.97 to -44.6) | -12.91  (-16.42 to -9.26) |
| Syrian Arab Republic | 0 (0 to 0) | 0.11 (0.08 to 0.15) | 0 (0 to 0) | 0 (0 to 0) | -100  (-100 to -100) | -75.81  (-81.18 to -68.9) |
| Tajikistan | 4847 (2 to 12933) | 31363.38 (16.08 to 83693.63) | 0 (0 to 0) | 0 (0 to 0) | -100  (-100 to -100) | -72.8  (-80.63 to -61.82) |
| Thailand | 150 (127 to 176) | 186.28 (158.32 to 218.38) | 0 (0 to 1) | 0.62 (0.02 to 1.12) | -99.82  (-99.99 to -99.65) | -15  (-16.35 to -13.63) |
| Timor-Leste | 649 (18 to 3523) | 24181.08 (656.49 to 131293.86) | 0 (0 to 0) | 0.25 (0 to 0.63) | -100  (-100 to -99.95) | -26.94  (-34.17 to -18.92) |
| Togo | 5481 (3347 to 7484) | 44870.96 (27397.01 to 61263.89) | 4571 (2164 to 6879) | 25718.71 (12173.04 to 38698.36) | -16.6  (-57.63 to 25.14) | -1.89  (-2.36 to -1.43) |
| Tonga | 0 (0 to 0) | 0 (0 to 0) | 0 (0 to 0) | 0 (0 to 0) | 0  (0 to 0) | 0  (0 to 0) |
| Tunisia | 0 (0 to 0) | 0 (0 to 0) | 0 (0 to 0) | 0 (0 to 0) | 0  (0 to 0) | 0  (0 to 0) |
| Turkey | 2 (1 to 4) | 1.54 (0.55 to 3.59) | 0 (0 to 0) | 0 (0 to 0) | -100  (-100 to -100) | -74.85  (-81.53 to -65.74) |
| Turkmenistan | 0 (0 to 0) | 0 (0 to 0) | 0 (0 to 0) | 0 (0 to 0) | 0  (0 to 0) | -39.21  (-63.48 to 1.2) |
| Tuvalu | 0 (0 to 0) | 0 (0 to 0) | 0 (0 to 0) | 0 (0 to 0) | 0  (0 to 0) | 0  (0 to 0) |
| Uganda | 31438 (24369 to 39714) | 45474.74 (35249.14 to 57446.4) | 27264 (10986 to 43309) | 22984.66 (9261.47 to 36511.16) | -13.28  (-60.86 to 28.6) | -2.44  (-2.94 to -1.94) |
| Ukraine | 0 (0 to 0) | 0 (0 to 0) | 0 (0 to 0) | 0 (0 to 0) | 0  (0 to 0) | 0  (0 to 0) |
| United Republic of Tanzania | 30113 (19430 to 38911) | 33111.31 (21364.48 to 42785.57) | 13968 (6005 to 23690) | 8998.15 (3868.4 to 15261.25) | -53.62  (-75.26 to -30.77) | -5.88  (-6.54 to -5.22) |
| Uzbekistan | 0 (0 to 0) | 0 (0 to 0.01) | 0 (0 to 0) | 0 (0 to 0) | -100  (-100 to -100) | -62.09  (-74.79 to -42.98) |
| Vanuatu | 217 (148 to 301) | 50250.1 (34250.99 to 69771.09) | 0 (0 to 1) | 37.56 (3.79 to 181.92) | -99.9  (-99.99 to -99.48) | -19.56  (-22.88 to -16.1) |
| Venezuela (Bolivarian Republic of) | 69 (53 to 89) | 159.98 (123.59 to 205.32) | 956 (68 to 4827) | 2657.02 (190.32 to 13418.32) | 1281.72  (-3.36 to 7013.94) | 6.91  (3.87 to 10.04) |
| Viet Nam | 28 (10 to 63) | 19.93 (7.34 to 45.78) | 1 (0 to 2) | 1.19 (0.4 to 2.44) | -95.6  (-98.84 to -85.31) | -11.27  (-12.83 to -9.67) |
| Yemen | 2978 (405 to 11030) | 5894.29 (801.21 to 21829.82) | 708 (158 to 2239) | 1004.88 (223.64 to 3178.27) | -76.24  (-96.55 to 121.38) | -9.98  (-11.91 to -8) |
| Zambia | 10523 (7260 to 15350) | 36622.3 (25265.35 to 53418.57) | 5860 (3084 to 10631) | 12672.9 (6668.89 to 22992.12) | -44.32  (-68.36 to -7.63) | -4.33  (-4.89 to -3.78) |
| Zimbabwe | 1016 (701 to 1433) | 3524.1 (2430.08 to 4967.58) | 454 (33 to 2213) | 1357.51 (97.33 to 6613.3) | -55.31  (-96.55 to 133.14) | -4.37  (-6.8 to -1.86) |

CI, confidence interval; EAPC, estimated annual percentage change; LMICs, low- and middle-income countries and territories; UI, uncertain interval.

## Supplementary Table S15. National burden and trends of incident cases and incidence rates (per 100,000 liveborn neonates) of neonatal sepsis and other neonatal infections in 131 LMICs between 1990 and 2019.

| **Location** | **Incident cases in 1990**  **(95% UI)** | **Incident rate in 1990**  **(95% UI)** | **Incident cases in 2019**  **(95% UI)** | **Incident rate in 2019**  **(95% UI)** | **Percent change**  **(%, 95% CI)** | **EAPC**  **(%, 95% CI)** |
| --- | --- | --- | --- | --- | --- | --- |
| Global | 5182419 (3658215 to 7099306) | 50195.03 (35432.14 to 68761.31) | 5924916 (4171504 to 8070144) | 57983.08 (40823.65 to 78976.96) | 14.33  (11.93 to 16.88) | 0.5  (0.48 to 0.52) |
| Afghanistan | 5020 (3877 to 6755) | 12974.15 (10021.08 to 17459.4) | 17393 (13075 to 22887) | 15551.5 (11691.18 to 20464.21) | 246.5  (204.91 to 288.93) | 0.7  (0.56 to 0.83) |
| Albania | 788 (576 to 1033) | 12113.28 (8864.42 to 15890.2) | 446 (315 to 607) | 16747.67 (11805.1 to 22769.18) | -43.34  (-51.66 to -33.61) | 0.94  (0.67 to 1.22) |
| Algeria | 10460 (7289 to 13858) | 18009.25 (12549.07 to 23859.64) | 19642 (14157 to 25429) | 29617.19 (21346.49 to 38343.85) | 87.78  (67.43 to 109.8) | 1.78  (1.63 to 1.93) |
| American Samoa | 31 (23 to 39) | 23748.77 (17555.84 to 30459.03) | 13 (10 to 17) | 16203.21 (12147.42 to 20773.72) | -57.83  (-62.88 to -51.53) | -1.28  (-1.72 to -0.85) |
| Angola | 14761 (10957 to 19764) | 38952.52 (28913.01 to 52155.67) | 35096 (25307 to 47201) | 42093.94 (30353.02 to 56612.69) | 137.76  (101.39 to 176.22) | 0.25  (0.22 to 0.28) |
| Armenia | 11177 (8588 to 14750) | 194621.16 (149544.43 to 256842.98) | 3563 (2658 to 4695) | 124589.99 (92930.39 to 164164.01) | -68.12  (-73.55 to -61.54) | -2.03  (-2.33 to -1.74) |
| Azerbaijan | 6997 (4863 to 9428) | 48918.11 (34002.53 to 65916.83) | 4767 (3331 to 6378) | 43857.95 (30643.78 to 58678.77) | -31.87  (-39.83 to -22.8) | -0.71  (-0.83 to -0.58) |
| Bangladesh | 218685 (150638 to 305534) | 74708.73 (51462.08 to 104378.86) | 271698 (197698 to 346829) | 133731.58 (97308.25 to 170711.69) | 24.24  (-4.1 to 51.87) | 2.11  (1.94 to 2.29) |
| Belarus | 12311 (9119 to 15754) | 114801.33 (85037.66 to 146900.84) | 7896 (5775 to 10184) | 100484.02 (73489.56 to 129611.11) | -35.87  (-45.34 to -25.18) | -0.74  (-0.87 to -0.6) |
| Belize | 248 (196 to 302) | 55604.32 (43896.17 to 67691.03) | 341 (272 to 410) | 58561.72 (46839.62 to 70588.21) | 37.47  (21.7 to 54.8) | 0.58  (0.37 to 0.8) |
| Benin | 10490 (7581 to 14249) | 58959.81 (42608.83 to 80085.36) | 19740 (14564 to 26270) | 52199.69 (38512.89 to 69465.45) | 88.18  (68.57 to 111.63) | -0.69  (-0.77 to -0.62) |
| Bhutan | 606 (448 to 804) | 36878.95 (27285.73 to 48920.39) | 704 (506 to 952) | 69508.11 (49918.99 to 93975.38) | 16.23  (0.19 to 29.41) | 2.58  (2.38 to 2.78) |
| Bolivia (Plurinational State of) | 17801 (13224 to 23543) | 97476.94 (72411.15 to 128918.18) | 20197 (14865 to 26930) | 82791.25 (60932.16 to 110389.61) | 13.46  (-1.28 to 28.58) | -0.54  (-0.67 to -0.41) |
| Bosnia and Herzegovina | 1426 (1020 to 1941) | 27572.63 (19730.64 to 37534.98) | 1021 (701 to 1426) | 49935.91 (34256.16 to 69710.56) | -28.35  (-42.48 to -10.93) | 2.6  (2.36 to 2.84) |
| Botswana | 1281 (912 to 1726) | 41681.04 (29654.67 to 56166.51) | 2008 (1382 to 2754) | 55170.09 (37980.25 to 75668.97) | 56.74  (35.41 to 78.04) | 0.91  (0.75 to 1.07) |
| Brazil | 137059 (91306 to 195585) | 52807.82 (35179.39 to 75357.48) | 158060 (103687 to 228118) | 67265.63 (44126.2 to 97080.46) | 15.32  (9.66 to 21.04) | 0.95  (0.84 to 1.06) |
| Bulgaria | 823 (648 to 1057) | 10968.53 (8631.73 to 14089.22) | 1944 (1440 to 2437) | 42463.62 (31455.88 to 53231.14) | 136.18  (96.15 to 179.94) | 6.25  (5.7 to 6.8) |
| Burkina Faso | 20491 (14292 to 29514) | 59778.58 (41695.83 to 86102.31) | 45558 (31797 to 63184) | 64590.8 (45080.83 to 89580.13) | 122.33  (94.41 to 157.22) | 0.19  (0.14 to 0.23) |
| Burundi | 11962 (8511 to 16308) | 60468.7 (43024.41 to 82438.79) | 23856 (17194 to 32721) | 68020.24 (49023.21 to 93294.06) | 99.44  (79.1 to 123.45) | 0.7  (0.5 to 0.9) |
| Cabo Verde | 389 (271 to 533) | 43711.34 (30470.81 to 59949.15) | 621 (448 to 869) | 76287.38 (55034.45 to 106720.41) | 59.76  (37.17 to 82.78) | 2.22  (2.11 to 2.34) |
| Cambodia | 22877 (16416 to 31438) | 69785.67 (50077.85 to 95899.26) | 21816 (15175 to 29828) | 78725.65 (54760.2 to 107638.09) | -4.64  (-17.32 to 7.46) | 0.54  (0.41 to 0.67) |
| Cameroon | 18386 (13313 to 24495) | 52765.45 (38205.51 to 70298.79) | 36753 (27087 to 49865) | 54068.2 (39848.06 to 73356.95) | 99.9  (79.32 to 124.6) | -0.03  (-0.19 to 0.13) |
| Central African Republic | 3380 (2478 to 4599) | 35932.68 (26342 to 48899) | 4880 (3553 to 6633) | 33113.4 (24110.7 to 45014.32) | 44.38  (29.34 to 60.65) | -0.05  (-0.14 to 0.04) |
| Chad | 11977 (8607 to 16746) | 50529.4 (36310.23 to 70645.83) | 25921 (18589 to 35371) | 43814.94 (31421.29 to 59788.68) | 116.42  (90.28 to 147.33) | -0.72  (-0.91 to -0.54) |
| China | 995417 (650630 to 1419973) | 54660.23 (35727.34 to 77973.44) | 1043607 (685407 to 1468586) | 91761.29 (60265.87 to 129128.46) | 4.84  (-7.26 to 18.53) | 2.07  (1.96 to 2.19) |
| Colombia | 39577 (33523 to 45098) | 56106.25 (47523.99 to 63932.45) | 67808 (52778 to 83656) | 112598.17 (87640.38 to 138914.12) | 71.33  (44.1 to 102.82) | 2.57  (2.22 to 2.92) |
| Comoros | 1032 (737 to 1391) | 70128.08 (50087.57 to 94517.13) | 922 (661 to 1264) | 74203.58 (53146.64 to 101649.1) | -10.6  (-21.11 to 0.65) | 0.42  (0.28 to 0.55) |
| Congo | 2563 (1903 to 3427) | 34140.26 (25349.42 to 45643.25) | 4215 (3061 to 5617) | 38808.51 (28185.18 to 51727.03) | 64.44  (44.78 to 83.73) | 0.74  (0.6 to 0.89) |
| Costa Rica | 4084 (3220 to 4926) | 66297.49 (52282.43 to 79969.47) | 1426 (1141 to 1740) | 28175.69 (22549.59 to 34396.82) | -65.09  (-69.6 to -60.07) | -3.03  (-3.31 to -2.74) |
| Côte d'Ivoire | 27034 (19808 to 37068) | 63451.03 (46491.36 to 87000.37) | 36560 (26971 to 49095) | 55189.87 (40714.06 to 74112.55) | 35.24  (21.31 to 54.06) | -0.67  (-0.77 to -0.57) |
| Cuba | 6670 (5327 to 8177) | 48339.22 (38608.73 to 59262.82) | 3445 (2670 to 4179) | 43149.87 (33448.23 to 52350.29) | -48.35  (-55.69 to -39.88) | -0.06  (-0.26 to 0.14) |
| Democratic People's Republic of Korea | 7853 (5791 to 10550) | 14476.82 (10675.16 to 19449.97) | 3871 (2966 to 5141) | 14816.15 (11352.74 to 19679.45) | -50.71  (-56.72 to -43.78) | 0.02  (-0.31 to 0.35) |
| Democratic Republic of the Congo | 46929 (34337 to 63941) | 33502.85 (24513.27 to 45648.43) | 82173 (58802 to 110570) | 37344.2 (26723.17 to 50249.72) | 75.1  (53.32 to 102.08) | 0.39  (0.33 to 0.44) |
| Djibouti | 1234 (855 to 1694) | 74378.51 (51525 to 102143.95) | 1946 (1354 to 2642) | 74593.85 (51903.63 to 101293.12) | 57.72  (38.39 to 79.22) | 0.23  (0.11 to 0.36) |
| Dominica | 105 (81 to 134) | 80979.35 (62478.94 to 103676.58) | 65 (50 to 81) | 103639.52 (79705.02 to 128897.29) | -37.72  (-46.38 to -29.3) | 1.31  (1.12 to 1.49) |
| Dominican Republic | 19936 (14881 to 24797) | 110662.75 (82604.43 to 137646.74) | 22726 (18177 to 26943) | 131639.5 (105287.92 to 156069.54) | 13.99  (-1.83 to 33.14) | 0.41  (0.12 to 0.71) |
| Ecuador | 21766 (17097 to 26061) | 96086.09 (75474.14 to 115049.21) | 17824 (13887 to 21730) | 67118.26 (52292.57 to 81828.21) | -18.11  (-30.51 to -4.03) | -1.87  (-2.37 to -1.36) |
| Egypt | 20928 (15791 to 27701) | 14730.93 (11115.14 to 19498.65) | 32812 (23559 to 42757) | 20378.84 (14632.11 to 26555.67) | 56.78  (32.36 to 81.22) | 0.84  (0.68 to 0.99) |
| El Salvador | 9327 (7013 to 11893) | 75301.45 (56623.71 to 96021.75) | 4774 (3638 to 6093) | 55806.41 (42526.81 to 71224.08) | -48.81  (-55.84 to -39.15) | -1.28  (-1.57 to -1) |
| Equatorial Guinea | 579 (421 to 790) | 33847.1 (24629.55 to 46214.3) | 1397 (1008 to 1884) | 47832.39 (34527.61 to 64519.56) | 141.37  (111.29 to 173.49) | 1.64  (1.43 to 1.86) |
| Eritrea | 5135 (3640 to 6910) | 55065.06 (39036.59 to 74099.32) | 9528 (6799 to 12740) | 63458.69 (45284.57 to 84855.91) | 85.55  (63.65 to 106.59) | 0.31  (0.25 to 0.37) |
| Eswatini | 963 (698 to 1307) | 40116.45 (29056.83 to 54422.77) | 989 (688 to 1324) | 44565.24 (30983.56 to 59630.21) | 2.71  (-8.33 to 15.69) | 0.32  (0.16 to 0.49) |
| Ethiopia | 119403 (80757 to 171863) | 64830.41 (43847.29 to 93313.7) | 203508 (133210 to 296840) | 73996.06 (48435.53 to 107931.44) | 70.44  (60.19 to 81.3) | 0.59  (0.48 to 0.69) |
| Fiji | 715 (517 to 929) | 49634.06 (35886.11 to 64521.87) | 524 (385 to 664) | 39411.26 (28956.02 to 49951.33) | -26.7  (-35.77 to -15.25) | -1.16  (-1.39 to -0.92) |
| Gabon | 1276 (901 to 1727) | 43207.43 (30497.32 to 58495.52) | 1855 (1312 to 2558) | 57595.46 (40710.43 to 79415.95) | 45.42  (27.06 to 65.4) | 1.12  (1.02 to 1.21) |
| Gambia | 2626 (1872 to 3632) | 72875.23 (51958.75 to 100791.3) | 3268 (2395 to 4403) | 60912.17 (44638.15 to 82057.42) | 24.47  (8.6 to 43.35) | -0.95  (-1.04 to -0.86) |
| Georgia | 5598 (4224 to 7487) | 88574.35 (66827.2 to 118456.32) | 2783 (2047 to 3685) | 80125.29 (58954 to 106119.01) | -50.29  (-59.37 to -38.51) | -0.88  (-1.52 to -0.24) |
| Ghana | 32182 (22679 to 43680) | 71208.35 (50181.89 to 96651.85) | 57359 (40922 to 76625) | 89292.52 (63704.23 to 119284.6) | 78.24  (54.73 to 103.06) | 0.69  (0.62 to 0.75) |
| Grenada | 77 (62 to 95) | 51473.91 (41247.62 to 62851.11) | 67 (54 to 80) | 63493.96 (51224.69 to 76131.73) | -13.48  (-23.06 to -3.25) | 0.75  (0.68 to 0.81) |
| Guatemala | 13686 (10616 to 16786) | 53875.89 (41791.31 to 66079.96) | 17765 (13607 to 22300) | 56771.55 (43483.7 to 71262.55) | 29.8  (4.58 to 58.47) | 0.38  (0.18 to 0.58) |
| Guinea | 12027 (8725 to 16264) | 51343.12 (37247.47 to 69428.92) | 16627 (12406 to 22675) | 46234.89 (34497.43 to 63053.68) | 38.24  (22.89 to 55.05) | -0.65  (-0.74 to -0.56) |
| Guinea-Bissau | 1777 (1318 to 2365) | 51336.61 (38059.53 to 68307.77) | 2129 (1575 to 2835) | 45482.61 (33641.11 to 60568.45) | 19.78  (5.2 to 33.63) | -0.61  (-0.7 to -0.51) |
| Guyana | 1125 (916 to 1344) | 67407.19 (54883.49 to 80492.15) | 675 (540 to 811) | 62285.22 (49788.04 to 74819.03) | -40.01  (-46.91 to -32.93) | -0.35  (-0.51 to -0.19) |
| Haiti | 5697 (4305 to 7945) | 30846.88 (23307.11 to 43016.2) | 8741 (6540 to 11691) | 35163.3 (26309.69 to 47028.79) | 53.42  (36.55 to 72.36) | 0.65  (0.55 to 0.75) |
| Honduras | 8892 (6265 to 12205) | 65565.34 (46192.45 to 89991.8) | 11761 (8302 to 15649) | 65949.38 (46553.95 to 87748.71) | 32.26  (18.69 to 48.57) | -0.01  (-0.18 to 0.17) |
| India | 623510 (429448 to 889166) | 31757.81 (21873.45 to 45288.68) | 704485 (473989 to 1011510) | 39742.02 (26739.06 to 57062.17) | 12.99  (4.09 to 21.48) | 0.32  (0.15 to 0.5) |
| Indonesia | 337108 (231303 to 452974) | 93961.56 (64470.68 to 126256.93) | 182897 (121941 to 265322) | 62796.34 (41867.46 to 91096.24) | -45.75  (-48.94 to -38.17) | -1.58  (-1.68 to -1.49) |
| Iran (Islamic Republic of) | 37457 (24283 to 54019) | 27856.93 (18059.28 to 40173.87) | 42578 (27449 to 61102) | 41518 (26765.93 to 59580.37) | 13.67  (8.87 to 18.43) | 1.37  (1.22 to 1.53) |
| Iraq | 20211 (13897 to 27511) | 38249.02 (26300.01 to 52062.71) | 39080 (28825 to 51458) | 53716.17 (39620.97 to 70729.74) | 93.36  (65.33 to 131.72) | 1.53  (1.26 to 1.79) |
| Jamaica | 2880 (2219 to 3588) | 67809.43 (52237.07 to 84481.54) | 2708 (2150 to 3241) | 98887.96 (78528.88 to 118371.44) | -5.98  (-19.18 to 8.82) | 1.81  (1.53 to 2.1) |
| Jordan | 1668 (1299 to 2112) | 16161.23 (12581.02 to 20465.3) | 4623 (3416 to 5925) | 24737.88 (18277.41 to 31703.44) | 177.13  (131.51 to 227.02) | 1.56  (1.48 to 1.65) |
| Kazakhstan | 17741 (12875 to 23009) | 65869.41 (47800.73 to 85426.49) | 14930 (11235 to 19163) | 55556.86 (41808.4 to 71308.99) | -15.84  (-30.24 to 4.05) | -0.47  (-0.6 to -0.34) |
| Kenya | 47717 (32289 to 69526) | 63408.68 (42906.71 to 92388.68) | 53975 (36313 to 77952) | 53214.01 (35800.9 to 76852.76) | 13.11  (9.95 to 16.18) | -0.77  (-0.88 to -0.66) |
| Kiribati | 42 (31 to 56) | 21895.37 (15903.02 to 28899.14) | 48 (35 to 64) | 21123.25 (15318.23 to 27926.86) | 13.7  (0.23 to 28.38) | -0.14  (-0.28 to -0.01) |
| Kyrgyzstan | 3556 (2595 to 4480) | 37379.83 (27280.61 to 47097) | 3628 (2872 to 4387) | 32846.09 (26001.51 to 39717.18) | 2.03  (-16.05 to 28.22) | -0.89  (-1.11 to -0.68) |
| Lao People's Democratic Republic | 10530 (7391 to 14198) | 85101.04 (59734.61 to 114743.86) | 10296 (7220 to 13823) | 79586.87 (55814.15 to 106853) | -2.22  (-12.54 to 10.48) | -0.23  (-0.28 to -0.19) |
| Lebanon | 1831 (1301 to 2471) | 22057.09 (15668.87 to 29761.67) | 3086 (2153 to 4185) | 38454.49 (26828.84 to 52162.41) | 68.52  (47.33 to 92.75) | 2.15  (2.03 to 2.26) |
| Lesotho | 1984 (1436 to 2666) | 43804.45 (31713.19 to 58853.31) | 1567 (1109 to 2115) | 44994.81 (31851.59 to 60726.1) | -21.02  (-29.63 to -11.89) | 0.01  (-0.09 to 0.11) |
| Liberia | 3245 (2364 to 4419) | 54110.34 (39419.02 to 73703.51) | 4812 (3511 to 6452) | 47540.23 (34682.85 to 63742.45) | 48.31  (30.56 to 69.09) | -0.65  (-0.84 to -0.47) |
| Libya | 1982 (1415 to 2646) | 17518.56 (12505.46 to 23382.28) | 1142 (819 to 1517) | 18390.75 (13193.44 to 24430.3) | -42.38  (-49.3 to -33.85) | 0.42  (0.22 to 0.61) |
| Madagascar | 25639 (17721 to 35039) | 64249.37 (44407.31 to 87806.09) | 39244 (27729 to 54216) | 60853.38 (42997.26 to 84069.67) | 53.06  (36.92 to 70.7) | -0.21  (-0.34 to -0.08) |
| Malawi | 24558 (16934 to 33615) | 70618.85 (48695.95 to 96666.2) | 27119 (19310 to 36847) | 65679.06 (46766.45 to 89238.96) | 10.43  (-2.29 to 24.16) | -0.05  (-0.17 to 0.07) |
| Malaysia | 34785 (24900 to 45549) | 92061.67 (65899.12 to 120547.28) | 31195 (22666 to 40552) | 75399.26 (54783.72 to 98014.69) | -10.32  (-21.51 to 1.99) | -0.51  (-0.66 to -0.37) |
| Maldives | 479 (340 to 628) | 72340.85 (51363.97 to 94874.23) | 535 (371 to 721) | 83470.88 (57857.65 to 112453.07) | 11.74  (-2.53 to 27.14) | 0.79  (0.6 to 0.98) |
| Mali | 18696 (14184 to 24659) | 56912.44 (43178.02 to 75063.31) | 48841 (36772 to 64202) | 67732.92 (50995.32 to 89035.64) | 161.23  (131.43 to 193.56) | 0.46  (0.32 to 0.59) |
| Marshall Islands | 30 (22 to 40) | 26341.41 (19096.16 to 35092.74) | 20 (14 to 26) | 20865.38 (15286.21 to 27530.36) | -35.5  (-42.33 to -27.79) | -0.88  (-0.97 to -0.79) |
| Mauritania | 3743 (2795 to 5036) | 56150.27 (41920.88 to 75541.88) | 5660 (4093 to 7640) | 68596.71 (49609.3 to 92590.42) | 51.2  (31.13 to 74.15) | 0.41  (0.31 to 0.51) |
| Mexico | 154255 (105264 to 221440) | 82167.91 (56071.4 to 117955.96) | 163022 (107861 to 230699) | 102312.91 (67693.64 to 144787.11) | 5.68  (-5.13 to 18.63) | 0.66  (0.42 to 0.9) |
| Micronesia (Federated States of) | 69 (49 to 91) | 26228.95 (18713.04 to 34540.78) | 36 (26 to 47) | 24257.68 (17497.42 to 32001.79) | -47.94  (-53.1 to -42.17) | -0.38  (-0.51 to -0.26) |
| Mongolia | 1875 (1336 to 2497) | 34050.23 (24258.72 to 45329.45) | 1777 (1257 to 2358) | 29161.51 (20626.36 to 38706.17) | -5.27  (-18.53 to 10.69) | -0.53  (-0.69 to -0.37) |
| Montenegro | 777 (571 to 1052) | 102187.47 (75023.93 to 138359.25) | 344 (251 to 463) | 67339.74 (49073.95 to 90681.92) | -55.73  (-62.2 to -47.7) | -1.41  (-1.55 to -1.27) |
| Morocco | 7056 (5595 to 9198) | 11870.23 (9411.42 to 15472.96) | 6924 (5185 to 9140) | 15166.68 (11358.34 to 20023) | -1.88  (-14.74 to 13.01) | 0.76  (0.62 to 0.91) |
| Mozambique | 36022 (25469 to 49892) | 82508.31 (58336.64 to 114275.38) | 65308 (46528 to 89987) | 77277.58 (55054.92 to 106479.59) | 81.3  (60.73 to 106.08) | -0.37  (-0.47 to -0.26) |
| Myanmar | 94730 (66675 to 121105) | 95460.15 (67188.5 to 122038.53) | 71448 (51141 to 95259) | 90644.01 (64881.72 to 120852.08) | -24.58  (-33.86 to -13.47) | -0.15  (-0.29 to -0.02) |
| Namibia | 1339 (961 to 1846) | 36713.11 (26343.83 to 50617.55) | 2587 (1797 to 3613) | 54713.24 (38002.4 to 76413.57) | 93.26  (65.13 to 118.46) | 1.65  (1.53 to 1.77) |
| Nepal | 27014 (18346 to 38167) | 44542.95 (30250.63 to 62933.04) | 26063 (17760 to 35843) | 56069.64 (38207.46 to 77111.15) | -3.52  (-15.3 to 11.58) | 1.21  (1.04 to 1.38) |
| Nicaragua | 12108 (9285 to 14854) | 111485.69 (85496.28 to 136772.65) | 10456 (7971 to 13213) | 104927.96 (79991.26 to 132594.46) | -13.64  (-31.51 to 9.76) | 0.32  (0.05 to 0.6) |
| Niger | 15863 (11079 to 22527) | 47728.15 (33335.95 to 67779.99) | 46038 (32693 to 62801) | 53304.69 (37853.5 to 72713.15) | 190.23  (154.19 to 234.54) | 0.39  (0.33 to 0.45) |
| Nigeria | 153787 (107876 to 224768) | 49931.03 (35024.76 to 72977.27) | 274193 (192011 to 404732) | 48544.43 (33994.46 to 71655.69) | 78.29  (73.92 to 83.09) | -0.17  (-0.25 to -0.1) |
| North Macedonia | 401 (296 to 530) | 15083.49 (11137.08 to 19949.41) | 1609 (1196 to 2169) | 94980.35 (70608.29 to 128082.17) | 301.18  (226.83 to 388.32) | 8.41  (7.73 to 9.09) |
| Pakistan | 184754 (122537 to 263049) | 51656.59 (34260.72 to 73547.46) | 331160 (221622 to 471216) | 66503.42 (44506 to 94629.42) | 79.24  (65.53 to 92.74) | 1.16  (1.05 to 1.27) |
| Papua New Guinea | 3335 (2416 to 4471) | 29736.75 (21546.98 to 39869.51) | 7009 (4950 to 9411) | 28370.86 (20039.23 to 38097.47) | 110.17  (89.31 to 136.32) | -0.33  (-0.45 to -0.22) |
| Paraguay | 6479 (5070 to 8005) | 66920.63 (52366.32 to 82688.26) | 2775 (2133 to 3348) | 28895.56 (22216.6 to 34863.45) | -57.17  (-63.77 to -49.47) | -3.65  (-4.02 to -3.29) |
| Peru | 66851 (54479 to 79560) | 133835.83 (109067.36 to 159278.88) | 64100 (49819 to 76797) | 131987.23 (102580.71 to 158131.16) | -4.11  (-16.26 to 6.87) | 0.29  (0.15 to 0.43) |
| Philippines | 223998 (158826 to 295955) | 143330.53 (101628.52 to 189373.53) | 215818 (148630 to 291321) | 107461.43 (74006.54 to 145056.25) | -3.65  (-10.81 to 8.74) | -1.08  (-1.28 to -0.89) |
| Republic of Moldova | 7847 (6684 to 8864) | 133893.27 (114055.7 to 151258.01) | 3158 (2733 to 3538) | 128880.45 (111554.1 to 144406.62) | -59.76  (-63.71 to -55.73) | -0.54  (-0.86 to -0.21) |
| Russian Federation | 116990 (78260 to 165589) | 79475.04 (53164.24 to 112489.31) | 99024 (65083 to 141393) | 77666.89 (51045.7 to 110897.79) | -15.36  (-18.95 to -11.84) | -0.21  (-0.33 to -0.09) |
| Rwanda | 14275 (10086 to 19790) | 60023.13 (42407.57 to 83213.82) | 20248 (14045 to 27472) | 76090.21 (52778.12 to 103236.05) | 41.84  (25.42 to 60.1) | 1.21  (1.05 to 1.37) |
| Saint Lucia | 112 (91 to 135) | 38997.05 (31776.95 to 47000.88) | 76 (62 to 93) | 56441.63 (45822.58 to 68428.9) | -31.74  (-38.52 to -24.23) | 1.65  (1.32 to 1.98) |
| Saint Vincent and the Grenadines | 143 (113 to 174) | 71077.18 (56357.56 to 86637.48) | 85 (67 to 104) | 74182.76 (57899.73 to 90495.79) | -40.2  (-47.05 to -31.55) | 0.36  (-0.05 to 0.77) |
| Samoa | 87 (60 to 117) | 33483.88 (23130.23 to 45117.05) | 82 (56 to 111) | 28151.3 (19317.25 to 38128.8) | -5.86  (-15.79 to 5.54) | -0.84  (-1.04 to -0.64) |
| Senegal | 15626 (10501 to 22188) | 60908.38 (40931.97 to 86485.2) | 16199 (11108 to 21939) | 46389.11 (31811.04 to 62828.28) | 3.66  (-9.84 to 19.72) | -1.27  (-1.37 to -1.16) |
| Serbia | 11192 (8231 to 15086) | 110814.44 (81497.63 to 149368.52) | 2424 (1775 to 3190) | 38487.61 (28182.81 to 50648.51) | -78.34  (-82 to -73.38) | -4.73  (-5.23 to -4.22) |
| Sierra Leone | 7923 (5730 to 10831) | 67707.37 (48967.05 to 92563.05) | 12262 (8938 to 16730) | 58479.81 (42627.94 to 79785.46) | 54.77  (33.18 to 77.33) | -0.77  (-0.9 to -0.64) |
| Solomon Islands | 261 (188 to 345) | 24246.51 (17462.95 to 32055.45) | 330 (236 to 442) | 20932.58 (14982.57 to 27990.49) | 26.5  (12.91 to 43.33) | -0.92  (-1.14 to -0.69) |
| Somalia | 15697 (10868 to 21656) | 68159.29 (47191.9 to 94036.41) | 35753 (24714 to 49167) | 54775.31 (37863.46 to 75326.84) | 127.77  (102.08 to 164.28) | -0.68  (-0.77 to -0.58) |
| South Africa | 34933 (23060 to 49812) | 47630.37 (31441.03 to 67916.84) | 45215 (30008 to 64852) | 58037.29 (38517.06 to 83242.44) | 29.43  (22.94 to 37.33) | 1.09  (0.83 to 1.35) |
| South Sudan | 12076 (8306 to 16871) | 64445.81 (44323.63 to 90031.4) | 15865 (11276 to 21674) | 58554.68 (41616.23 to 79993.89) | 31.38  (15.72 to 48.48) | -0.21  (-0.28 to -0.13) |
| Sri Lanka | 21720 (15395 to 28715) | 80171.64 (56826.16 to 105992.69) | 24161 (16544 to 32100) | 106819.23 (73142.33 to 141921.75) | 11.24  (-3.92 to 29.04) | 1.38  (1.21 to 1.56) |
| Sudan | 9317 (7061 to 12316) | 12822.72 (9718.15 to 16949.93) | 14178 (10337 to 18749) | 16000.93 (11665.98 to 21160.01) | 52.18  (30.84 to 76.4) | 0.58  (0.39 to 0.77) |
| Suriname | 636 (476 to 833) | 92907.84 (69472.3 to 121637.61) | 689 (499 to 878) | 101446.6 (73480.14 to 129307.74) | 8.34  (-4.46 to 22.64) | 0.52  (0.4 to 0.64) |
| Syrian Arab Republic | 7673 (5338 to 10210) | 20196.55 (14049.62 to 26873.41) | 3374 (2482 to 4405) | 19036.89 (14005.13 to 24851.19) | -56.02  (-61.05 to -49.33) | -0.07  (-0.17 to 0.02) |
| Tajikistan | 8671 (5920 to 11956) | 56114.02 (38307.9 to 77367.19) | 6969 (5002 to 9223) | 36195.65 (25977.33 to 47899.17) | -19.63  (-30.57 to -5.22) | -2.01  (-2.29 to -1.72) |
| Thailand | 55349 (38253 to 74611) | 68841.69 (47578.25 to 92799.71) | 49737 (36202 to 64776) | 111537.95 (81184.69 to 145264.79) | -10.14  (-25.66 to 6.22) | 2.51  (2.16 to 2.85) |
| Timor-Leste | 2210 (1525 to 2975) | 82346.55 (56816.66 to 110848.49) | 2163 (1488 to 2955) | 74519.36 (51260.48 to 101829.1) | -2.14  (-12.86 to 10.06) | -0.21  (-0.29 to -0.14) |
| Togo | 7657 (5193 to 10848) | 62678.17 (42508.81 to 88807.08) | 10063 (7214 to 13816) | 56611.52 (40583.47 to 77729.94) | 31.43  (14.48 to 49.28) | -0.53  (-0.65 to -0.42) |
| Tonga | 101 (71 to 136) | 51261.59 (35796.58 to 69033.04) | 90 (64 to 121) | 50867.78 (36095.5 to 68274.63) | -10.71  (-20.17 to -0.06) | -0.05  (-0.2 to 0.1) |
| Tunisia | 4195 (2915 to 5760) | 23453.69 (16297.46 to 32203.5) | 4011 (2848 to 5286) | 31553.26 (22401.56 to 41582.94) | -4.38  (-17.25 to 14.73) | 1.04  (1 to 1.08) |
| Turkey | 47144 (30761 to 66895) | 38053.39 (24829.67 to 53995.77) | 41545 (27947 to 58547) | 55817.44 (37548.64 to 78660.7) | -11.88  (-28.55 to 7.14) | 1.27  (1.2 to 1.34) |
| Turkmenistan | 2762 (2038 to 3581) | 27943.43 (20619.85 to 36227.92) | 3834 (2729 to 5088) | 45171.59 (32154.6 to 59947.72) | 38.79  (18.33 to 58.19) | 1.77  (1.55 to 2) |
| Tuvalu | 5 (4 to 7) | 23910.56 (17225.32 to 31896.64) | 4 (3 to 6) | 25418.37 (17919.15 to 33545.03) | -19.18  (-27.11 to -8.64) | -0.06  (-0.2 to 0.08) |
| Uganda | 46127 (32364 to 63486) | 66723.09 (46814 to 91832.84) | 83158 (58857 to 113332) | 70105.69 (49618.73 to 95543.59) | 80.28  (60.06 to 100.99) | 0.37  (0.21 to 0.53) |
| Ukraine | 63703 (42168 to 86970) | 127040.96 (84094.34 to 173439.62) | 26779 (17525 to 37963) | 90196.97 (59025.16 to 127865.95) | -57.96  (-63.87 to -50.54) | -0.95  (-1.12 to -0.77) |
| United Republic of Tanzania | 62559 (43283 to 84748) | 68787.48 (47592.52 to 93185.48) | 123847 (86778 to 171219) | 79782.93 (55903.01 to 110300.74) | 97.97  (72.6 to 124.75) | 0.65  (0.52 to 0.79) |
| Uzbekistan | 14824 (11040 to 18666) | 27499 (20478.55 to 34624.87) | 19319 (15241 to 23695) | 35613.09 (28096.55 to 43680.99) | 30.32  (11.84 to 55.41) | 0.83  (0.66 to 1) |
| Vanuatu | 95 (70 to 127) | 21940.47 (16115.4 to 29403.35) | 111 (81 to 148) | 18981.89 (13893.46 to 25256.44) | 17.17  (5.62 to 30.4) | -0.7  (-0.86 to -0.53) |
| Venezuela (Bolivarian Republic of) | 44361 (37019 to 52258) | 102583.55 (85606.23 to 120846.08) | 41965 (32826 to 50680) | 116648.53 (91243.89 to 140874.24) | -5.4  (-18.51 to 9.25) | 0.85  (0.64 to 1.05) |
| Viet Nam | 133024 (90167 to 179880) | 96385.35 (65332.74 to 130336.19) | 87792 (59028 to 119647) | 86473.73 (58142.03 to 117849.8) | -34  (-42.17 to -25.4) | -0.34  (-0.4 to -0.29) |
| Yemen | 7267 (5286 to 9623) | 14382.69 (10461.59 to 19044.8) | 10912 (7993 to 14335) | 15493.09 (11348.71 to 20353.33) | 50.15  (32.42 to 69.71) | 0.44  (0.36 to 0.52) |
| Zambia | 18542 (12881 to 25726) | 64527.28 (44826.86 to 89531.11) | 35179 (24457 to 47707) | 76082.98 (52894.32 to 103178.87) | 89.73  (67.77 to 117.86) | 0.76  (0.62 to 0.9) |
| Zimbabwe | 14615 (10110 to 19624) | 50676.61 (35055.23 to 68046.91) | 14971 (10650 to 19963) | 44746.59 (31831.23 to 59666.48) | 2.44  (-9.03 to 16.05) | -0.57  (-0.76 to -0.37) |

CI, confidence interval; EAPC, estimated annual percentage change; LMICs, low- and middle-income countries and territories; UI, uncertain interval.

## Supplementary Table S16. National burden and trends of incident cases and incidence rates (per 100,000 liveborn neonates) of otitis media in 131 LMICs between 1990 and 2019.

| **Location** | **Incident cases in 1990**  **(95% UI)** | **Incident rate in 1990**  **(95% UI)** | **Incident cases in 2019**  **(95% UI)** | **Incident rate in 2019**  **(95% UI)** | **Percent change**  **(%, 95% CI)** | **EAPC**  **(%, 95% CI)** |
| --- | --- | --- | --- | --- | --- | --- |
| Global | 1788377 (1046657 to 2829314) | 17321.58 (10137.55 to 27403.72) | 1883248 (1104001 to 2977182) | 18430.06 (10804.1 to 29135.64) | 5.3  (4.41 to 6.31) | 0.16  (0.11 to 0.21) |
| Afghanistan | 8246 (4797 to 13075) | 21314.22 (12399.51 to 33793.6) | 23839 (13868 to 37792) | 21315.27 (12399.98 to 33791.48) | 189.08  (188.99 to 189.2) | 0  (0 to 0) |
| Albania | 506 (290 to 812) | 7778.75 (4456.09 to 12487.09) | 207 (119 to 333) | 7782.45 (4457.85 to 12491.22) | -59  (-59.01 to -58.98) | 0  (0 to 0) |
| Algeria | 12382 (7204 to 19634) | 21318.39 (12403.44 to 33804.51) | 14138 (8225 to 22416) | 21317.67 (12402.77 to 33800) | 14.18  (14.14 to 14.22) | 0  (0 to 0) |
| American Samoa | 14 (8 to 22) | 10507.41 (6106.45 to 16709.36) | 8 (5 to 13) | 10504.25 (6103.84 to 16706.07) | -38.21  (-38.25 to -38.18) | 0  (0 to 0) |
| Angola | 7444 (4391 to 11799) | 19644.43 (11588.47 to 31137.57) | 16386 (9670 to 25972) | 19653.2 (11597.78 to 31151.13) | 120.12  (120.02 to 120.23) | 0  (0 to 0) |
| Armenia | 611 (356 to 977) | 10635.74 (6203.72 to 17018.83) | 305 (178 to 488) | 10665.66 (6223.98 to 17063.88) | -50.06  (-50.19 to -49.94) | 0.01  (0 to 0.01) |
| Azerbaijan | 1519 (887 to 2394) | 10616.6 (6202.21 to 16739.41) | 1158 (677 to 1825) | 10652.16 (6225.95 to 16790.35) | -23.75  (-23.99 to -23.54) | 0.01  (0.01 to 0.01) |
| Bangladesh | 63707 (37853 to 102171) | 21764.13 (12931.53 to 34904.39) | 44054 (25667 to 70750) | 21683.47 (12633.27 to 34823.52) | -30.85  (-40.59 to -21.06) | -0.02  (-0.02 to -0.01) |
| Belarus | 1140 (665 to 1824) | 10630.02 (6200.12 to 17011.73) | 835 (487 to 1337) | 10632.22 (6201.63 to 17014.63) | -26.71  (-26.73 to -26.7) | 0  (0 to 0) |
| Belize | 88 (52 to 139) | 19653.36 (11599 to 31150.64) | 114 (67 to 181) | 19650.78 (11597.59 to 31143.5) | 30.51  (30.48 to 30.53) | 0  (0 to 0) |
| Benin | 3495 (2062 to 5540) | 19644.47 (11588.85 to 31136.91) | 7431 (4385 to 11777) | 19648.7 (11594.41 to 31143.11) | 112.6  (112.55 to 112.65) | 0  (0 to 0) |
| Bhutan | 356 (208 to 572) | 21679.61 (12628.68 to 34813.31) | 220 (128 to 353) | 21681.33 (12632.25 to 34820.61) | -38.33  (-38.35 to -38.3) | 0  (0 to 0) |
| Bolivia (Plurinational State of) | 3588 (2117 to 5687) | 19646.39 (11592.16 to 31138.7) | 4794 (2829 to 7597) | 19649.5 (11596.88 to 31141.13) | 33.61  (33.58 to 33.64) | 0  (0 to 0) |
| Bosnia and Herzegovina | 401 (233 to 645) | 7747.38 (4499.35 to 12467.78) | 159 (92 to 255) | 7750.37 (4501.61 to 12470.71) | -60.42  (-60.44 to -60.41) | 0  (0 to 0) |
| Botswana | 604 (357 to 958) | 19660.77 (11601.77 to 31169.63) | 715 (422 to 1134) | 19655.84 (11599.36 to 31157.17) | 18.39  (18.35 to 18.43) | 0  (0 to 0) |
| Brazil | 58976 (35138 to 95424) | 22723.08 (13538.22 to 36766.16) | 53386 (31808 to 86383) | 22719.35 (13536.49 to 36762.28) | -9.48  (-9.5 to -9.45) | 0  (0 to 0) |
| Bulgaria | 583 (334 to 935) | 7767.34 (4449.01 to 12467.38) | 356 (204 to 571) | 7767.92 (4448.98 to 12467.59) | -38.99  (-39 to -38.98) | 0  (0 to 0) |
| Burkina Faso | 6735 (3974 to 10677) | 19649.5 (11593.15 to 31148.35) | 13860 (8178 to 21968) | 19649.7 (11595.17 to 31145.16) | 105.77  (105.74 to 105.81) | 0  (0 to 0) |
| Burundi | 3890 (2295 to 6167) | 19662.6 (11602.51 to 31177.15) | 6896 (4070 to 10934) | 19663.12 (11605.41 to 31174.31) | 77.3  (77.28 to 77.34) | 0  (0 to 0) |
| Cabo Verde | 175 (103 to 277) | 19652.6 (11598.53 to 31149.53) | 160 (94 to 254) | 19652.6 (11598.86 to 31147.07) | -8.46  (-8.47 to -8.45) | 0  (0 to 0) |
| Cambodia | 3505 (2004 to 5583) | 10692.91 (6112.37 to 17029.62) | 2963 (1693 to 4720) | 10693.27 (6110.34 to 17034.28) | -15.47  (-15.49 to -15.44) | 0  (0 to 0) |
| Cameroon | 6847 (4040 to 10854) | 19650.63 (11594.03 to 31149.62) | 13359 (7883 to 21176) | 19652.72 (11597.23 to 31151.7) | 95.1  (95.08 to 95.14) | 0  (0 to 0) |
| Central African Republic | 1849 (1091 to 2932) | 19658.21 (11598.86 to 31168.51) | 2897 (1709 to 4593) | 19657.3 (11598.33 to 31164.58) | 56.67  (56.64 to 56.7) | 0  (0 to 0) |
| Chad | 4658 (2748 to 7383) | 19649.62 (11592.6 to 31148.89) | 11625 (6859 to 18426) | 19649.62 (11594.58 to 31145.56) | 149.59  (149.55 to 149.63) | 0  (0 to 0) |
| China | 133957 (76433 to 216584) | 7355.85 (4197.07 to 11893.03) | 83583 (47690 to 134972) | 7349.18 (4193.25 to 11867.7) | -37.6  (-37.69 to -37.52) | 0  (0 to 0) |
| Colombia | 13860 (8179 to 21965) | 19647.79 (11594.66 to 31138.43) | 11830 (6982 to 18745) | 19643.5 (11593.66 to 31126.24) | -14.65  (-14.68 to -14.61) | 0  (0 to 0) |
| Comoros | 289 (171 to 458) | 19651.23 (11593.88 to 31151.92) | 244 (144 to 387) | 19653.2 (11597.35 to 31151.44) | -15.5  (-15.51 to -15.48) | 0  (0 to 0) |
| Congo | 1476 (871 to 2341) | 19666.49 (11605.48 to 31182.65) | 2136 (1261 to 3387) | 19668.54 (11608.27 to 31184.74) | 44.67  (44.66 to 44.69) | 0  (0 to 0) |
| Costa Rica | 1210 (710 to 1907) | 19646.59 (11524.47 to 30966.85) | 994 (583 to 1567) | 19649.51 (11526.61 to 30971.5) | -17.84  (-17.86 to -17.82) | 0  (0 to 0) |
| Côte d'Ivoire | 8371 (4939 to 13270) | 19648.14 (11591.18 to 31145.09) | 13016 (7680 to 20631) | 19648.68 (11593.32 to 31144.29) | 55.48  (55.47 to 55.51) | 0  (0 to 0) |
| Cuba | 2710 (1599 to 4293) | 19638.01 (11590.51 to 31113.39) | 1568 (925 to 2483) | 19635.65 (11589.37 to 31107.51) | -42.15  (-42.16 to -42.14) | 0  (0 to 0) |
| Democratic People's Republic of Korea | 3597 (2055 to 5703) | 6631 (3789.23 to 10512.91) | 1732 (990 to 2746) | 6631.11 (3790.92 to 10512.76) | -51.84  (-51.84 to -51.83) | 0  (0 to 0) |
| Democratic Republic of the Congo | 27529 (16243 to 43638) | 19653.27 (11596.24 to 31153.78) | 43260 (25530 to 68579) | 19659.94 (11602.6 to 31166.61) | 57.14  (57.1 to 57.2) | 0  (0 to 0) |
| Djibouti | 326 (192 to 516) | 19633.84 (11583.81 to 31110.9) | 512 (302 to 812) | 19636.44 (11586.95 to 31113.42) | 57.28  (57.26 to 57.31) | 0  (0 to 0) |
| Dominica | 25 (15 to 40) | 19650.52 (11597.48 to 31142.78) | 12 (7 to 20) | 19651.02 (11597.5 to 31144.57) | -51.34  (-51.34 to -51.33) | 0  (0 to 0) |
| Dominican Republic | 3540 (2089 to 5611) | 19650.67 (11596.28 to 31146) | 3392 (2002 to 5375) | 19647.66 (11594.53 to 31137.61) | -4.19  (-4.21 to -4.16) | 0  (0 to 0) |
| Ecuador | 4451 (2627 to 7054) | 19649.03 (11595.61 to 31141.17) | 5218 (3080 to 8269) | 19648.63 (11596.39 to 31138.12) | 17.23  (17.22 to 17.25) | 0  (0 to 0) |
| Egypt | 30286 (17621 to 48023) | 21317.83 (12402.9 to 33802.73) | 34325 (19971 to 54424) | 21318.8 (12403.71 to 33802.32) | 13.34  (13.31 to 13.37) | 0  (0 to 0) |
| El Salvador | 2434 (1436 to 3858) | 19651.72 (11597.28 to 31148.07) | 1680 (992 to 2663) | 19642.32 (11593.14 to 31123.13) | -30.96  (-31.01 to -30.91) | 0  (0 to 0) |
| Equatorial Guinea | 336 (198 to 533) | 19645.83 (11590.47 to 31139.71) | 574 (339 to 909) | 19642.69 (11591.02 to 31127.3) | 70.77  (70.7 to 70.84) | 0  (0 to 0) |
| Eritrea | 1832 (1081 to 2904) | 19650.63 (11596.74 to 31146.5) | 2951 (1742 to 4678) | 19655.31 (11600.18 to 31155.43) | 61.05  (61 to 61.1) | 0  (0 to 0) |
| Eswatini | 472 (279 to 748) | 19660.73 (11601.9 to 31168.66) | 436 (257 to 691) | 19649.9 (11596.04 to 31143.07) | -7.6  (-7.67 to -7.53) | 0  (0 to 0) |
| Ethiopia | 41828 (24913 to 67673) | 22710.89 (13526.88 to 36743.49) | 62472 (37216 to 101081) | 22715.08 (13531.9 to 36753.24) | 49.35  (49.31 to 49.41) | 0  (0 to 0) |
| Fiji | 151 (88 to 241) | 10506.91 (6106.6 to 16708.43) | 140 (81 to 222) | 10506.53 (6106.01 to 16708.22) | -7.69  (-7.71 to -7.69) | 0  (0 to 0) |
| Gabon | 581 (343 to 921) | 19661.22 (11602.01 to 31170.43) | 633 (374 to 1004) | 19658.32 (11601.37 to 31161.59) | 9.07  (9.04 to 9.11) | 0  (0 to 0) |
| Gambia | 708 (418 to 1123) | 19654.77 (11597.1 to 31158.39) | 1055 (622 to 1671) | 19653.54 (11599.12 to 31151.75) | 48.91  (48.88 to 48.95) | 0  (0 to 0) |
| Georgia | 672 (392 to 1075) | 10631.04 (6200.64 to 17011.95) | 369 (215 to 591) | 10636.8 (6204.66 to 17021.36) | -45.02  (-45.05 to -45) | 0  (-0.01 to 0) |
| Ghana | 8879 (5239 to 14073) | 19646.13 (11591.42 to 31138.53) | 12621 (7448 to 20003) | 19647.45 (11593.91 to 31138.99) | 42.15  (42.13 to 42.17) | 0  (0 to 0) |
| Grenada | 30 (17 to 47) | 19659.79 (11604.54 to 31164.18) | 21 (12 to 33) | 19647.4 (11595.73 to 31135.07) | -29.9  (-29.96 to -29.85) | 0  (0 to 0) |
| Guatemala | 4984 (2958 to 7912) | 19618.67 (11643.11 to 31146.48) | 6141 (3645 to 9750) | 19624.89 (11648.65 to 31157.71) | 23.22  (23.18 to 23.27) | 0  (0 to 0) |
| Guinea | 4603 (2715 to 7296) | 19647.67 (11589.79 to 31146.31) | 7066 (4170 to 11200) | 19649.34 (11594.79 to 31144.99) | 53.53  (53.5 to 53.58) | 0  (0 to 0) |
| Guinea-Bissau | 680 (401 to 1078) | 19645.2 (11588.94 to 31140.84) | 920 (543 to 1458) | 19651.71 (11595.99 to 31150.45) | 35.24  (35.2 to 35.29) | 0  (0 to 0) |
| Guyana | 328 (194 to 520) | 19620.76 (11644.41 to 31152.35) | 213 (126 to 338) | 19619.37 (11644.18 to 31146.19) | -35.09  (-35.1 to -35.07) | 0  (0 to 0) |
| Haiti | 3625 (2152 to 5757) | 19628 (11648.8 to 31171.44) | 4878 (2895 to 7746) | 19624.02 (11646.7 to 31159.47) | 34.56  (34.51 to 34.61) | 0  (0 to 0) |
| Honduras | 2660 (1579 to 4223) | 19615.02 (11640.61 to 31137.46) | 3498 (2076 to 5552) | 19613.88 (11640.29 to 31132.32) | 31.48  (31.46 to 31.5) | 0  (0 to 0) |
| India | 414116 (240217 to 662748) | 21092.55 (12235.19 to 33756.32) | 373703 (216744 to 597960) | 21081.64 (12227.15 to 33732.65) | -9.76  (-9.86 to -9.68) | -0.01  (-0.03 to 0) |
| Indonesia | 44211 (25625 to 70710) | 12323 (7142.29 to 19708.95) | 35888 (20801 to 57398) | 12321.7 (7141.96 to 19707.07) | -18.83  (-18.85 to -18.81) | 0  (0 to 0) |
| Iran (Islamic Republic of) | 33924 (20104 to 55203) | 25228.98 (14951.49 to 41053.99) | 25875 (15335 to 42095) | 25230.65 (14953.26 to 41046.62) | -23.73  (-23.75 to -23.7) | 0  (0 to 0) |
| Iraq | 11264 (6554 to 17861) | 21317.18 (12402.52 to 33801.05) | 15510 (9024 to 24592) | 21318.44 (12403.47 to 33802.45) | 37.69  (37.67 to 37.72) | 0  (0 to 0) |
| Jamaica | 835 (493 to 1323) | 19655.3 (11599.98 to 31154.17) | 538 (318 to 853) | 19653.4 (11599.52 to 31149.65) | -35.54  (-35.55 to -35.53) | 0  (0 to 0) |
| Jordan | 2198 (1282 to 3487) | 21296.56 (12425.03 to 33782.19) | 3980 (2322 to 6314) | 21297.94 (12425.54 to 33785.25) | 81.06  (81.04 to 81.09) | 0  (0 to 0) |
| Kazakhstan | 2857 (1669 to 4506) | 10607.77 (6196.81 to 16731.35) | 2852 (1666 to 4498) | 10613.83 (6201.02 to 16736.32) | -0.17  (-0.22 to -0.12) | 0  (0 to 0) |
| Kenya | 17107 (10193 to 27678) | 22732.18 (13544.8 to 36779.39) | 23048 (13732 to 37293) | 22723.39 (13538.69 to 36766.93) | 34.73  (34.67 to 34.81) | 0  (0 to 0) |
| Kiribati | 20 (12 to 32) | 10507.77 (6106.53 to 16709.07) | 24 (14 to 38) | 10502.86 (6102.73 to 16704.05) | 17.8  (17.68 to 17.9) | 0  (0 to 0) |
| Kyrgyzstan | 1009 (589 to 1592) | 10605.84 (6195.27 to 16730.67) | 1173 (685 to 1849) | 10615.74 (6202.12 to 16738.15) | 16.22  (16.13 to 16.32) | 0  (0 to 0) |
| Lao People's Democratic Republic | 1323 (756 to 2107) | 10693.84 (6111.23 to 17030.35) | 1383 (790 to 2204) | 10693.31 (6109.63 to 17034.26) | 4.54  (4.51 to 4.58) | 0  (0 to 0) |
| Lebanon | 1768 (1031 to 2804) | 21295.93 (12425.29 to 33780.43) | 1709 (997 to 2711) | 21296.5 (12428.17 to 33780.45) | -3.34  (-3.35 to -3.32) | 0  (0 to 0) |
| Lesotho | 890 (525 to 1412) | 19660.31 (11601.14 to 31170.27) | 685 (404 to 1086) | 19660.83 (11601.91 to 31169.31) | -23.1  (-23.11 to -23.09) | 0  (0 to 0) |
| Liberia | 1178 (695 to 1867) | 19645.15 (11590.33 to 31139.23) | 1988 (1173 to 3151) | 19642.27 (11590.66 to 31127.49) | 68.78  (68.72 to 68.86) | 0  (0 to 0) |
| Libya | 2413 (1404 to 3827) | 21322.66 (12407.47 to 33818.16) | 1324 (770 to 2099) | 21318.28 (12403.38 to 33800.73) | -45.13  (-45.18 to -45.08) | 0  (0 to 0) |
| Madagascar | 7841 (4627 to 12429) | 19649.96 (11594.5 to 31147.36) | 12673 (7478 to 20086) | 19650.8 (11596.34 to 31145.56) | 61.61  (61.6 to 61.64) | 0  (0 to 0) |
| Malawi | 6838 (4034 to 10843) | 19663.23 (11601.69 to 31179.48) | 8122 (4793 to 12879) | 19669.78 (11608.91 to 31190.27) | 18.78  (18.74 to 18.82) | 0  (0 to 0) |
| Malaysia | 4039 (2308 to 6434) | 10689.09 (6109.34 to 17027.22) | 4422 (2527 to 7044) | 10687.02 (6108.83 to 17024.88) | 9.48  (9.45 to 9.5) | 0  (0 to 0) |
| Maldives | 71 (40 to 113) | 10694.77 (6109.13 to 17035.83) | 69 (39 to 109) | 10689.14 (6109.08 to 17028.26) | -3.21  (-3.28 to -3.15) | 0  (0 to 0) |
| Mali | 6453 (3806 to 10229) | 19643.52 (11586.62 to 31138.72) | 14167 (8358 to 22454) | 19646.36 (11591.31 to 31140.03) | 119.53  (119.49 to 119.59) | 0  (0 to 0) |
| Marshall Islands | 12 (7 to 19) | 10505.56 (6104.97 to 16706.81) | 10 (6 to 16) | 10505.52 (6105.37 to 16706.93) | -18.57  (-18.59 to -18.57) | 0  (0 to 0) |
| Mauritania | 1310 (773 to 2076) | 19645.39 (11590.43 to 31138.39) | 1621 (957 to 2570) | 19649.92 (11596.08 to 31144.65) | 23.79  (23.76 to 23.83) | 0  (0 to 0) |
| Mexico | 42676 (25430 to 69050) | 22732.57 (13545.71 to 36781.22) | 36206 (21573 to 58585) | 22722.99 (13539.34 to 36768.02) | -15.16  (-15.21 to -15.11) | 0  (0 to 0) |
| Micronesia (Federated States of) | 28 (16 to 44) | 10505.26 (6104.88 to 16706.53) | 16 (9 to 25) | 10505.6 (6105.73 to 16706.96) | -43.71  (-43.72 to -43.7) | 0  (0 to 0) |
| Mongolia | 584 (341 to 921) | 10601.83 (6192.81 to 16727.08) | 646 (377 to 1019) | 10605.26 (6195.4 to 16729.05) | 10.64  (10.6 to 10.68) | 0  (0 to 0) |
| Montenegro | 59 (34 to 95) | 7752.91 (4502.94 to 12474.62) | 40 (23 to 64) | 7761.65 (4508.11 to 12487.71) | -32.75  (-32.8 to -32.7) | 0  (0 to 0) |
| Morocco | 12674 (7374 to 20100) | 21320.22 (12404.84 to 33810.97) | 9733 (5663 to 15433) | 21319.99 (12404.82 to 33806.83) | -23.21  (-23.24 to -23.18) | 0  (0 to 0) |
| Mozambique | 8584 (5065 to 13612) | 19662.38 (11601.28 to 31177.1) | 16617 (9807 to 26345) | 19662.6 (11604.39 to 31173.38) | 93.57  (93.53 to 93.62) | 0  (0 to 0) |
| Myanmar | 10613 (6067 to 16902) | 10694.63 (6114.1 to 17032.42) | 8428 (4816 to 13425) | 10691.88 (6109.37 to 17031.32) | -20.59  (-20.63 to -20.57) | 0  (0 to 0) |
| Namibia | 717 (423 to 1137) | 19666.84 (11605.75 to 31183.19) | 930 (549 to 1475) | 19672.64 (11610.56 to 31193.86) | 29.71  (29.67 to 29.76) | 0  (0 to 0) |
| Nepal | 16034 (9488 to 25527) | 26438.57 (15643.83 to 42091.93) | 12288 (7275 to 19561) | 26435.47 (15650.25 to 42081.7) | -23.36  (-23.4 to -23.32) | 0  (0 to 0) |
| Nicaragua | 2134 (1259 to 3383) | 19651.09 (11596.77 to 31146.86) | 1958 (1156 to 3103) | 19650.04 (11598.6 to 31141.35) | -8.25  (-8.27 to -8.23) | 0  (0 to 0) |
| Niger | 6529 (3852 to 10349) | 19645.51 (11590.71 to 31138.49) | 16971 (10015 to 26898) | 19649.3 (11595.53 to 31143.96) | 159.92  (159.87 to 159.98) | 0  (0 to 0) |
| Nigeria | 70013 (41713 to 113272) | 22731.63 (13543.14 to 36776.91) | 128347 (76465 to 207663) | 22723.12 (13537.66 to 36765.6) | 83.32  (83.23 to 83.43) | 0  (0 to 0) |
| North Macedonia | 206 (119 to 330) | 7756.77 (4463.78 to 12407.7) | 131 (76 to 210) | 7755.7 (4462.87 to 12407.69) | -36.3  (-36.31 to -36.29) | 0  (0 to 0) |
| Pakistan | 92877 (54988 to 148424) | 25968.08 (15374.37 to 41498.74) | 129279 (76534 to 206645) | 25961.72 (15369.5 to 41498.26) | 39.19  (39.13 to 39.28) | 0  (0 to 0) |
| Papua New Guinea | 1178 (685 to 1874) | 10505.46 (6104.4 to 16706.79) | 2595 (1508 to 4127) | 10504.52 (6104.13 to 16706) | 120.27  (120.23 to 120.3) | 0  (0 to 0) |
| Paraguay | 1901 (1122 to 3013) | 19640.98 (11591.13 to 31122.69) | 1886 (1113 to 2987) | 19635.02 (11588.37 to 31107.28) | -0.84  (-0.88 to -0.79) | 0  (0 to 0) |
| Peru | 9815 (5792 to 15557) | 19650.01 (11595.24 to 31145.3) | 9542 (5632 to 15121) | 19647.37 (11595.94 to 31134.77) | -2.78  (-2.81 to -2.75) | 0  (0 to 0) |
| Philippines | 19250 (11159 to 30793) | 12317.83 (7140.46 to 19703.72) | 24736 (14340 to 39568) | 12316.44 (7140.01 to 19702.14) | 28.49  (28.47 to 28.52) | 0  (0 to 0) |
| Republic of Moldova | 622 (363 to 981) | 10608.37 (6197.23 to 16731.8) | 260 (152 to 410) | 10614.51 (6201.39 to 16736.84) | -58.17  (-58.19 to -58.15) | 0  (0 to 0) |
| Russian Federation | 18189 (10415 to 28500) | 12356.48 (7075.01 to 19360.76) | 15759 (9023 to 24699) | 12360.31 (7077.14 to 19371.69) | -13.36  (-13.41 to -13.3) | 0  (0 to 0) |
| Rwanda | 4676 (2759 to 7414) | 19662.37 (11601.83 to 31175.97) | 5232 (3088 to 8295) | 19662.6 (11604.06 to 31172.44) | 11.89  (11.87 to 11.92) | 0  (0 to 0) |
| Saint Lucia | 56 (33 to 89) | 19658.39 (11602.77 to 31161) | 27 (16 to 42) | 19654.13 (11600.18 to 31150.87) | -52.85  (-52.86 to -52.83) | 0  (0 to 0) |
| Saint Vincent and the Grenadines | 39 (23 to 63) | 19654.4 (11598.9 to 31152.9) | 23 (13 to 36) | 19655.48 (11600.81 to 31154.03) | -42.7  (-42.71 to -42.7) | 0  (0 to 0) |
| Samoa | 27 (16 to 44) | 10505.65 (6105.06 to 16707.04) | 31 (18 to 49) | 10502.18 (6102.01 to 16703.71) | 11.93  (11.87 to 12) | 0  (0 to 0) |
| Senegal | 5043 (2976 to 7994) | 19656.03 (11598.33 to 31160.94) | 6863 (4050 to 10878) | 19652.45 (11596.92 to 31150.53) | 36.09  (36.04 to 36.13) | 0  (0 to 0) |
| Serbia | 777 (447 to 1243) | 7688.73 (4423.18 to 12310.04) | 488 (281 to 781) | 7750.12 (4457.95 to 12399.98) | -37.14  (-37.52 to -36.8) | 0.03  (0.03 to 0.03) |
| Sierra Leone | 2299 (1357 to 3646) | 19651.58 (11592.81 to 31155.12) | 4122 (2432 to 6534) | 19656.11 (11599.88 to 31159.86) | 79.24  (79.2 to 79.29) | 0  (0 to 0) |
| Solomon Islands | 113 (66 to 180) | 10504.08 (6103.56 to 16705.26) | 166 (96 to 264) | 10503.83 (6103.66 to 16704.86) | 46.52  (46.51 to 46.55) | 0  (0 to 0) |
| Somalia | 4522 (2668 to 7166) | 19634.83 (11585.09 to 31114.42) | 12822 (7566 to 20321) | 19644.34 (11592.02 to 31133.87) | 183.56  (183.42 to 183.71) | 0  (0 to 0) |
| South Africa | 16673 (9935 to 26977) | 22733.7 (13546.2 to 36782.42) | 17711 (10554 to 28657) | 22733.71 (13546.68 to 36783.26) | 6.22  (6.22 to 6.23) | 0  (0 to 0) |
| South Sudan | 3681 (2172 to 5834) | 19644.17 (11588.55 to 31135.62) | 5323 (3141 to 8437) | 19645.86 (11591.04 to 31137.3) | 44.61  (44.59 to 44.63) | 0  (0 to 0) |
| Sri Lanka | 2898 (1655 to 4616) | 10695.37 (6109.61 to 17038.21) | 2418 (1382 to 3852) | 10691.13 (6108.56 to 17032.6) | -16.55  (-16.59 to -16.51) | 0  (0 to 0) |
| Sudan | 15481 (9004 to 24536) | 21306.81 (12392.75 to 33769.09) | 18889 (10989 to 29949) | 21317.51 (12402.36 to 33799.77) | 22.01  (21.88 to 22.18) | 0  (0 to 0) |
| Suriname | 135 (79 to 213) | 19663.37 (11604.07 to 31174.79) | 134 (79 to 212) | 19655.11 (11599.6 to 31153.95) | -0.82  (-0.88 to -0.76) | 0  (0 to 0) |
| Syrian Arab Republic | 8099 (4712 to 12841) | 21317.25 (12402.32 to 33799.01) | 3779 (2198 to 5991) | 21317.55 (12402.42 to 33797.46) | -53.34  (-53.35 to -53.33) | 0  (0 to 0) |
| Tajikistan | 1639 (958 to 2586) | 10608.69 (6197.46 to 16732.83) | 2044 (1194 to 3223) | 10613.61 (6200.63 to 16736.86) | 24.66  (24.61 to 24.71) | 0  (0 to 0) |
| Thailand | 8432 (4943 to 13037) | 10487.73 (6148.37 to 16215.8) | 4767 (2725 to 7594) | 10689.29 (6109.92 to 17029.1) | -43.47  (-51.87 to -33.41) | 0.09  (0.08 to 0.11) |
| Timor-Leste | 287 (164 to 457) | 10696.44 (6110.53 to 17037.9) | 310 (177 to 494) | 10693.03 (6108.76 to 17033.97) | 8.1  (8.06 to 8.14) | 0  (0 to 0) |
| Togo | 2400 (1416 to 3805) | 19650.72 (11593.52 to 31150.62) | 3493 (2062 to 5537) | 19651.9 (11598.05 to 31149.08) | 45.52  (45.5 to 45.55) | 0  (0 to 0) |
| Tonga | 21 (12 to 33) | 10504.8 (6104.67 to 16706.18) | 19 (11 to 30) | 10503.44 (6103.38 to 16704.93) | -10.03  (-10.05 to -10.02) | 0  (0 to 0) |
| Tunisia | 3812 (2218 to 6044) | 21314.58 (12399.86 to 33791.14) | 2709 (1576 to 4295) | 21313.82 (12399.31 to 33786.87) | -28.93  (-28.95 to -28.9) | 0  (0 to 0) |
| Turkey | 26409 (15365 to 41875) | 21316.73 (12402.08 to 33800.31) | 15867 (9231 to 25157) | 21317.69 (12402.84 to 33800.27) | -39.92  (-39.93 to -39.9) | 0  (0 to 0) |
| Turkmenistan | 1048 (612 to 1654) | 10603.6 (6193.86 to 16728.23) | 901 (526 to 1420) | 10612.56 (6200.1 to 16735.37) | -14.07  (-14.14 to -14.01) | 0  (0 to 0) |
| Tuvalu | 2 (1 to 4) | 10504.86 (6104 to 16705.98) | 2 (1 to 3) | 10504.08 (6104.14 to 16705.47) | -23.98  (-24 to -23.97) | 0  (0 to 0) |
| Uganda | 13593 (8021 to 21552) | 19662.66 (11602.77 to 31174.91) | 23321 (13763 to 36973) | 19661 (11602.93 to 31169.84) | 71.57  (71.53 to 71.6) | 0  (0 to 0) |
| Ukraine | 6173 (3607 to 9766) | 12310.6 (7192.41 to 19475.04) | 3657 (2138 to 5788) | 12316.05 (7202.1 to 19494.56) | -40.76  (-40.84 to -40.7) | 0  (0 to 0) |
| United Republic of Tanzania | 17881 (10551 to 28352) | 19661.69 (11601.37 to 31174.31) | 30526 (18015 to 48398) | 19664.97 (11605.28 to 31178.43) | 70.71  (70.68 to 70.75) | 0  (0 to 0) |
| Uzbekistan | 5715 (3338 to 9017) | 10601.26 (6192.47 to 16726.17) | 5762 (3367 to 9083) | 10622.16 (6206.31 to 16743.41) | 0.83  (0.64 to 1) | 0.01  (0.01 to 0.01) |
| Vanuatu | 45 (26 to 72) | 10505.4 (6104.99 to 16706.64) | 61 (36 to 98) | 10505.92 (6105.62 to 16707.24) | 35.44  (35.43 to 35.46) | 0  (0 to 0) |
| Venezuela (Bolivarian Republic of) | 8495 (5013 to 13461) | 19643.64 (11591.52 to 31129.17) | 7066 (4170 to 11196) | 19641.14 (11591.53 to 31121.61) | -16.82  (-16.84 to -16.79) | 0  (0 to 0) |
| Viet Nam | 14758 (8436 to 23509) | 10693.34 (6112.54 to 17033.93) | 10848 (6203 to 17281) | 10685.52 (6110.17 to 17021.54) | -26.49  (-26.56 to -26.43) | 0  (0 to 0) |
| Yemen | 10771 (6267 to 17079) | 21316.9 (12401.8 to 33800.63) | 15016 (8736 to 23809) | 21319.35 (12403.95 to 33804.63) | 39.4  (39.38 to 39.44) | 0  (0 to 0) |
| Zambia | 5651 (3335 to 8961) | 19667.69 (11606.27 to 31185.66) | 9094 (5367 to 14418) | 19667.27 (11607.59 to 31182.89) | 60.91  (60.89 to 60.93) | 0  (0 to 0) |
| Zimbabwe | 5671 (3347 to 8991) | 19665.1 (11605.72 to 31177.95) | 6579 (3883 to 10431) | 19664.61 (11605.36 to 31176.52) | 16.01  (16 to 16.02) | 0  (0 to 0) |

CI, confidence interval; EAPC, estimated annual percentage change; LMICs, low- and middle-income countries and territories; UI, uncertain interval.

## Supplementary Table S17. National burden and trends of incident cases and incidence rates (per 100,000 liveborn neonates) of scabies in 131 LMICs between 1990 and 2019.

| **Location** | **Incident cases in 1990**  **(95% UI)** | **Incident rate in 1990**  **(95% UI)** | **Incident cases in 2019**  **(95% UI)** | **Incident rate in 2019**  **(95% UI)** | **Percent change**  **(%, 95% CI)** | **EAPC**  **(%, 95% CI)** |
| --- | --- | --- | --- | --- | --- | --- |
| Global | 992110 (757759 to 1264629) | 9609.22 (7339.38 to 12248.74) | 862401 (659991 to 1094540) | 8439.72 (6458.88 to 10711.51) | -13.07  (-14.01 to -12.16) | -0.47  (-0.51 to -0.43) |
| Afghanistan | 1271 (968 to 1620) | 3285.23 (2501.22 to 4187.34) | 3671 (2775 to 4748) | 3282.25 (2481.64 to 4245.14) | 188.81  (159.25 to 219.64) | -0.01  (-0.01 to 0) |
| Albania | 311 (237 to 404) | 4790.47 (3651.2 to 6206.8) | 127 (97 to 163) | 4765.3 (3628.92 to 6133.2) | -59.23  (-62.27 to -56.04) | -0.04  (-0.05 to -0.03) |
| Algeria | 1838 (1393 to 2341) | 3164.17 (2397.82 to 4030.45) | 2096 (1583 to 2684) | 3160.16 (2387.34 to 4047.11) | 14.04  (5.54 to 26.97) | 0  (-0.01 to 0) |
| American Samoa | 25 (19 to 32) | 19157.13 (14342.83 to 24516.08) | 15 (12 to 20) | 18976.12 (14439.94 to 24572.89) | -38.78  (-44.12 to -32.2) | -0.18  (-0.22 to -0.13) |
| Angola | 1276 (968 to 1665) | 3367.57 (2553.96 to 4393.59) | 2803 (2116 to 3624) | 3361.72 (2538.52 to 4346.04) | 119.63  (97.05 to 145.04) | 0  (-0.01 to 0.01) |
| Armenia | 102 (77 to 132) | 1774.18 (1348.21 to 2292.76) | 50 (38 to 64) | 1763.58 (1335.52 to 2246.52) | -50.5  (-54.31 to -45.81) | -0.04  (-0.05 to -0.03) |
| Azerbaijan | 255 (190 to 329) | 1779.57 (1331.31 to 2300.38) | 193 (146 to 248) | 1772.42 (1345.08 to 2280.47) | -24.31  (-30.74 to -16.31) | -0.05  (-0.07 to -0.04) |
| Bangladesh | 25345 (19306 to 32797) | 8658.53 (6595.52 to 11204.25) | 17415 (13430 to 22156) | 8571.96 (6610.2 to 10905.18) | -31.29  (-38.03 to -24.6) | -0.02  (-0.03 to -0.01) |
| Belarus | 55 (41 to 71) | 509.95 (385.83 to 660.11) | 40 (30 to 51) | 505.15 (384.8 to 654.84) | -27.42  (-33.94 to -20.61) | -0.05  (-0.05 to -0.04) |
| Belize | 53 (41 to 67) | 11931.8 (9097.1 to 15140.09) | 69 (52 to 89) | 11902.66 (8983.6 to 15330.63) | 30.2  (19.55 to 43.18) | 0.01  (-0.01 to 0.02) |
| Benin | 400 (303 to 515) | 2245.63 (1701.51 to 2895.09) | 837 (634 to 1070) | 2214.37 (1677.66 to 2828.29) | 109.59  (91.02 to 130.3) | -0.03  (-0.04 to -0.03) |
| Bhutan | 142 (108 to 182) | 8622.74 (6592.43 to 11054.12) | 85 (64 to 110) | 8360.94 (6346.92 to 10860.39) | -40.2  (-45.61 to -34.83) | -0.11  (-0.13 to -0.09) |
| Bolivia (Plurinational State of) | 2128 (1611 to 2680) | 11652.63 (8820.31 to 14675.79) | 2801 (2127 to 3550) | 11480.34 (8716.91 to 14553.5) | 31.61  (19.46 to 43.49) | -0.03  (-0.04 to -0.02) |
| Bosnia and Herzegovina | 246 (188 to 318) | 4755.08 (3627.82 to 6142.74) | 97 (73 to 121) | 4725.36 (3575.7 to 5937.83) | -60.69  (-64.13 to -56.8) | -0.04  (-0.05 to -0.02) |
| Botswana | 98 (74 to 123) | 3176.39 (2417.81 to 4017.23) | 112 (84 to 144) | 3081.69 (2304.17 to 3955.84) | 14.89  (5.43 to 26.84) | -0.09  (-0.1 to -0.08) |
| Brazil | 54154 (41166 to 69300) | 20865.26 (15860.8 to 26700.85) | 48561 (36970 to 62212) | 20665.98 (15733.46 to 26475.5) | -10.33  (-12.6 to -7.96) | -0.26  (-0.74 to 0.22) |
| Bulgaria | 356 (270 to 458) | 4743.94 (3595.52 to 6107.32) | 217 (164 to 277) | 4734.99 (3588.6 to 6045.49) | -39.11  (-44.62 to -33.46) | -0.01  (-0.02 to 0) |
| Burkina Faso | 773 (588 to 984) | 2256.48 (1714.54 to 2870.5) | 1583 (1202 to 2015) | 2244.6 (1704.49 to 2856.67) | 104.69  (83.1 to 125.7) | -0.01  (-0.02 to 0) |
| Burundi | 832 (636 to 1065) | 4205.72 (3215.81 to 5384.78) | 1465 (1110 to 1879) | 4177.1 (3164.17 to 5357.65) | 76.09  (59.74 to 95.88) | -0.02  (-0.02 to -0.01) |
| Cabo Verde | 20 (15 to 25) | 2243.44 (1708.82 to 2839.11) | 18 (13 to 23) | 2188.35 (1655.92 to 2842.7) | -10.71  (-18.98 to -1.54) | -0.08  (-0.09 to -0.07) |
| Cambodia | 7300 (5602 to 9512) | 22268.9 (17088.2 to 29016.57) | 6056 (4624 to 7604) | 21852.57 (16686.04 to 27441.6) | -17.05  (-25.14 to -9.49) | -0.06  (-0.07 to -0.05) |
| Cameroon | 781 (592 to 997) | 2241.38 (1699.97 to 2862.55) | 1509 (1135 to 1943) | 2220.29 (1669.92 to 2858.97) | 93.25  (78.4 to 111.27) | -0.01  (-0.02 to 0) |
| Central African Republic | 317 (241 to 405) | 3368.27 (2559.37 to 4302.02) | 498 (378 to 631) | 3377.34 (2563.62 to 4281.38) | 57.1  (41.87 to 74.73) | 0.02  (0.01 to 0.02) |
| Chad | 533 (405 to 681) | 2250.23 (1707.07 to 2875.09) | 1323 (1009 to 1673) | 2236.48 (1705.12 to 2827.07) | 148.06  (129.57 to 170.53) | -0.01  (-0.01 to 0) |
| China | 295436 (225320 to 376035) | 16222.97 (12372.73 to 20648.81) | 180994 (137300 to 228877) | 15914.3 (12072.39 to 20124.52) | -38.74  (-39.98 to -37.47) | -0.07  (-0.08 to -0.06) |
| Colombia | 6990 (5309 to 8932) | 9908.89 (7526.49 to 12662.27) | 5913 (4489 to 7612) | 9819.53 (7454.55 to 12640.2) | -15.4  (-23.16 to -6.07) | -0.01  (-0.03 to 0) |
| Comoros | 62 (47 to 79) | 4183.37 (3197.14 to 5361.44) | 51 (40 to 65) | 4111.82 (3183.84 to 5242.66) | -16.95  (-26.16 to -8.6) | -0.05  (-0.06 to -0.05) |
| Congo | 251 (192 to 322) | 3346.52 (2558.09 to 4288.32) | 360 (273 to 458) | 3318.35 (2514.81 to 4219.57) | 43.44  (29.17 to 58.22) | -0.01  (-0.02 to 0) |
| Costa Rica | 606 (462 to 763) | 9837.19 (7495.23 to 12389.03) | 493 (374 to 632) | 9746.66 (7397.46 to 12500.26) | -18.61  (-24.89 to -9.79) | -0.01  (-0.03 to 0) |
| Côte d'Ivoire | 955 (729 to 1209) | 2242.43 (1712.05 to 2836.63) | 1468 (1117 to 1890) | 2216.34 (1686.4 to 2852.45) | 53.67  (39.54 to 70.01) | -0.02  (-0.03 to -0.01) |
| Cuba | 1628 (1233 to 2062) | 11800.5 (8936.82 to 14946.83) | 930 (706 to 1192) | 11643.63 (8841.7 to 14926.9) | -42.91  (-48.59 to -38.05) | -0.02  (-0.04 to -0.01) |
| Democratic People's Republic of Korea | 8328 (6333 to 10762) | 15353.78 (11674.93 to 19839.47) | 3987 (3025 to 5042) | 15260.57 (11577.49 to 19301.31) | -52.13  (-57.17 to -46.89) | -0.01  (-0.02 to -0.01) |
| Democratic Republic of the Congo | 4693 (3558 to 5981) | 3350.57 (2539.8 to 4269.64) | 7370 (5671 to 9405) | 3349.45 (2577.29 to 4274.23) | 57.04  (40.28 to 74.68) | 0  (-0.01 to 0.01) |
| Djibouti | 69 (53 to 89) | 4183.95 (3188.87 to 5348.5) | 108 (82 to 138) | 4122.7 (3137.36 to 5284.29) | 54.96  (41.39 to 69.5) | -0.05  (-0.05 to -0.04) |
| Dominica | 15 (12 to 20) | 11847.1 (8998.22 to 15177.38) | 7 (6 to 9) | 11669.86 (8854.45 to 14863.93) | -52.06  (-56.31 to -47.68) | -0.03  (-0.05 to -0.02) |
| Dominican Republic | 2152 (1648 to 2798) | 11946.27 (9148.39 to 15533.73) | 2034 (1542 to 2586) | 11781.56 (8934.49 to 14978.09) | -5.49  (-16 to 4.65) | -0.02  (-0.03 to -0.01) |
| Ecuador | 2641 (2028 to 3383) | 11659.77 (8953.05 to 14934.28) | 3056 (2310 to 3931) | 11508.37 (8698.16 to 14803.16) | 15.71  (5.62 to 26.84) | -0.02  (-0.04 to 0) |
| Egypt | 5886 (4506 to 7565) | 4142.91 (3171.77 to 5325.06) | 6475 (5006 to 8411) | 4021.6 (3108.93 to 5223.95) | 10.01  (-1.08 to 23.27) | -0.11  (-0.13 to -0.09) |
| El Salvador | 1244 (946 to 1608) | 10047.4 (7634.06 to 12978.67) | 839 (636 to 1067) | 9809.62 (7438 to 12467.97) | -32.56  (-39.2 to -26.07) | -0.06  (-0.07 to -0.04) |
| Equatorial Guinea | 57 (44 to 73) | 3354.18 (2559.75 to 4288.7) | 97 (74 to 124) | 3317.48 (2525.55 to 4240.44) | 68.93  (55.86 to 85.67) | -0.06  (-0.08 to -0.05) |
| Eritrea | 390 (298 to 497) | 4187.61 (3199.34 to 5331.41) | 621 (477 to 791) | 4137.79 (3179.83 to 5266.21) | 59.09  (45.95 to 72.78) | -0.04  (-0.04 to -0.03) |
| Eswatini | 76 (58 to 97) | 3172.25 (2423.62 to 4035.24) | 69 (53 to 91) | 3127.15 (2378.82 to 4102.98) | -8.86  (-16.51 to 1.6) | -0.02  (-0.03 to -0.01) |
| Ethiopia | 14681 (11206 to 18675) | 7970.88 (6084.22 to 10139.5) | 21654 (16460 to 27343) | 7873.44 (5984.88 to 9942.11) | 47.5  (40.78 to 55.05) | 0.06  (0.03 to 0.09) |
| Fiji | 312 (235 to 397) | 21689.24 (16308.4 to 27554.22) | 285 (216 to 361) | 21448.17 (16232.45 to 27131.99) | -8.72  (-16.86 to 2.8) | -0.06  (-0.09 to -0.03) |
| Gabon | 98 (74 to 124) | 3325.06 (2518.59 to 4214.22) | 106 (80 to 136) | 3276.46 (2485.22 to 4217.38) | 7.49  (-1.63 to 19.69) | -0.07  (-0.09 to -0.04) |
| Gambia | 81 (61 to 104) | 2250.31 (1698.66 to 2877.61) | 119 (90 to 152) | 2212.47 (1683.63 to 2841.75) | 46.41  (32.62 to 62.09) | -0.03  (-0.05 to -0.02) |
| Georgia | 112 (85 to 145) | 1770.99 (1340.26 to 2287.4) | 61 (47 to 79) | 1768.75 (1347.97 to 2272.89) | -45.12  (-49.97 to -39.42) | -0.03  (-0.04 to -0.02) |
| Ghana | 498 (375 to 639) | 1101.12 (830.67 to 1414.41) | 705 (540 to 917) | 1097.61 (841.09 to 1427.01) | 41.69  (28.51 to 56.15) | -0.04  (-0.08 to 0) |
| Grenada | 18 (14 to 23) | 11841.06 (9032.41 to 15152.04) | 12 (9 to 16) | 11633.34 (8924.74 to 14747.46) | -31.09  (-37.44 to -24.72) | -0.03  (-0.05 to -0.02) |
| Guatemala | 2568 (1952 to 3259) | 10109.27 (7685.92 to 12830.31) | 3103 (2381 to 3975) | 9917.51 (7607.9 to 12704.15) | 20.85  (11.86 to 33.59) | -0.05  (-0.06 to -0.04) |
| Guinea | 529 (403 to 673) | 2259.81 (1720.23 to 2873.97) | 802 (606 to 1040) | 2229.29 (1684.21 to 2891.93) | 51.44  (38.43 to 71.1) | -0.02  (-0.03 to -0.01) |
| Guinea-Bissau | 78 (59 to 98) | 2247.99 (1704.89 to 2838.54) | 104 (79 to 133) | 2226.29 (1694.97 to 2832.37) | 33.89  (23.01 to 46.64) | 0  (-0.01 to 0.01) |
| Guyana | 197 (150 to 252) | 11831.44 (8991.97 to 15069.69) | 128 (98 to 163) | 11798.7 (9016.98 to 15039.48) | -35.26  (-41.1 to -28.61) | 0  (-0.01 to 0.01) |
| Haiti | 2212 (1678 to 2848) | 11976.79 (9082.8 to 15417.37) | 2950 (2244 to 3772) | 11868.52 (9028.48 to 15174.7) | 33.37  (21.06 to 48.6) | -0.01  (-0.03 to 0) |
| Honduras | 1352 (1028 to 1741) | 9971.11 (7581.23 to 12838.26) | 1749 (1325 to 2230) | 9807.74 (7427.33 to 12502.33) | 29.33  (17.35 to 41.16) | -0.04  (-0.05 to -0.03) |
| India | 177960 (135948 to 227440) | 9064.2 (6924.36 to 11584.4) | 158995 (121043 to 203984) | 8969.35 (6828.4 to 11507.3) | -10.66  (-12.76 to -8.75) | -0.08  (-0.23 to 0.07) |
| Indonesia | 81644 (62307 to 103448) | 22756.41 (17366.72 to 28833.91) | 65826 (50408 to 83572) | 22600.98 (17307.3 to 28693.64) | -19.37  (-21.78 to -17.18) | -0.01  (-0.02 to 0) |
| Iran (Islamic Republic of) | 4404 (3373 to 5568) | 3275.51 (2508.86 to 4141.18) | 3335 (2552 to 4203) | 3252.14 (2488.82 to 4098.23) | -24.28  (-26.02 to -22.47) | -0.01  (-0.02 to 0) |
| Iraq | 1322 (1010 to 1674) | 2502.31 (1911.74 to 3167.15) | 1810 (1368 to 2313) | 2487.31 (1880.75 to 3179.17) | 36.86  (26.52 to 49.18) | -0.03  (-0.05 to -0.01) |
| Jamaica | 500 (379 to 634) | 11773.42 (8934.9 to 14919.73) | 319 (243 to 404) | 11648.26 (8878.11 to 14751.65) | -36.22  (-41.68 to -29.79) | 0  (-0.02 to 0.01) |
| Jordan | 327 (247 to 415) | 3164.07 (2393.17 to 4022.62) | 590 (449 to 757) | 3155.19 (2404.78 to 4052.99) | 80.54  (63.85 to 98.31) | -0.01  (-0.02 to -0.01) |
| Kazakhstan | 479 (363 to 609) | 1776.75 (1349.05 to 2261.27) | 476 (364 to 611) | 1773.1 (1354.77 to 2274.69) | -0.43  (-8.81 to 8.86) | -0.04  (-0.04 to -0.03) |
| Kenya | 4354 (3332 to 5496) | 5785.21 (4427.38 to 7302.66) | 5685 (4343 to 7271) | 5604.65 (4282.11 to 7168.6) | 30.58  (26.97 to 33.3) | -0.16  (-0.18 to -0.14) |
| Kiribati | 38 (29 to 48) | 19452.64 (14763.74 to 24730.5) | 44 (33 to 57) | 19298.63 (14611.11 to 24845.32) | 16.92  (6.51 to 27.67) | -0.05  (-0.05 to -0.04) |
| Kyrgyzstan | 169 (128 to 220) | 1781.41 (1347.86 to 2310) | 197 (151 to 252) | 1779.14 (1366.13 to 2278.96) | 15.97  (5.89 to 27.89) | -0.04  (-0.05 to -0.02) |
| Lao People's Democratic Republic | 2772 (2091 to 3549) | 22403.59 (16896.91 to 28685.57) | 2847 (2163 to 3692) | 22006.28 (16722.44 to 28537.76) | 2.7  (-6.79 to 12.94) | -0.05  (-0.05 to -0.04) |
| Lebanon | 265 (202 to 337) | 3187.13 (2433.4 to 4054.88) | 256 (196 to 322) | 3185.23 (2437.92 to 4015.63) | -3.4  (-12.39 to 6.96) | 0.01  (0 to 0.02) |
| Lesotho | 144 (110 to 184) | 3180.95 (2430.64 to 4060.53) | 109 (84 to 141) | 3140.45 (2400.57 to 4038.05) | -24.09  (-30.94 to -16.72) | -0.01  (-0.02 to 0) |
| Liberia | 135 (104 to 171) | 2249.82 (1729.13 to 2854.32) | 226 (171 to 290) | 2236.1 (1689.24 to 2869.04) | 67.78  (49.98 to 85.53) | -0.01  (-0.02 to -0.01) |
| Libya | 357 (274 to 454) | 3156.96 (2420.36 to 4008.22) | 196 (150 to 251) | 3161.27 (2418.02 to 4037.67) | -45.04  (-49.84 to -39.77) | 0.02  (0.01 to 0.03) |
| Madagascar | 1668 (1276 to 2108) | 4179.13 (3197.55 to 5283.08) | 2683 (2039 to 3451) | 4159.87 (3161.91 to 5351.47) | 60.86  (48.64 to 77.79) | -0.02  (-0.03 to -0.02) |
| Malawi | 1455 (1114 to 1856) | 4184.4 (3203.15 to 5336.91) | 1713 (1306 to 2187) | 4149.69 (3162.92 to 5297.22) | 17.75  (7.79 to 30.1) | -0.02  (-0.03 to -0.01) |
| Malaysia | 8233 (6259 to 10537) | 21788.15 (16564.15 to 27886.01) | 8916 (6833 to 11552) | 21550.5 (16515.56 to 27921.73) | 8.3  (-2.26 to 19.07) | -0.02  (-0.03 to 0) |
| Maldives | 149 (114 to 192) | 22505.89 (17252.52 to 28955.06) | 141 (107 to 184) | 22077.34 (16699.04 to 28745.22) | -5  (-13.46 to 6.38) | -0.05  (-0.06 to -0.04) |
| Mali | 778 (591 to 991) | 2368.33 (1800.51 to 3016.83) | 1697 (1289 to 2177) | 2352.94 (1787.35 to 3019.22) | 118.07  (99.83 to 140.41) | -0.01  (-0.02 to 0) |
| Marshall Islands | 22 (17 to 29) | 19426.71 (14672.98 to 24996.26) | 18 (14 to 23) | 19151.18 (14447.61 to 24837.63) | -19.73  (-24.92 to -13.91) | -0.07  (-0.07 to -0.06) |
| Mauritania | 150 (115 to 190) | 2244.58 (1722.52 to 2845.96) | 183 (138 to 233) | 2212.39 (1673.43 to 2820.3) | 21.99  (12.13 to 33.72) | -0.04  (-0.04 to -0.03) |
| Mexico | 19092 (14558 to 24185) | 10169.61 (7754.71 to 12882.57) | 16435 (12547 to 20871) | 10314.43 (7874.39 to 13098.41) | -13.92  (-16 to -11.25) | 0.21  (0.1 to 0.32) |
| Micronesia (Federated States of) | 51 (39 to 66) | 19399.23 (14670.74 to 24974.25) | 28 (21 to 36) | 19085.17 (14467.85 to 24177) | -44.62  (-49.4 to -38.8) | -0.06  (-0.06 to -0.05) |
| Mongolia | 99 (75 to 129) | 1791.48 (1366.15 to 2336.29) | 109 (82 to 139) | 1790.33 (1349.94 to 2273.83) | 10.54  (1.34 to 22.18) | -0.01  (-0.02 to -0.01) |
| Montenegro | 36 (27 to 46) | 4740.11 (3601.68 to 6097.04) | 24 (18 to 31) | 4756.81 (3619.68 to 6105.67) | -32.59  (-37.92 to -25.38) | -0.01  (-0.01 to 0) |
| Morocco | 1925 (1455 to 2450) | 3238 (2447.29 to 4122.07) | 1456 (1121 to 1842) | 3188.55 (2454.73 to 4034.28) | -24.38  (-30.57 to -17.64) | -0.05  (-0.06 to -0.04) |
| Mozambique | 1829 (1399 to 2358) | 4189.96 (3204.13 to 5401.3) | 3520 (2674 to 4502) | 4164.54 (3163.67 to 5326.83) | 92.4  (71.06 to 113) | -0.04  (-0.05 to -0.03) |
| Myanmar | 22283 (16832 to 28975) | 22454.26 (16961.78 to 29198.57) | 17417 (13221 to 22449) | 22096.5 (16772.83 to 28480.67) | -21.84  (-29 to -13.47) | -0.04  (-0.05 to -0.04) |
| Namibia | 115 (88 to 147) | 3163.25 (2422.1 to 4034.95) | 147 (112 to 192) | 3102.32 (2362.29 to 4053.43) | 27.18  (15.94 to 38.23) | -0.05  (-0.06 to -0.04) |
| Nepal | 5165 (3917 to 6669) | 8516.22 (6459.32 to 10996.97) | 3860 (2947 to 4940) | 8303.68 (6339.87 to 10627.9) | -25.27  (-31.78 to -19.1) | -0.06  (-0.07 to -0.04) |
| Nicaragua | 1099 (834 to 1405) | 10117.68 (7677.74 to 12941.15) | 979 (748 to 1249) | 9827.16 (7510.87 to 12534.17) | -10.88  (-18.95 to -2.46) | -0.08  (-0.09 to -0.07) |
| Niger | 749 (572 to 956) | 2253.79 (1720.64 to 2876.65) | 1933 (1485 to 2469) | 2237.86 (1719.28 to 2858.65) | 158.03  (132.3 to 182.58) | -0.02  (-0.03 to -0.01) |
| Nigeria | 15135 (11592 to 19155) | 4913.95 (3763.5 to 6219.09) | 27298 (20854 to 34498) | 4832.99 (3692.04 to 6107.71) | 80.37  (76.32 to 84.86) | -0.06  (-0.1 to -0.03) |
| North Macedonia | 125 (96 to 160) | 4708.53 (3604.63 to 6007.75) | 80 (61 to 101) | 4718.67 (3580.59 to 5969) | -36.15  (-41.56 to -30.6) | -0.02  (-0.03 to -0.01) |
| Pakistan | 32723 (24931 to 41926) | 9149.23 (6970.64 to 11722.22) | 45269 (34633 to 57776) | 9090.92 (6955.03 to 11602.64) | 38.34  (31.74 to 44.87) | -0.01  (-0.02 to 0) |
| Papua New Guinea | 2194 (1655 to 2822) | 19565.39 (14761.37 to 25167.83) | 4792 (3549 to 6186) | 19397.61 (14366.71 to 25039.52) | 118.4  (98.72 to 140.67) | -0.03  (-0.04 to -0.02) |
| Paraguay | 1935 (1468 to 2501) | 19990.75 (15166.47 to 25829.23) | 1878 (1433 to 2437) | 19559.23 (14919.93 to 25374.16) | -2.95  (-11.73 to 7.98) | -0.08  (-0.09 to -0.07) |
| Peru | 5832 (4391 to 7575) | 11675.24 (8791.05 to 15164.49) | 5591 (4269 to 7079) | 11512.41 (8789.26 to 14575.77) | -4.13  (-12.93 to 5.59) | -0.05  (-0.05 to -0.04) |
| Philippines | 36035 (27587 to 46120) | 23057.93 (17651.95 to 29511.26) | 45727 (34943 to 58208) | 22768.47 (17398.79 to 28983.44) | 26.89  (24.74 to 29.24) | -0.02  (-0.03 to -0.01) |
| Republic of Moldova | 30 (23 to 39) | 513.03 (386.6 to 660.61) | 12 (9 to 16) | 507.51 (385.61 to 651.73) | -58.64  (-62.26 to -54.68) | -0.05  (-0.06 to -0.04) |
| Russian Federation | 2126 (1612 to 2712) | 1444.49 (1094.98 to 1842.52) | 1829 (1392 to 2350) | 1434.69 (1091.67 to 1843.3) | -13.97  (-15.69 to -12.37) | -0.02  (-0.03 to -0.02) |
| Rwanda | 371 (282 to 486) | 1560.82 (1184.19 to 2041.78) | 410 (311 to 535) | 1542.16 (1169.9 to 2008.6) | 10.55  (1.65 to 20.93) | -0.08  (-0.13 to -0.04) |
| Saint Lucia | 34 (26 to 43) | 11804.12 (9028.21 to 15088.28) | 16 (12 to 20) | 11651.49 (8851.52 to 14963.73) | -53.45  (-57.46 to -48.75) | -0.02  (-0.03 to -0.01) |
| Saint Vincent and the Grenadines | 24 (18 to 31) | 11883.8 (9052.75 to 15395.64) | 14 (10 to 18) | 11733.79 (8913.6 to 15303.52) | -43.43  (-48.59 to -37.69) | -0.02  (-0.04 to 0) |
| Samoa | 50 (37 to 64) | 19063.91 (14335.86 to 24755.31) | 55 (42 to 71) | 18967.02 (14370.67 to 24403.74) | 11.4  (-0.04 to 22.27) | -0.03  (-0.04 to -0.02) |
| Senegal | 574 (436 to 733) | 2236.06 (1697.62 to 2857.14) | 767 (583 to 975) | 2197.22 (1670.45 to 2792.98) | 33.75  (23.32 to 46.27) | -0.04  (-0.05 to -0.02) |
| Serbia | 477 (362 to 613) | 4719.08 (3588.53 to 6064.76) | 298 (228 to 381) | 4734.79 (3626.84 to 6042.87) | -37.43  (-42.81 to -30.53) | -0.02  (-0.03 to -0.01) |
| Sierra Leone | 263 (200 to 336) | 2249.02 (1710.91 to 2872.58) | 470 (355 to 607) | 2240.75 (1693.39 to 2896.54) | 78.54  (60.96 to 98.17) | -0.01  (-0.02 to 0) |
| Solomon Islands | 209 (158 to 271) | 19430.03 (14702.23 to 25154.81) | 303 (227 to 390) | 19204.22 (14399.84 to 24697.81) | 44.82  (32.06 to 63.68) | -0.04  (-0.05 to -0.03) |
| Somalia | 963 (740 to 1238) | 4183.39 (3212.78 to 5375.52) | 2721 (2069 to 3466) | 4168.56 (3169.92 to 5310.66) | 182.42  (162.39 to 205.27) | -0.03  (-0.04 to -0.02) |
| South Africa | 2391 (1823 to 3062) | 3260.26 (2485.69 to 4175.26) | 2508 (1923 to 3188) | 3218.98 (2467.97 to 4092.5) | 4.88  (0.9 to 8.94) | -0.02  (-0.02 to -0.01) |
| South Sudan | 792 (600 to 1027) | 4225.22 (3200.16 to 5478.07) | 1133 (864 to 1440) | 4180.83 (3187.18 to 5313.99) | 43.07  (29.41 to 58.42) | -0.02  (-0.03 to -0.01) |
| Sri Lanka | 5316 (4010 to 6915) | 19621.83 (14803.13 to 25522.75) | 4361 (3332 to 5570) | 19280.72 (14729.34 to 24627.98) | -17.96  (-25.64 to -9.3) | 0.07  (0.02 to 0.12) |
| Sudan | 2346 (1757 to 2968) | 3229.28 (2418.23 to 4084.47) | 2875 (2182 to 3750) | 3244.35 (2463.03 to 4232) | 22.52  (12.82 to 34.53) | 0.02  (0.01 to 0.03) |
| Suriname | 81 (62 to 103) | 11791.18 (9016.2 to 15035.26) | 79 (60 to 101) | 11669.76 (8854.36 to 14863.65) | -1.8  (-10.61 to 9.02) | 0  (-0.02 to 0.02) |
| Syrian Arab Republic | 1207 (926 to 1525) | 3177.72 (2437.23 to 4013.66) | 561 (424 to 719) | 3166.62 (2389.65 to 4055.58) | -53.51  (-57.35 to -49.62) | 0  (0 to 0) |
| Tajikistan | 276 (209 to 351) | 1786.47 (1352.49 to 2273.82) | 344 (260 to 445) | 1785.96 (1348.13 to 2313.3) | 24.57  (11.88 to 37.14) | -0.02  (-0.03 to -0.01) |
| Thailand | 17992 (13726 to 22849) | 22378.39 (17071.93 to 28418.96) | 9829 (7502 to 12608) | 22041.81 (16824.61 to 28274.56) | -45.37  (-50.78 to -40.1) | -0.04  (-0.05 to -0.03) |
| Timor-Leste | 682 (528 to 872) | 25398.78 (19676.68 to 32508.43) | 725 (546 to 919) | 24968.26 (18818.72 to 31656.93) | 6.31  (-3.05 to 17.65) | 0.32  (-0.16 to 0.8) |
| Togo | 274 (209 to 349) | 2242.69 (1711.74 to 2859.64) | 398 (302 to 517) | 2236.79 (1698.87 to 2907.64) | 45.13  (33.41 to 60.11) | 0  (-0.01 to 0.01) |
| Tonga | 38 (29 to 49) | 19322.93 (14798.48 to 24831.56) | 34 (25 to 44) | 19149.11 (14277.24 to 24636.01) | -10.83  (-18.76 to -3.56) | -0.06  (-0.07 to -0.05) |
| Tunisia | 570 (434 to 713) | 3187.75 (2427.28 to 3988.01) | 403 (307 to 510) | 3171.53 (2415.09 to 4012.74) | -29.29  (-34.4 to -21.72) | 0  (-0.01 to 0.01) |
| Turkey | 3965 (3028 to 5003) | 3200.53 (2444.1 to 4038.1) | 2368 (1792 to 2990) | 3181.82 (2407.83 to 4017.01) | -40.27  (-46.1 to -33.92) | -0.02  (-0.03 to -0.01) |
| Turkmenistan | 176 (135 to 228) | 1785.13 (1361.81 to 2305.87) | 151 (115 to 191) | 1773.31 (1350.36 to 2252.66) | -14.71  (-22.6 to -6.92) | -0.03  (-0.04 to -0.02) |
| Tuvalu | 4 (3 to 5) | 19340.89 (14692.88 to 24944.77) | 3 (2 to 4) | 19028.71 (14429.3 to 24466.09) | -25.2  (-31.86 to -17.43) | -0.06  (-0.06 to -0.06) |
| Uganda | 2897 (2229 to 3731) | 4190.87 (3224.29 to 5397.56) | 4899 (3748 to 6307) | 4129.98 (3160.08 to 5317.4) | 69.09  (53.94 to 85.79) | -0.05  (-0.06 to -0.04) |
| Ukraine | 265 (199 to 339) | 528.57 (397.23 to 675.64) | 156 (118 to 199) | 525.16 (396.39 to 668.87) | -41.17  (-45.94 to -35.56) | -0.01  (-0.02 to -0.01) |
| United Republic of Tanzania | 6281 (4753 to 8031) | 6906.3 (5225.71 to 8831.09) | 10273 (7837 to 12997) | 6617.99 (5048.93 to 8372.57) | 63.56  (47.83 to 82.03) | -0.17  (-0.2 to -0.14) |
| Uzbekistan | 956 (735 to 1209) | 1772.81 (1362.75 to 2243.53) | 961 (730 to 1234) | 1772.11 (1344.89 to 2275.06) | 0.59  (-8.94 to 12.65) | -0.03  (-0.04 to -0.02) |
| Vanuatu | 75 (57 to 96) | 17299.3 (13145.11 to 22346.12) | 100 (75 to 126) | 17193.05 (12868.41 to 21556.47) | 34.61  (22.09 to 50.59) | -0.03  (-0.04 to -0.03) |
| Venezuela (Bolivarian Republic of) | 4303 (3269 to 5508) | 9949.97 (7560 to 12736.42) | 3519 (2674 to 4473) | 9781.36 (7431.99 to 12432.45) | -18.22  (-25.04 to -9.77) | -0.05  (-0.06 to -0.04) |
| Viet Nam | 30591 (23148 to 39561) | 22165.19 (16772.36 to 28665.01) | 22222 (16870 to 29274) | 21888.5 (16616.22 to 28833.95) | -27.36  (-33.99 to -18.58) | -0.04  (-0.05 to -0.03) |
| Yemen | 1646 (1247 to 2128) | 3256.65 (2468.51 to 4212.11) | 2276 (1744 to 2866) | 3231.88 (2475.7 to 4068.8) | 38.33  (26.57 to 51.92) | -0.04  (-0.05 to -0.03) |
| Zambia | 1194 (914 to 1549) | 4154.2 (3179.65 to 5389.46) | 1907 (1469 to 2475) | 4123.37 (3177.92 to 5351.74) | 59.72  (47.21 to 76.77) | -0.02  (-0.03 to -0.02) |
| Zimbabwe | 911 (695 to 1167) | 3157.26 (2408.29 to 4045.91) | 1049 (795 to 1343) | 3135.06 (2376.04 to 4015) | 15.2  (3.98 to 28.36) | 0.02  (0 to 0.03) |

CI, confidence interval; EAPC, estimated annual percentage change; LMICs, low- and middle-income countries and territories; UI, uncertain interval.

## Supplementary Table S18. National burden and trends of incident cases and incidence rates (per 100,000 liveborn neonates) of tetanus in 131 LMICs between 1990 and 2019.

| **Location** | **Incident cases in 1990**  **(95% UI)** | **Incident rate in 1990**  **(95% UI)** | **Incident cases in 2019**  **(95% UI)** | **Incident rate in 2019**  **(95% UI)** | **Percent change**  **(%, 95% CI)** | **EAPC**  **(%, 95% CI)** |
| --- | --- | --- | --- | --- | --- | --- |
| Global | 370885 (267832 to 497794) | 3592.26 (2594.12 to 4821.46) | 27171 (17694 to 41344) | 265.9 (173.16 to 404.61) | -92.67  (-94.95 to -87.95) | -8.84  (-9.12 to -8.57) |
| Afghanistan | 2304 (645 to 4473) | 5953.8 (1667.35 to 11561.76) | 598 (201 to 1330) | 534.9 (179.99 to 1189.12) | -74.03  (-92.3 to -5.85) | -8.59  (-9.48 to -7.7) |
| Albania | 0 (0 to 1) | 3.99 (1.83 to 7.77) | 0 (0 to 0) | 0.05 (0.02 to 0.13) | -99.44  (-99.83 to -98.08) | -14.12  (-15.72 to -12.49) |
| Algeria | 19 (4 to 47) | 32.34 (7.4 to 81.2) | 2 (1 to 5) | 2.77 (0.9 to 7.01) | -90.23  (-97.84 to -41.46) | -7.95  (-8.03 to -7.87) |
| American Samoa | 0 (0 to 0) | 0.62 (0.22 to 1.48) | 0 (0 to 0) | 0.04 (0.02 to 0.06) | -96.16  (-98.38 to -89.78) | -10.64  (-11.87 to -9.39) |
| Angola | 592 (148 to 1427) | 1561.23 (389.24 to 3764.64) | 82 (18 to 204) | 98.89 (21.23 to 244.92) | -86.06  (-96.95 to -33.01) | -9  (-9.31 to -8.7) |
| Armenia | 0 (0 to 0) | 0.12 (0.07 to 0.19) | 0 (0 to 0) | 0.01 (0.01 to 0.02) | -95.06  (-97.56 to -89.16) | -9.06  (-9.86 to -8.25) |
| Azerbaijan | 0 (0 to 0) | 1.64 (0.85 to 2.84) | 0 (0 to 0) | 0.27 (0.08 to 0.67) | -87.57  (-96.61 to -64.51) | -5.77  (-6.45 to -5.08) |
| Bangladesh | 34886 (22401 to 50424) | 11917.87 (7652.76 to 17226.11) | 315 (98 to 813) | 155.18 (48.41 to 400.02) | -99.1  (-99.72 to -97.42) | -17.86  (-19.81 to -15.87) |
| Belarus | 0 (0 to 0) | 0 (0 to 0) | 0 (0 to 0) | 0 (0 to 0) | -45.6  (-79.08 to 26.49) | 0.61  (-0.71 to 1.95) |
| Belize | 0 (0 to 0) | 9.97 (5.55 to 16.4) | 0 (0 to 0) | 0.58 (0.26 to 1.17) | -92.41  (-96.69 to -82.92) | -9.47  (-10.26 to -8.66) |
| Benin | 230 (72 to 593) | 1290.22 (405.1 to 3333.88) | 110 (35 to 282) | 292.12 (92.35 to 745.65) | -51.88  (-83.42 to 50.94) | -4.21  (-4.44 to -3.98) |
| Bhutan | 148 (32 to 325) | 8992.98 (1956.26 to 19792.19) | 1 (0 to 4) | 130.46 (23.63 to 422.76) | -99.11  (-99.82 to -96.58) | -14.33  (-14.65 to -14.01) |
| Bolivia (Plurinational State of) | 99 (27 to 244) | 541.17 (147.6 to 1336.18) | 4 (1 to 9) | 14.4 (4.89 to 36.08) | -96.45  (-98.93 to -85.86) | -11.26  (-11.76 to -10.75) |
| Bosnia and Herzegovina | 0 (0 to 0) | 0.05 (0.03 to 0.09) | 0 (0 to 0) | 0.03 (0.02 to 0.04) | -77.4  (-87.26 to -59.82) | -3.4  (-4.25 to -2.54) |
| Botswana | 1 (0 to 3) | 38.89 (14.58 to 84.78) | 0 (0 to 1) | 5.74 (2.03 to 14.4) | -82.54  (-94.39 to -42.03) | -5.89  (-6.24 to -5.54) |
| Brazil | 787 (546 to 1132) | 303.39 (210.42 to 436.09) | 8 (5 to 12) | 3.4 (2.23 to 4.98) | -98.99  (-99.31 to -98.5) | -14.65  (-15.21 to -14.09) |
| Bulgaria | 0 (0 to 0) | 0.31 (0.19 to 0.52) | 0 (0 to 0) | 0.01 (0 to 0.02) | -98.48  (-99.38 to -96.28) | -11.68  (-12.79 to -10.55) |
| Burkina Faso | 789 (352 to 1543) | 2302.84 (1026.8 to 4500.18) | 235 (96 to 510) | 332.89 (135.5 to 722.98) | -70.25  (-90.11 to -11.86) | -7.38  (-7.92 to -6.84) |
| Burundi | 320 (96 to 777) | 1620.13 (482.85 to 3925.91) | 102 (28 to 289) | 291.3 (78.9 to 824.53) | -68.12  (-91.3 to 27.39) | -6.55  (-7.18 to -5.92) |
| Cabo Verde | 1 (0 to 2) | 100.74 (31.81 to 272.29) | 0 (0 to 0) | 2.82 (1.04 to 7.04) | -97.43  (-99.32 to -89.16) | -13.15  (-13.74 to -12.56) |
| Cambodia | 3606 (1087 to 8254) | 10998.53 (3316.52 to 25178.98) | 54 (15 to 168) | 193.11 (52.62 to 607.18) | -98.52  (-99.54 to -95.92) | -14.12  (-14.64 to -13.59) |
| Cameroon | 318 (113 to 694) | 913.02 (325.32 to 1992.58) | 97 (39 to 215) | 143.38 (57.32 to 316.96) | -69.36  (-89.31 to 1.74) | -6.27  (-6.69 to -5.84) |
| Central African Republic | 104 (36 to 228) | 1109.71 (384.62 to 2428.1) | 95 (32 to 222) | 641.4 (220.37 to 1503.55) | -9.45  (-76.37 to 258.61) | -2.7  (-3.22 to -2.18) |
| Chad | 1130 (326 to 2948) | 4769.22 (1376.63 to 12436.79) | 714 (222 to 1660) | 1206.64 (375.23 to 2805.77) | -36.85  (-76.77 to 99.83) | -4.3  (-4.87 to -3.72) |
| China | 41633 (28439 to 61069) | 2286.15 (1561.62 to 3353.4) | 144 (87 to 233) | 12.71 (7.63 to 20.52) | -99.65  (-99.77 to -99.41) | -18.36  (-19.2 to -17.51) |
| Colombia | 161 (100 to 236) | 228.33 (142.41 to 335.07) | 4 (2 to 9) | 6.51 (2.73 to 14.75) | -97.56  (-98.99 to -94.76) | -10.38  (-10.96 to -9.8) |
| Comoros | 14 (3 to 36) | 925.62 (211.01 to 2460.64) | 1 (1 to 3) | 115.57 (46.56 to 245.08) | -89.45  (-96.52 to -39.45) | -7.64  (-7.99 to -7.29) |
| Congo | 19 (5 to 47) | 257.59 (64.74 to 627.38) | 6 (1 to 16) | 54.78 (5.93 to 144.54) | -69.23  (-94.8 to 8.46) | -5.86  (-6.49 to -5.22) |
| Costa Rica | 0 (0 to 0) | 0.39 (0.22 to 0.62) | 0 (0 to 0) | 0 (0 to 0.01) | -99.04  (-99.6 to -97.78) | -15.93  (-18.6 to -13.17) |
| Côte d'Ivoire | 520 (140 to 1335) | 1220.11 (328.64 to 3133.09) | 159 (57 to 357) | 240.57 (85.82 to 539.36) | -69.34  (-89.05 to -0.04) | -5.01  (-5.35 to -4.67) |
| Cuba | 0 (0 to 0) | 0 (0 to 0.01) | 0 (0 to 0) | 0 (0 to 0) | -72.98  (-88.79 to -42.03) | -1.47  (-2.02 to -0.91) |
| Democratic People's Republic of Korea | 203 (45 to 636) | 374.63 (83.34 to 1172.28) | 20 (5 to 65) | 77.39 (20.2 to 250.29) | -90.05  (-98.72 to -34.31) | -5.91  (-6.89 to -4.92) |
| Democratic Republic of the Congo | 1240 (536 to 2510) | 884.97 (382.77 to 1792.07) | 380 (66 to 1330) | 172.84 (30.21 to 604.44) | -69.32  (-94.39 to 41.87) | -5.38  (-6.15 to -4.59) |
| Djibouti | 10 (2 to 27) | 578.56 (139.38 to 1618.83) | 3 (1 to 6) | 108.46 (38.83 to 245.85) | -70.52  (-91 to 27.95) | -6.52  (-7.33 to -5.71) |
| Dominica | 0 (0 to 0) | 19.83 (11.28 to 32.3) | 0 (0 to 0) | 4.67 (1.91 to 9.53) | -88.53  (-95.24 to -73.04) | -5.54  (-5.9 to -5.18) |
| Dominican Republic | 9 (5 to 16) | 51.99 (28.45 to 91.36) | 2 (0 to 4) | 8.84 (2.6 to 23.04) | -83.71  (-95.18 to -52.86) | -5.54  (-5.94 to -5.15) |
| Ecuador | 15 (9 to 24) | 64.8 (39.05 to 104.84) | 1 (0 to 1) | 2.32 (1.07 to 4.67) | -95.81  (-98.15 to -91.2) | -12.61  (-13.22 to -11.99) |
| Egypt | 486 (246 to 899) | 341.99 (173.42 to 632.55) | 34 (4 to 153) | 21.28 (2.21 to 95.33) | -92.95  (-99.3 to -61.57) | -9.21  (-9.45 to -8.97) |
| El Salvador | 13 (7 to 24) | 107.72 (57.68 to 194.08) | 0 (0 to 0) | 1.08 (0.3 to 2.71) | -99.31  (-99.82 to -97.92) | -16  (-17.23 to -14.75) |
| Equatorial Guinea | 27 (9 to 65) | 1604.06 (544.84 to 3791.81) | 1 (0 to 2) | 23.19 (2.21 to 67.29) | -97.53  (-99.69 to -90.09) | -14.77  (-15.74 to -13.78) |
| Eritrea | 356 (142 to 712) | 3812.69 (1519.22 to 7630.39) | 27 (11 to 57) | 182.7 (73.58 to 381.45) | -92.28  (-97.25 to -78.1) | -9.28  (-10.04 to -8.51) |
| Eswatini | 2 (1 to 4) | 74.23 (25.1 to 175.69) | 0 (0 to 1) | 19.08 (3.75 to 46.77) | -76.23  (-94.73 to -17.24) | -3.99  (-4.59 to -3.39) |
| Ethiopia | 4255 (2046 to 7360) | 2310.18 (1111.14 to 3996) | 536 (292 to 932) | 194.82 (105.99 to 338.87) | -87.41  (-93.88 to -72.82) | -8.17  (-8.93 to -7.4) |
| Fiji | 0 (0 to 0) | 0.1 (0.04 to 0.23) | 0 (0 to 0) | 0.08 (0.04 to 0.18) | -25.93  (-75.57 to 143.75) | 1.33  (0.46 to 2.21) |
| Gabon | 5 (1 to 10) | 153.04 (22.9 to 341.46) | 1 (0 to 2) | 22.36 (2.73 to 66.32) | -84.06  (-97.68 to -31.58) | -6.76  (-7.64 to -5.86) |
| Gambia | 26 (15 to 43) | 728.71 (407.92 to 1197.88) | 11 (2 to 27) | 203.06 (43.36 to 508.01) | -58.5  (-91.52 to 22.04) | -4.31  (-4.51 to -4.12) |
| Georgia | 0 (0 to 0) | 0 (0 to 0) | 0 (0 to 0) | 0 (0 to 0) | -52.72  (-79.33 to 5.69) | 1.13  (0.34 to 1.93) |
| Ghana | 719 (293 to 1448) | 1590.65 (648.97 to 3204.41) | 134 (51 to 303) | 208.05 (78.7 to 471.1) | -81.41  (-94.79 to -34.58) | -5.74  (-6.05 to -5.43) |
| Grenada | 0 (0 to 0) | 18.45 (9.51 to 30.68) | 0 (0 to 0) | 0.49 (0.23 to 0.97) | -98.12  (-99.23 to -95.82) | -11.45  (-12.41 to -10.49) |
| Guatemala | 5 (3 to 8) | 21.45 (13.42 to 33.04) | 0 (0 to 0) | 0.58 (0.24 to 1.15) | -96.66  (-98.51 to -93.07) | -12.19  (-13.1 to -11.28) |
| Guinea | 801 (367 to 1562) | 3417.9 (1565.86 to 6667.05) | 206 (71 to 515) | 571.96 (196.8 to 1432.64) | -74.31  (-93.11 to -19.08) | -5.44  (-5.91 to -4.96) |
| Guinea-Bissau | 92 (27 to 219) | 2658.86 (771.42 to 6331.69) | 28 (10 to 62) | 590.52 (210.61 to 1315.96) | -69.97  (-89.74 to 16.58) | -4.82  (-5.28 to -4.37) |
| Guyana | 0 (0 to 0) | 14.03 (8.06 to 22) | 0 (0 to 0) | 0.85 (0.42 to 1.56) | -96.06  (-98.17 to -92.11) | -8.94  (-9.27 to -8.61) |
| Haiti | 822 (337 to 1631) | 4449.1 (1822.3 to 8828.21) | 176 (57 to 474) | 708.65 (227.83 to 1904.99) | -78.56  (-93.81 to -10.45) | -6.26  (-6.34 to -6.17) |
| Honduras | 6 (3 to 14) | 46.92 (19.88 to 100.12) | 2 (1 to 9) | 13.24 (3.44 to 48.02) | -62.89  (-90.88 to 54.04) | -4.29  (-4.54 to -4.05) |
| India | 156732 (109815 to 213599) | 7982.96 (5593.29 to 10879.43) | 8066 (4004 to 15339) | 455 (225.89 to 865.3) | -94.85  (-97.42 to -89.91) | -9.67  (-10.35 to -8.99) |
| Indonesia | 19462 (10863 to 31440) | 5424.72 (3027.82 to 8763.17) | 1027 (649 to 1561) | 352.56 (222.97 to 536.08) | -94.72  (-97.22 to -89.52) | -8.36  (-8.64 to -8.08) |
| Iran (Islamic Republic of) | 101 (39 to 174) | 75.46 (29.21 to 129.48) | 2 (1 to 4) | 2.22 (0.88 to 4.23) | -97.76  (-99.3 to -90.06) | -11.6  (-12.25 to -10.94) |
| Iraq | 183 (29 to 444) | 345.45 (55.38 to 840.82) | 16 (6 to 37) | 22.57 (7.91 to 50.38) | -91  (-97.98 to -32.6) | -9.42  (-10.24 to -8.59) |
| Jamaica | 0 (0 to 0) | 4.29 (2.21 to 7.67) | 0 (0 to 0) | 0.11 (0.04 to 0.23) | -98.4  (-99.43 to -95.96) | -12.45  (-13.27 to -11.62) |
| Jordan | 4 (1 to 11) | 39.82 (10.74 to 102.6) | 0 (0 to 1) | 1.74 (0.6 to 4.43) | -92.07  (-97.98 to -60.86) | -12.49  (-13.47 to -11.51) |
| Kazakhstan | 0 (0 to 0) | 0.53 (0.27 to 0.94) | 0 (0 to 0) | 0 (0 to 0.01) | -99.45  (-99.79 to -98.63) | -19.12  (-20.79 to -17.42) |
| Kenya | 2309 (803 to 5896) | 3068.73 (1066.88 to 7834.35) | 986 (364 to 2184) | 971.81 (359.18 to 2153.39) | -57.32  (-72.87 to -16.49) | -3.14  (-3.44 to -2.84) |
| Kiribati | 1 (1 to 2) | 629.51 (287.24 to 1177.36) | 1 (0 to 3) | 637.51 (190.38 to 1509.32) | 19.35  (-65.03 to 309.13) | 3.45  (1.63 to 5.3) |
| Kyrgyzstan | 0 (0 to 0) | 0 (0 to 0) | 0 (0 to 0) | 0 (0 to 0.01) | 49.71  (-30.24 to 212.67) | 2.97  (2.23 to 3.72) |
| Lao People's Democratic Republic | 2229 (499 to 4771) | 18012.18 (4029 to 38556.92) | 50 (9 to 128) | 389.55 (70.68 to 990.58) | -97.74  (-99.21 to -93.8) | -12.67  (-13.23 to -12.11) |
| Lebanon | 1 (0 to 2) | 11.53 (4.04 to 27.42) | 0 (0 to 0) | 1 (0.15 to 4.9) | -91.62  (-98.63 to -46.14) | -7.82  (-8.19 to -7.46) |
| Lesotho | 9 (3 to 23) | 199.32 (71.52 to 506.03) | 1 (0 to 3) | 37.21 (9.27 to 92.78) | -85.65  (-96.59 to -43.35) | -5.21  (-5.66 to -4.77) |
| Liberia | 109 (46 to 217) | 1810.11 (761.94 to 3610.8) | 13 (3 to 48) | 129.11 (26.91 to 474.08) | -87.96  (-97.47 to -53.24) | -9.05  (-9.46 to -8.64) |
| Libya | 1 (0 to 4) | 12.19 (1.92 to 31.6) | 0 (0 to 0) | 2.52 (0.15 to 7.57) | -88.67  (-99.36 to -5.46) | -5.86  (-6.54 to -5.17) |
| Madagascar | 703 (450 to 1034) | 1760.96 (1126.99 to 2591.79) | 173 (44 to 458) | 267.77 (67.65 to 710.3) | -75.43  (-93.71 to -33.24) | -5.81  (-6.21 to -5.41) |
| Malawi | 406 (203 to 770) | 1167.96 (583.72 to 2215.68) | 161 (77 to 307) | 389.43 (185.82 to 744.32) | -60.41  (-83.76 to -5.76) | -3.04  (-3.56 to -2.52) |
| Malaysia | 15 (6 to 33) | 40.8 (15.82 to 86.95) | 1 (0 to 2) | 2.75 (1.09 to 5.83) | -92.63  (-97.55 to -76.43) | -9.5  (-10.38 to -8.61) |
| Maldives | 1 (0 to 3) | 174.31 (24.22 to 400.87) | 0 (0 to 0) | 4.33 (1.91 to 9.22) | -97.59  (-99.24 to -81.49) | -12.71  (-13.65 to -11.77) |
| Mali | 299 (132 to 609) | 910.91 (403.06 to 1854.1) | 480 (224 to 957) | 666.25 (310.31 to 1327.48) | 60.54  (-38 to 348.54) | -1.26  (-2.65 to 0.16) |
| Marshall Islands | 0 (0 to 0) | 14 (2.21 to 37.27) | 0 (0 to 0) | 2.94 (0.18 to 8.37) | -82.9  (-96.34 to -48.99) | -5.95  (-6.61 to -5.28) |
| Mauritania | 81 (33 to 173) | 1212.14 (488.9 to 2589.56) | 9 (3 to 20) | 108.43 (40.73 to 247.48) | -88.93  (-96.14 to -62.29) | -7.09  (-7.55 to -6.63) |
| Mexico | 262 (184 to 355) | 139.35 (97.9 to 189) | 1 (1 to 2) | 0.93 (0.61 to 1.39) | -99.43  (-99.61 to -99.21) | -15.61  (-16.43 to -14.78) |
| Micronesia (Federated States of) | 0 (0 to 0) | 10.98 (1.4 to 29.34) | 0 (0 to 0) | 1.55 (0.09 to 4.98) | -92.03  (-98.42 to -65.9) | -6.74  (-7.19 to -6.3) |
| Mongolia | 0 (0 to 0) | 0.02 (0.02 to 0.03) | 0 (0 to 0) | 0.02 (0.02 to 0.04) | 12.34  (-22.43 to 61.12) | 0.13  (-0.08 to 0.34) |
| Montenegro | 0 (0 to 0) | 0.04 (0.02 to 0.06) | 0 (0 to 0) | 0.03 (0.02 to 0.04) | -51.3  (-74.59 to -25.11) | -1.41  (-1.55 to -1.27) |
| Morocco | 2113 (869 to 4177) | 3554.93 (1462.5 to 7026.53) | 94 (15 to 400) | 205.19 (32.69 to 875.51) | -95.57  (-99.24 to -77.63) | -9.14  (-9.29 to -8.98) |
| Mozambique | 785 (334 to 1601) | 1798.08 (764.29 to 3667.51) | 108 (43 to 233) | 128.21 (51.09 to 275.97) | -86.2  (-95.79 to -58.03) | -8.83  (-9.06 to -8.6) |
| Myanmar | 5088 (1152 to 12918) | 5127.35 (1160.88 to 13017.42) | 213 (58 to 557) | 270.59 (73.07 to 706.62) | -95.81  (-98.76 to -83.98) | -9.5  (-9.68 to -9.32) |
| Namibia | 4 (1 to 9) | 102.43 (28.64 to 257.35) | 0 (0 to 1) | 8.27 (2.18 to 21.53) | -89.53  (-96.91 to -58.66) | -8.39  (-8.82 to -7.95) |
| Nepal | 17990 (8481 to 30385) | 29663.88 (13984.26 to 50101.92) | 596 (221 to 1369) | 1281.56 (475.3 to 2946.12) | -96.69  (-98.85 to -88.84) | -10.37  (-10.72 to -10.02) |
| Nicaragua | 1 (0 to 1) | 5.48 (2.25 to 11.5) | 0 (0 to 0) | 0.03 (0.02 to 0.04) | -99.56  (-99.81 to -98.82) | -20.76  (-22.46 to -19.02) |
| Niger | 1006 (337 to 2302) | 3026.98 (1012.56 to 6924.89) | 344 (123 to 770) | 398.17 (142.7 to 891.15) | -65.82  (-90.44 to 50.93) | -7.39  (-7.84 to -6.94) |
| Nigeria | 14336 (6738 to 28699) | 4654.69 (2187.72 to 9317.88) | 3851 (1967 to 6576) | 681.73 (348.3 to 1164.28) | -73.14  (-91.1 to -37.03) | -6.78  (-7.34 to -6.21) |
| North Macedonia | 0 (0 to 0) | 0.03 (0.02 to 0.05) | 0 (0 to 0) | 0.03 (0.02 to 0.04) | -42.6  (-61 to -19.19) | -0.25  (-0.3 to -0.19) |
| Pakistan | 38251 (21730 to 64020) | 10694.87 (6075.63 to 17899.6) | 3702 (2129 to 6437) | 743.37 (427.6 to 1292.75) | -90.32  (-95.16 to -78.75) | -9.08  (-9.42 to -8.74) |
| Papua New Guinea | 1 (0 to 2) | 5.67 (0.29 to 18.45) | 1 (0 to 2) | 2.19 (0.1 to 8.19) | -14.84  (-77.06 to 208) | -3.02  (-3.1 to -2.95) |
| Paraguay | 49 (28 to 86) | 509.61 (286.24 to 883.95) | 0 (0 to 1) | 3.98 (1.35 to 9.15) | -99.23  (-99.75 to -97.81) | -17.67  (-18.64 to -16.7) |
| Peru | 30 (16 to 51) | 59.84 (32.05 to 101.83) | 1 (0 to 2) | 1.9 (0.7 to 4.5) | -96.91  (-98.96 to -91.09) | -11.96  (-12.38 to -11.55) |
| Philippines | 3176 (2235 to 4424) | 2032.31 (1430.3 to 2830.56) | 241 (140 to 374) | 119.95 (69.93 to 186.27) | -92.42  (-95.3 to -88.5) | -9.01  (-9.33 to -8.7) |
| Republic of Moldova | 0 (0 to 0) | 0 (0 to 0.01) | 0 (0 to 0) | 0 (0 to 0.01) | -50.55  (-77.43 to 3.12) | -1.03  (-1.92 to -0.13) |
| Russian Federation | 2 (1 to 2) | 1.11 (0.8 to 1.47) | 0 (0 to 0) | 0.03 (0.02 to 0.04) | -98.03  (-98.59 to -97.32) | -14  (-15.22 to -12.76) |
| Rwanda | 413 (114 to 839) | 1734.56 (479.97 to 3526.84) | 39 (16 to 85) | 144.97 (61.49 to 319.58) | -90.65  (-96.97 to -53.34) | -9.61  (-10.36 to -8.85) |
| Saint Lucia | 0 (0 to 0) | 12.45 (7.53 to 19.33) | 0 (0 to 0) | 1.26 (0.54 to 2.43) | -95.24  (-98.02 to -89.74) | -7.55  (-8.09 to -7) |
| Saint Vincent and the Grenadines | 0 (0 to 0) | 1.89 (0.95 to 3.45) | 0 (0 to 0) | 0.1 (0.04 to 0.19) | -96.84  (-98.77 to -92.53) | -10.39  (-11.18 to -9.59) |
| Samoa | 0 (0 to 0) | 6.46 (0.63 to 20.23) | 0 (0 to 0) | 1.26 (0.07 to 4.59) | -78.2  (-95.21 to -20.62) | -5.06  (-5.31 to -4.81) |
| Senegal | 367 (123 to 871) | 1429.12 (479.12 to 3394.7) | 99 (30 to 254) | 283.65 (85.53 to 727.54) | -72.99  (-90.59 to -20.6) | -4.97  (-5.21 to -4.72) |
| Serbia | 0 (0 to 0) | 1.58 (0.3 to 4.69) | 0 (0 to 0) | 0.04 (0.02 to 0.07) | -98.55  (-99.58 to -91.59) | -16.91  (-19.85 to -13.86) |
| Sierra Leone | 193 (54 to 531) | 1653.57 (460.15 to 4540.52) | 81 (23 to 207) | 384.62 (107.39 to 989.22) | -58.32  (-86.01 to 46.91) | -5.59  (-6.41 to -4.76) |
| Solomon Islands | 0 (0 to 1) | 17.74 (5.37 to 46.76) | 0 (0 to 0) | 2.39 (0.58 to 6.78) | -80.23  (-94.04 to -34.04) | -6.33  (-6.77 to -5.88) |
| Somalia | 1253 (270 to 3493) | 5438.61 (1171.44 to 15166.4) | 1682 (317 to 6235) | 2576.56 (485.02 to 9553.18) | 34.27  (-56.31 to 233.63) | -2.34  (-2.62 to -2.06) |
| South Africa | 22 (11 to 46) | 30.45 (14.85 to 62.14) | 4 (2 to 6) | 4.73 (2.82 to 7.89) | -83.51  (-92.98 to -63.68) | -7.15  (-7.56 to -6.73) |
| South Sudan | 541 (39 to 1345) | 2885.55 (207.35 to 7177.02) | 185 (21 to 412) | 683.11 (76.27 to 1521.75) | -65.77  (-88.02 to 2.3) | -4.97  (-5.4 to -4.53) |
| Sri Lanka | 23 (10 to 46) | 83.79 (36.73 to 170.97) | 1 (0 to 2) | 3.83 (1.33 to 9.55) | -96.18  (-98.87 to -86.08) | -10.75  (-11.16 to -10.34) |
| Sudan | 318 (70 to 889) | 437.13 (96.23 to 1223) | 20 (5 to 59) | 22.51 (5.48 to 66.51) | -93.72  (-98.73 to -71.71) | -8.83  (-9.23 to -8.44) |
| Suriname | 0 (0 to 0) | 44.66 (27.27 to 71.54) | 0 (0 to 0) | 5.52 (2.12 to 12.13) | -87.74  (-95.23 to -71.85) | -7.93  (-8.32 to -7.54) |
| Syrian Arab Republic | 141 (40 to 324) | 372.12 (105.79 to 852.35) | 2 (0 to 5) | 9.06 (2.51 to 26) | -98.86  (-99.8 to -91.96) | -12.48  (-15.02 to -9.87) |
| Tajikistan | 0 (0 to 0) | 1.04 (0.56 to 1.89) | 0 (0 to 0) | 0.04 (0.02 to 0.08) | -95.16  (-97.65 to -89.74) | -6.47  (-8.76 to -4.13) |
| Thailand | 158 (58 to 339) | 196.63 (72.13 to 422.15) | 1 (0 to 2) | 1.61 (0.66 to 3.38) | -99.55  (-99.85 to -98.44) | -18.05  (-19.91 to -16.15) |
| Timor-Leste | 291 (80 to 563) | 10846.71 (2988.28 to 20990.39) | 8 (3 to 18) | 279.74 (95.38 to 614.51) | -97.21  (-99 to -91.81) | -13.92  (-14.72 to -13.1) |
| Togo | 92 (31 to 241) | 750.48 (253.11 to 1973.87) | 29 (11 to 67) | 163.09 (63.65 to 377.89) | -68.38  (-88.51 to 11.33) | -5.13  (-5.6 to -4.65) |
| Tonga | 0 (0 to 0) | 3.11 (0.45 to 8.41) | 0 (0 to 0) | 0.79 (0.06 to 2.25) | -77.28  (-94.58 to -29.52) | -4.52  (-4.7 to -4.34) |
| Tunisia | 4 (1 to 9) | 21.36 (6.56 to 51.68) | 0 (0 to 1) | 1.58 (0.47 to 4.15) | -94.73  (-99.08 to -71.35) | -8.51  (-8.8 to -8.22) |
| Turkey | 95 (13 to 220) | 76.74 (10.3 to 177.65) | 1 (0 to 1) | 0.75 (0.33 to 1.42) | -99.42  (-99.81 to -94.41) | -17.1  (-18.03 to -16.17) |
| Turkmenistan | 0 (0 to 0) | 1.75 (1.06 to 2.67) | 0 (0 to 0) | 0.31 (0.13 to 0.68) | -84.71  (-93.74 to -66.42) | -1.82  (-3.21 to -0.4) |
| Tuvalu | 0 (0 to 0) | 12.29 (2.49 to 35.41) | 0 (0 to 0) | 1.42 (0.09 to 4.85) | -91.19  (-98.81 to -69.17) | -6.62  (-6.94 to -6.31) |
| Uganda | 1094 (385 to 2349) | 1582.28 (556.46 to 3397.12) | 174 (64 to 399) | 146.8 (53.6 to 336.15) | -84.08  (-95.82 to -38.04) | -8.21  (-8.58 to -7.84) |
| Ukraine | 0 (0 to 0) | 0 (0 to 0) | 0 (0 to 0) | 0 (0 to 0.01) | -5.16  (-59.22 to 101.73) | 3.58  (2.76 to 4.41) |
| United Republic of Tanzania | 632 (282 to 1235) | 694.7 (309.86 to 1358.28) | 240 (85 to 532) | 154.72 (54.61 to 342.51) | -61.99  (-88.92 to 16.16) | -4.71  (-4.97 to -4.44) |
| Uzbekistan | 0 (0 to 1) | 0.56 (0.31 to 0.93) | 0 (0 to 0) | 0.01 (0 to 0.01) | -99.06  (-99.61 to -97.82) | -12.09  (-14.31 to -9.81) |
| Vanuatu | 0 (0 to 0) | 15.03 (3.17 to 36.74) | 0 (0 to 0) | 4.16 (0.4 to 13.37) | -62.55  (-92.94 to 15.07) | -3.52  (-4.26 to -2.79) |
| Venezuela (Bolivarian Republic of) | 3 (2 to 4) | 6.34 (3.74 to 10.34) | 0 (0 to 1) | 0.58 (0.23 to 1.41) | -92.33  (-97.04 to -79.73) | -7.51  (-7.87 to -7.14) |
| Viet Nam | 1863 (642 to 4142) | 1349.64 (464.88 to 3000.88) | 41 (16 to 92) | 40.35 (16.25 to 90.31) | -97.8  (-99.33 to -90.04) | -10.93  (-11.26 to -10.6) |
| Yemen | 179 (36 to 488) | 355.04 (71.52 to 965.25) | 31 (9 to 86) | 44.41 (12.98 to 121.75) | -82.57  (-96.35 to -1.29) | -7.7  (-8.06 to -7.35) |
| Zambia | 445 (135 to 1020) | 1548.94 (470.29 to 3550.98) | 81 (30 to 202) | 175.7 (64.3 to 436.99) | -81.75  (-95.34 to -17.36) | -7.58  (-8.19 to -6.97) |
| Zimbabwe | 2 (1 to 5) | 7.17 (2.99 to 16.07) | 2 (0 to 7) | 6.56 (1.48 to 20.47) | 6.28  (-83.79 to 421.3) | 0.89  (0 to 1.78) |

CI, confidence interval; EAPC, estimated annual percentage change; LMICs, low- and middle-income countries and territories; UI, uncertain interval.

## Supplementary Table S19. National burden and trends of incident cases and incidence rates (per 100,000 liveborn neonates) of tuberculosis in 131 LMICs between 1990 and 2019.

| **Location** | **Incident cases in 1990**  **(95% UI)** | **Incident rate in 1990**  **(95% UI)** | **Incident cases in 2019**  **(95% UI)** | **Incident rate in 2019**  **(95% UI)** | **Percent change**  **(%, 95% CI)** | **EAPC**  **(%, 95% CI)** |
| --- | --- | --- | --- | --- | --- | --- |
| Global | 13137 (8954 to 18284) | 127.24 (86.73 to 177.1) | 6286 (4359 to 8466) | 61.52 (42.66 to 82.85) | -52.15  (-56.05 to -47.44) | -2.61  (-2.73 to -2.49) |
| Afghanistan | 25 (17 to 35) | 64.91 (43.57 to 90.78) | 22 (15 to 31) | 19.86 (13.7 to 27.98) | -11.58  (-31.47 to 15.12) | -4.45  (-4.92 to -3.99) |
| Albania | 0 (0 to 0) | 2.88 (1.89 to 4.09) | 0 (0 to 0) | 0.77 (0.5 to 1.13) | -89.09  (-91.42 to -85.94) | -4.59  (-4.91 to -4.28) |
| Algeria | 8 (5 to 11) | 13.66 (9.13 to 19.74) | 3 (2 to 4) | 3.98 (2.6 to 5.74) | -66.7  (-72.97 to -57.98) | -4.12  (-4.23 to -4.01) |
| American Samoa | 0 (0 to 0) | 2.7 (1.74 to 4) | 0 (0 to 0) | 2.38 (1.55 to 3.48) | -45.56  (-53.93 to -34.19) | -0.49  (-0.66 to -0.32) |
| Angola | 136 (96 to 188) | 358.15 (252.23 to 497.02) | 180 (126 to 243) | 215.3 (151.43 to 291.2) | 32.26  (9.3 to 61.19) | -1.61  (-2.04 to -1.18) |
| Armenia | 1 (1 to 2) | 20.98 (14.17 to 29.47) | 0 (0 to 0) | 7.42 (5.03 to 10.55) | -82.38  (-85.96 to -77.54) | -3.6  (-3.89 to -3.32) |
| Azerbaijan | 6 (4 to 8) | 40.05 (27.92 to 54.97) | 2 (1 to 2) | 14.45 (10.05 to 20.28) | -72.58  (-78.1 to -65.64) | -3.67  (-3.86 to -3.47) |
| Bangladesh | 476 (326 to 642) | 162.78 (111.34 to 219.36) | 83 (57 to 114) | 40.94 (28.12 to 56.02) | -82.54  (-85.41 to -79.3) | -4.67  (-4.8 to -4.54) |
| Belarus | 1 (1 to 2) | 11.24 (7.42 to 16.01) | 0 (0 to 0) | 4.02 (2.72 to 5.67) | -73.8  (-79.3 to -66.58) | -3.97  (-4.41 to -3.53) |
| Belize | 0 (0 to 0) | 44.51 (30.9 to 61.42) | 0 (0 to 0) | 13.57 (9.36 to 18.79) | -60.21  (-67.84 to -51.13) | -4.21  (-4.36 to -4.06) |
| Benin | 56 (39 to 77) | 314.42 (217.49 to 431.66) | 41 (29 to 57) | 108.48 (76.31 to 149.51) | -26.67  (-42.27 to -6.19) | -3.57  (-3.73 to -3.4) |
| Bhutan | 2 (1 to 2) | 108.52 (73.68 to 148.17) | 0 (0 to 0) | 30.41 (20.36 to 42.13) | -82.72  (-85.85 to -78.68) | -4.59  (-4.71 to -4.47) |
| Bolivia (Plurinational State of) | 25 (17 to 34) | 135.71 (94.26 to 184.63) | 8 (6 to 12) | 34.81 (24.49 to 47.6) | -65.74  (-71.68 to -58.49) | -4.58  (-4.62 to -4.53) |
| Bosnia and Herzegovina | 0 (0 to 0) | 5.89 (4.06 to 8.32) | 0 (0 to 0) | 2.09 (1.42 to 3) | -85.97  (-88.81 to -82.23) | -3.96  (-4.16 to -3.76) |
| Botswana | 17 (11 to 24) | 555.23 (353.69 to 796.16) | 11 (8 to 16) | 308.15 (217.03 to 427.89) | -34.28  (-47.5 to -16.01) | -1.1  (-1.67 to -0.52) |
| Brazil | 103 (72 to 142) | 39.7 (27.71 to 54.54) | 21 (15 to 29) | 9.09 (6.34 to 12.48) | -79.26  (-81.47 to -76.62) | -5.77  (-6.25 to -5.3) |
| Bulgaria | 0 (0 to 1) | 6.01 (4.16 to 8.48) | 0 (0 to 0) | 3.32 (2.23 to 4.67) | -66.28  (-73.17 to -57.38) | -1.96  (-2.04 to -1.87) |
| Burkina Faso | 146 (99 to 202) | 425.71 (288.71 to 588.68) | 118 (81 to 164) | 166.88 (115.23 to 233.11) | -19.34  (-35.92 to 3.74) | -3.14  (-3.33 to -2.95) |
| Burundi | 59 (38 to 85) | 300.12 (193.08 to 432.16) | 49 (33 to 69) | 139.25 (93.53 to 196.46) | -17.74  (-35.53 to 10.47) | -2.68  (-2.75 to -2.61) |
| Cabo Verde | 2 (1 to 3) | 223.16 (153.98 to 308.88) | 0 (0 to 1) | 52.2 (36.22 to 72.5) | -78.59  (-83.01 to -72.85) | -5.38  (-5.64 to -5.12) |
| Cambodia | 73 (49 to 103) | 221.8 (150.62 to 313.21) | 24 (17 to 33) | 86.05 (60.32 to 118.21) | -67.2  (-74.76 to -56.88) | -4.17  (-4.58 to -3.75) |
| Cameroon | 109 (74 to 151) | 311.54 (210.94 to 433.2) | 77 (53 to 108) | 113.59 (77.47 to 158.79) | -28.87  (-43.44 to -9.51) | -3.49  (-3.68 to -3.29) |
| Central African Republic | 64 (44 to 89) | 683.8 (472.93 to 950.17) | 91 (65 to 123) | 620.3 (438.19 to 837.42) | 42.12  (18.06 to 68.93) | -0.62  (-1 to -0.23) |
| Chad | 98 (67 to 137) | 412.73 (284.25 to 577.53) | 103 (72 to 143) | 174.84 (122.07 to 241.35) | 5.73  (-16.68 to 36.49) | -2.85  (-2.95 to -2.75) |
| China | 1863 (1194 to 2749) | 102.33 (65.58 to 150.97) | 220 (155 to 304) | 19.35 (13.66 to 26.69) | -88.19  (-89.89 to -85.78) | -6.2  (-6.5 to -5.9) |
| Colombia | 18 (12 to 25) | 25.39 (17.31 to 34.85) | 4 (3 to 5) | 6.67 (4.64 to 8.97) | -77.58  (-81.94 to -72.06) | -4.35  (-4.48 to -4.22) |
| Comoros | 2 (2 to 3) | 153.94 (106.26 to 211.84) | 1 (1 to 1) | 61.18 (41.06 to 86.63) | -66.42  (-73.56 to -56.11) | -3.21  (-3.26 to -3.16) |
| Congo | 24 (16 to 33) | 318.27 (212.7 to 440.44) | 18 (12 to 25) | 166.09 (112.61 to 230.02) | -24.51  (-37.41 to -7.69) | -2.39  (-2.74 to -2.04) |
| Costa Rica | 1 (0 to 1) | 10.16 (6.73 to 14.34) | 0 (0 to 0) | 3.08 (2.1 to 4.37) | -75.09  (-79.56 to -69.67) | -4.24  (-4.43 to -4.06) |
| Côte d'Ivoire | 147 (101 to 201) | 345.73 (238.15 to 471.29) | 80 (54 to 110) | 120.73 (82.13 to 166.44) | -45.7  (-56.79 to -31.57) | -3.76  (-3.88 to -3.64) |
| Cuba | 1 (1 to 2) | 10.85 (7.34 to 15.38) | 0 (0 to 1) | 4.51 (3.07 to 6.45) | -75.95  (-80.35 to -69.68) | -3.04  (-3.14 to -2.95) |
| Democratic People's Republic of Korea | 93 (66 to 127) | 172.08 (121.25 to 233.85) | 21 (15 to 29) | 80.51 (55.8 to 110.79) | -77.47  (-80.95 to -73.46) | -2.44  (-2.81 to -2.06) |
| Democratic Republic of the Congo | 560 (376 to 780) | 400.02 (268.64 to 556.73) | 549 (387 to 750) | 249.61 (175.88 to 340.97) | -1.98  (-18.74 to 17.27) | -1.55  (-1.84 to -1.26) |
| Djibouti | 3 (2 to 4) | 183.46 (125.07 to 254.41) | 2 (1 to 2) | 59.58 (40.96 to 84.59) | -48.93  (-61.17 to -32.85) | -3.73  (-3.83 to -3.63) |
| Dominica | 0 (0 to 0) | 27.01 (18.88 to 37.14) | 0 (0 to 0) | 13.98 (9.82 to 19.38) | -74.81  (-79.24 to -69.81) | -2.3  (-2.47 to -2.12) |
| Dominican Republic | 13 (9 to 18) | 71.59 (50.09 to 98.75) | 4 (3 to 5) | 20.84 (14.96 to 28.62) | -72.1  (-77.06 to -65.19) | -3.93  (-4.14 to -3.72) |
| Ecuador | 13 (9 to 18) | 56.51 (38.8 to 78.16) | 3 (2 to 5) | 12.53 (8.44 to 17.32) | -74.01  (-79.04 to -67.96) | -5.3  (-5.5 to -5.1) |
| Egypt | 15 (9 to 21) | 10.24 (6.66 to 14.92) | 4 (3 to 7) | 2.79 (1.86 to 4.04) | -69.1  (-76.09 to -61.01) | -4.24  (-4.41 to -4.07) |
| El Salvador | 3 (2 to 4) | 21.82 (14.58 to 30.79) | 0 (0 to 0) | 3.82 (2.54 to 5.46) | -87.9  (-90.17 to -85.12) | -6.13  (-6.42 to -5.84) |
| Equatorial Guinea | 6 (4 to 9) | 380.08 (261.9 to 535.81) | 4 (3 to 6) | 140.73 (96.94 to 196.21) | -36.76  (-49.39 to -18.19) | -3.81  (-4.07 to -3.55) |
| Eritrea | 32 (21 to 44) | 337.89 (225.32 to 473.62) | 19 (13 to 27) | 129.6 (89.11 to 178.55) | -38.24  (-51.99 to -19.83) | -3.22  (-3.36 to -3.08) |
| Eswatini | 13 (9 to 19) | 560.36 (377.98 to 779.18) | 8 (5 to 12) | 368.63 (241.46 to 523.96) | -39.18  (-50.79 to -24.01) | -0.73  (-0.96 to -0.5) |
| Ethiopia | 453 (297 to 651) | 246.05 (161.02 to 353.52) | 231 (156 to 320) | 83.86 (56.56 to 116.27) | -49.11  (-56.43 to -40.73) | -4.14  (-4.38 to -3.9) |
| Fiji | 0 (0 to 0) | 6.74 (4.57 to 9.42) | 0 (0 to 0) | 7.02 (4.8 to 9.83) | -3.89  (-20.25 to 14.92) | 0.36  (0.27 to 0.45) |
| Gabon | 9 (6 to 12) | 300.85 (203.59 to 420.58) | 5 (4 to 7) | 169.08 (118.21 to 232.76) | -38.69  (-49.23 to -26.32) | -2.37  (-3.04 to -1.7) |
| Gambia | 19 (14 to 27) | 535.5 (377.8 to 737.45) | 9 (6 to 12) | 162.03 (112.18 to 221.25) | -54.94  (-63.93 to -43.16) | -4.1  (-4.4 to -3.79) |
| Georgia | 2 (1 to 3) | 31.19 (21.57 to 43.8) | 0 (0 to 1) | 12.96 (8.73 to 18.13) | -77.17  (-81.49 to -71.51) | -2.98  (-3.15 to -2.8) |
| Ghana | 155 (106 to 215) | 342.76 (235.17 to 474.73) | 69 (47 to 96) | 106.81 (73.85 to 148.95) | -55.71  (-66.26 to -42.95) | -3.91  (-4.04 to -3.77) |
| Grenada | 0 (0 to 0) | 22.91 (15.75 to 31.68) | 0 (0 to 0) | 10.71 (7.45 to 14.92) | -67.21  (-72.76 to -59.33) | -2.52  (-2.65 to -2.38) |
| Guatemala | 9 (6 to 13) | 35.09 (22.59 to 49.91) | 3 (2 to 4) | 8.73 (5.84 to 12.12) | -69.34  (-76.18 to -60.44) | -4.68  (-4.99 to -4.37) |
| Guinea | 86 (60 to 119) | 366.86 (254.47 to 507.1) | 48 (33 to 67) | 133.09 (92.33 to 187.33) | -44.31  (-55.27 to -27.75) | -3.33  (-3.41 to -3.25) |
| Guinea-Bissau | 12 (8 to 16) | 334.31 (226.26 to 473.45) | 5 (3 to 7) | 102.76 (70.54 to 144.36) | -58.44  (-67.95 to -45.01) | -3.9  (-4.13 to -3.67) |
| Guyana | 1 (1 to 1) | 55.93 (38.19 to 76.82) | 0 (0 to 0) | 19.47 (13.55 to 27.1) | -77.4  (-81.38 to -72.54) | -3.58  (-3.74 to -3.43) |
| Haiti | 40 (27 to 55) | 215.48 (148.38 to 298.85) | 17 (12 to 24) | 68.72 (48.15 to 95.48) | -57.08  (-65.33 to -46.14) | -3.89  (-4.06 to -3.71) |
| Honduras | 5 (3 to 7) | 34.53 (23.57 to 48.28) | 2 (1 to 2) | 9.84 (6.82 to 13.57) | -62.53  (-69.02 to -54.19) | -4.25  (-4.36 to -4.15) |
| India | 1703 (1044 to 2591) | 86.76 (53.19 to 131.97) | 816 (566 to 1115) | 46.05 (31.92 to 62.92) | -52.08  (-59.98 to -40.63) | -1.96  (-2.18 to -1.75) |
| Indonesia | 756 (517 to 1049) | 210.64 (144.03 to 292.27) | 115 (70 to 165) | 39.41 (23.88 to 56.64) | -84.81  (-87.44 to -82.25) | -6.02  (-6.23 to -5.8) |
| Iran (Islamic Republic of) | 11 (7 to 16) | 8.17 (5.33 to 12.13) | 2 (1 to 3) | 2.15 (1.25 to 3.28) | -79.94  (-84.21 to -75.86) | -4.76  (-5.14 to -4.37) |
| Iraq | 14 (9 to 19) | 25.58 (17.44 to 36.33) | 5 (3 to 7) | 6.96 (4.66 to 9.78) | -62.55  (-70.31 to -52.1) | -4.53  (-4.7 to -4.36) |
| Jamaica | 0 (0 to 0) | 8.23 (5.44 to 11.65) | 0 (0 to 0) | 4.31 (2.9 to 6.02) | -66.28  (-72.74 to -58.74) | -2.17  (-2.31 to -2.03) |
| Jordan | 0 (0 to 1) | 3.56 (2.35 to 5.11) | 0 (0 to 0) | 0.85 (0.53 to 1.29) | -56.54  (-67.15 to -45.74) | -5.19  (-5.4 to -4.99) |
| Kazakhstan | 13 (9 to 18) | 49.23 (33.83 to 67.54) | 3 (2 to 4) | 11.57 (7.9 to 16.04) | -76.54  (-81.27 to -70.45) | -5.74  (-6.64 to -4.83) |
| Kenya | 147 (97 to 209) | 195.91 (129.04 to 277.71) | 39 (25 to 55) | 38.19 (24.9 to 54.39) | -73.72  (-76.01 to -71.59) | -6.41  (-6.69 to -6.13) |
| Kiribati | 0 (0 to 0) | 45.28 (30.59 to 63.09) | 0 (0 to 0) | 37.05 (25.82 to 51.91) | -3.56  (-16.28 to 13.49) | -0.59  (-0.65 to -0.53) |
| Kyrgyzstan | 5 (3 to 7) | 52.79 (36.49 to 72.83) | 2 (1 to 3) | 17.37 (11.99 to 24.42) | -61.8  (-69.68 to -51.57) | -3.79  (-4.2 to -3.38) |
| Lao People's Democratic Republic | 9 (6 to 13) | 72.57 (49.73 to 101.61) | 5 (3 to 6) | 34.8 (22.9 to 49.71) | -49.87  (-60.26 to -36.71) | -3.07  (-3.31 to -2.83) |
| Lebanon | 1 (1 to 1) | 10.51 (7.05 to 15.07) | 0 (0 to 0) | 2.77 (1.8 to 4.04) | -74.57  (-80.06 to -66.9) | -4.55  (-4.61 to -4.49) |
| Lesotho | 29 (19 to 41) | 649.64 (426.74 to 915.7) | 18 (12 to 25) | 514.64 (349.38 to 725.34) | -39.09  (-50.55 to -23.52) | -0.61  (-0.73 to -0.49) |
| Liberia | 23 (16 to 31) | 381.35 (266.26 to 522.68) | 9 (7 to 13) | 92.91 (64.61 to 127.22) | -58.87  (-67.56 to -48.23) | -5.05  (-5.18 to -4.91) |
| Libya | 1 (1 to 2) | 10.15 (6.85 to 14.46) | 0 (0 to 0) | 3.4 (2.16 to 5.13) | -81.64  (-85.96 to -76.74) | -3.85  (-3.96 to -3.74) |
| Madagascar | 84 (57 to 114) | 209.7 (143.92 to 285.66) | 55 (38 to 76) | 84.88 (58.47 to 117.4) | -34.58  (-47.38 to -18.43) | -2.98  (-3.1 to -2.86) |
| Malawi | 112 (72 to 162) | 321.91 (207.13 to 464.58) | 67 (48 to 91) | 163.37 (115.78 to 220.87) | -39.74  (-51.82 to -23.57) | -2.25  (-2.41 to -2.1) |
| Malaysia | 12 (9 to 17) | 33.02 (22.59 to 45.9) | 6 (4 to 9) | 15.25 (9.77 to 21.68) | -49.44  (-59.9 to -36.53) | -3.67  (-3.97 to -3.36) |
| Maldives | 0 (0 to 0) | 23.73 (16.02 to 33.81) | 0 (0 to 0) | 9.38 (6.41 to 13.27) | -61.74  (-70.49 to -50.71) | -3.73  (-3.9 to -3.55) |
| Mali | 114 (80 to 155) | 345.67 (243.94 to 472.55) | 90 (62 to 127) | 125.03 (85.37 to 175.71) | -20.61  (-37.45 to 0.88) | -3.6  (-3.79 to -3.4) |
| Marshall Islands | 0 (0 to 0) | 33.05 (22.89 to 46.48) | 0 (0 to 0) | 20.45 (14.11 to 28.04) | -49.62  (-57.32 to -40.92) | -1.5  (-1.82 to -1.18) |
| Mauritania | 14 (9 to 19) | 202.81 (140.83 to 279.43) | 4 (3 to 6) | 52.85 (35.87 to 72.97) | -67.75  (-74.31 to -58.99) | -4.52  (-4.7 to -4.34) |
| Mexico | 29 (19 to 41) | 15.21 (10.07 to 21.65) | 6 (4 to 8) | 3.47 (2.32 to 4.98) | -80.63  (-82.8 to -78.31) | -6.37  (-6.85 to -5.88) |
| Micronesia (Federated States of) | 0 (0 to 0) | 16.46 (11.16 to 22.8) | 0 (0 to 0) | 10.64 (7.08 to 15.24) | -63.63  (-69.74 to -56.1) | -1.59  (-1.63 to -1.55) |
| Mongolia | 4 (3 to 6) | 78.18 (54.24 to 107.26) | 1 (1 to 2) | 23.63 (16.5 to 32.82) | -66.57  (-73.62 to -57.16) | -4.48  (-4.68 to -4.27) |
| Montenegro | 0 (0 to 0) | 2.72 (1.83 to 3.88) | 0 (0 to 0) | 1.02 (0.67 to 1.51) | -74.79  (-80.47 to -67.86) | -3.71  (-3.93 to -3.49) |
| Morocco | 31 (21 to 43) | 51.88 (35.13 to 72.71) | 7 (5 to 10) | 15.25 (9.97 to 21.63) | -77.43  (-82.23 to -71.62) | -4.07  (-4.14 to -4) |
| Mozambique | 95 (57 to 140) | 218.64 (131.46 to 319.96) | 121 (73 to 181) | 143.04 (86.39 to 214.18) | 26.64  (-5.89 to 71.39) | -1.76  (-2.46 to -1.05) |
| Myanmar | 150 (103 to 208) | 151.12 (104.29 to 209.22) | 51 (35 to 71) | 64.55 (44.54 to 90.22) | -66.07  (-72.33 to -58.95) | -3.09  (-3.27 to -2.92) |
| Namibia | 19 (13 to 27) | 531.08 (349.71 to 751.68) | 15 (10 to 22) | 324.12 (215.33 to 461.68) | -20.86  (-35.66 to 1.32) | -1.75  (-1.96 to -1.54) |
| Nepal | 116 (82 to 156) | 192.09 (135.59 to 257.15) | 20 (14 to 28) | 43.69 (29.87 to 59.84) | -82.57  (-85.34 to -78.99) | -4.94  (-5.15 to -4.72) |
| Nicaragua | 4 (2 to 5) | 33.91 (22.92 to 46.82) | 1 (1 to 1) | 7.88 (5.37 to 10.74) | -78.68  (-82.58 to -73.41) | -5.25  (-5.35 to -5.16) |
| Niger | 120 (83 to 164) | 360.66 (250.52 to 492.14) | 102 (72 to 142) | 118.35 (82.92 to 163.93) | -14.72  (-33.26 to 9.26) | -4  (-4.2 to -3.81) |
| Nigeria | 1296 (905 to 1759) | 420.91 (293.72 to 570.98) | 698 (490 to 958) | 123.54 (86.74 to 169.69) | -46.17  (-52.37 to -38.42) | -4.37  (-4.68 to -4.07) |
| North Macedonia | 0 (0 to 0) | 8.21 (5.61 to 11.55) | 0 (0 to 0) | 1.96 (1.33 to 2.77) | -84.81  (-88.03 to -80.67) | -5.19  (-5.39 to -4.99) |
| Pakistan | 975 (669 to 1328) | 272.58 (186.93 to 371.36) | 576 (388 to 793) | 115.69 (77.93 to 159.22) | -40.91  (-47.86 to -32.18) | -2.52  (-2.81 to -2.23) |
| Papua New Guinea | 5 (4 to 7) | 46.43 (32.18 to 64.88) | 10 (7 to 14) | 39.22 (26.64 to 54.96) | 86.07  (57.19 to 119.77) | -0.37  (-0.53 to -0.22) |
| Paraguay | 3 (2 to 4) | 27.18 (18.64 to 36.91) | 1 (1 to 1) | 9.17 (6.08 to 12.78) | -66.53  (-73.23 to -58.51) | -3.85  (-3.99 to -3.71) |
| Peru | 64 (44 to 87) | 128.5 (88.28 to 174.75) | 11 (7 to 15) | 21.8 (14.75 to 30.53) | -83.5  (-86.87 to -80) | -6.48  (-6.72 to -6.24) |
| Philippines | 318 (224 to 439) | 203.24 (143.03 to 280.69) | 321 (222 to 432) | 159.63 (110.52 to 214.96) | 0.93  (-10.8 to 13.19) | 1.37  (-1.66 to 4.5) |
| Republic of Moldova | 1 (1 to 2) | 22.09 (14.86 to 31.02) | 0 (0 to 0) | 9.23 (6.19 to 12.92) | -82.54  (-86.36 to -77.49) | -3.34  (-3.58 to -3.11) |
| Russian Federation | 37 (25 to 52) | 25.19 (17.28 to 35.13) | 10 (7 to 15) | 8.12 (5.49 to 11.65) | -72.07  (-76.51 to -66.31) | -4.43  (-4.82 to -4.04) |
| Rwanda | 42 (27 to 60) | 177.23 (114.83 to 251.68) | 16 (11 to 23) | 60.24 (41.06 to 85.36) | -61.97  (-70.79 to -49.43) | -3.79  (-3.93 to -3.64) |
| Saint Lucia | 0 (0 to 0) | 22.65 (15.35 to 31.59) | 0 (0 to 0) | 7.22 (4.96 to 9.96) | -84.97  (-87.8 to -81.34) | -3.99  (-4.1 to -3.87) |
| Saint Vincent and the Grenadines | 0 (0 to 0) | 27.1 (18.47 to 37.2) | 0 (0 to 0) | 10.25 (7.06 to 14.11) | -78.33  (-82.14 to -73.71) | -3.28  (-3.46 to -3.11) |
| Samoa | 0 (0 to 0) | 10.83 (7.36 to 15.35) | 0 (0 to 0) | 7.77 (5.22 to 10.87) | -19.69  (-31.98 to -5.2) | -0.91  (-1.08 to -0.73) |
| Senegal | 74 (51 to 100) | 286.54 (200.56 to 391.13) | 29 (19 to 39) | 81.66 (55.64 to 112.71) | -61.21  (-69.04 to -50.78) | -4.36  (-4.48 to -4.25) |
| Serbia | 0 (0 to 1) | 4.54 (3.01 to 6.56) | 0 (0 to 0) | 1.47 (0.94 to 2.19) | -79.79  (-84.5 to -73.8) | -3.95  (-4.15 to -3.74) |
| Sierra Leone | 50 (35 to 69) | 427.06 (294.88 to 588.55) | 38 (27 to 54) | 181.77 (126.68 to 255.79) | -23.73  (-39.79 to -3.36) | -3.1  (-3.31 to -2.89) |
| Solomon Islands | 0 (0 to 0) | 9.18 (6.19 to 12.89) | 0 (0 to 0) | 6.83 (4.53 to 9.88) | 9  (-7.5 to 29.6) | -0.74  (-0.99 to -0.5) |
| Somalia | 39 (27 to 53) | 168.85 (116.68 to 231.53) | 68 (46 to 97) | 104.5 (70.81 to 148.53) | 75.41  (40.25 to 120.66) | -1.42  (-1.56 to -1.28) |
| South Africa | 572 (401 to 781) | 780.35 (546.14 to 1064.38) | 215 (147 to 295) | 276.05 (189.17 to 378.62) | -62.42  (-65.49 to -59.13) | -4.44  (-5.38 to -3.5) |
| South Sudan | 31 (21 to 43) | 166.93 (112.65 to 231.88) | 27 (19 to 37) | 99.17 (69.88 to 137.23) | -14.1  (-31.91 to 10.15) | -1.63  (-1.87 to -1.39) |
| Sri Lanka | 3 (2 to 4) | 11.71 (7.93 to 16.51) | 2 (1 to 3) | 8.7 (6 to 12.24) | -38.01  (-49.2 to -24.67) | -1.24  (-1.64 to -0.84) |
| Sudan | 19 (12 to 27) | 26.1 (17.14 to 37.67) | 8 (5 to 11) | 8.76 (5.9 to 12.39) | -59.06  (-67.44 to -48.78) | -3.53  (-3.58 to -3.48) |
| Suriname | 0 (0 to 0) | 20.83 (13.93 to 28.95) | 0 (0 to 0) | 7.29 (4.96 to 10.14) | -65.26  (-71.88 to -57.34) | -3.67  (-3.87 to -3.48) |
| Syrian Arab Republic | 3 (2 to 5) | 8.35 (5.49 to 11.93) | 0 (0 to 1) | 2.49 (1.61 to 3.6) | -86.09  (-88.97 to -82.28) | -4.09  (-4.39 to -3.8) |
| Tajikistan | 6 (4 to 8) | 39.44 (27.23 to 54.03) | 4 (2 to 5) | 18.5 (12.83 to 25.41) | -41.54  (-52.75 to -28.39) | -2.66  (-2.81 to -2.51) |
| Thailand | 54 (36 to 76) | 67.53 (45.16 to 95.1) | 10 (7 to 15) | 23.43 (16.04 to 32.69) | -80.76  (-84.88 to -75.8) | -4.5  (-4.8 to -4.21) |
| Timor-Leste | 2 (1 to 2) | 61.55 (41.2 to 84.77) | 1 (1 to 2) | 38.3 (26.33 to 53.14) | -32.71  (-45.43 to -17.13) | -2.13  (-2.33 to -1.93) |
| Togo | 40 (27 to 54) | 324.17 (225.05 to 441.24) | 20 (14 to 28) | 112.24 (77.19 to 158.53) | -49.62  (-59.53 to -37.23) | -3.74  (-3.88 to -3.61) |
| Tonga | 0 (0 to 0) | 7.58 (5.16 to 10.53) | 0 (0 to 0) | 6.3 (4.21 to 8.8) | -25.19  (-36.93 to -11.87) | -0.52  (-0.61 to -0.43) |
| Tunisia | 2 (1 to 3) | 11.59 (7.79 to 16.36) | 0 (0 to 1) | 2.91 (1.93 to 4.05) | -82.16  (-85.95 to -76.96) | -4.67  (-4.77 to -4.57) |
| Turkey | 26 (17 to 37) | 20.67 (13.88 to 29.81) | 3 (2 to 4) | 3.77 (2.52 to 5.22) | -89.05  (-91.54 to -85.66) | -6.17  (-6.34 to -6) |
| Turkmenistan | 4 (3 to 6) | 41.7 (28.49 to 57.58) | 1 (1 to 2) | 15.1 (10.12 to 20.97) | -68.91  (-75.05 to -61.42) | -3.49  (-3.7 to -3.28) |
| Tuvalu | 0 (0 to 0) | 16.63 (11.21 to 23.55) | 0 (0 to 0) | 7.74 (5.32 to 10.84) | -64.62  (-71.08 to -56.26) | -2.64  (-2.81 to -2.48) |
| Uganda | 174 (95 to 270) | 251.75 (137.74 to 390.45) | 114 (78 to 161) | 96.25 (65.38 to 135.36) | -34.4  (-50.44 to -7.99) | -3.02  (-3.15 to -2.88) |
| Ukraine | 7 (4 to 9) | 13.07 (8.87 to 18.57) | 3 (2 to 4) | 8.54 (5.77 to 12.28) | -61.3  (-69.11 to -51.12) | -1.62  (-1.84 to -1.4) |
| United Republic of Tanzania | 223 (138 to 331) | 244.92 (151.29 to 364.5) | 136 (92 to 191) | 87.3 (59.44 to 123.11) | -39.16  (-54.99 to -17.07) | -3.18  (-3.53 to -2.83) |
| Uzbekistan | 23 (16 to 32) | 43.03 (29.55 to 58.94) | 9 (6 to 12) | 16.44 (11.46 to 22.43) | -61.54  (-69.62 to -51.39) | -3.4  (-3.59 to -3.21) |
| Vanuatu | 0 (0 to 0) | 14.77 (10.06 to 20.62) | 0 (0 to 0) | 13.41 (9.09 to 18.64) | 23.02  (2.21 to 48.34) | -0.06  (-0.19 to 0.07) |
| Venezuela (Bolivarian Republic of) | 12 (8 to 17) | 27.92 (18.55 to 38.3) | 3 (2 to 4) | 7.54 (5.11 to 10.38) | -77.54  (-81.41 to -72.62) | -4.56  (-4.93 to -4.19) |
| Viet Nam | 103 (70 to 143) | 74.28 (50.99 to 103.87) | 36 (25 to 51) | 35.83 (24.31 to 50.13) | -64.52  (-70.35 to -56.92) | -2.33  (-2.64 to -2.03) |
| Yemen | 12 (8 to 18) | 24.21 (16.31 to 35.6) | 6 (4 to 9) | 8.98 (5.99 to 12.86) | -48.31  (-59.33 to -34.9) | -3.51  (-3.59 to -3.43) |
| Zambia | 89 (53 to 134) | 309.46 (184.15 to 466.22) | 62 (39 to 91) | 135.09 (83.74 to 196.08) | -29.75  (-47.29 to -3.89) | -3.01  (-3.2 to -2.82) |
| Zimbabwe | 102 (53 to 163) | 354.37 (183.6 to 564.59) | 88 (57 to 127) | 262.28 (169.91 to 378.33) | -14.13  (-36.75 to 24.16) | -0.28  (-0.56 to 0) |

CI, confidence interval; EAPC, estimated annual percentage change; LMICs, low- and middle-income countries and territories; UI, uncertain interval

## Supplementary Table S20. National burden and trends of incident cases and incidence rates (per 100,000 liveborn neonates) of upper respiratory infections in 131 LMICs between 1990 and 2019.

| **Location** | **Incident cases in 1990**  **(95% UI)** | **Incident rate in 1990**  **(95% UI)** | **Incident cases in 2019**  **(95% UI)** | **Incident rate in 2019**  **(95% UI)** | **Percent change**  **(%, 95% CI)** | **EAPC**  **(%, 95% CI)** |
| --- | --- | --- | --- | --- | --- | --- |
| Global | 32591416 (25160925 to 42035903) | 315668.68 (243699.64 to 407144.58) | 31337636 (24164899 to 40798121) | 306679.94 (236485.29 to 399263.22) | -3.85  (-5.43 to -2.57) | -0.19  (-0.25 to -0.13) |
| Afghanistan | 130298 (98662 to 170870) | 336777.18 (255009.36 to 441644.72) | 378417 (287614 to 500004) | 338355.08 (257165.05 to 447069.84) | 190.42  (163.78 to 222.51) | 0  (0 to 0.01) |
| Albania | 13294 (9957 to 17559) | 204464.67 (153135.39 to 270064.29) | 5447 (4089 to 7177) | 204417.08 (153463.97 to 269348.79) | -59.03  (-62.76 to -53.86) | -0.08  (-0.17 to 0.02) |
| Algeria | 196798 (149322 to 258571) | 338836.15 (257093.98 to 445194.06) | 225047 (170935 to 290663) | 339342.52 (257748.53 to 438284.47) | 14.35  (3.47 to 27.08) | 0  (-0.01 to 0.01) |
| American Samoa | 365 (278 to 472) | 281737.13 (214918.76 to 364567.07) | 227 (171 to 294) | 284140.76 (213272.14 to 368054.68) | -37.67  (-43.33 to -31.53) | -0.04  (-0.07 to -0.01) |
| Angola | 178242 (135789 to 235330) | 470362.09 (358331.54 to 621008.91) | 391551 (297053 to 514426) | 469626.84 (356286.63 to 617004.41) | 119.67  (98.71 to 142.3) | 0.01  (0 to 0.02) |
| Armenia | 12304 (9279 to 16241) | 214242.97 (161564.87 to 282806.11) | 6134 (4649 to 7968) | 214501.86 (162563.27 to 278622.9) | -50.14  (-55.61 to -43.97) | -0.18  (-0.26 to -0.11) |
| Azerbaijan | 20054 (15166 to 26469) | 140202.81 (106034.36 to 185056.22) | 15296 (11556 to 20522) | 140723.44 (106310.3 to 188801.02) | -23.72  (-31.22 to -15.92) | -0.02  (-0.06 to 0.02) |
| Bangladesh | 1065887 (801303 to 1386625) | 364135.96 (273746.97 to 473708.88) | 736721 (557825 to 964704) | 362619.42 (274565.43 to 474834.44) | -30.88  (-37.38 to -23.6) | -0.11  (-0.38 to 0.17) |
| Belarus | 23722 (18341 to 31121) | 221208.68 (171032.06 to 290196.03) | 17299 (13279 to 22818) | 220160.02 (168989.91 to 290387.47) | -27.08  (-34.84 to -18.72) | 0.01  (0 to 0.03) |
| Belize | 1588 (1198 to 2106) | 356563.18 (268869.8 to 472711.69) | 2049 (1547 to 2674) | 352395.69 (265990.89 to 459879.88) | 29  (17.79 to 43.76) | -0.02  (-0.03 to -0.01) |
| Benin | 38972 (29237 to 50609) | 219040.74 (164328.12 to 284443.93) | 82984 (63116 to 107284) | 219436.01 (166898.83 to 283692.06) | 112.93  (91.82 to 141.96) | -0.65  (-0.87 to -0.43) |
| Bhutan | 4656 (3556 to 6087) | 283333.55 (216359.74 to 370384.12) | 2868 (2167 to 3743) | 283017.66 (213786.47 to 369326.48) | -38.4  (-44.25 to -31.89) | -0.01  (-0.01 to -0.01) |
| Bolivia (Plurinational State of) | 54095 (42174 to 69835) | 296211.93 (230933.51 to 382399.29) | 73956 (56057 to 96140) | 303153.39 (229783.43 to 394086.73) | 36.72  (20.41 to 56.09) | 0.15  (0.07 to 0.23) |
| Bosnia and Herzegovina | 10627 (8044 to 13906) | 205538.12 (155591.1 to 268971.22) | 4198 (3164 to 5543) | 205221.78 (154678.89 to 271008.31) | -60.5  (-64.5 to -55.81) | 0  (-0.02 to 0.03) |
| Botswana | 9496 (7216 to 12405) | 308934.65 (234741.18 to 403560.74) | 11169 (8491 to 14444) | 306853.78 (233282.64 to 396830.36) | 17.62  (7.89 to 30.13) | -0.03  (-0.03 to -0.02) |
| Brazil | 1267294 (969566 to 1654381) | 488278.47 (373566.05 to 637420.19) | 1168406 (883387 to 1537803) | 497239.23 (375943.78 to 654443.89) | -7.8  (-11.58 to -4.14) | -0.14  (-0.25 to -0.02) |
| Bulgaria | 15360 (11490 to 20074) | 204716.16 (153132.83 to 267534.84) | 9351 (7126 to 12387) | 204285.44 (155673.75 to 270624.31) | -39.12  (-45.62 to -32.25) | 0.01  (-0.01 to 0.02) |
| Burkina Faso | 93676 (69507 to 123150) | 273283.46 (202773.03 to 359267.77) | 170621 (129962 to 220778) | 241899.94 (184255.7 to 313010.87) | 82.14  (46.81 to 110.01) | -1.26  (-1.56 to -0.96) |
| Burundi | 42092 (31330 to 55110) | 212783.72 (158376.57 to 278589.56) | 74563 (57270 to 96484) | 212596.31 (163290.21 to 275099.37) | 77.14  (57.6 to 99.96) | -0.22  (-0.35 to -0.09) |
| Cabo Verde | 2013 (1534 to 2646) | 226368.47 (172429.38 to 297555.31) | 1833 (1375 to 2379) | 225201.99 (168931.33 to 292209.57) | -8.93  (-19.08 to 1.79) | -0.02  (-0.03 to -0.01) |
| Cambodia | 92117 (69381 to 120613) | 280999.43 (211643.36 to 367925.44) | 77549 (59076 to 102189) | 279846.65 (213184.98 to 368763.43) | -15.81  (-24.44 to -6.6) | 0.09  (0.01 to 0.16) |
| Cameroon | 112931 (85955 to 143912) | 324098.32 (246679.6 to 413008.84) | 199598 (152308 to 261661) | 293632.24 (224062.1 to 384934.2) | 76.74  (51.45 to 104.1) | -0.42  (-0.56 to -0.28) |
| Central African Republic | 53148 (41908 to 66908) | 565056.86 (445550.87 to 711343.11) | 78408 (59523 to 101787) | 532068.18 (403916.13 to 690721.79) | 47.53  (25.8 to 69.91) | -0.28  (-0.33 to -0.23) |
| Chad | 59007 (45020 to 77148) | 248936.04 (189929.41 to 325472.41) | 147662 (112669 to 195835) | 249593.64 (190445.83 to 331021.32) | 150.25  (123.08 to 176.92) | -0.09  (-0.27 to 0.09) |
| China | 4707182 (3619591 to 6090089) | 258480.39 (198758.66 to 334418.41) | 2999938 (2320212 to 3859215) | 263775.73 (204009.44 to 339329.44) | -36.27  (-37.93 to -34.95) | -0.04  (-0.09 to 0.01) |
| Colombia | 285040 (224668 to 361571) | 404084.02 (318498.05 to 512577.14) | 256337 (195962 to 334500) | 425660.68 (325403.77 to 555453.02) | -10.07  (-21.82 to 3.33) | 0.67  (0.37 to 0.96) |
| Comoros | 4699 (3548 to 6118) | 319408.82 (241163.45 to 415860.09) | 3931 (3016 to 5129) | 316233.18 (242636.24 to 412592.61) | -16.35  (-24.56 to -8.53) | -0.08  (-0.1 to -0.06) |
| Congo | 33326 (25641 to 43398) | 443917.55 (341549.87 to 578082.9) | 48326 (36495 to 64140) | 445000.95 (336050.03 to 590612.57) | 45.01  (30.48 to 59.77) | -0.04  (-0.07 to 0) |
| Costa Rica | 20436 (15538 to 26643) | 331785.52 (252265.74 to 432554.05) | 16614 (12563 to 21837) | 328349.84 (248287.14 to 431583.79) | -18.7  (-26.57 to -10.63) | -0.05  (-0.06 to -0.04) |
| Côte d'Ivoire | 120263 (89574 to 155889) | 282265.21 (210235.67 to 365881.97) | 169815 (128466 to 224106) | 256347.09 (193927.83 to 338303.34) | 41.2  (11.95 to 62.04) | -0.61  (-0.71 to -0.52) |
| Cuba | 49415 (37146 to 64644) | 358129.23 (269208.09 to 468493.65) | 28373 (21567 to 37649) | 355386.93 (270144.99 to 471584.9) | -42.58  (-48.88 to -34.91) | -0.05  (-0.06 to -0.03) |
| Democratic People's Republic of Korea | 135637 (102288 to 177992) | 250051.58 (188572.6 to 328134.75) | 66812 (50739 to 87225) | 255746.97 (194221 to 333883.94) | -50.74  (-56.51 to -45.58) | -0.04  (-0.09 to 0.01) |
| Democratic Republic of the Congo | 599639 (452485 to 793654) | 428089.67 (323034.41 to 566598.84) | 934121 (709008 to 1230512) | 424521.33 (322216.42 to 559219.49) | 55.78  (39.79 to 72.57) | -0.23  (-0.3 to -0.16) |
| Djibouti | 5124 (3856 to 6761) | 308917.23 (232469.84 to 407581.47) | 8118 (6066 to 10784) | 311198.52 (232522.65 to 413388.71) | 58.43  (43.66 to 76.9) | 0.01  (0 to 0.02) |
| Dominica | 461 (348 to 603) | 355660.83 (268512.83 to 465730.64) | 223 (169 to 290) | 353136.69 (268769.69 to 459415.06) | -51.68  (-56.49 to -46.87) | -0.02  (-0.03 to -0.02) |
| Dominican Republic | 58471 (45554 to 74430) | 324568.33 (252865.63 to 413156.81) | 56014 (42155 to 73885) | 324459.51 (244182.41 to 427982.23) | -4.2  (-16.5 to 9.39) | -0.26  (-0.41 to -0.1) |
| Ecuador | 76201 (57558 to 99649) | 336390.05 (254092.46 to 439905.6) | 89315 (68101 to 115486) | 336322.93 (256442.03 to 434871.96) | 17.21  (6.65 to 33.08) | 0  (0 to 0) |
| Egypt | 448701 (347791 to 558068) | 315835.65 (244806.01 to 392817.9) | 412372 (308347 to 533502) | 256118.72 (191510.62 to 331351.43) | -8.1  (-27.92 to 8.19) | -0.8  (-0.95 to -0.65) |
| El Salvador | 40734 (30765 to 53200) | 328879.89 (248387.66 to 429526.11) | 27953 (21120 to 36281) | 326736.8 (246872.07 to 424085.2) | -31.38  (-38.52 to -22.88) | -0.03  (-0.04 to -0.02) |
| Equatorial Guinea | 8030 (6046 to 10553) | 469603.18 (353546.18 to 617113.13) | 13666 (10466 to 17821) | 467896.43 (358350.98 to 610157.76) | 70.17  (54.35 to 86.93) | 0  (-0.01 to 0) |
| Eritrea | 28677 (21655 to 37490) | 307527.23 (232223.86 to 402045.43) | 45972 (34799 to 59942) | 306194.59 (231777.65 to 399241.13) | 60.31  (43.5 to 77.2) | -0.01  (-0.02 to 0) |
| Eswatini | 7009 (5320 to 9145) | 291893.07 (221554.2 to 380876.52) | 6419 (4868 to 8407) | 289160.69 (219303.7 to 378733.87) | -8.41  (-17.51 to 2.55) | -0.03  (-0.03 to -0.02) |
| Ethiopia | 396162 (290676 to 531374) | 215097.94 (157823.89 to 288511.55) | 593520 (437460 to 797939) | 215805.05 (159061.32 to 290132.06) | 49.82  (41.7 to 58.78) | -0.11  (-0.38 to 0.16) |
| Fiji | 4055 (3085 to 5324) | 281460.46 (214186.86 to 369576.32) | 3757 (2875 to 4916) | 282536.59 (216208.71 to 369684.73) | -7.34  (-16.52 to 2.31) | -0.05  (-0.07 to -0.02) |
| Gabon | 14239 (10833 to 18268) | 482151.58 (366818.3 to 618597.14) | 15645 (11900 to 20680) | 485623.46 (369383.09 to 641934.33) | 9.87  (-0.67 to 23.22) | -0.06  (-0.08 to -0.03) |
| Gambia | 5488 (4106 to 7297) | 152325.29 (113949.3 to 202517.85) | 8251 (6109 to 10921) | 153774.27 (113851.39 to 203538.11) | 50.33  (34.06 to 66.63) | -0.26  (-0.36 to -0.17) |
| Georgia | 9105 (6865 to 11973) | 144065.66 (108628.91 to 189447.47) | 5020 (3762 to 6584) | 144548.82 (108318.11 to 189576.36) | -44.87  (-50.57 to -38.54) | 0  (-0.01 to 0.01) |
| Ghana | 101358 (77105 to 132555) | 224276.06 (170610.89 to 293305.36) | 144025 (109019 to 185468) | 224207.33 (169713.69 to 288722.68) | 42.09  (24.97 to 60.08) | -0.11  (-0.17 to -0.05) |
| Grenada | 536 (409 to 690) | 355877.54 (271770.53 to 458619.02) | 376 (283 to 504) | 355784.66 (268469.68 to 477581.02) | -29.87  (-37.73 to -21.37) | -0.01  (-0.02 to 0) |
| Guatemala | 72155 (57349 to 89857) | 284039.53 (225755.48 to 353723.45) | 89818 (67373 to 119102) | 287025.98 (215300.47 to 380609.71) | 24.48  (5.69 to 45.53) | 0.06  (0.04 to 0.08) |
| Guinea | 51715 (39123 to 67212) | 220762.3 (167009.01 to 286918.41) | 79210 (59421 to 102312) | 220262.59 (165234.57 to 284500.42) | 53.17  (36.94 to 69.72) | -0.32  (-0.52 to -0.12) |
| Guinea-Bissau | 7855 (5884 to 10370) | 226893.19 (169970.86 to 299537.81) | 10610 (8045 to 13888) | 226689.36 (171900.74 to 296737.4) | 35.07  (21.3 to 51.07) | -0.01  (-0.02 to 0) |
| Guyana | 5881 (4508 to 7694) | 352321.24 (270039.2 to 460945.7) | 3782 (2896 to 4979) | 349043.26 (267246.66 to 459488.88) | -35.69  (-41.95 to -28.79) | -0.06  (-0.12 to 0) |
| Haiti | 83536 (62298 to 107789) | 452284.28 (337296.89 to 583593.78) | 98287 (74479 to 127809) | 395390.39 (299614.91 to 514153.2) | 17.66  (-5.07 to 36.71) | -0.91  (-1.07 to -0.75) |
| Honduras | 45397 (34533 to 59693) | 334713.41 (254612.31 to 440123.71) | 59409 (45578 to 77138) | 333132.73 (255574.31 to 432546.24) | 30.87  (17.69 to 47.74) | -0.3  (-0.45 to -0.16) |
| India | 5266420 (4057744 to 6806248) | 268239.29 (206676.7 to 346668.71) | 4588863 (3524781 to 5944851) | 258870.85 (198842.94 to 335365.96) | -12.87  (-15.26 to -10.94) | -0.05  (-0.5 to 0.4) |
| Indonesia | 1409903 (1082415 to 1824866) | 392980.41 (301699.95 to 508642.45) | 1150377 (875090 to 1507374) | 394972.7 (300455.17 to 517544.95) | -18.41  (-22.98 to -14.1) | 0.17  (0.08 to 0.27) |
| Iran (Islamic Republic of) | 466780 (356988 to 603959) | 347143.32 (265491.54 to 449163.34) | 356511 (273239 to 464926) | 347634.88 (266435.39 to 453350.21) | -23.62  (-25.24 to -21.76) | 0  (0 to 0) |
| Iraq | 179041 (136264 to 235074) | 338827.76 (257875.16 to 444868.93) | 246493 (186666 to 324629) | 338809.62 (256575.82 to 446209.7) | 37.67  (23.02 to 52.4) | -0.01  (-0.02 to 0) |
| Jamaica | 15166 (11575 to 19959) | 357079.54 (272535.87 to 469920.71) | 9734 (7471 to 12788) | 355487.71 (272854.77 to 467021.22) | -35.82  (-43.29 to -28.96) | -0.01  (-0.02 to 0) |
| Jordan | 25233 (19034 to 32611) | 244462.42 (184408.64 to 315938.82) | 45655 (34500 to 59022) | 244310.65 (184616.98 to 315836.57) | 80.93  (62.44 to 101.84) | -0.33  (-0.49 to -0.17) |
| Kazakhstan | 49521 (38341 to 64010) | 183863.23 (142353.17 to 237656.66) | 49253 (37479 to 64147) | 183277.15 (139462.64 to 238699.62) | -0.54  (-11.74 to 11.61) | -0.06  (-0.07 to -0.05) |
| Kenya | 324070 (245448 to 425887) | 430636.69 (326161.56 to 565935.8) | 420945 (318117 to 549819) | 415010.11 (313632.42 to 542067.52) | 29.89  (27.05 to 32.49) | -0.24  (-0.38 to -0.1) |
| Kiribati | 548 (415 to 715) | 282305.39 (213836.07 to 368559.82) | 654 (498 to 854) | 285910.26 (217857.33 to 373352.93) | 19.36  (6.65 to 32.69) | -0.02  (-0.05 to 0.01) |
| Kyrgyzstan | 12511 (9238 to 16687) | 131510.17 (97110.95 to 175411.88) | 14566 (10801 to 19435) | 131859.08 (97783.53 to 175938.78) | 16.42  (5.42 to 30) | -0.5  (-0.64 to -0.37) |
| Lao People's Democratic Republic | 43070 (32477 to 55933) | 348086.44 (262475.3 to 452043.76) | 44854 (33763 to 58733) | 346730.92 (260990.69 to 454017.25) | 4.14  (-6.47 to 14.37) | -0.04  (-0.05 to -0.02) |
| Lebanon | 28094 (21069 to 36781) | 338433.11 (253806.7 to 443082.23) | 27302 (20603 to 35674) | 340251.66 (256768.89 to 444596.34) | -2.82  (-13.14 to 10.29) | -0.01  (-0.01 to 0) |
| Lesotho | 14052 (10604 to 18300) | 310247.52 (234121.36 to 404045.29) | 10748 (8130 to 13940) | 308602.82 (233446.7 to 400266.56) | -23.51  (-30.19 to -15.84) | 0.2  (0.09 to 0.31) |
| Liberia | 15351 (11660 to 20061) | 256015 (194461.28 to 334568.61) | 25908 (19595 to 34562) | 255959.33 (193590.94 to 341457.25) | 68.77  (50.75 to 89.2) | 0.13  (0.06 to 0.2) |
| Libya | 38181 (28981 to 50144) | 337434.9 (256125.11 to 443158.75) | 21069 (16049 to 27631) | 339253.71 (258413.39 to 444920.15) | -44.82  (-49.76 to -38.31) | 0  (-0.01 to 0.01) |
| Madagascar | 127832 (94448 to 176375) | 320338.23 (236680.29 to 441985.63) | 183258 (138268 to 236425) | 284167.05 (214402.78 to 366609.23) | 43.36  (4.29 to 66.72) | -0.68  (-0.94 to -0.43) |
| Malawi | 136315 (101406 to 181634) | 391994.02 (291607.43 to 522315.46) | 141690 (108292 to 187771) | 343153.62 (262267.94 to 454755.49) | 3.94  (-19.33 to 21.36) | -0.83  (-0.97 to -0.68) |
| Malaysia | 130932 (98914 to 172588) | 346517.84 (261780.6 to 456763.85) | 142899 (109155 to 187638) | 345389.96 (263830.62 to 453525.51) | 9.14  (-0.03 to 19.84) | -0.02  (-0.03 to -0.02) |
| Maldives | 2190 (1660 to 2867) | 330891.61 (250888.84 to 433245.94) | 2123 (1621 to 2778) | 331296.18 (252903.2 to 433415.47) | -3.04  (-12.18 to 8.34) | -0.1  (-0.21 to 0.01) |
| Mali | 71162 (54032 to 91191) | 216619.06 (164474.48 to 277589.45) | 143331 (107933 to 183985) | 198773.18 (149683.06 to 255151.98) | 101.42  (62.73 to 128.96) | -0.42  (-0.53 to -0.32) |
| Marshall Islands | 324 (247 to 423) | 281594.1 (214677.3 to 368177.66) | 267 (202 to 351) | 285042.05 (215522.22 to 374494.82) | -17.58  (-25.14 to -7.54) | -0.03  (-0.06 to 0) |
| Mauritania | 15105 (11319 to 19658) | 226565.59 (169780.95 to 294867.77) | 18730 (14143 to 24526) | 227004.54 (171411.78 to 297241.41) | 24  (12.48 to 38.99) | 0  (0 to 0.01) |
| Mexico | 646926 (493878 to 844301) | 344601.85 (263076.85 to 449738.81) | 542530 (413707 to 709634) | 340492.33 (259643.21 to 445367.32) | -16.14  (-18.2 to -14.06) | -0.06  (-0.07 to -0.05) |
| Micronesia (Federated States of) | 743 (567 to 980) | 282826.46 (215905.36 to 372781.94) | 423 (324 to 560) | 286103.7 (218990.69 to 378785.63) | -43.06  (-48.22 to -35.55) | -0.03  (-0.06 to 0) |
| Mongolia | 7916 (5903 to 10449) | 143712.3 (107171.43 to 189707.97) | 8761 (6617 to 11533) | 143803.38 (108609.12 to 189306.1) | 10.68  (-0.98 to 25.92) | -0.02  (-0.03 to -0.01) |
| Montenegro | 1554 (1166 to 2041) | 204310.84 (153372.63 to 268397.06) | 1045 (789 to 1366) | 204559.71 (154337.38 to 267271.56) | -32.74  (-39.6 to -24.74) | 0.01  (0 to 0.03) |
| Morocco | 211662 (167436 to 262028) | 356053.6 (281656.54 to 440777.69) | 167643 (126842 to 222819) | 367237.83 (277858.66 to 488104.53) | -20.8  (-31.75 to -5.65) | 0.11  (0.07 to 0.15) |
| Mozambique | 107821 (81821 to 141504) | 246959.57 (187408.15 to 324109.99) | 208803 (156655 to 269956) | 247071.54 (185366.13 to 319432.62) | 93.66  (71.41 to 120.37) | -0.52  (-0.7 to -0.34) |
| Myanmar | 344134 (259799 to 450957) | 346785.31 (261800.21 to 454430.95) | 270269 (205637 to 354901) | 342882.47 (260886.25 to 450252.26) | -21.46  (-29.74 to -12.39) | -0.04  (-0.05 to -0.04) |
| Namibia | 13254 (9827 to 17564) | 363524.56 (269521.69 to 481718.41) | 15160 (11663 to 19865) | 320643.3 (246680.09 to 420155.73) | 14.38  (-10.88 to 31.57) | -0.09  (-0.39 to 0.21) |
| Nepal | 181115 (137407 to 236207) | 298638.51 (226568.82 to 389479.06) | 137235 (104668 to 179533) | 295238.5 (225176.71 to 386235.56) | -24.23  (-30.91 to -16.83) | -0.05  (-0.16 to 0.07) |
| Nicaragua | 30952 (23316 to 39932) | 285003.89 (214695.63 to 367690.39) | 28103 (21439 to 36457) | 282023.39 (215151.5 to 365854.04) | -9.2  (-19.46 to 0.21) | 0.21  (0.04 to 0.39) |
| Niger | 72320 (55184 to 90781) | 217598.17 (166038.53 to 273145.7) | 178051 (135130 to 231423) | 206155.12 (156459.53 to 267950.66) | 146.2  (111.17 to 180.73) | -0.53  (-0.64 to -0.43) |
| Nigeria | 737115 (557842 to 971417) | 239324.66 (181118.87 to 315397.12) | 1283011 (968437 to 1700192) | 227150.17 (171456.51 to 301009.83) | 74.06  (67.67 to 80.94) | -0.82  (-1.06 to -0.59) |
| North Macedonia | 5437 (4093 to 7195) | 204522.26 (153972.2 to 270667.88) | 3464 (2620 to 4583) | 204532.89 (154695.94 to 270617.73) | -36.29  (-42.4 to -28.43) | -0.01  (-0.03 to 0.01) |
| Pakistan | 968768 (730349 to 1272116) | 270863.53 (204202.78 to 355678.64) | 1208187 (926850 to 1561368) | 242627.39 (186129.47 to 313553.02) | 24.71  (14.9 to 33.5) | -0.7  (-0.85 to -0.55) |
| Papua New Guinea | 31732 (24119 to 41596) | 282957.74 (215071.39 to 370920.71) | 70595 (53216 to 92878) | 285768.06 (215419.11 to 375970.68) | 122.47  (99.89 to 145.01) | -0.03  (-0.06 to 0) |
| Paraguay | 47657 (36214 to 62914) | 492261.96 (374063.14 to 649857.13) | 46881 (35414 to 61001) | 488187.68 (368780.74 to 635228.89) | -1.63  (-11.37 to 8.23) | -0.02  (-0.03 to -0.01) |
| Peru | 186135 (141744 to 245574) | 372644.15 (283772.75 to 491641.07) | 180672 (137939 to 238137) | 372019.24 (284027.74 to 490344.39) | -2.93  (-11.92 to 6.63) | -0.52  (-0.72 to -0.31) |
| Philippines | 763229 (584727 to 996702) | 488369.6 (374151.29 to 637763.11) | 760552 (579811 to 994135) | 378698.1 (288702.64 to 495004.66) | -0.35  (-3.84 to 3.46) | -0.97  (-1.2 to -0.75) |
| Republic of Moldova | 12991 (9837 to 17000) | 221679.33 (167853.98 to 290095.75) | 5402 (4106 to 7003) | 220509.87 (167609.21 to 285846.89) | -58.41  (-61.9 to -53.89) | -0.03  (-0.15 to 0.08) |
| Russian Federation | 334727 (256344 to 433174) | 227390.12 (174141.88 to 294268.15) | 287602 (221698 to 372969) | 225572.66 (173882.6 to 292528.25) | -14.08  (-15.58 to -12.56) | 0  (-0.01 to 0.02) |
| Rwanda | 74068 (58265 to 93344) | 311438.32 (244989.7 to 392488.53) | 82550 (63356 to 107907) | 310213.16 (238086.6 to 405502.37) | 11.45  (-3.52 to 29.08) | -0.06  (-0.1 to -0.01) |
| Saint Lucia | 1016 (779 to 1319) | 354519.2 (271610 to 460015.95) | 479 (362 to 628) | 353952.51 (267983.28 to 464615.65) | -52.91  (-57.49 to -47.48) | -0.01  (-0.02 to -0.01) |
| Saint Vincent and the Grenadines | 714 (542 to 931) | 355595.44 (269910.41 to 463434.77) | 407 (311 to 524) | 353426.77 (269907.15 to 455092.74) | -43.06  (-48.67 to -35.49) | -0.03  (-0.03 to -0.02) |
| Samoa | 737 (559 to 968) | 283032.19 (214740.84 to 371520.98) | 830 (625 to 1092) | 284726.26 (214460.41 to 374442.69) | 12.64  (3.37 to 24.84) | -0.04  (-0.07 to -0.01) |
| Senegal | 67626 (51466 to 86455) | 263593.96 (200605.97 to 336984.08) | 81359 (61230 to 106845) | 232988.89 (175347.1 to 305974.27) | 20.31  (-2.27 to 40.07) | -0.63  (-0.73 to -0.54) |
| Serbia | 20619 (15630 to 27211) | 204157.78 (154759.01 to 269429.19) | 12881 (9635 to 16921) | 204527.29 (152987.33 to 268680.57) | -37.53  (-43.42 to -30.13) | 0  (-0.01 to 0.02) |
| Sierra Leone | 22581 (17037 to 29129) | 192973.68 (145601.49 to 248939.74) | 40220 (30417 to 51813) | 191810.26 (145063.04 to 247099.29) | 78.12  (59.42 to 98.19) | -0.18  (-0.27 to -0.08) |
| Solomon Islands | 3042 (2309 to 3958) | 282397.93 (214403.38 to 367413.43) | 4504 (3444 to 5885) | 285388.54 (218232.94 to 372880.14) | 48.08  (33.14 to 66.39) | -0.03  (-0.05 to 0) |
| Somalia | 70630 (54475 to 91502) | 306690.26 (236541.28 to 397319.19) | 199755 (152316 to 257178) | 306037.56 (233357.69 to 394013.05) | 182.82  (154.22 to 215.28) | 0  (-0.01 to 0.01) |
| South Africa | 232704 (177831 to 303359) | 317284.25 (242466.3 to 413620.74) | 243590 (187556 to 317233) | 312666.6 (240741.91 to 407193.21) | 4.68  (0.3 to 9.75) | -0.05  (-0.06 to -0.04) |
| South Sudan | 57759 (44307 to 75520) | 308230.38 (236444.02 to 403015.94) | 83193 (63439 to 108348) | 307039.48 (234134.77 to 399880.82) | 44.04  (27.77 to 59.11) | -0.01  (-0.02 to -0.01) |
| Sri Lanka | 93162 (70753 to 121869) | 343877.17 (261159.51 to 449837.67) | 77557 (59051 to 103615) | 342894.26 (261076.25 to 458101.64) | -16.75  (-24.69 to -6.63) | -0.02  (-0.02 to -0.01) |
| Sudan | 243838 (187406 to 324964) | 335595.49 (257928.16 to 447249.48) | 297766 (224375 to 389237) | 336051.9 (253224.06 to 439283.57) | 22.12  (8.28 to 36.12) | 0  (-0.01 to 0.01) |
| Suriname | 2456 (1864 to 3219) | 358846.24 (272241.14 to 470310.43) | 2420 (1833 to 3170) | 356339.5 (269835.6 to 466711.04) | -1.47  (-11.26 to 8.8) | -0.02  (-0.04 to -0.01) |
| Syrian Arab Republic | 128447 (97573 to 167731) | 338086.56 (256823.33 to 441485.79) | 59818 (45259 to 78814) | 337471.71 (255335.72 to 444641.8) | -53.43  (-57.94 to -48.73) | 0  (-0.01 to 0.01) |
| Tajikistan | 15675 (11547 to 20809) | 101438.61 (74720.85 to 134657.87) | 19390 (14269 to 25664) | 100700.78 (74109.09 to 133287.95) | 23.69  (11.73 to 37.2) | -0.16  (-0.21 to -0.11) |
| Thailand | 425657 (319969 to 556308) | 529425.91 (397973 to 691927.17) | 225691 (171729 to 291294) | 506125.99 (385113.67 to 653245.81) | -46.98  (-52.32 to -40.72) | -0.11  (-0.14 to -0.07) |
| Timor-Leste | 8505 (6521 to 11118) | 316903.4 (242981.06 to 414301.14) | 9138 (6864 to 12066) | 314867.94 (236512.97 to 415788.09) | 7.44  (-3.37 to 21.45) | -0.05  (-0.06 to -0.04) |
| Togo | 31053 (23657 to 39912) | 254206.68 (193664.03 to 326731.62) | 45094 (34038 to 59209) | 253696.66 (191495.22 to 333104.15) | 45.22  (27.78 to 61.91) | -0.4  (-0.53 to -0.26) |
| Tonga | 559 (423 to 734) | 282861.98 (214212.06 to 371648.51) | 507 (386 to 668) | 285318.87 (217236.24 to 376110.12) | -9.24  (-17.78 to 0.33) | -0.04  (-0.07 to -0.01) |
| Tunisia | 60958 (46391 to 78826) | 340829.09 (259379.57 to 440732.26) | 43115 (32792 to 56001) | 339163.71 (257955.03 to 440534.76) | -29.27  (-36 to -21.86) | -0.01  (-0.01 to 0) |
| Turkey | 532489 (402499 to 702998) | 429809.99 (324886.43 to 567440.18) | 318676 (241885 to 419482) | 428158.22 (324985.56 to 563595.94) | -40.15  (-45.82 to -33.85) | -0.04  (-0.08 to 0) |
| Turkmenistan | 14167 (10664 to 18525) | 143310.42 (107877.23 to 187403.85) | 12256 (9127 to 15839) | 144402.58 (107532.93 to 186618.66) | -13.49  (-21.98 to -3.7) | 0.01  (0 to 0.01) |
| Tuvalu | 61 (47 to 80) | 282992.35 (217567 to 369873.34) | 47 (36 to 60) | 284498.25 (215846.24 to 366936.28) | -23.57  (-31.5 to -14.78) | -0.04  (-0.07 to -0.02) |
| Uganda | 278119 (217104 to 357104) | 402297.65 (314040.86 to 516549.31) | 452463 (343375 to 597269) | 381446.51 (289480.56 to 503524.54) | 62.69  (41.01 to 86.39) | -0.24  (-0.3 to -0.18) |
| Ukraine | 113927 (87021 to 150112) | 227199.66 (173541.41 to 299362.86) | 67007 (51046 to 87660) | 225689.92 (171928.87 to 295252.43) | -41.18  (-46.7 to -34.74) | 0.01  (-0.01 to 0.02) |
| United Republic of Tanzania | 325717 (254043 to 409833) | 358146.26 (279335.97 to 450636.23) | 519268 (393770 to 680406) | 334516.37 (253669.69 to 438323.22) | 59.42  (38.39 to 85.2) | -0.51  (-0.64 to -0.39) |
| Uzbekistan | 62873 (46297 to 82819) | 116630.2 (85881.61 to 153630.18) | 63382 (46908 to 82389) | 116842.78 (86473.08 to 151880.85) | 0.81  (-9.74 to 13.51) | 0.12  (0.07 to 0.17) |
| Vanuatu | 1213 (912 to 1576) | 281276.9 (211470.59 to 365364.97) | 1657 (1261 to 2160) | 283598.27 (215850.34 to 369832.24) | 36.56  (23.57 to 53.55) | -0.04  (-0.07 to -0.01) |
| Venezuela (Bolivarian Republic of) | 143577 (109153 to 188877) | 332020.71 (252416.22 to 436777.18) | 118308 (90724 to 154200) | 328855.85 (252181.16 to 428624.13) | -17.6  (-25.76 to -7.99) | -0.05  (-0.06 to -0.05) |
| Viet Nam | 387619 (296265 to 506225) | 280857.96 (214665.38 to 366797.05) | 280069 (210670 to 364960) | 275862.96 (207506.83 to 359479.92) | -27.75  (-35.55 to -20.22) | 0.12  (0.05 to 0.19) |
| Yemen | 207380 (167154 to 259337) | 410414.34 (330804.65 to 513239.26) | 283858 (216902 to 370650) | 403024.36 (307959.91 to 526251.49) | 36.88  (15.3 to 62.56) | -0.1  (-0.12 to -0.07) |
| Zambia | 125549 (91534 to 176468) | 436926.41 (318548.65 to 614130.01) | 164365 (129612 to 210760) | 355479.29 (280318.49 to 455821.45) | 30.92  (-16.28 to 62.15) | -1.71  (-2.07 to -1.35) |
| Zimbabwe | 89471 (67873 to 116385) | 310242.1 (235352.08 to 403565.55) | 102836 (78378 to 133912) | 307367.37 (234265.82 to 400251.63) | 14.94  (4.65 to 26.43) | -0.63  (-0.95 to -0.3) |

CI, confidence interval; EAPC, estimated annual percentage change; LMICs, low- and middle-income countries and territories; UI, uncertain interval.

## Supplementary Table S21. National burden and trends of incident cases and incidence rates (per 100,000 liveborn neonates) of varicella and herpes zoster in 131 LMICs between 1990 and 2019.

| **Location** | **Incident cases in 1990**  **(95% UI)** | **Incident rate in 1990**  **(95% UI)** | **Incident cases in 2019**  **(95% UI)** | **Incident rate in 2019**  **(95% UI)** | **Percent change**  **(%, 95% CI)** | **EAPC**  **(%, 95% CI)** |
| --- | --- | --- | --- | --- | --- | --- |
| Global | 2354253 (1270562 to 4031560) | 22802.45 (12306.2 to 39048.24) | 2088387 (1110595 to 3614224) | 20437.61 (10868.63 to 35369.93) | -11.29  (-15.48 to -7.74) | -0.35  (-0.37 to -0.34) |
| Afghanistan | 13082 (7299 to 22485) | 33812.58 (18865.54 to 58116.63) | 31543 (16475 to 54345) | 28204.03 (14730.52 to 48591.47) | 141.12  (97.36 to 191.68) | -0.77  (-0.88 to -0.67) |
| Albania | 1106 (562 to 1979) | 17002.79 (8638.22 to 30439.92) | 408 (211 to 722) | 15295.34 (7930.79 to 27085.5) | -63.13  (-68.94 to -57.06) | -0.36  (-0.39 to -0.32) |
| Algeria | 13001 (6694 to 22796) | 22385.15 (11525.25 to 39249.43) | 13796 (6910 to 24333) | 20803.28 (10419.58 to 36691.42) | 6.11  (-6.68 to 21.21) | -0.23  (-0.27 to -0.2) |
| American Samoa | 24 (12 to 42) | 18530.5 (9296.92 to 32365.57) | 15 (8 to 27) | 18720.14 (9407.69 to 33272.61) | -37.56  (-44.97 to -27.23) | -0.03  (-0.06 to 0.01) |
| Angola | 10721 (5724 to 18209) | 28291.35 (15105.83 to 48051.15) | 17049 (8666 to 29926) | 20449.04 (10394.26 to 35893.19) | 59.03  (23.46 to 98.95) | -1.21  (-1.26 to -1.16) |
| Armenia | 1028 (529 to 1821) | 17894.98 (9202.87 to 31708.9) | 478 (240 to 837) | 16712.24 (8402.21 to 29263.34) | -53.49  (-60.36 to -46.9) | -0.26  (-0.3 to -0.22) |
| Azerbaijan | 2703 (1343 to 4834) | 18898.03 (9391.81 to 33798.75) | 1920 (995 to 3308) | 17662.2 (9149.28 to 30434.72) | -28.97  (-40.28 to -17.46) | -0.28  (-0.31 to -0.24) |
| Bangladesh | 59935 (32399 to 105300) | 20475.32 (11068.25 to 35973.48) | 33509 (17110 to 60059) | 16493.28 (8421.57 to 29561.58) | -44.09  (-52.93 to -35.73) | -0.97  (-1.09 to -0.84) |
| Belarus | 1819 (932 to 3161) | 16962.77 (8690.26 to 29477.31) | 1256 (643 to 2204) | 15980.06 (8178.11 to 28050.57) | -30.97  (-40.36 to -18.51) | -0.26  (-0.29 to -0.23) |
| Belize | 95 (48 to 166) | 21259.1 (10805.39 to 37194.59) | 118 (60 to 208) | 20376.68 (10379.1 to 35715.58) | 25.11  (8.59 to 43.88) | -0.11  (-0.14 to -0.08) |
| Benin | 4086 (2159 to 6964) | 22964.3 (12135.28 to 39142.43) | 7718 (3979 to 13501) | 20408.17 (10520.53 to 35701.86) | 88.89  (63.62 to 117.57) | -0.4  (-0.44 to -0.37) |
| Bhutan | 335 (174 to 581) | 20384.48 (10576.36 to 35357.03) | 161 (80 to 282) | 15892.98 (7868.36 to 27872.24) | -51.92  (-58.77 to -42.81) | -0.96  (-1.02 to -0.89) |
| Bolivia (Plurinational State of) | 4849 (2576 to 8497) | 26551.47 (14104.97 to 46528.62) | 5209 (2774 to 8930) | 21350.23 (11372.45 to 36603.41) | 7.42  (-9.19 to 26.5) | -0.49  (-0.6 to -0.39) |
| Bosnia and Herzegovina | 864 (433 to 1535) | 16713.82 (8379.61 to 29695.77) | 319 (163 to 580) | 15572.13 (7964.68 to 28333.68) | -63.14  (-68.55 to -54.84) | -0.38  (-0.44 to -0.31) |
| Botswana | 603 (318 to 1036) | 19606.07 (10341.99 to 33717.82) | 632 (324 to 1132) | 17374.38 (8893.29 to 31095.67) | 4.94  (-11.71 to 24.21) | -0.34  (-0.37 to -0.31) |
| Brazil | 68907 (36542 to 120322) | 26549.25 (14079.26 to 46359.27) | 61243 (31793 to 107145) | 26063.1 (13530.33 to 45597.8) | -11.12  (-17.22 to -4.41) | -0.02  (-0.07 to 0.04) |
| Bulgaria | 1193 (605 to 2140) | 15896.11 (8060.07 to 28514.5) | 704 (372 to 1259) | 15385.75 (8127.8 to 27498.82) | -40.95  (-48.74 to -32.42) | -0.13  (-0.15 to -0.1) |
| Burkina Faso | 8099 (4146 to 13938) | 23626.44 (12093.99 to 40662.91) | 14477 (7480 to 25894) | 20525.06 (10605.41 to 36711.49) | 78.76  (51.53 to 110.39) | -0.47  (-0.54 to -0.39) |
| Burundi | 5096 (2764 to 8936) | 25761.86 (13970.99 to 45171.67) | 7593 (3978 to 13013) | 21650.27 (11343.48 to 37102.85) | 49  (23.94 to 81.68) | -0.71  (-0.81 to -0.61) |
| Cabo Verde | 168 (85 to 298) | 18920.04 (9562.41 to 33539.26) | 137 (70 to 241) | 16767.08 (8590.48 to 29593.93) | -18.88  (-31.24 to -4.49) | -0.5  (-0.53 to -0.47) |
| Cambodia | 5479 (2948 to 9592) | 16713.34 (8993.58 to 29260.71) | 3792 (1986 to 6711) | 13684.87 (7165.55 to 24217.87) | -30.79  (-41.41 to -17.22) | -0.77  (-0.82 to -0.71) |
| Cameroon | 7917 (4033 to 13894) | 22721.44 (11573.61 to 39873.56) | 13267 (6707 to 23454) | 19517.2 (9866.65 to 34503.96) | 67.57  (40.85 to 99.11) | -0.56  (-0.63 to -0.48) |
| Central African Republic | 2679 (1429 to 4674) | 28482.22 (15189.01 to 49693.83) | 4054 (2182 to 7209) | 27512.04 (14803.7 to 48918.13) | 51.34  (27.52 to 74.88) | -0.25  (-0.32 to -0.18) |
| Chad | 5966 (3050 to 10449) | 25167.92 (12868.4 to 44080.7) | 13484 (6860 to 23459) | 22792.25 (11596.24 to 39652.98) | 126.03  (98.8 to 167.68) | -0.33  (-0.42 to -0.24) |
| China | 439573 (235402 to 764932) | 24137.78 (12926.37 to 42003.89) | 236902 (123923 to 414958) | 20830.06 (10896.16 to 36486.03) | -46.11  (-51.65 to -40.5) | -0.62  (-0.68 to -0.57) |
| Colombia | 19110 (9722 to 33753) | 27091.05 (13782.24 to 47849.1) | 14645 (7590 to 26296) | 24318.96 (12603.06 to 43666.15) | -23.36  (-33.62 to -14.51) | -0.35  (-0.37 to -0.32) |
| Comoros | 362 (184 to 632) | 24590.97 (12527.8 to 42986.6) | 249 (130 to 443) | 19993.65 (10435.85 to 35633.9) | -31.3  (-42.42 to -19.74) | -0.82  (-0.88 to -0.76) |
| Congo | 1795 (933 to 3101) | 23911.94 (12430.63 to 41311.26) | 2156 (1108 to 3761) | 19853.25 (10205.08 to 34634.01) | 20.11  (3.03 to 39.98) | -0.81  (-0.91 to -0.7) |
| Costa Rica | 1545 (792 to 2713) | 25075.91 (12860.64 to 44054.11) | 1202 (612 to 2099) | 23749.52 (12090.57 to 41486.22) | -22.2  (-29.74 to -13.55) | -0.18  (-0.21 to -0.15) |
| Côte d'Ivoire | 9471 (4823 to 16810) | 22228.2 (11319.36 to 39454.71) | 13712 (7153 to 24570) | 20699.78 (10797.67 to 37089.99) | 44.79  (25.06 to 64.51) | -0.33  (-0.37 to -0.29) |
| Cuba | 2620 (1330 to 4743) | 18990.02 (9639.03 to 34371.9) | 1462 (740 to 2571) | 18314.37 (9269.47 to 32206.39) | -44.2  (-51.33 to -36.86) | -0.11  (-0.12 to -0.09) |
| Democratic People's Republic of Korea | 11010 (5635 to 19596) | 20298.09 (10389.19 to 36126.24) | 5588 (2803 to 9792) | 21389.18 (10729.06 to 37482.65) | -49.25  (-55.1 to -42.17) | 0.15  (0.08 to 0.23) |
| Democratic Republic of the Congo | 34957 (18496 to 60720) | 24956.48 (13204.62 to 43349.02) | 47708 (24695 to 83741) | 21681.21 (11222.97 to 38056.9) | 36.47  (9.79 to 56.3) | -0.48  (-0.54 to -0.42) |
| Djibouti | 365 (192 to 624) | 22034.33 (11579.95 to 37636.49) | 499 (251 to 854) | 19126.07 (9622.95 to 32751.55) | 36.51  (15.46 to 60.71) | -0.73  (-0.83 to -0.63) |
| Dominica | 27 (14 to 46) | 20508.53 (10557.92 to 35818.29) | 13 (7 to 22) | 20605.78 (10598.73 to 35649.58) | -51.11  (-55.56 to -46.18) | 0.04  (0.01 to 0.06) |
| Dominican Republic | 4063 (2013 to 7127) | 22553.41 (11171.29 to 39564.15) | 3513 (1772 to 6257) | 20347.62 (10266.68 to 36244.75) | -13.54  (-25.58 to 0.3) | -0.26  (-0.31 to -0.22) |
| Ecuador | 5078 (2592 to 8571) | 22416.19 (11443.35 to 37837.9) | 5204 (2693 to 9013) | 19597.06 (10141.4 to 33938.09) | 2.49  (-12.33 to 17.18) | -0.4  (-0.47 to -0.34) |
| Egypt | 35002 (18308 to 60196) | 24637.59 (12886.63 to 42371.46) | 35452 (18311 to 63244) | 22019.05 (11372.64 to 39280.07) | 1.29  (-14.05 to 21.6) | -0.35  (-0.39 to -0.31) |
| El Salvador | 3664 (1897 to 6326) | 29585.47 (15318.47 to 51072.72) | 2137 (1111 to 3767) | 24975.99 (12985.01 to 44031.13) | -41.69  (-50.82 to -34.58) | -0.52  (-0.6 to -0.45) |
| Equatorial Guinea | 538 (272 to 941) | 31450.99 (15888.79 to 55041.14) | 531 (259 to 946) | 18184.88 (8868.41 to 32383.99) | -1.25  (-27.47 to 28.18) | -2.11  (-2.36 to -1.86) |
| Eritrea | 2899 (1564 to 5028) | 31087.73 (16772.31 to 53916.54) | 3248 (1641 to 5606) | 21633.02 (10932.09 to 37337.45) | 12.04  (-10.02 to 38.75) | -1.13  (-1.22 to -1.05) |
| Eswatini | 501 (262 to 840) | 20868.03 (10909.11 to 35004.54) | 435 (217 to 766) | 19580.59 (9755.16 to 34522.52) | -13.25  (-25.62 to -0.3) | -0.09  (-0.23 to 0.04) |
| Ethiopia | 55910 (30820 to 95112) | 30356.4 (16733.89 to 51641.38) | 59866 (31396 to 103698) | 21767.22 (11415.79 to 37704.73) | 7.08  (-12.64 to 25.45) | -1.22  (-1.27 to -1.17) |
| Fiji | 284 (147 to 487) | 19683.04 (10185.51 to 33794.01) | 262 (133 to 458) | 19725.05 (10017.29 to 34470.67) | -7.49  (-19.78 to 5.81) | -0.04  (-0.06 to -0.03) |
| Gabon | 622 (307 to 1099) | 21071.13 (10408.11 to 37210.64) | 593 (284 to 1053) | 18408.34 (8814.79 to 32682.69) | -4.7  (-20.33 to 9.11) | -0.46  (-0.49 to -0.42) |
| Gambia | 782 (412 to 1366) | 21700.01 (11443.09 to 37919.4) | 1078 (528 to 1866) | 20100.24 (9847.4 to 34773.06) | 37.94  (19.33 to 59.93) | -0.24  (-0.27 to -0.21) |
| Georgia | 1090 (541 to 1973) | 17250.9 (8553.6 to 31225.58) | 600 (314 to 1076) | 17278.52 (9034.58 to 30988.49) | -44.96  (-52.3 to -36.91) | 0.06  (0.01 to 0.1) |
| Ghana | 9517 (4997 to 16147) | 21057.71 (11056.36 to 35728.93) | 12120 (6218 to 21195) | 18867.12 (9679.74 to 32995.46) | 27.35  (9.78 to 53.93) | -0.29  (-0.33 to -0.26) |
| Grenada | 32 (16 to 56) | 21213.03 (10650.36 to 37394.53) | 21 (11 to 38) | 20028.34 (10167.89 to 36308.29) | -33.77  (-41.07 to -25.45) | -0.14  (-0.18 to -0.1) |
| Guatemala | 8434 (4457 to 14713) | 33199.88 (17545.3 to 57917.9) | 8559 (4351 to 15293) | 27350.42 (13904.9 to 48872.65) | 1.48  (-12.67 to 17.44) | -0.67  (-0.76 to -0.57) |
| Guinea | 5938 (3103 to 10597) | 25348.86 (13247.56 to 45237.75) | 7924 (4116 to 13851) | 22033.59 (11444.36 to 38517.14) | 33.44  (12.08 to 55.71) | -0.46  (-0.51 to -0.41) |
| Guinea-Bissau | 923 (493 to 1610) | 26674.39 (14229.66 to 46503.01) | 1045 (537 to 1807) | 22330.58 (11464.9 to 38601.88) | 13.18  (-6.12 to 38.57) | -0.62  (-0.69 to -0.56) |
| Guyana | 382 (197 to 673) | 22898.12 (11814.51 to 40326.84) | 233 (117 to 421) | 21517.92 (10789.65 to 38864.62) | -39  (-45.83 to -30.8) | -0.12  (-0.15 to -0.08) |
| Haiti | 5585 (3012 to 9385) | 30237.99 (16309.61 to 50813.31) | 6191 (3378 to 10508) | 24905.25 (13587.96 to 42272.53) | 10.85  (-4.62 to 31) | -0.6  (-0.63 to -0.57) |
| Honduras | 4243 (2212 to 7367) | 31280.43 (16306.26 to 54318.29) | 4884 (2474 to 8737) | 27386.14 (13872.54 to 48991.52) | 15.12  (-0.64 to 30.55) | -0.44  (-0.47 to -0.41) |
| India | 487430 (256769 to 824443) | 24826.71 (13078.26 to 41992.09) | 377909 (200135 to 643651) | 21318.91 (11290.21 to 36310.19) | -22.47  (-28.39 to -16.76) | -0.25  (-0.36 to -0.13) |
| Indonesia | 56150 (28790 to 97905) | 15650.67 (8024.72 to 27288.81) | 43828 (22806 to 77756) | 15048.15 (7830.26 to 26697.01) | -21.94  (-26.75 to -17.57) | -0.08  (-0.11 to -0.05) |
| Iran (Islamic Republic of) | 31381 (16462 to 54143) | 23337.69 (12242.96 to 40265.96) | 22326 (11446 to 38854) | 21769.64 (11160.59 to 37887.04) | -28.86  (-33.29 to -23.49) | -0.21  (-0.24 to -0.18) |
| Iraq | 12248 (6485 to 21162) | 23179.63 (12272.91 to 40047.7) | 15400 (7933 to 26879) | 21168.03 (10903.81 to 36945.21) | 25.73  (7.75 to 45.74) | -0.4  (-0.44 to -0.35) |
| Jamaica | 845 (428 to 1523) | 19899.2 (10082.33 to 35848.79) | 535 (264 to 943) | 19535.73 (9646.46 to 34423.05) | -36.71  (-43.96 to -27.09) | -0.05  (-0.08 to -0.01) |
| Jordan | 2296 (1173 to 4015) | 22242.22 (11365.7 to 38902.26) | 3812 (1962 to 6516) | 20397.93 (10501.15 to 34870.16) | 66.04  (41.57 to 105.28) | -0.37  (-0.39 to -0.35) |
| Kazakhstan | 4867 (2441 to 8922) | 18071.4 (9062.15 to 33126.6) | 4551 (2361 to 8089) | 16934.01 (8786.48 to 30099.95) | -6.5  (-19.44 to 8.44) | -0.33  (-0.39 to -0.28) |
| Kenya | 16773 (8776 to 29025) | 22289.08 (11662.23 to 38569.74) | 21629 (11325 to 37786) | 21323.91 (11165.31 to 37253.24) | 28.95  (22.56 to 35.38) | -0.14  (-0.18 to -0.09) |
| Kiribati | 47 (25 to 81) | 24437.58 (13069.78 to 41943.36) | 53 (27 to 90) | 23021.14 (11739.12 to 39408.44) | 11.03  (-3.44 to 27.76) | -0.2  (-0.24 to -0.16) |
| Kyrgyzstan | 1789 (919 to 3133) | 18808.6 (9656.02 to 32929.86) | 1950 (1013 to 3364) | 17655.34 (9173.36 to 30451.85) | 9  (-5.01 to 25.65) | -0.25  (-0.27 to -0.24) |
| Lao People's Democratic Republic | 2297 (1230 to 3868) | 18563.49 (9939.51 to 31257.6) | 1882 (996 to 3253) | 14547.98 (7696.36 to 25147.67) | -18.07  (-30.62 to -5.25) | -0.89  (-0.92 to -0.87) |
| Lebanon | 1763 (909 to 3071) | 21238.03 (10947.77 to 37000.39) | 1548 (790 to 2661) | 19296.03 (9848.91 to 33169.2) | -12.18  (-23.5 to 1.13) | -0.38  (-0.4 to -0.35) |
| Lesotho | 979 (524 to 1660) | 21615.15 (11573.35 to 36640.32) | 732 (389 to 1289) | 21009.38 (11174.26 to 37012.24) | -25.26  (-33.63 to -14.01) | 0  (-0.12 to 0.12) |
| Liberia | 1453 (749 to 2590) | 24233.54 (12499.1 to 43195.9) | 2044 (1055 to 3487) | 20195.82 (10422.73 to 34453.91) | 40.68  (16.99 to 70.64) | -0.68  (-0.78 to -0.59) |
| Libya | 2490 (1273 to 4287) | 22010.31 (11247.32 to 37885.77) | 1317 (672 to 2285) | 21199.54 (10817.49 to 36795.93) | -47.14  (-55.06 to -39.12) | -0.19  (-0.23 to -0.15) |
| Madagascar | 9456 (5092 to 16209) | 23696.85 (12759.03 to 40618.7) | 13742 (6930 to 23936) | 21308.56 (10745.65 to 37115.47) | 45.32  (17.4 to 70.62) | -0.32  (-0.41 to -0.23) |
| Malawi | 7931 (4100 to 13781) | 22805.58 (11789.18 to 39628.78) | 8386 (4295 to 14646) | 20310.5 (10401.03 to 35471.69) | 5.75  (-8.96 to 20.89) | -0.51  (-0.57 to -0.45) |
| Malaysia | 4808 (2494 to 8522) | 12724.52 (6601.09 to 22553.94) | 4932 (2538 to 8675) | 11921.3 (6135.28 to 20966.79) | 2.58  (-9 to 17.77) | -0.25  (-0.3 to -0.21) |
| Maldives | 90 (47 to 157) | 13591.29 (7077.14 to 23659.75) | 74 (38 to 133) | 11553.94 (5887.16 to 20808.55) | -17.68  (-27.89 to -4.24) | -0.63  (-0.69 to -0.58) |
| Mali | 8235 (4222 to 14217) | 25068.18 (12853.07 to 43276.98) | 14936 (7799 to 26211) | 20713.32 (10815.61 to 36350.21) | 81.37  (53.47 to 115.15) | -0.63  (-0.74 to -0.52) |
| Marshall Islands | 25 (13 to 42) | 21527.85 (11339.17 to 36647.16) | 20 (10 to 34) | 21105.82 (10968.4 to 36174.94) | -20.17  (-31.62 to -10.44) | -0.13  (-0.18 to -0.09) |
| Mauritania | 1509 (770 to 2590) | 22634.31 (11544.09 to 38853.73) | 1542 (806 to 2740) | 18691.82 (9765.74 to 33205.23) | 2.21  (-13.63 to 18.31) | -0.61  (-0.64 to -0.59) |
| Mexico | 63812 (33119 to 111251) | 33991.16 (17641.74 to 59260.73) | 46188 (24447 to 80922) | 28987.96 (15343.2 to 50786.5) | -27.62  (-33.1 to -23.02) | -0.33  (-0.43 to -0.23) |
| Micronesia (Federated States of) | 57 (30 to 98) | 21665.82 (11282.37 to 37236.79) | 29 (15 to 51) | 19765.47 (10386.76 to 34528.13) | -48.65  (-56.09 to -39.77) | -0.3  (-0.33 to -0.28) |
| Mongolia | 1189 (615 to 2040) | 21582.17 (11170.35 to 37034.64) | 1136 (597 to 2023) | 18642.51 (9798.92 to 33201.42) | -4.46  (-21.6 to 14.44) | -0.66  (-0.72 to -0.6) |
| Montenegro | 116 (60 to 212) | 15273.07 (7883.77 to 27838.93) | 77 (39 to 136) | 14977.91 (7705.77 to 26595.51) | -34.12  (-44.09 to -23.58) | -0.1  (-0.13 to -0.08) |
| Morocco | 14905 (7767 to 25969) | 25073.67 (13065.8 to 43683.87) | 10118 (5270 to 17199) | 22164.37 (11544.58 to 37676.97) | -32.12  (-41.38 to -18.19) | -0.43  (-0.44 to -0.41) |
| Mozambique | 12041 (6508 to 21341) | 27579.86 (14906.03 to 48879.87) | 17807 (9466 to 29844) | 21070.58 (11200.74 to 35313.61) | 47.88  (19.23 to 82.71) | -0.87  (-0.95 to -0.8) |
| Myanmar | 16293 (8567 to 28484) | 16418.2 (8633.49 to 28703.37) | 10971 (5806 to 19753) | 13918.26 (7365.6 to 25060.4) | -32.66  (-40.7 to -20.65) | -0.66  (-0.7 to -0.62) |
| Namibia | 799 (424 to 1414) | 21921.08 (11642.55 to 38769.03) | 857 (410 to 1538) | 18123.65 (8681.48 to 32520.94) | 7.21  (-10.65 to 28.42) | -0.66  (-0.72 to -0.6) |
| Nepal | 12643 (6737 to 21301) | 20846.55 (11109.18 to 35122.88) | 7907 (4145 to 13986) | 17010.53 (8917.45 to 30089.58) | -37.46  (-46.19 to -28.02) | -0.66  (-0.73 to -0.59) |
| Nicaragua | 3136 (1629 to 5676) | 28872.49 (14997.08 to 52260.27) | 2482 (1282 to 4400) | 24906.52 (12862.68 to 44152.5) | -20.85  (-31.95 to -9.86) | -0.54  (-0.57 to -0.51) |
| Niger | 8768 (4660 to 15295) | 26381.01 (14020.49 to 46020.71) | 18772 (9727 to 32628) | 21734.74 (11262.79 to 37777.64) | 114.1  (82.87 to 157.65) | -0.76  (-0.81 to -0.7) |
[truncated: 217,094 more chars]
